# A mechanistic framework for the recognition of chemically diverse brassinosteroids by BRI1-family receptor kinases

**DOI:** 10.1038/s41477-026-02346-0

**Published:** 2026-08-05

**Authors:** Alberto Caregnato, Houming Chen, Miroslav Kvasnica, Ulrich Hohmann, Jana Oklestkova, Karoll Ferrer, Larissa Broger, Ludwig A. Hothorn, Miroslav Strnad, Michael Hothorn

**Affiliations:** 1 Structural Plant Biology Laboratory, Department of Plant Sciences, University of Geneva, Geneva, Switzerland; 2 Laboratory of Growth Regulators, Faculty of Science, Palacký University & Institute of Experimental Botany, The Czech Academy of Sciences, Olomouc, Czech Republic; 3 Leibniz University Hannover, Hanover, Germany; 4 Present Address: Institute of Molecular Biology, Mainz, Germany

**Keywords:** Brassinosteroid, Plant signalling, X-ray crystallography, Biochemical assays, Genetic engineering

## Abstract

Brassinosteroids (BRs) are chemically diverse plant steroid hormones produced via a branched biosynthetic pathway. The potent BR brassinolide is sensed by the membrane receptor kinase BRI1 and a SERK co-receptor, but the physiological functions of other abundant BRs remain to be characterized. Here we present quantitative binding kinetics for 4 *Arabidopsis thaliana* BR receptors and 15 BRs, which define the key chemical features required for high-affinity receptor binding, ligand positioning and co-receptor recognition. BRI1, BRL1 and BRL3 share overlapping ligand preferences, whereas BRL2 binds C_28_ BRs with moderate affinity. Structural analyses of BR-bound BRI1 and BRL3 ectodomains combined with extensive in vitro and in vivo mutagenesis studies reveal a high structural plasticity of the hormone-binding pocket. Functional assays using structure-based BR agonists and antagonists uncover that BR receptor–co-receptor signalling complexes can recognize chemically diverse BRs, introducing an additional, intriguing layer of BR signalling regulation.

## Abstract (web summary)

The ligand-binding spectrum of the four brassinosteroid receptors in the model plant *Arabidopsis* is investigated, revealing the key chemical features defining brassinosteroid bioactivity.

## Main

**Fig. 1:**
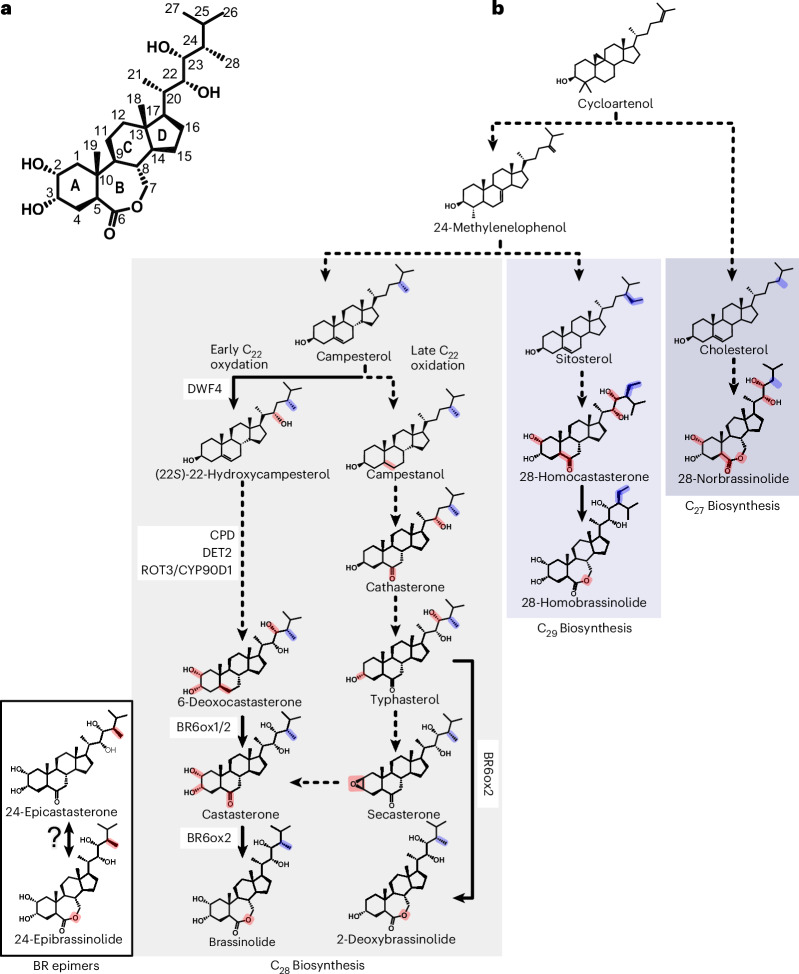
The branched architecture of the BR biosynthesis pathway. **a**, Chemical structure of brassinolide with labelled steroidal rings (letters) and carbon atoms (numbers). **b**, Schematic representation of the BR biosynthesis pathway, highlighting the relative positions of the BRs discussed in this study. The C_28_, C_29_ and C_27_ branches are shaded from light to dark grey, respectively. Key chemical differences between branches are indicated in blue, while red marks chemical modifications along each biosynthetic route. Solid lines denote single-step enzymatic reactions; dotted lines indicate multi-step conversions. Known enzymes involved in the canonical C_28_ pathway are shown alongside. Source data

Brassinosteroids (BRs) are a class of lipophilic, polyhydroxylated steroid hormones first isolated from rapeseed pollen and later recognized for their growth-promoting roles in plants^1^. BRs are synthesized via three main biosynthetic branches, producing C_27_, C_28_ or C_29_ compounds. While all share a steroidal ring core, they differ in the structure of their aliphatic side chains^2^ (Fig. 1a). The C_27_ branch, derived from cholesterol, yields 28-norbrassinolide^3,4^, whereas the C_29_ branch, from sitosterol, produces 28-homocastasterone and 28-homobrassinolide (Fig. 1b). The C_28_ pathway originates from campesterol and leads to brassinolide, one of the most bioactive BRs^2^ (Fig. 1b). The C_28_ pathway includes two routes: early and late C_22_ oxidation (Fig. 1b). In the early pathway, DWARF4 (DWF4), a cytochrome P450 monooxygenase, converts campesterol to (22*S*)-22-hydroxycampesterol^3,5,6^. Further steps involve the P450 enzymes CPD^7,8^ and ROT3^9^, and the steroid-5α-reductase DET2 (ref. ^10–12^), producing 6-deoxocastasterone (Fig. 1b). The enzymes BR6ox1 and BR6ox2 then convert this intermediate to castasterone^13^, and BR6ox2 can further catalyse formation of a seven-membered lactone ring from the C_6_ ketone, yielding brassinolide^13^ (Fig. 1b).

In the late C_22_ oxidation pathway, campestanol is processed into cathasterone and typhasterol^14^ (Fig. 1b). BR6ox2 can convert typhasterol into 2-deoxybrassinolide^15^, or alternatively, typhasterol may be converted to castasterone via secasterone, a 2,3-epoxy intermediate^16^ (Fig. 1b). The different branches of BR biosynthesis are likely interconnected^2^. BR homeostasis is regulated by several catabolic enzymes, including the P450 enzyme BAS1 (ref. ^17^), the UDP-glycosyltransferase UGT73C5 (ref. ^18^) and sulfotransferases^19^.

BR biosynthesis occurs, at least in part, within the endoplasmic reticulum^20,21^. Local BR transport is facilitated via plasmodesmata^22^. The ATP-binding cassette transporters ABCB1 and ABCB19 can translocate BRs across the plasma membrane into the apoplast^23^, in which BR perception takes place^24^.

BRs are perceived by the plasma membrane-localized receptor kinases BRASSINOSTEROID INSENSITIVE 1 (BRI1)^24–28^, BRL1^29–31^ and BRL3^29^, which share a conserved extracellular leucine-rich repeat (LRR) ligand-binding domain. BRI1 is broadly expressed, and *bri1* loss-of-function mutants show a characteristic dwarf phenotype^25^. BRL1 and BRL3 are present in vascular tissues and can rescue the growth defects of the weak *bri1-301* allele^32^, when expressed from the *BRI1* promoter^29^. Expression of the sequence-related receptor kinase BRL2 could not rescue the *bri1-301* mutant phenotype^29^, or a *bri1 brl1 brl3* triple mutant^33^. BRL2 is also known as VASCULAR HIGHWAY1 (VH1) and has been implicated in leaf vein patterning^34^.

BRs bind to a steroid-binding pocket formed by the LRR core and an ~70-amino-acid island domain within BRI1 or BRL1^26,27,31^. BR binding enables BRI1 to recruit SOMATIC EMBRYOGENESIS RECEPTOR KINASEs (SERKs) to form an active signalling complex^26,35,36^. In the absence of BRs, SERKs are sequestered by BAK1-INTERACTING RECEPTOR-LIKE KINASE (BIR) pseudokinases^37,38^. Assembly of the BRI1–BR–SERK complex activates the cytoplasmic kinase domains^39^, triggering a downstream signalling cascade that leads to dephosphorylation of the BR transcription factors BES1 and BZR1^40,41^.

Bioassays with synthetic, exogenously applied BRs have been performed across various plant species. In *Arabidopsis*, nanomolar levels of brassinolide promote hypocotyl elongation in both wild-type and *det2* mutant seedlings^42^. Brassinolide or 24-epibrassinolide applied at high nanomolar to micromolar concentrations inhibits root growth^43^, whereas low concentrations—high picomolar to low nanomolar—of 24-epibrassinolide or 24-epicastasterone stimulate root growth^44^.

Initial BR-binding assays used microsomal fractions from transgenic *Arabidopsis* expressing BRI1-GFP and [^3^H]-brassinolide, yielding estimated dissociation constants (*K*_d_) of ~10–15 nM for brassinolide and 40–75 nM for castasterone^24^. In the same assay, BRL1 and BRL3 bound brassinolide with *K*_d_ values of ~5 nM and ~50 nM, respectively, while BRL2 showed no detectable binding^29^. Grating-coupled interferometry (GCI) quantified binding of unlabelled brassinolide to BRI1 with a *K*_d_ of ~10 nM (ref. ^28^). Brassinolide-bound BRI1 enables the binding of the SERK3/BAK1 LRR ectodomain, with *K*_d_ values of ~0.2–1 μM, as determined by GCI and isothermal titration calorimetry^28,36^. Mutation of Gly644 to Asp (*bri1-6*/*bri1-119*)^45^ in the BRI1 island domain^26^ reduces BR binding in vitro^28,46^. The corresponding *bri1-6* allele shows intermediate growth defects^45^. Mutation of neighbouring Gly643 to Glu in the gain-of-function BRI1^*sud1*^ allele^47^ enhances the structural stability of the island domain^26^. Five simultaneous point mutations in the BRI1 BR-binding pocket were required to completely abolish brassinolide binding in vitro and to mimic the severe growth phenotype of *bri1* null alleles in vivo^46^.

The natural occurrence of different BRs has been characterized using high-performance liquid chromatography coupled with gas chromatography–mass spectrometry. In *Arabidopsis* shoots, castasterone, 6-deoxocastasterone and typhasterol were identified^11,48,49^. Like in other species, brassinolide accumulates more prominently in *Arabidopsis* seeds^50^. Brassinolide, the most bioactive BR^2^, is widely considered the primary signalling ligand for BRI1, BRL1 and BRL3. Consequently, previous structural and biochemical investigations have focused on brassinolide^24,26–29,31,36,46^. Given the chemical diversity of BRs from the C_27_, C_28_ and C_29_ biosynthetic branches that accumulate in plants, we set out to systematically characterize their binding kinetics to the ectodomains of the *Arabidopsis thaliana* BR receptors BRI1, BRL1, BRL2 and BRL3. By integrating these measurements with structural and mutational analyses of the BRI1-binding pocket—both in vitro and in planta—as well as targeted BR bioassays, we define the ligand-binding specificities of each receptor and uncover the chemical features in BRs required for receptor binding and co-receptor recruitment to form an active signalling complex.

## Results

### BRI1, BRL1 and BRL3 bind chemically diverse BRs with high affinity

**Fig. 2:**
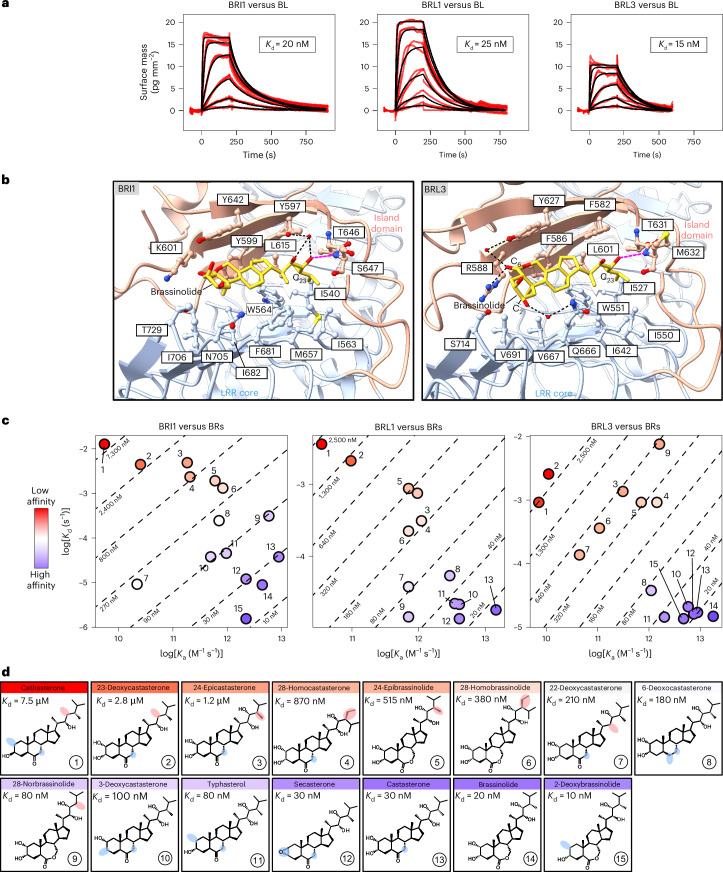
BRI1, BRL1 and BRL3 bind chemically diverse BRs. **a**, GCI analysis of ligand-binding kinetics between BRI1, BRL1, BRL3 and brassinolide. Shown are sensorgrams (*n* = 2, red) with global fits (black) and equilibrium *K*_d_ indicated. **b**, BR-binding pockets of BRI1 (left; PDB ID, 3RJ0) and BRL3 (right) shown as ribbon diagrams. LRRs are shown in blue, island domains in red and brassinolide in yellow. Pocket residues are shown as ball and sticks, hydrogen bonds as dotted lines. The conserved C_23_-OH hydrogen bond is highlighted in purple. **c**, Isoaffinity plots of association (*K*_a_) and dissociation (*K*_d_) rates for receptor–BR pairs. Each point (*n* = 2) reflects a global fit; diagonal lines denote *K*_d_. High- and low-affinity interactions are in blue and red, respectively. **d**, Structures of tested BRs, numbered as in **c**, with BRI1 *K*_d_ values. Red highlights indicate modifications that reduce binding, while blue highlights indicate modifications that have a minimal effect.

**Extended Data Fig. 1:**
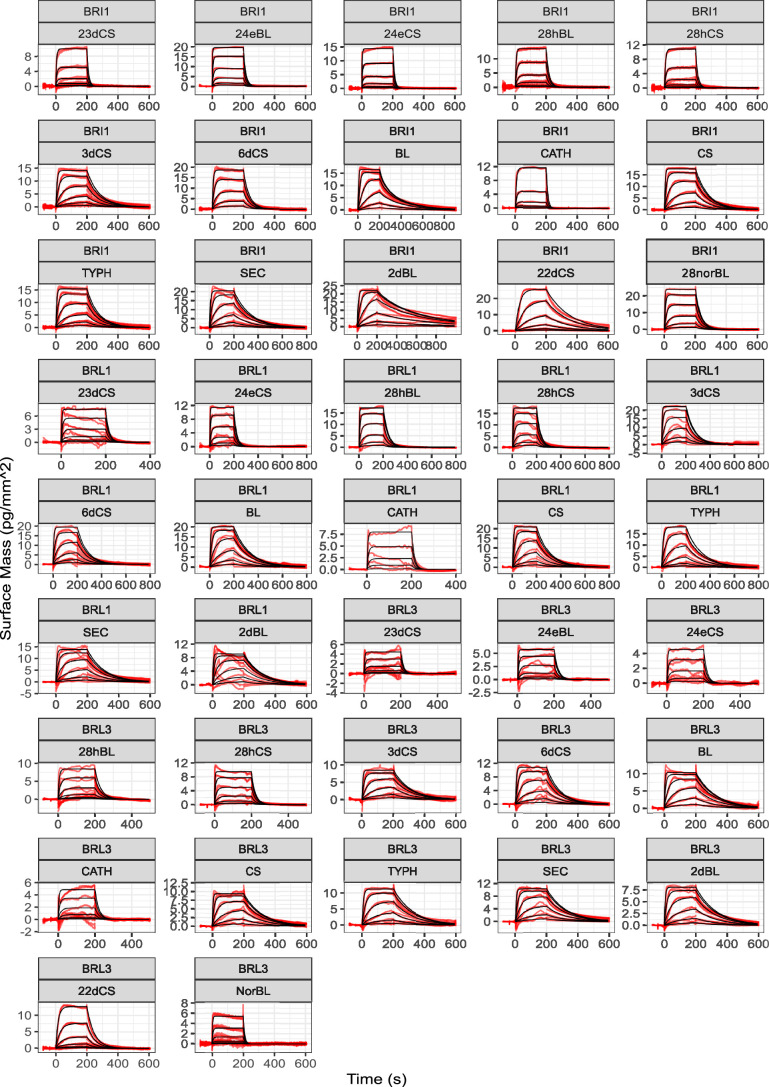
Complete set of GCI sensorgrams for the experiments described in Fig. 2a, c, d. Grating Coupling Interferometry (GCI)-derived binding kinetics for BRI1, BRL1, and BRL3 in response to different BRs. Raw sensorgrams are shown with experimental traces in red, and global fits as black lines. CATH: cathasterone, 23dCS: 23-deoxycastasterone, 24eCS: 24-epicastasterone, 28hCS: 28-homocastasterone, 24eBL: 24-epibrassinolide, 28hBL: 28-homobrassinolide, 22dCS: 22-deoxycastasterone, 6dCS: 6-deoxocastasterone, NorBL: 28-norbrassinolide, 3dCS: 3-deoxycastasterone, TYPH: typhasterol, CS: castasterone, SEC: secasterone, 2dBL: 2-deoxybrassinolide, BL: brassinolide.

**Extended Data Fig. 2:**
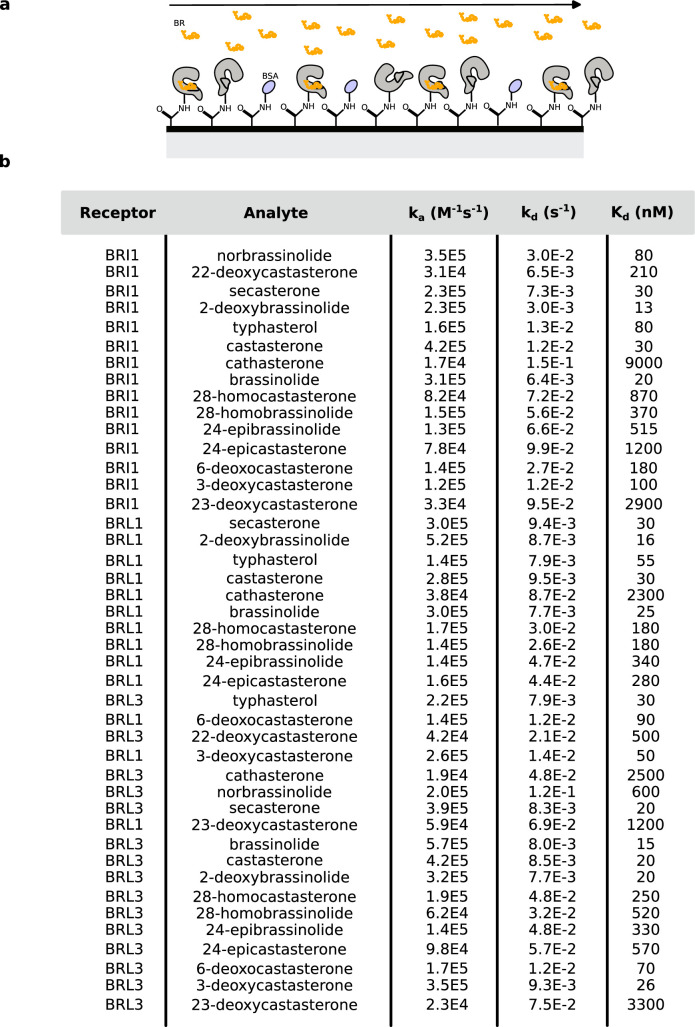
Complete set of GCI kinetic parameters for the experiments described in Fig. 2a, c, d. **a**, Schematic illustration of the Grating Coupling Interferometry (GCI) setup used to screen receptor - brassinosteroid interactions. **b**, Kinetic parameters obtained for BRI1, BRL1, and BRL3 in response to the 15 BRs analyzed in this study.

We obtained the LRR ectodomains of BRI1, BRL1 and BRL3 by secreted expression in baculovirus-infected insect cells, as described^26,28,46^. Quantitative GCI kinetic binding assays confirmed^28,29^ that BRI1, BRL1 and BRL3 bind brassinolide with nanomolar affinity (Fig. 2a, Extended Data Figs. 1 and 2). We then determined a crystal structure of the BRL3 ectodomain in complex with brassinolide at 2.45 Å resolution (Fig. 2b, Supplementary Figs. 1 and 2 and Supplementary Table 1). BRI1 (ref. ^26,27^), BRL1 (ref. ^31^) and BRL3 share a highly conserved ligand-binding pocket (root mean square deviation, r.m.s.d., is ~0.7 Å comparing 180 corresponding C_α_ atoms; Supplementary Fig. 1a), formed by conserved hydrophobic residues along the inner LRR surface and island domain (Fig. 2b). The brassinolide C_23_ hydroxyl forms a hydrogen bond with a main chain amino group in the island domain of all three receptors (Fig. 2b and Supplementary Fig. 1a). Few receptor-specific polar contacts with the ligand are observed. In BRI1, Tyr597 forms hydrogen bonds with the brassinolide C_22_ and C_23_ hydroxyls via a water molecule (Fig. 2b), whereas in BRL1 and BRL3, Tyr597 is replaced by phenylalanine. In the BRL3 complex, Arg588 (corresponding to Lys601 in BRI1) and Tyr627 form hydrogen bonds with the C_6_ hydroxyl group of brassinolide (Fig. 2b). BRL3 Gln666 (Phe681 in BRI1) coordinates the C_2_ hydroxyl via a water molecule (Fig. 2b). Additional water molecules that could potentially be involved in polar contacts between the receptor and the ligand may not be resolved in our medium-resolution complex structures (Supplementary Fig. 2 and Supplementary Table 1). Taken together, BRI1, BRL1 and BRL3 share a structurally conserved BR-binding pocket with limited receptor-specific features, and all bind brassinolide with high affinity.

Next, we quantified the interaction of BRI1, BRL1 and BRL3 with 12 natural BRs from the three biosynthetic branches, along with the synthetic compounds 3-deoxycastasterone, 22-deoxycastasterone and 23-deoxycastasterone (Fig. 2c,d). BRI1 showed binding constants ranging from low micromolar (red in *K*_a_/*K*_d_ plots, Fig. 2c) to low nanomolar (blue in Fig. 2c, Extended Data Figs. 1 and 2). Low-affinity ligands include cathasterone (an early C_28_ precursor), 24-epibrassinolide and 24-epicastasterone (late C_28_ branch), and the C_29_ products 28-homocastasterone and 28-homobrassinolide (Figs. 1b and 2c,d). 28-Norbrassinolide (C_27_ branch) as well as brassinolide and castasterone (late C_28_ branch) represent high-affinity ligands (Figs. 1b and 2c,d). Additional high-affinity binders include typhasterol, secasterone and 2-deoxybrassinolide (late C_22_ oxidation), as well as 6-deoxocastasterone (early C_22_ oxidation), all within the C_28_ branch (Figs. 1b and 2c,d). BRL1 and BRL3 share highly similar ligand-binding specificity profiles with BRI1 (Fig. 2c, Extended Data Figs. 1 and 2), consistent with their structurally conserved BR-binding pockets (Fig. 2b and Supplementary Fig. 1) and their ability to rescue *bri1-301* mutant phenotypes^29^.

We next sought to define the chemical features of BRs that contribute to high-affinity receptor binding. Side chain modifications—such as the C_24_ ethyl substitution in 28-homocastasterone or 28-homobrassinolide (Fig. 1a,b)—were associated with ~10- to 30-fold and ~10- to 35-fold reductions in binding affinity, respectively, compared to castasterone and brassinolide (Fig. 2c,d). Similarly, the brassinolide / castasterone enantiomers 24-epibrassinolide and 24-epicastasterone (Fig. 1a,b) showed ~10- to 20-fold and ~10- to 40-fold reduced affinities, respectively (Fig. 2c,d). The absence of a hydroxyl group at C_23_—as in cathasterone—correlated with weak receptor binding (Figs. 1b and 2c,d).

To further assess the role of side chain hydroxylation, we synthesized 23-deoxycastasterone (Supplementary Note 1). 23-Deoxycastasterone bound BRI1 with a *K*_d_ of ~3 μM, representing a 100-fold reduction compared to castasterone (Fig. 2c,d). By contrast, 22-deoxycastasterone—lacking the C_22_ hydroxyl group present in castasterone and brassinolide (Fig. 1b)—still bound BRI1 with medium to high affinity (*K*_d_ ≈ 200 nM; Fig. 2c,d). These results are consistent with our structural data showing that the C_23_, but not the C_22_, hydroxyl group forms conserved hydrogen bonds with the island domains of BRI1, BRL1 and BRL3 (Fig. 2b and Supplementary Fig. 1a).

Ring modifications were generally well tolerated by the BRI1, BRL1 and BRL3 ectodomains (Fig. 2c,d). Lactonization of the B-ring had minimal impact on binding, as brassinolide and castasterone showed nearly identical *K*_d_ (Fig. 2c,d). Although C_6_ oxidation is a key biosynthetic step towards brassinolide^13,51^, 6-deoxocastasterone bound all three receptors with high affinity (Fig. 2c,d). Supporting this, the complex structures of BRI1, BRL1 and BRL3 show no conserved interactions with the C_6_ ketone^26,27,31^ (Fig. 2b and Supplementary Fig. 1a). The 2α and 3α hydroxyl groups in the A-ring also appear dispensable for receptor binding, as ligands lacking either—such as 2-deoxybrassinolide, 3-deoxycastasterone, typhasterol and secasterone—bound BRI1, BRL1 and BRL3 with nanomolar affinity (Fig. 2c,d).

Overall, our quantitative binding data show that BR receptors recognize a surprisingly broad range of structurally diverse BRs with high affinity. The configuration and size of the aliphatic side chain, particularly the presence of a C_23_ hydroxyl group and the correct C_24_ stereochemistry, drive high-affinity receptor binding.

### High structural plasticity of the hormone-binding pocket enables BRI1/BRLs to sense different BRs

**Fig. 3:**
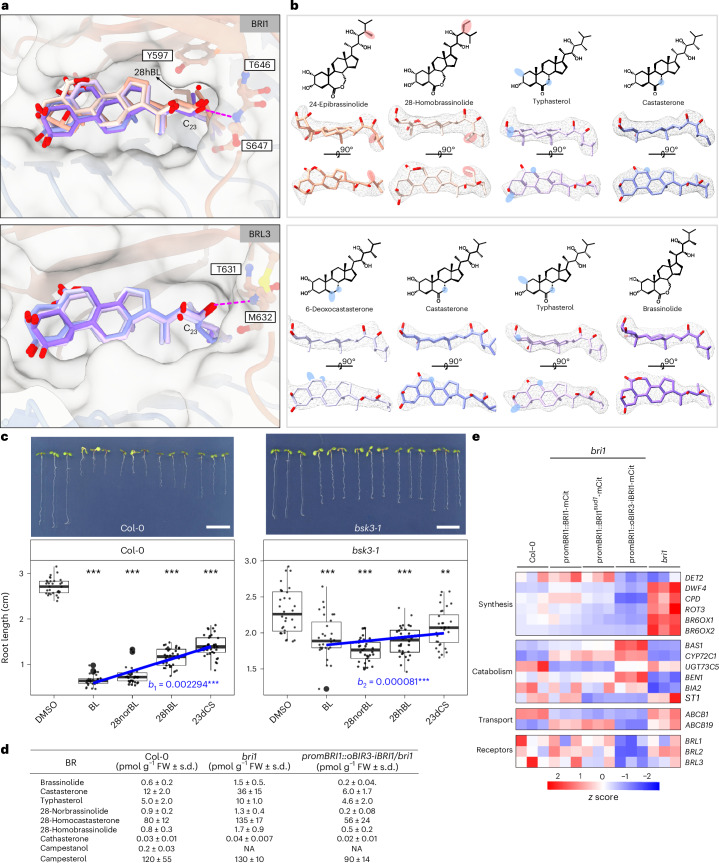
The hormone-binding pocket can accommodate structurally diverse BRs. **a**, Structural superposition of different BRI1–BR (top, r.m.s.d. of ~0.4 Å comparing 186 corresponding C_α_ atoms) and BRL3–BR complexes (bottom, r.m.s.d. of ~0.3 Å comparing 190 corresponding C_α_ atoms). The receptors are shown as a combined ribbon diagram (LRR domain in light blue, island domain in light red) and molecular surface views. BRs are in bonds representation colour coded according to their receptor binding affinities, ranging from low (light red) to high affinity (purple; BRI1, brassinolide complex PDB ID, 3RJ0). Dashed lines highlight key polar interactions among the ligand and the receptor-binding pockets. A conserved hydrogen bond between the ligand and the island domains of BRI1, BRL1 and BRL3 is marked in light orange. **b**, Structures of BRs (in bonds representation, colour coded as in **a** with BRI1 (top) or BRL3 (bottom) and including final mF_o_–DF_c_ omit electron density maps (grey mesh, contoured at 4 *σ*). The respective chemical structures are shown above, with modifications relative to brassinolide marked in red (for changes that reduce binding affinity) or blue (neutral). **c**, Root growth inhibition assay of 7-day-old Col-0 wild-type and *bsk3-1*^77^ mutant seedlings (*n* = 30, individual seedlings per condition) grown on ^1/2^MS plates supplemented with the indicated BR (BL, brassinolide; 28norBL, 28-norbrassinolide; 28hBL, 28-homobrassinolide; 23dCS, 23-deoxycastasterone; 0.1% (v/v) DMSO as control) at a final concentration of 100 nM (scale bars, 1 cm). Quantifications are shown as box plots (centre line, median; box, interquartile range (IQR); whiskers, lowest/highest data point within 1.5 IQR of the lower/upper quartile, raw data shown as grey dots). Multiple comparisons of the different treatments versus the DMSO control were performed using a two-sided Dunnett^89^ test as implemented in the R package multcomp^90^ (*P < 0.05; **P < 0.01; ***P < 0.001). The relationships between the estimated *K*_d_ for each ligand and root length were tested using a two-sided Tukey trend test^91,92^. The blue line represents the linear trend with slope (*b*) and the associated test *P* value (*P < 0.05; **P < 0.01; ***P < 0.001). The exact *P* values associated to *b*_1_ and *b*_2_ are 0.0000000 and 0.0000170, respectively. The experiment was repeated three times with similar results. **d**, BR quantification in 10-day-old Col-0, *bri1* (GABI_134E10)^47^ and *promBRI1*::*oBIR3-iBRI1 / bri1* (ref. ^52^) seedlings (FW, fresh weight; NA, not available). **e**, RNA-seq derived expression profile heat map of BR biosynthesis, catabolism, transport and receptor genes in 10-day-old seedlings in untreated BR signalling mutants and the Col-0 wild type. Expression values were transformed into *z* scores based on the normalized counts; three biological replicates are shown in three horizontal rows; the colour scale indicates expression level (red, up-regulation; blue, down-regulation).

To clarify the structural basis of ligand-binding specificity in BR receptors, we co-crystallized the BRI1 ectodomain with various BRs. Crystals grown with cathasterone, 24-epicastasterone or 28-homocastasterone showed no interpretable difference in electron density in the BR-binding pocket, while a BRI1–6-deoxocastasterone complex yielded poorly diffracting crystals. A 2.1 Å structure of the BRI1–castasterone complex revealed a ligand conformation nearly identical to that of brassinolide (Fig. 3a,b, Supplementary Fig. 2 and Supplementary Table 1), consistent with its high binding affinity (Fig. 2c,d) and confirming that B-ring lactonization is not essential for receptor recognition. Similarly, a 2.7 Å structure of the BRI1–typhasterol complex showed that the absence of a C_2_ hydroxyl group does not affect ligand coordination by the receptor (Fig. 3a,b and Supplementary Fig. 2). By contrast, the 2.4 Å BRI1–24-epibrassinolide complex structure showed weak electron density for the BR side chain, consistent with its reduced binding affinity (Figs. 2c,d and 3a,b and Supplementary Fig. 2). Finally, the 2.4 Å BRI1–28-homobrassinolide complex revealed that the receptor can in principle accommodate a C_24_ ethyl substitution in its binding pocket (Fig. 3a,b and Supplementary Fig. 2).

We next resolved the crystal structures of the BRL3 ectodomain bound to castasterone and typhasterol at 2.6 Å and 2.3 Å, respectively. In both structures, the ligands adopt conformations closely resembling that of brassinolide (Fig. 3a,b and Supplementary Fig. 2). A 3.2 Å structure of the BRL3–6-deoxocastasterone complex indicates that, despite lacking B-ring modifications, the ligand is accommodated in the binding pocket, albeit in a shifted conformation (Fig. 3a,b and Supplementary Fig. 2). In all BR receptor–ligand complexes analysed, the BR C_23_ hydroxyl group consistently forms a conserved hydrogen bond with the main chain amino group of Ser647 in BRI1 or Met632 in BRL1/BRL3 (Fig. 3a). We note that, given the moderate resolution of several datasets (∼2.5–3.2 Å), the level of detail observable in the electron density maps inherently limits the precision with which the ligand orientation can be defined.

Several catabolic enzymes contribute to BR homeostasis, including BAS1, which hydroxylates side chain C_26_ (ref. ^17^), UGT73C5, which glucosylates the hydroxyl group at C_23_ (ref. ^18^), and ST1/ST4a, which sulfonylates the C_22_ hydroxyl^19^. Structural analysis of our receptor–ligand complexes indicates that such tail modifications are not compatible with the BR-binding pocket (Supplementary Fig. 3). Together, the BRI1 and BRL3 ligand-bound structures rationalize the BR binding kinetics data, emphasizing the importance of the BR side chain for high-affinity receptor binding, while showing that modifications to the steroid ring core are generally tolerated.

To evaluate the physiological relevance of our biochemical and structural experiments, we selected ligands representing high-, medium- and low-affinity binders for root growth inhibition assays, as previously described^43^. Brassinolide (*K*_d_ ≈ 20 nM) and 28-norbrassinolide (*K*_d_ ≈ 80 nM) were tested as high-affinity binders, 28-homobrassinolide (*K*_d_ ≈ 380 nM) as medium-affinity and 23-deoxycastasterone (*K*_d_ ≈ 2,800 nM) as a low-affinity binder (Fig. 2d). Root growth inhibition assays were performed using each compound at a final concentration of 100 nM. A strong trend for increasing root length with increasing *K*_d_ was observed in treated wild-type seedlings and a weaker trend in the *bsk3-1* control (Fig. 3c), suggesting that in vivo BR bioactivities correlate well with their receptor binding affinity in vitro. Even the low-affinity binder 23-deoxycastasterone significantly inhibited root growth of wild-type seedlings at a concentration of 100 nM (Fig. 3c).

We next correlated in vitro BR receptor binding affinities with BR levels in planta. We quantified BR levels in 10-day-old *Arabidopsis* plants using ultra-high-performance liquid chromatography coupled with tandem mass spectrometry (UHPLC–MS/MS) (Methods). In whole wild-type seedlings, brassinolide was detected at low levels (~0.6 pmol per gram fresh weight (FW)), while castasterone levels were ~20-fold higher (Fig. 3d). 28-Norbrassinolide (~0.9 pmol g^−1^ FW) and 28-homobrassinolide (~0.8 pmol g^−1^ FW) were also present at low concentrations, whereas 28-homocastasterone (~80 pmol g^−1^ FW) and typhasterol (~5.0 pmol g^−1^ FW) were abundant (Fig. 3d). Correlating these endogenous BR levels with their respective in vitro binding affinities (Fig. 2c,d) suggests that, in addition to brassinolide, other BRs—such as castasterone, 28-norbrassinolide and 28-homocastasterone—may act as physiologically relevant ligands for BRI1, BRL1 and BRL3.

### Constitutive activation or inactivation of BRI1 modulates BR homeostasis

We next compared BR levels in wild-type, *bri1* null^47^ and oBIR3-iBRI1 (ref. ^52^) constitutively active seedlings (Supplementary Table 2). In the *bri1* mutant, brassinolide, castasterone, typhasterol and 28-homo-brassinolide levels were elevated by 2- to 3-fold, respectively, compared to wild type (Fig. 3d), as previously described^45^. This aligns with earlier findings that BR metabolic and catabolic gene expression is altered in *bri1* loss-of-function mutants^53^, partly mediated by the BR-regulated transcription factor BZR1^40,54^. In oBIR3-iBRI1 seedlings, which show constitutive BR responses^52^, brassinolide, castasterone and 28-norbrassinolide levels were reduced compared to wild type (Fig. 3d).

To further investigate, we performed RNA sequencing (RNA-seq) on untreated 10-day-old Col-0 seedlings, a *bri1* null mutant^47^ and in *bri1* complementation lines expressing either a wild-type BRI1 receptor^47^, the BRI1^*sud1*^ gain-of-function allele^36,47^ or the constitutively active, ligand-independent oBIR3-iBRI1 receptor chimera^52^. We found many BR biosynthesis genes to be mildly induced in *bri1* mutant seedlings and to be repressed in the constitutively active oBIR3-iBRI1^52^ line (Fig. 3e). BR biosynthesis genes were up-regulated in *bri1* and down-regulated in oBIR3-iBRI1 seedlings (Fig. 3e). Conversely, several BR catabolic genes were repressed in *bri1* and induced in oBIR3-iBRI1 plants (Fig. 3e). ABCB1 and ABCB19, which function as BR transporters required for signalling^23^, also showed differential expression in *bri1* and oBIR3-iBRI1 seedlings (Fig. 3e). *BRL1*, *BRL2* and *BRL3* transcript levels were reduced in oBIR3-iBRI1 and partially induced in *bri1* (Fig. 3e). Expression of a wild-type BRI1 receptor restored gene expression in *bri1* mutants to wild-type levels (Fig. 3e). The gain-of-function allele BRI1^*sud1*^ mimicked the effects of the constitutively active oBIR3-iBRI1 receptor chimera, albeit with less pronounced effects (Fig. 3e). Together, these experiments indicate that BRI1 signalling activity shapes BR homeostasis by modulating the expression of BR metabolic, catabolic and transport genes.

### BRL2 binds BRs with medium affinity in vitro

**Fig. 4:**
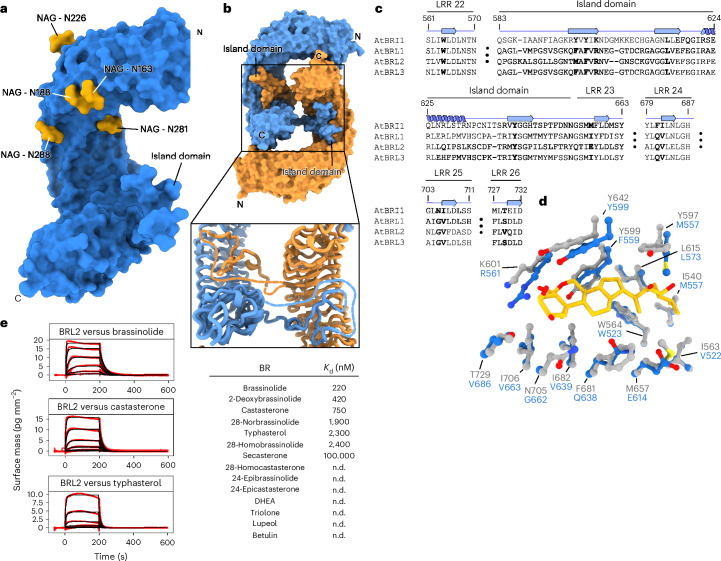
BRL2 harbours a conserved ligand-binding pocket and binds BRs with medium affinity. **a**, Crystal structure of the apo BRL2 ectodomain. Shown is a molecular surface view with the LRR and island domains in blue and *N*-glycans in yellow. **b**, BRL2 forms a domain-swapped crystallographic dimer. Shown are two BRL2 molecules in the asymmetric unit of the monoclinic crystal form (in blue and light orange, respectively), with the island domain of each molecule binding to the LRR domain of its neighbour. The island domains are partially disordered, indicated by dotted lines (inset). **c**, Structure-based multiple sequence alignment of steroid-binding pockets of BRI1, BRL1, BRL2 and BRL3. Residues involved in ligand coordination are highlighted in bold-face (residue and secondary structure numbering according to BRI1). **d**, Structural superposition of the BRI1 ectodomain (ribbon diagram, in grey; PDB ID, 3RJ0) with the AlphaFold2 model of the apo BRL2 ectodomain (in blue, AlphaFoldDB-ID, AF-Q9ZPS9-F1; r.m.s.d. of ~0.8 Å comparing 163 C_α_ atoms). Brassinolide is in bonds representation; interacting residues are shown as ball and sticks. **e**, GCI-derived binding kinetics of the BRL2 ectodomain versus brassinolide, castasterone and typhasterol. Red traces represent experimental sensorgrams (*n* = 2), and black lines show the global fits. A *K*_d_ table of the BRL2 ectodomain versus different BRs, non-BR steroids and triterpenpoids is shown alongside (n.d., no binding detected).

The structural flexibility of the BR-binding pockets in BRI1, BRL1 and BRL3, along with altered BRL2 expression in BRI1 signalling mutants (Fig. 3e), prompted us to re-examine BRL2’s ligand-binding properties. BRI1 and BRL2 share ~40% sequence identity in their extracellular domains. We expressed and purified the BRL2 ectodomain from insect cells and solved its apo crystal structure to 2.6 Å resolution in two independent crystal forms (Methods and Supplementary Table 1). The *N*-glycosylated BRL2 ectodomain adopts a superhelical architecture with 21 LRRs (compared to 25 in BRI1) with a more open conformation (Fig. 4a and Supplementary Fig. 4a). BRL2’s island domain is partially disordered and participates in a domain-swapped crystallographic dimer (Fig. 4b and Supplementary Fig. 4b).

**Extended Data Fig. 3:**
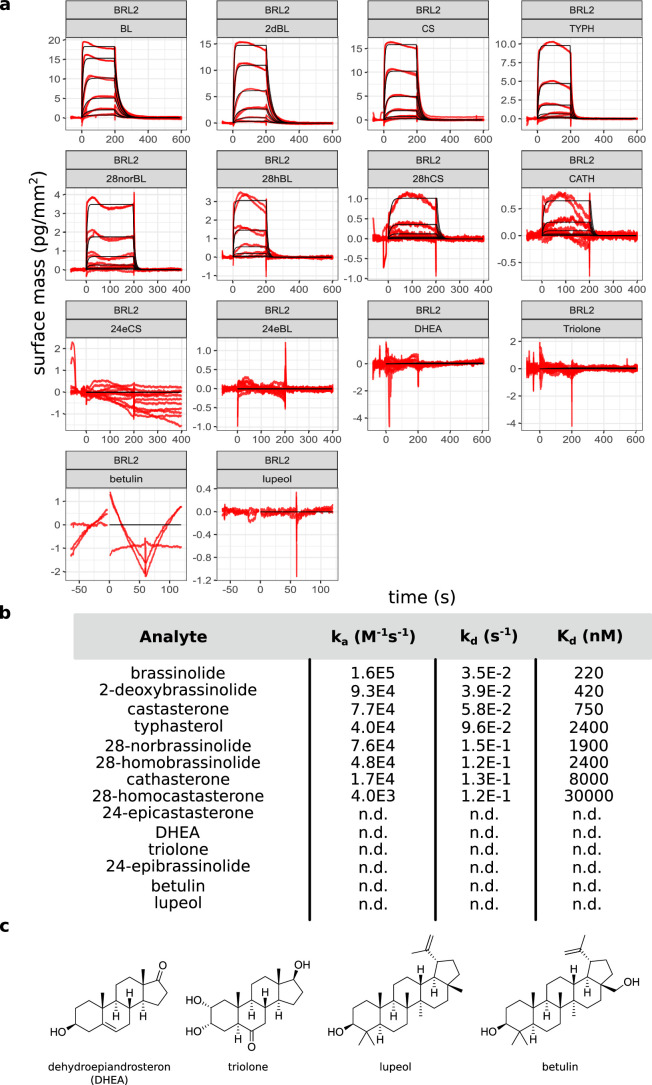
Complete set of GCI sensorgrams and derived kinetic parameters for the experiments described in Fig. 4e. **a**, Grating Coupling Interferometry (GCI)-derived binding kinetics for BRL2 in response to different BRs and other plant steroids. Raw sensorgrams are shown with experimental traces in red (n=2), and global fits as black lines (n.d., no detectable binding). BL: brassinolide, 2dBL: 2-deoxybrassinolide, CS: castasterone, TYPH: typhasterol, 28norBL: 28-norbrassinolide, 28hBL: 28-homobrassinolide, 28hCS: 28-homocastasterone, CATH: cathasterone, 24eCS: 24-epicastasterone, 24eBL: 24-epibrassinolide, DHEA: dehydroepiandrosterone. **b**, Kinetic parameters obtained for BRL2 in response to the different BR and non-BR ligand candidates analyzed in this study. **c**, Chemical structures of dehydroepiandrosterone (DHEA), triolone, lupeol and betulin.

The ligand-binding pocket of BRL2 shows a high degree of sequence and structural similarity with the BR-binding sites of BRI1, BRL1 and BRL3 (Fig. 4c,d and Supplementary Fig. 4c). Using GCI assays, we found that BRL2 can bind BRs, but it has a distinct ligand-binding profile compared to BRI1, BRL1 and BRL3 (Figs. 2c,d and 4e). BRL2 bound brassinolide and castasterone with *K*_d_ values of ~200 nM and ~400 nM, respectively (Fig. 4e, Extended Data Fig. 3 and Supplementary Table 5), 10- to 30-fold weaker than BRI1 (Fig. 2c,d). Unlike BRI1, which binds typhasterol and 28-norbrassinolide with nanomolar affinity, BRL2 bound these ligands only weakly (Fig. 4e). BRL2 showed no detectable binding to 28-homocastasterone, 24-epicastasterone, 24-epibrassinolide, dehydroepiandrosterone, triolone, lupeol or betulin (Fig. 4e). These observed differences in the ligand-binding specificity between BRI1, BRL1 and BRL3, and BRL2 correlate with amino acid changes in the BRL2 BR-binding pocket (Fig. 4c). Tyr597 and Met657 in BRI1 correspond to Met557 and Glu614 in BRL2, respectively (Fig. 4d). The corresponding BRI1^M657E^ and BRI1^Y597M/Y599F/M657E^ mutant isoforms show moderate to severe growth phenotypes and reduced BR signalling responses^46^. Overall, BRL2 contains a structurally conserved BR-binding pocket and can bind selected BRs in vitro with mid-nanomolar to micromolar affinity.

VH1/BRL2 has been characterized as a vascular receptor kinase involved in leaf vein patterning^34,55^. The previously described *brl2-1* mutant^55^ showed wild-type-like responses in BR hypocotyl growth and BES1 dephosphorylation assays (Supplementary Fig. 5a,b). In our hands, the leaf vein patterns of *brl2-1* plants were not significantly different from the Col-0 wild type (Supplementary Fig. 5c). Transcriptional *BRL2prom*::NLS-3xmVenus and translational *BRL2prom*::BRL2-mCitrine reporter lines confirmed the expression of BRL2 in vascular tissues across different developmental stages (Supplementary Fig. 5d,e and Supplementary Table 3). We generated a second *brl2-2* loss-of-function allele using CRISPR/Cas9 (clustered regularly interspaced short palindromic repeats and CRISPR-associated protein 9) gene editing (Supplementary Fig. 5f), which like *brl2-1* was indistinguishable from wild type (Supplementary Fig. 5g). We have previously reported that receptor chimeras, in which the extracellular and transmembrane domains of BIR3 (oBIR3) are fused to the cytoplasmic kinase domain of a SERK-dependent receptor kinase (iRK), trigger constitutive, ligand-independent signalling responses^38,52^. A *BRL2prom*::oBIR3-iBRL2 chimera in Col-0 wild-type background had elongated, slender petioles and curly leaves, similar to BRI1^*sud1*^ gain-of-function mutants^24,47^ (Supplementary Fig. 5g and Supplementary Table 3). However, the rosette phenotypes were milder than those of *BRI1prom*::oBIR3-iBRI1 chimeras^52^ (Supplementary Fig. 5g). These experiments suggest that BRL2 may have the capacity to initiate a SERK-dependent signalling cascade, yet its physiological ligand and physiological function remain to be characterized.

### A mutational map of the BR-binding pocket rationalizes the specificity and plasticity of BR receptors

**Fig. 5:**
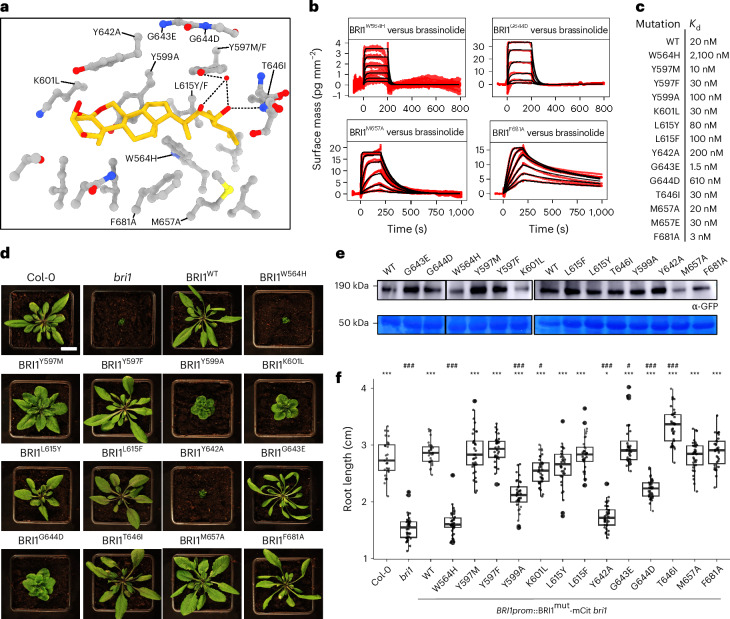
A conserved aromatic residue in the hormone-binding pocket is critical for BR perception. **a**, Coordination of brassinolide (in bonds representation, in yellow, PDB ID, 3RJ0) in the BRI1 steroid-binding pocket. Residues in contact with the ligand and used for the mutational analysis are displayed as ball and sticks; hydrogen bonds are shown as dotted lines. **b**, GCI-derived binding kinetics for BRI1^W564H^, BRI1^G644D^, BRI1^M657A^ and BRI1^F681A^ versus brassinolide. Red traces represent experimental data (*n* = 2), black lines show the global fits. **c**, *K*_d_ table for BRI1 point mutants versus brassinolide. **d**, Rosette phenotypes of representative 5-week-old transgenic T3 *BRI1prom*::BRI1-Citrine^mut^ complementation lines in *bri1* null^47^ background compared to the Col-0 wild-type control (scale bar, 2 cm). **e**, Quantification of BRI1^mut^-mCitrine (theoretical molecular mass of ~158.6 kDa) protein levels by western blot from 10-day-old seedling protein extracts, immuno-blotted with an anti-GFP antibody. ReadyBlue-stained RuBisCO is shown below as input loading control. **f**, Root length phenotypes of 8-day-old seedlings (*n* = 30) shown as box plots (centre line, median; box, IQR; whiskers, lowest/highest data point within 1.5 IQR of the lower/upper quartile; raw data shown as grey dots). Multiple comparison of the different treatments versus the indicated controls were performed using a two-sided Dunnett^89^ test as implemented in the R package multcomp^90^ (multiple comparison against *bri1*: *P < 0.05; **P < 0.01; ***P < 0.001; against Col-0 ^#^*P* < 0.05; ^##^*P* < 0.01; ^###^*P* < 0.001).

**Extended Data Fig. 4:**
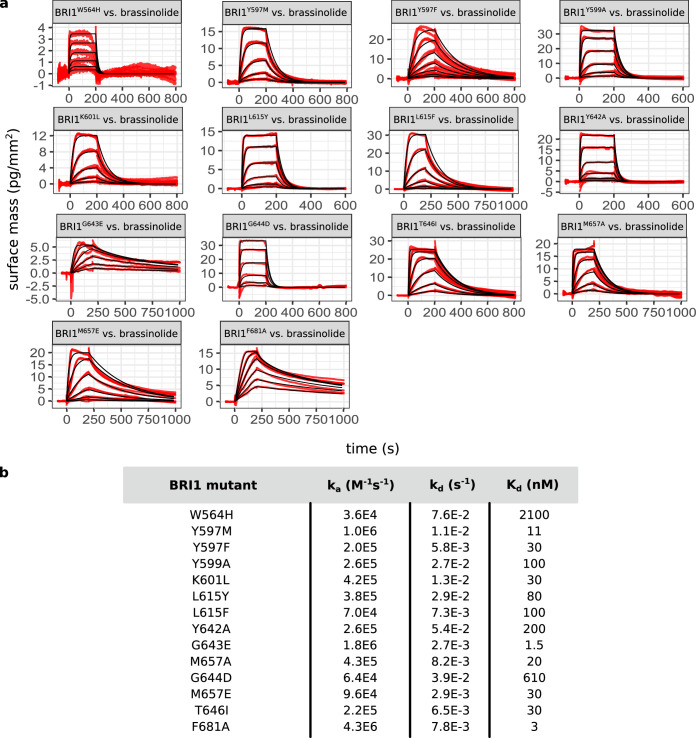
Complete set of GCI sensorgrams and derived kinetic parameters for the experiments described in Fig. 5b, c. **a**, Grating Coupling Interferometry (GCI)-derived binding kinetics for different BRI1 ectodomains carrying point mutations in the BR-binding pocket vs. brassinolide. Raw sensorgrams are shown with experimental traces in red, and global fits as black lines. **b**, Table summaries of kinetic parameters obtained for the experiments shown in **a**.

Given the high structural conservation of the BR-binding site in BRI1, BRL2, BRL1 and BRL3 (Supplementary Figs. 1 and 6a), we examined the role of individual pocket-lining residues in BR perception. On the basis of our BRI1–BR complex structures (Fig. 3a), we mutated 11 residues in the LRR core and island domain (Fig. 5a and Supplementary Table 3) and obtained brassinolide binding kinetics for each mutant ectodomain (Fig. 5b,c and Extended Data Fig. 4). All mutants behaved as well-folded monomers in solution (Supplementary Fig. 6b).

Consistent with the BR-binding site’s structural plasticity, mutation of the conserved Thr646, Tyr597 or Lys601 from the island domain had no detectable effect on brassinolide binding (Fig. 5a–c). Substitution of Tyr599 or Tyr642 with alanine moderately reduced binding (*K*_d_ ≈ 100 nM and ~200 nM, respectively), and replacing the invariant Leu615 with phenylalanine or tyrosine likewise had little effect on BR binding (Fig. 5a–c). By contrast, mutation of Trp564 to His caused an ~100-fold drop in BR binding (*K*_d_ ≈ 2 μM) (Fig. 5a–c). Mutating Met657 (which corresponds to Glu614 in BRL2; Fig. 4c,d) to either alanine or glutamate did not affect brassinolide binding (Fig. 5a–c). A moderate growth phenotype has been previously reported for the BRI1^M657E^ mutant^46^. These findings underscore the presence of a conserved BR-binding site in BRI1/BRL1/BRL3 and in BRL2 (Fig. 4d). As previously shown^46^, the *bri1-6*^45^ mutation (Gly644 to Asp) strongly reduces brassinolide binding (Fig. 5a–c). By contrast, the neighbouring Gly643 to Glu mutation in the BRI1^*sud1*^ gain-of-function allele^47^ stabilizes the island domain^36^ and enhances brassinolide binding by ~13-fold compared to wild type (Fig. 5a–c). Similarly, mutating LRR core Phe681 to alanine increases binding by ~7-fold. These results further highlight the BR-binding site’s structural plasticity and identify Trp564 from the LRR core as essential for ligand recognition.

To assess the functional impact of these mutations, we expressed mutant BRI1 receptors under the native BRI1 promoter in a *bri1* null background (Fig. 5d). T3 generation transgenic lines with wild-type-like BRI1 expression (Fig. 5e and Supplementary Fig. 6c) and proper plasma membrane localization (Supplementary Fig. 6d) were selected. High-affinity mutants (Tyr597 to Phe, Leu615 to Phe, Thr646 to Ile) restored wild-type rosette and root growth phenotypes (Fig. 5d–f) and BR responses (Supplementary Fig. 6e,f and Supplementary Table 4). Intermediate phenotypes were observed in lines with Tyr597 to Met, Leu615 to Tyr, or Met657 to Ala, consistent with their moderate in vitro binding affinities (Fig. 5a–c). The Gly644 to Asp mutant showed medium-to-strong defects, while Trp564 to His, which showed ~100-fold reduced binding, phenocopied the *bri1* null at both seedling and adult stages (Fig. 5d–f).

Conversely, Gly643 to Glu and Phe681 to Ala conferred gain-of-function rosette phenotypes, matching their enhanced binding affinities compared to wild type (Fig. 5a–d). Three mutants (Tyr599 to Ala, Lys601 to Leu, Tyr642 to Ala) showed severe growth phenotypes despite retaining strong brassinolide binding in vitro (Fig. 5a–f; see below). Overall, we observed a strong correlation between the in vitro ligand-binding affinities and the in vivo growth phenotypes of BRI1 receptor mutants that target amino acids involved in ligand coordination. Our analysis revealed novel loss-of-function (Trp564-His) and gain-of-function (Phe681-Ala) alleles, which can be used to precisely control BRI1 activity in planta.

### BR antagonists target the interaction of BR receptors with SERK co-receptors

**Fig. 6:**
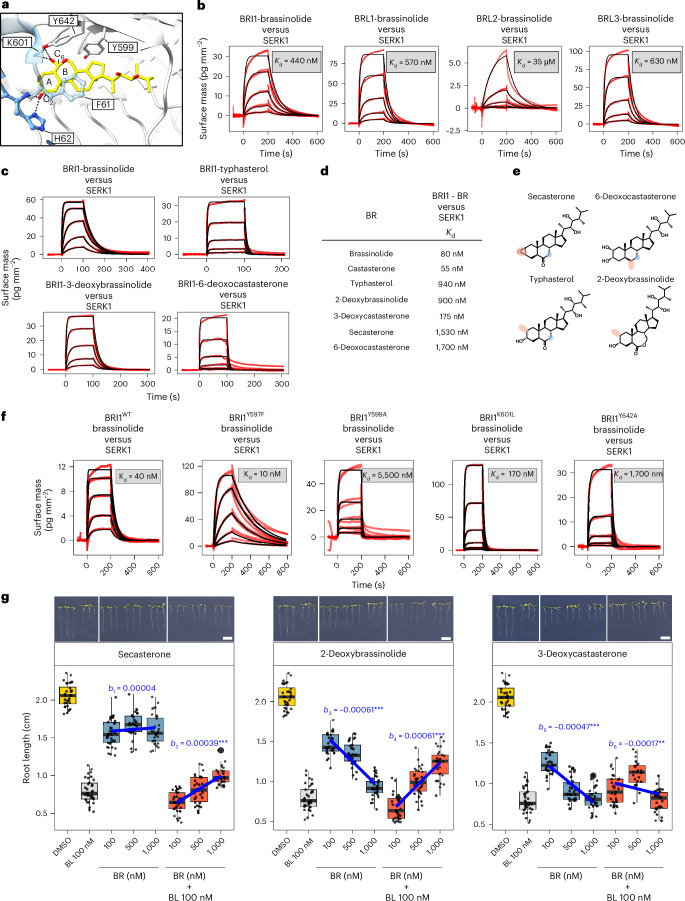
BR C_2_ hydroxylation and C_6_ oxidation are critical for SERK co-receptor binding. **a**, Close-up view of the SERK1-bound (in blue) BRI1 (in grey)–brassinolide (in bonds representation, in yellow) complex (PDB ID, 4LSX). Receptor and co-receptor amino acids involved in BR-binding are highlighted as ball and sticks in grey and blue, respectively. Polar interactions between the brassinolide C_2_ hydroxyl group and SERK1 His62, and between the C_6_ oxygen and BRI1 Tyr642 or Lys601 are shown as dotted lines (in black). **b**, GCI binding kinetics of amine-coupled and brassinolide-bound BRI1, BRL1, BRL2 and BRL3 ectodomains versus SERK1 in the presence of a constant concentration of 1 µM BL. Shown are sensorgrams with red traces indicating experimental data and black lines representing global fits (*n* = 1). Binding affinities (*K*_d_) are reported alongside. **c**, Representative GCI traces showing the interaction between SERK1 and the biotinylated, Avi-tagged BRI1 ectodomain in the presence of either brassinolide, typhasterol, 3-deoxybrassinolide or 6-deoxocastasterone. **d**, Table summaries of the receptor–BR–co-receptor interaction *K*_d_. **e**, Chemical structures of selected BRs tested in **c** and **d**. Structural modifications relative to brassinolide that reduce co-receptor binding are highlighted in red. Modifications that have little effect on complex formation are shown in blue. **f**, GCI binding kinetics of amine-coupled wild-type BRI1, BRI1^Y597F^, BRI1^Y599A^, BRI1^K601L^ and BRI1^Y642A^ ectodomains versus SERK1 in the presence of a high, saturating concentration of brassinolide. Shown are sensorgrams with red traces indicating experimental data and black lines representing global fits (*n* = 2). Binding affinities (*K*_d_) are reported alongside. **g**, BR seedling root growth inhibition antagonist assay (*n* = 30, individual seedlings per condition). Brassinolide (BL) at a fixed concentration of 100 nM was used in the different treatments, versus 0.02% (v/v) DMSO as control (in yellow). The antagonist candidate compounds secasterone, 2-deoxybrassinolide and 3-deoxycastasterone were tested for their ability to inhibit seedling root growth by themselves (in blue) or to reverse BL-induced root growth inhibition (in orange). Shown are boxplots with centre lines (medians); box limits indicate the 25th and 75th percentiles; whiskers extend to the full data range. The relationship between antagonist concentration and root length were tested using a two-sided Tukey trend test^91,92^. The blue lines represent the linear trend with slope *b* (negative values indicate agonistic behaviour; positive values indicate antagonistic behaviour) and the associated test *P* value (*P < 0.05; **P < 0.01; ***P < 0.001). The exact *P* values are 0.4943212 (*b*_1_), 0.0000000 (*b*_2_), 0.0000000 (*b*_3_), 0.0000000 (*b*_4_), 0.0000000 (*b*_5_) and 0.0032034 (*b*_6_). Representative seedling images are shown above (scale bars, 0.5 cm). The DMSO and BL 100 nM controls are the same in each panel, indicated by a white vertical line. The experiment was repeated three times with similar results.

Previously, we demonstrated that the BR-binding site encompasses the LRR core and island domain of BRI1, and the LRR ectodomain of a SERK co-receptor^26,28,36^ (Fig. 6a). SERK1 Phe61 stacks with the A/B rings of brassinolide, while His62 forms a conserved hydrogen bond with the C_2_ hydroxyl group of the ligand^36^ (Fig. 6a). Mutating the corresponding Phe60 and His61 in SERK3/BAK1 to alanine abolishes both co-receptor interaction in vitro and BR signalling in planta^28^.

**Extended Data Fig. 5:**
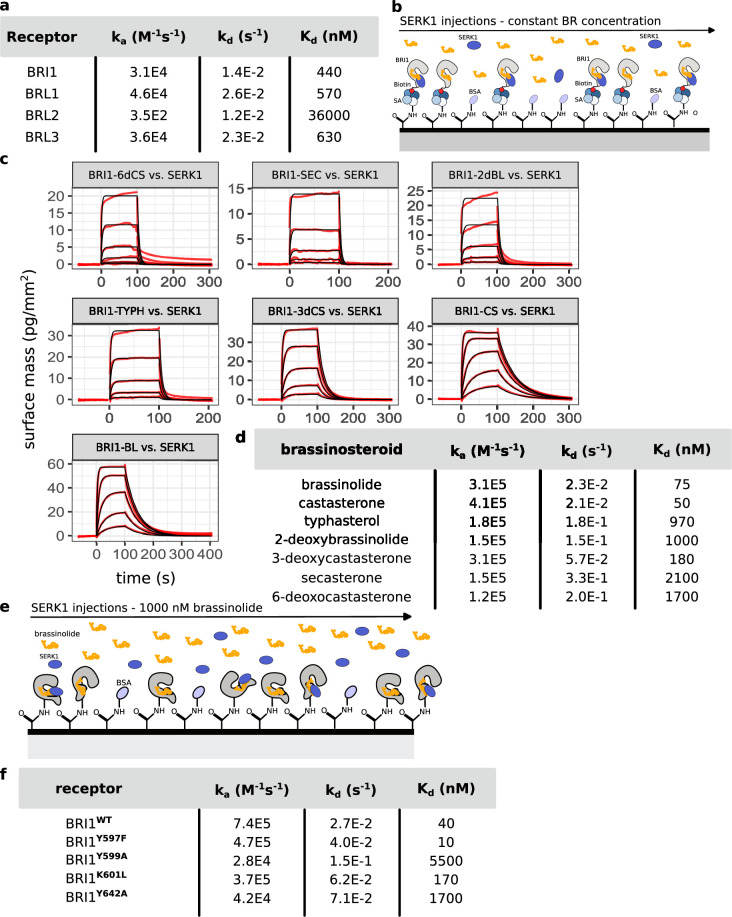
Complete set of GCI sensorgrams and derived kinetic parameters for experiments shown in Fig. 6b–f. **a**, Table summaries of kinetic parameters obtained for the experiments shown in Fig. 6b. **b**, Schematic overview of the GCI receptor capturing and SERK1 binding assay. For Avi-tag based coupling, streptavidin (SA, shown as a tetramer) was immobilized using the amine-coupling method on the GCI chip (black line). Next, the biotinylated avi-tagged BR receptor ectodomain was captured by streptavidin, followed by passivation and quenching of the surface with BSA (in light blue). Then, in the presence of a constant concentration of brassinolide in the analyte buffer (in yellow), SERK1 (in dark blue) association and dissociation at different protein concentrations was performed. **c**, Grating Coupling Interferometry (GCI)-derived binding kinetics for the avi-tagged BRI1 ectodomain vs. different antagonist candidate ligands including 6dCS: 6-deoxocastasterone, SEC: secasterone, TYPH: typhasterol, 3dCS: 3-deoxocastasterone, CS: castasterone, BL: brassinolide. Raw sensorgrams are shown with experimental traces in red, and global fits as black lines. **d**, Table summaries of kinetic parameters obtained for the experiments shown in Fig. 6c, d. **e**, Schematic overview of the GCI receptor capturing and SERK1 binding assay to analyze the effect of BR binding site missense mutations in BRI1 for SERK1 co-receptor binding (related to Fig. 6f). BRI1^WT^, BRI1^Y597F^, BRI1^Y599A^, BRI1^K601L^ and BRI1^Y642A^ were immobilized by amine-coupling on the GCI chip (black line). SERK1 binding to the receptors (in gray) was then quantified in the presence of a constant, high concentration of brassinolide (in yellow) in the running buffer and by injecting the co-receptor at different concentrations (in blue). **f**, Table summaries of kinetic parameters obtained for the experiments shown in Fig. 6f.

To test whether this activation mechanism is conserved across BR receptors, we immobilized BRI1, BRL1, BRL2 and BRL3 ectodomains on a GCI chip and examined SERK1 binding in the presence of 100 nM brassinolide. BRL1 and BRL3, like BRI1, bound SERK1 with high affinity, while BRL2 showed much weaker SERK1 binding (*K*_d_ ≈ 35 μM) (Fig. 6b and Extended Data Fig. 5).

We next quantified the interaction of SERK1 with BRI1 in the presence of different BRs. We used an avi-tagged BRI1 ectodomain to minimize steric hindrance between the GCI chip and the SERK1 co-receptor (Methods) and measured *K*_d_ of ~100 nM for SERK1 association to either brassinolide- or castasterone-bound BRI1 (Fig. 6c,d and Extended Data Fig. 5). However, SERK1 binding was ~15-fold weaker with typhasterol, when compared to castasterone-bound BRI1 (Fig. 6c,d), despite these ligands having similar receptor binding affinities (Fig. 2c,d). This reveals that the C_2_ hydroxyl coordinated by SERK1 His62 is key for receptor–co-receptor complex formation (Fig. 6a,e). Similarly, 2-deoxybrassinolide reduced SERK1 binding ~10-fold, while 3-deoxycastasterone, lacking the C_3_ hydroxyl, still supported high-affinity SERK1 binding (Fig. 6c–e). Secasterone—with a bulky 2,3-α-epoxy group—impaired complex formation, as did 6-deoxocastasterone, despite possessing both C_2_ and C_3_ hydroxyls (Fig. 6c–e), likely because of its distorted binding mode in the BR pocket (Fig. 3a,b).

Our data identify the BR C_2_ hydroxyl as essential for SERK recruitment. While C_6_ oxidation on the B-ring is not required for receptor binding, its coordination by BRI1 island domain residues Tyr599, Tyr642 and Lys601 may help orient the ligand for optimal co-receptor binding (Fig. 6a,c–e). Indeed, SERK1 binding is partially or severely compromised in the BRI1^Y599A^, BRI1^K601L^ and BRI1^Y642A^ mutants in the presence of high, saturating concentrations of brassinolide (Fig. 6f). These results rationalize the dwarf phenotypes of the BRI1 Tyr599 to Ala, Lys601 to Leu and Tyr642 to Ala mutants (Figs. 5d–f and 6a), despite their ability to bind brassinolide with high affinity (Fig. 5c).

We hypothesize that BRs that bind to BRI1 with high affinity but prevent SERK association may act as receptor antagonists. We performed seedling root growth inhibition assays in the presence of 100 nM brassinolide and with different antagonist candidates from our biochemical screen (Fig. 6d) and found that secasterone applied on its own did not trigger root growth inhibition in a concentration-dependent manner (Fig. 6g). However, applying increasing concentrations of secasterone significantly reversed brassinolide-induced root growth inhibition (Fig. 6g). 2-Deoxybrassinolide acted as a weak BR agonist when applied on its own but had a significant, dose-dependent antagonistic effect on brassinolide-induced root growth inhibition (Fig. 6g). By contrast, 3-deoxycastasterone acted as a weak BR agonist in both the absence and presence of brassinolide in the assay (Fig. 6g). We conclude from these experiments that BRs lacking a C_2_ but not C_3_ hydroxyl group behave as receptor antagonists (Fig. 6a,c–e,g). A bulky head group, such as in secasterone, is also effective in blocking receptor activation in vitro and in planta (Fig. 6a,c–e,g). Taken together, BRs that inhibit BRI1–SERK1 complex formation (Fig. 6c–e) in vitro without compromising isolated receptor binding (Fig. 2c,d) can partially reverse brassinolide-induced root growth inhibition in a dose-dependent manner (Fig. 6g), underscoring the importance of A-ring features in BR receptor activation^28,36^.

## Discussion

**Fig. 7:**
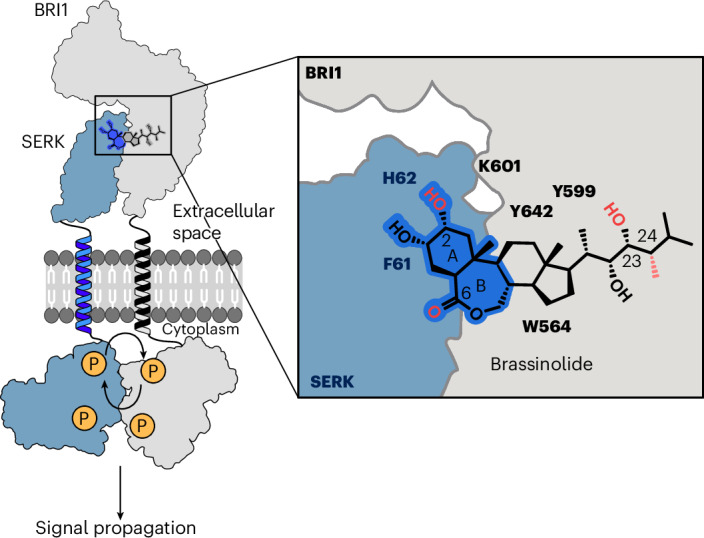
Strong receptor binding, correct positioning within the BR binding site and interaction with the co-receptor are essential for BR bioactivity. Schematic overview of BR-induced receptor–co-receptor complex formation and activation. A BR ligand binds to the BRI1 receptor ectodomain (in grey), creating a binding surface for a SERK co-receptor kinase (in blue). This brings the kinase domains of receptor and co-receptor in close proximity, leading to trans-phosphorylation and intracellular signalling. The BR-binding pocket is formed by the receptor (in grey) and co-receptor (in blue). Chemical features of the BR ligand (in black) involved in co-receptor binding are highlighted in dark blue; key carbon atoms critical for receptor or co-receptor interaction are numbered, and chemical features required for receptor and/or co-receptor binding are shown in red. The approximate positions of residues critically involved in BR binding, positioning and co-receptor binding are indicated.

Genetic and metabolite studies have revealed a complex BR biosynthetic network in plants^2,14^. In this study, we provide quantitative binding kinetics for 15 distinct BRs from this network, evaluated against the four BR receptors in *Arabidopsis*. Like other plant hormones^56,57^, BRs act as molecular glues promoting the association of receptor and co-receptor. Our work now defines the chemical features required for high-affinity receptor binding and co-receptor recruitment. The most unexpected finding is that hydroxylation at C_23_ and the C_24_ stereochemistry in the BR aliphatic tail are critical determinants for high-affinity receptor binding (Fig. 2b–d) and BR bioactivity (Figs. 3c and 7). Consistent with this, C_23_ hydroxylase mutants show severe dwarfism^9,51^. A second key finding from our analysis is that improper ligand positioning within the steroid-binding pocket can be accommodated by the isolated receptor, yet becomes detrimental for co-receptor recruitment. For instance, the absence of a C_6_ ketone is tolerated by BRI1 and BRL3 (Fig. 2b–d) but significantly compromises high-affinity binding to SERK1 (Figs. 6c–f and 7). By contrast, the absence of a B-ring lactone still allows for high-affinity receptor and co-receptor association (Figs. 2b–d and 6c,d), in agreement with the moderate growth phenotype of the *br6ox2* mutant^13,51^. C_2_ hydroxylation is only essential for SERK recognition^28,36^ (Fig. 6c–e) but not for receptor binding (Figs. 2b–d and 3a,b), providing a mechanistic basis for known BR antagonists^58,59^ (Fig. 7). In planta, 2-deoxybrassinolide and secasterone act as receptor antagonists (Fig. 6e,g), supporting the idea that these abundant^48^ biosynthetic intermediates may modulate BR signalling by competing with agonists for receptor binding (Figs. 1b and 6g). This could prevent formation of active BR signalling complexes, in a similar way to the known BIR receptor pseudo-kinases^37,38^.

Mutational analysis of the BR-binding pocket in BRI1 reveals that the receptor can tolerate various missense mutations (Figs. 5 and 7), in agreement with previous mutational studies^46,60^. We identified a single point mutation, Trp564His, that results in a strong BR loss-of-function phenotype. This finding shows that BRI1 can be effectively inactivated by a single amino acid substitution, rather than requiring the combination of multiple mutations as previously reported^46^. Two additional residues (Tyr599 and Tyr642) are critically involved in the proper positioning of the ligand in the BR-binding pocket, and we report a novel gain-of-function allele (Phe681Ala) at the entry of the steroid-binding pocket (Figs. 5d–f and 7). The structural plasticity of the hormone-binding pocket supports the receptor’s capacity to recognize chemically diverse BRs, while providing sufficient selectivity to discriminate between BRs, other phytosterols and triterpenoids. The BR-binding site is conserved among the four *Arabidopsis* receptors and their orthologues in crop species, implying that BR receptors in crops likely share a similar BR ligand-binding spectrum (Supplementary Fig. 5a).

On the basis of binding profiles (Figs. 2c,d and 6c,d), bioactivity data and in vivo BR levels^11,48,50^ (Fig. 3d), we propose that brassinolide and castasterone (C_28_), 28-norbrassinolide (C_27_) and possibly 28-homocastasterone (C_29_) may act as physiological BR ligands in *Arabidopsis*, but it is presently unknown whether and how these metabolites reach the cell wall compartment to interact with the ligand-binding domains of BR receptors. Feedback regulation of BR biosynthesis likely fine-tunes these BR levels (Fig. 3e). Given that BR biosynthetic enzymes have specific expression domains^51,61^, different BR ligands may be perceived by the broadly expressed BRI1 receptor in specific tissues and organs. Such a broad ligand spectrum has been previously reported for other small-molecule^62^ and peptide^63^ hormone receptors in plants. Our quantitative biochemical assays also suggest that BRL2 can bind BRs with medium affinity (Fig. 4e) and can weakly interact with SERK co-receptors (Fig. 6b). We note that our in vitro binding assays with the isolated LRR domains probably underestimate the actual BR binding affinities of intact BRI1/BRL–SERK receptor complexes embedded in the plasma membrane, as plants can respond to BRs applied externally at picomolar to low nanomolar concentrations^44,51^.

Taken together, our work defines the chemical determinants for a small-molecule-based molecular glue mechanism allowing formation of an active receptor–steroid ligand–co-receptor signalling complex. Distinct chemical features in BRs required for (1) high-affinity receptor binding, (2) proper ligand orientation in the receptor hormone-binding pocket and (3) high-affinity co-receptor binding (Fig. 7) have now been defined and can be exploited for rational BR agonist and antagonist design.

## Methods

### Protein expression and purification

The ectodomains from BRI1 (https://www.uniprot.org UniProt-ID O22476, residues 1–788), BRL1 (Q9ZWC8, 1–770), BRL2 (Q9ZPS9, 1–752), BRL3 (Q9LJF3, 1–770) and SERK1 (Q94AG2, 23–270) ectodomains were amplified from *A. thaliana* complementary DNA (ecotype Col-0). BRI1, BRL1, BRL2 and BRL3 were cloned in vector pBB2 providing tobacco etch virus (TEV) protease cleavable C-terminal Twin-StrepII and 9xHis affinity tags. SERK1 was cloned in the same pBB2 vector containing an N-terminal azurocidin signal peptide^28,64^. For GCI assays, AtBRI1 was also cloned in vector pBB2 containing a C-terminal Avitag followed by TEV cleavable C-terminal Twin-StrepII and 10xHis affinity tags.

All proteins were expressed in *Trichoplusia ni* (Tnao38 cells, Ximbio) using the baculovirus system^65^. Tnao cells in SF900 medium (Gibco) were grown to a density of 2.0 × 10^6^ cells per ml and subsequently infected with 10 ml of P2 virus. Cells were then incubated for 24 h at 28 °C and for an additional 48 h at 21 °C while shaking at 110 r.p.m. Cells were then pelleted at 4,000*g* for 20 min, and the supernatant containing the secreted protein was filtrated using 0.45 μm filters (Durapore Merck). The cleared medium was then loaded onto an Ni^2+^ metal affinity column (HisTrap Excel 5 ml, Cytiva) equilibrated in buffer A (50 mM KPi pH 7.8, 250 mM NaCl, 1 mM β-mercaptoethanol). The column was washed with 25 ml of buffer A and subsequently eluted onto a Strep-Tactin XT 4flow 5 ml column (IBA) in buffer A supplemented with 250 mM imidazole (pH 8.0). The Strep-Tactin affinity column was further washed with 25 ml buffer (20 mM Tris pH 8.0, 250 mM NaCl and 1 mM EDTA), and the protein was eluted from the Strep-Tactin column with buffer C (10 mM Tris pH 8.0, 150 mM NaCl, 1 mM EDTA, 50 mM biotin, 1xBTX, IBA). For protein crystallization, the affinity purified proteins were incubated overnight at 4 °C with TEV protease using a molar receptor/protease ratio of ~10:1. The tag-free protein was separated from the tag and from TEV protease by a second Ni^2+^ affinity chromatography step. Finally, the untagged protein was applied to a HiLoad Superdex 200 16/200 pg column (Cytiva) equilibrated in 20 mM sodium citrate pH 5.0, 150 mM NaCl. Fractions containing the purified protein were concentrated and stored at −80 °C.

Receptor biotinylation for GCI was achieved by incubating Avi-tagged BRI1 with His-tagged BirA^66^. Briefly, BRI1 at a final concentration of 20 µM was incubated for 1 h at 30 °C with BirA, biotin, ATP and MgCl_2_ at final concentrations of 85 μM, 150 μM, 2 mM and 5 mM, respectively. The BirA enzyme was subsequently removed using a Ni^2+^ affinity chromatography step. Biotinylated BRI1 was concentrated and loaded onto a HiLoad Superdex 200 16/200 pg column (Cytiva) equilibrated in 20 mM sodium citrate pH 5.0, 150 mM NaCl.

### Chemical synthesis and analysis of BRs

The chemical synthesis of typhasterol, 3-deoxycastasterone and 2-deoxybrassinolide are described in Supplementary Note 1.

### GCI

GCI experiments were performed using the Creoptix WAVE system (Malvern Panalytical). Binding of BRs to the isolated BRI1, BRL1, BRL2 and BRL3 ectodomains was measured using 4PCH WAVEchips. Borate buffer (100 mM sodium borate, pH 9.0, 1 M NaCl, Xantec) injections were used to condition the chips. For the interaction screening of BRI1, BRL1, BRL2, BRL3 or BRI1^mut^ with BRs, the purified ectodomains were immobilized using standard amine coupling. Chips were activated using a 1:1 mix of 400 mM *N*-(3-dimethylaminopropyl)-*N*′-ethylcarbodiimide hydrochloride and 100 mM *N*-hydroxysuccinimide (Xantec). Protein ligands (in 10 mM sodium acetate, pH 5.0) were injected to the desired chip surface at the concentration indicated in Supplementary Table 5. To minimize non-specific binding of analytes, a second injection of BSA (0.5% (w/v) in 10 mM sodium acetate, pH 5.0) was used to passivate the surface. Finally, the chip was then quenched by injecting 1 M ethanolamine. All kinetic analyses were performed at room temperature in citrate buffer (20 mM sodium citrate pH 5.0, 150 mM NaCl) supplemented with 0.05% (v/v) Tween and 0.025% (v/v) dimethyl sulfoxide (DMSO) to reduce unspecific binding of the respective BR analyte. Analyte concentrations were varied in 1:3 dilution series. All experiments were done in duplicates, blank injections were used for double referencing, and DMSO calibration was performed for bulk correction.

Binding of the co-receptor SERK1 to brassinolide-bound wild-type and mutant receptors was done by amine-coupling the BRI1, BRL1, BRL2 and BRL3 ectodomains onto 4PCP WAVEchips and using the purified SERK1 ectodomain as analyte in 20 mM sodium citrate pH 5.0, 150 mM NaCl) supplemented with 0.05% (v/v) Tween and 0.025% (v/v) DMSO and brassinolide to a final concentration of 1 μM. Binding of SERK1 to BRI1 in the presence of different BRs was performed by immobilizing Avi-tagged and biotinylated BRI1 by neutravidin capturing. After chip activation, neutravidin (100 μg ml^−1^ in 10 mM sodium acetate, pH 5.0) was injected. To passivate and quench the chip surface, a further injection of 0.5% (w/v) BSA followed by an injection of 1 M ethanolamine was performed, respectively. Biotinylated BRI1 (100 μg ml^−1^ in citrate buffer) was then injected to the desired surface concentration (Supplementary Table 5), followed by an injection of biotinylated 0.5% (w/v) BSA to passivate the surface. All the binding assays between BRI1 and SERK1 were done at room temperature in citrate buffer supplemented with 0.05% Tween and the respective BR at a final concentration of 1 μM. SERK1 injections were done in 1:3 dilution series. Immobilized receptor densities and analyte concentrations for all experiments are reported in Supplementary Table 5.

Data analysis was performed with the Creoptix WAVEcontrol software (version 4.7.2), and data were fitted using one-to-one binding models or mass transport model, using bulk correction. Plotting and data representation were performed using R (https://www.r-project.org, version 4.1.2).

### Crystallization and data collection

BRL3 (in 20 mM citric acid pH 5.0, 250 mM NaCl) at a concentration of ~1 mg ml^−1^ was incubated with the respective BR (10 mM stock solution in 100% (v/v) DMSO) to a final concentration of 50 μM for 4 h at room temperature and then concentrated to ~6–7 mg ml^−1^ using a Amicon Ultra concentrator (Merck Millipore) with a molecular weight cut-off of 30 kDa. Triclinic crystals developed in hanging drops composed of 1.0 μl of protein solution and 1.0 μl of crystallization buffer (23–25% (w/v) PEG 3,350, 0.2 M Li_2_SO_4_, 0.1 M citric acid pH 4.0) suspended over 1.0 ml of the latter as reservoir solution. Crystals were cryoprotected by serial transfer into crystallization buffer containing ethylene glycol to a final concentration of 25% (v/v) and snap frozen in liquid N_2_. BRI1 (in 20 mM citric acid pH 5.0, 250 mM NaCl)–BR (at a final concentration of 50 μM) complex crystals developed in the same crystallization condition. Crystals of apo BRL2 were obtained using micro-seeding protocols in hanging drops containing 1.0 μl of protein solution (BRL2 at 6 mg ml^−1^ in 20 mM citric acid pH 5.0, 250 mM NaCl) and 1.0 μl of crystallization buffer (23% (w/v) PEG 3,350, 0.2 M MgCl2·6H_2_O, 0.1 M Tris pH 9.0) over 1 ml of reservoir solution. Tetragonal crystals were cryoprotected by serial transfer in crystallization buffer supplemented with 20% (v/v) glycerol. A second, monoclinic crystal form for BRL2 developed in 22% (w/v) PEG 3,350, 0.2 M Na citrate, 0.1 M Bis–Tris pH 7.0 and in the presence of 50 µM brassinolide. Datasets were collected at beam line X06DA of the Swiss Light Source, Villigen, Switzerland; the monoclinic BRL2 dataset was collected at beam line ID30B of the European Synchrotron Radiation Facility, Grenoble, France (Supplementary Table 1). Data processing and scaling was done with XDS^67^ (version June 2024).

### Crystallographic structure solution and refinement

The structures of the BRL3–brassinolide complex and of apo BRL2 were solved by molecular replacement in PHASER (version 2.8.3)^68^, with BRI1 ectodomain structure^26^ (PDB ID, 3RJ0) as search model. For BRL3, the solution revealed a dimer in the asymmetric unit, closely resembling the previously reported BRL1 dimer^31^. The BRL2 structure was resolved at 2.6 Å resolution in space group *P* 4_1_ 2_1_ 2, containing a single molecule per asymmetric unit, and in space group *C* 2 with two molecules per asymmetric unit, respectively (Supplementary Table 1). Crystals of the BRI1–BR complex shared the same crystal form as the previously described apo BRI1 (ref. ^26^). Restraints for the BR ligand were generated from SMILES strings using phenix.elbow (version 1.21)^69^ or JLigand (version 3.21)^70^. Ligands were fit manually into mF_o_–DF_c_ omit maps, and ligand occupancies were set to 1. All structures were completed through iterative cycles of manual model building in COOT (version 0.9.8)^71^ and restrained TLS refinement in phenix.refine (version 1.21)^72^. Analysis of the refined structures with phenix.molprobity (version 4.5.2)^73^ revealed good to excellent stereochemistry. Final omit maps of the different BR ligands were generated with phenix.polder (version 1.21)^74^. Data collection and refinement statistics are provided in Supplementary Table 1. Structural figures were prepared with ChimeraX (version 1.10)^75^, and chemical structures were drawn with PubChem sketcher (version 2.4)^76^.

### Plant material

*A. thaliana* ecotype Col-0, transfer DNA insertion mutants *bsk3-1* (SALK_096500C)^77^, *bri1* null (GABI_134E10)^47^ and *brl2-1* (SALK_016024)^55^ were obtained from the Nottingham *Arabidopsis* Stock Center, alongside the previously reported *BRI1prom*::BRI1-mCitrine / *bri1* and *BRI1prom*::BRI1^*sud1*^-mCitrine / *bri1*^36^ and *BRI1prom*::oBIR3-iBRI1-mCitrine / *bri1*^52^ transgenic lines. Genotyping primers for the mutants are listed in Supplementary Table 2.

### Molecular cloning and generation of transgenic lines

To generate BRL2 expression reporter lines, a 3.6 kb fragment upstream of the BRL2 ORF was cloned into the entry vector pUC57- L4_EcoRV-XbaI-BamHI_R1 via Gibson assembly to produce L4-*BRL2prom*-R1 (ref. ^78^). Multi-site Gateway cloning (Thermo Fisher Scientific) with Gateway LR reaction (11791020; Thermo Fisher Scientific) was used to assemble the fragments L4-BRL2prom-R1, L1-NLS-3xmVenus-L2 (ref. ^79^) and R2-tHSP18.2-L3 (ref. ^80^) into the destination vector pB7m34GW (ref. ^81^) to generate *BRL2prom*::NLS-3xmVenus. The coding sequence of BRL2 was introduced into *pEN-L1(MCS)-L2* (ref. ^78^) by Gibson assembly and subsequently combined with *L4-BRL2prom-R1* and *mCitrine-pDONR P2R P3* in destination vector *pB7m34GW* (ref. ^81^) to produce the translational reporter construct *BRL2prom::BRL2-mCitrine*. The BRL2 chimera (*BRL2prom*::oBIR3-iBRL2-mCitrine) were generated using a construct in which the N-terminal part of AtBIR3 (residues 1–246, containing the native signal peptide, extracellular domain and transmembrane helix) and the C-terminal region of BRL2 (residues 779–1,143, comprising the juxtamembrane and kinase domains) were joined using two-fragment Gibson assembly, as previously described^52^. For the BRI1 complementary assays, the BRI1 coding sequence lacking a stop codon was cloned into entry vector pDONR221 (ref. ^36^) using the Gateway BP reaction (11789020; Thermo Fisher Scientific). The single point mutations were introduced by the site-directed mutagenesis by overlap extension^82^ on the entry vector. A 1,690 bp upstream fragment of BRI1 (*BRI1prom*) and a mCitrine coding sequence with the stop codon was cloned into pDONR P4-P1R and pDONR P2R-P3, respectively, as previously reported^52^. The final binary vectors *BRI1prom*::BRI1mu-mCitrine were generated by the multi-site Gateway technology (Thermo Fisher Scientific) with destination vector pB7m34GW (ref. ^81^). Cloning primers are list in Supplementary Table 3.

The *brl2-2* allele was generated using a CRISPR/Cas9 (ref. ^83^) system^84^. Two 20 bp single-guide RNA target sequences, designed using CRISPR-P 2.0 (ref. ^85^), were cloned into shuttle vectors pEF004/005 via mutagenesis PCR^86^. These cassettes were subsequently assembled into the pBay-Bar-CRmCherry vector using Gibson assembly, and the final construct was transformed into Col-0 wild-type plants using the floral dip method^87^. Primary transformants (T1) were selected on half-strength Murashige and Skoog (^1/2^MS) medium containing 15 mg l^−1^ phosphinothricin; Cas9-free mutant lines (T2/T3) were identified by screening for loss of the mCherry marker (Leica Stereo Microscope MSV269).

All constructs were transferred into *Agrobacterium tumefaciens* strain GV3101 by electroporation, and transgenic lines were generated by floral dip^87^. Transformants were selected on ^1/2^MS media containing 1% (w/v) sucrose, 0.8% (w/v) agar and 15 mg l^−1^ phosphinothricin.

### BR root growth inhibition and competition assays

For BR-mediated root growth inhibition assays^43,44^, surface-sterilized seeds were stratified at 4 °C for 2 days and plated on ^1/2^MS media plates containing 1% (w/v) sucrose and 0.8% (w/v) agar, supplemented with 100 nM of the indicated BR from 10 mM stock solutions prepared in 100% (v/v) DMSO (Carl ROTH) or with 0.01% (v/v) DMSO as control. The previously reported *bsk3-1* T-DNA insertion mutant (SALK_096500C)^77^ was used as negative control. For the BR antagonist competition assays, 100 nM brassinolide and 100, 500 or 1,000 nM of antagonist candidate were added to ^1/2^MS plates containing 1% (w/v) sucrose and 0.8% (w/v) agar. DMSO to a final concentration of 0.02% (v/v) was used as control treatment. In all cases, plates were moved to a Percival (SE-41AR3; CLF PlantClimatics, settings: 70% humidity, 16 h light period at 22 °C with 250 μmol m^−2^ s^−1^ light intensity and 8 h dark period at 18 °C), vertically placed for 7 days. The plates were scanned using a flat-bed scanner (Epson perfection V600 Photo, Epson). The primary root length of 30–35 individual seedlings was then determined using Fiji (version 1.54)^88^.

### Statistical analysis

Multiple comparisons of different treatments versus control(s) in one-way layouts were performed using a two-sided Dunnett^89^ test as implemented in the R package multcomp (version 1.4-28)^90^. The relationship between root length and *K*_d_ were tested using a Tukey trend test^91,92^, that is, a multiple regression model approach using arithmetic, ordinal or logarithmic scores. We chose the linear regression model and represented the corresponding slope *b* (positive slopes were interpreted as antagonistic; negative slopes as agonistic) and associated *P* values. Data were plotted in R^93^ (version 4.2.2); the associated raw data can be found in Supplementary Tables 6–8.

### BR in vivo quantification

The extraction and quantification of endogenous BRs were carried out as previously described^94^. In brief, five biological replicates, each consisting of approximately 10 mg FW of plant material, were extracted using ice-cold 60% (v/v) acetonitrile. To each sample, 25 pmol of deuterium-labelled BR internal standards (Olchemim) were added. After a 12 h incubation at 4 °C, samples were centrifuged, and the resulting supernatants were purified using 50 mg Discovery DPA-6S solid-phase extraction cartridges (Supelco). The eluates were then evaporated to dryness and reconstituted in 40 µl of methanol. BR quantification was performed using UHPLC–MS/MS, using the ACQUITY UPLC I-Class System (Waters) and the Xevo TQ-S triple quadrupole mass spectrometer (Waters MS Technologies), using the previously reported UHPLC–MS/MS parameters^94,95^.

### RNA-seq experiments

Ten-day-old *Arabidopsis* plants grown on ^1/2^MS media plates containing 1% (w/v) sucrose and 0.8% (w/v) agar were collected. Fifteen plants were pooled per individual biological replicate, and all samples were ground. RNA was extracted using a RNeasy Plant Mini Kit (74904; Qiagen), and total RNA concentration was estimated using a Qubit 4 Fluorometer (Q33238; Thermo Fisher Scientific) and a 2100 Bioanalyzer system (Agilent Technologies). Library preparation and sequencing (100 bp single-end with Novaseq 6000 sequencing system; Illumina) was performed at the iGE3 Genomic Platform at the Faculty of Medicine, University of Geneva, Switzerland (https://ige3.genomics.unige.ch/). Data analysis was conducted within the Galaxy EU platform (https://usegalaxy.eu/) using the following tools: quality control and adaptor trimming by MultiQC (version 1.34)^96^, reads mapping to the reference genome (*Arabidopsis* TAIR10 reference genome from TAIR; https://www.arabidopsis.org/) by HISAT2 (version 2.1.0)^97^, transcript assembly and fragments per kilobase of transcript per million mapped reads estimates by Cufflinks (version 2.2.1.2)^98^, reads counting by FeatureCounts (version 2.0.8)^99^ and differential gene expression analysis by DESeq2 (version 2.11.40.7)^100^. Visualization was done using SRplot^101^ (https://www.bioinformatics.com.cn/), the corresponding raw data are summarized in Supplementary Table 9.

### Confocal microscopy

To detect BRL2 promoter activity with *BRL2prom*::NLS-3xmVenus (in T2 generation) in the early vasculature of embryos, SCRI Renaissance 2200 staining was performed according to the protocol^84,102^. The embryos were dissected from the siliques in the flowering 5-week-old plants under a Leica S6D stereo microscope (Leica S6D) and incubated in an SR2200 solution containing 0.1% (v/v) SR2200, 1% (v/v) DMSO, 0.5% (v/v) Triton X-100, 5% (v/v) glycerol and 4% (v/v) paraformaldehyde in phosphate-buffered saline buffer (pH 8.0) for 5 min. Then, the embryos were washed once with H_2_O and mounted in 5% (v/v) glycerol for imaging. The cell wall signal stained by SR2200 was obtained using a 405 nm laser and a detection wavelength ranging from 415 to 475 nm. To image roots, propidium iodide (PI) staining was used to label the cell wall. The primary root of 10-day-old seedlings previously grown on ^1/2^MS plates containing 1% (w/v) sucrose and 0.8% (w/v) agar was incubated in a 10 mg l^−1^ PI solution for 10 min. Then, it was washed three times with water and incubated in a 5% (v/v) glycerol solution as the imaging buffer. PI fluorescence was detected from 571 nm to 656 nm following a 561 nm wavelength laser excitation. The mVenus signal was detected using a laser with a 514 nm wavelength laser for excitation and detectors with an emission range between 517 and 570 nm. To detect the mCitrine signal in the roots of T2 generation *BRI1prom*::BRI1^mut^-mCitrine complementation lines, primary root tips from 10-day-old seedlings growing on ^1/2^MS plates containing 1% (w/v) sucrose and 0.8% (w/v) agar were sampled. The mCitrine signal was detected by a laser with 514 nm wavelength for excitation; detectors recorded emission between 517 nm and 570 nm. All imaging was performed with a LSM 780 NLO microscope (Zeiss) at the Photonic Bioimaging Center, University of Geneva, Switzerland, and data were analysed using ZEN 2.0 blue edition and Fiji^88^.

### Analysis of leaf vein patterning

Leaf vein patterns were analysed as described^55^. Cotyledons from 2-week-old seedlings grown on ^1/2^MS plates containing 1% (w/v) sucrose and 0.8% (w/v) agar were dissected and cleared in 95% ethanol, hydrated in an ethanol dilution series to water and mounted on slides in 10% (v/v) glycerol for imaging on a Nikon binocular microscope (Nikon SMZ18).

### Hypocotyl growth assay

Surface-sterilized Col-0 wild-type and *brl2-1* mutant^55^ seeds were cold-stratified at 4 °C for 48 h and sown on ^1/2^MS medium containing 0.8% (w/v) agar. The growth medium was supplemented with either 1 μM brassinazole^103^ prepared from a 10 mM stock solution in 100% DMSO or 0.1% (v/v) DMSO as control. After a 1 h light treatment to promote germination, the plates were wrapped in aluminium foil and maintained in complete darkness at 22 °C for 5 days. Seedlings were then scanned using a flat-bed scanner (Epson Perfection V600 Photo scanner, Epson).

### BES1 and BRI1-mCitrine immunoblotting

*Arabidopsis* seeds (Col-0 wild type, *bri1* (ref. ^47^), *brl2-1* (ref. ^55^) and T3 generation *BRI1prom*::BRI1^mut^-mCitrine complementation lines) were surface-sterilized and grown on ^1/2^MS plates containing 1% (w/v) sucrose and 0.8% (w/v) agar for 10 or 14 days, respectively. Around 250 mg of tissue samples was collected for each line in 1.5 ml tubes containing glass beads and immediately frozen with liquid nitrogen. The samples were ground with Silamat S6 (Ivolar Vivodent) and then mixed with extraction buffer (50 mM Tris–HCl pH 7.5, 150 mM NaCl, 1 mM EDTA, 1% (v/v) Triton X-100, 1× cOmplete EDTA-free protease inhibitor cocktail (11873580001; Roche), 1× plant protease inhibitor cocktail (P9599; Sigma-Aldrich) and 1× PhosStop (4906845001; Roche)). After protein solubilization for 30 min on ice, cell debris was removed by centrifugation (14,000*g*, 2 × 15 min, 4 °C), and the supernatant of each sample was transferred to a fresh tube. Protein concentration was determined using Pierce BCA Protein Assay Kit (23225; Thermo Fisher Scientific). For BRI1-mCitrine fusion protein detection, an anti-GFP antibody coupled to horseradish peroxidase (anti-GFP-HRP, 130-091-833; Miltenyi Biotec) at a dilution of 1:5,000 was applied. Alternatively, BRI1 was detected using a primary BRI1 antibody^104^ (1:5,000 dilution) and a secondary anti-rabbit IgG–peroxidase antibody produced in goat (1:10,000 dilution, A6154; Sigma-Aldrich). BES1 dephosphorylation levels were estimated using a primary anti-BES1 antibody^41^ at a dilution of 1:2,000 and a secondary anti-rabbit IgG–peroxidase antibody produced in goat (1:10,000 dilution, A6154; Sigma-Aldrich). SuperSignal West Pico PLUS Chemiluminescent Substrate (34577; Thermo Fisher Scientific) was used for chemiluminescence detection on an Amersham Imager 680 (GE). ReadyBlue Protein Gel Stain (RSB-1L; Sigma-Aldrich) was used for the total protein input detection.

### Analytical size-exclusion chromatography

The oligomeric state of recombinantly expressed and purified wild-type and point-mutant BRI1 ectodomains was characterized using analytical size-exclusion chromatography; 500 µl sample at 1.0 mg ml^−1^ was loaded onto a Superdex 200 Increase 10/300 GL column (Cytiva) pre-equilibrated in 20 mM citrate buffer (pH 5.0), with 150 mM NaCl. Protein elution at a flow rate of 0.7 ml min^−1^ was monitored by UV absorption at 280 nm.

### Quantitative real-time PCR

Expression of the BR marker genes CPD and DWF4 was estimated by qRT-PCR, using ~30 individual 8-day-old seedlings per each T3 generation transgenic line. Samples were homogenized by Silamat S6 (Ivolar Vivodent), and RNA was extracted using a RNeasy Plant Mini Kit (74904; Qiagen). Subsequently, cDNA synthesis was performed with the SuperScript II Reverse Transcriptase (18064014; Invitrogen), following the manufacturer’s protocol. Quantitative real-time PCR was conducted on a QuantStudio 5 thermo-cycler (Applied Biosystems) using SYBR Select Master Mix for CFX (4472942; Applied Biosystems). The relative expression (*n* = 3) was quantified with the 2 –ΔΔCt method. ACT2 was used as the internal reference gene. qPCR primers are included in Supplementary Table 4.

### Reporting summary

Further information on research design is available in the Nature Portfolio Reporting Summary linked to this article.

## Supplementary information

Supplementary Information Supplementary Figs. 1–6, Supplementary References, Note 1: chemical synthesis and analysis of BRs, and uncropped western blots and stained membranes.

Reporting Summary

Peer Review file

Supplementary Table 1 Crystallographic data collection and refinement statistics.

Supplementary Table 2 List of genotyping primers used in this study.

Supplementary Table 3 Cloning primers used in this study.

Supplementary Table 4 qPCR primers for BR marker genes CPD and DWF4.

Supplementary Table 5 Immobilized receptor density and analyte concentrations used for the GCI assays in Fig. 2a,c,d, Fig. 4e, Fig. 5b,c and Fig. 6b–f.

Supplementary Table 6 BR-mediated root growth inhibition raw data corresponding to Fig. 3c.

Supplementary Table 7 BR-mediated root growth inhibition raw data corresponding to Fig. 6g.

Supplementary Table 8 BR-mediated root growth inhibition raw data corresponding to Fig. 5f.

Supplementary Table 9 Raw reads used to generate the heat maps shown in Fig. 3e.

## Source data

Source Data Fig. 1 Source data for Fig. 5e (uncropped western blots and Coomassie stained membranes).

## Data Availability

Raw RNA-seq reads have been deposited with the NCBI Sequence Read Archive with identifier PRJNA1299306 (https://www.ncbi.nlm.nih.gov/sra/PRJNA1299306). BRI1, BRL2 and BRL3 complex structures have been deposited with the Protein Data Bank (http://www.rcsb.org) with accession numbers 9S80 (BRL3, brassinolide), 9S87 (BRI1, castasterone), 9S8S (BRI1, typhasterol), 9S8V (BRI1, 24-epibrassinolide), 9S8Z (BRI1, 28-homobrassinolide), 9S90 (BRL3, castasterone), 9S96 (BRL3, typhasterol), 9S9A (BRL3, 6-deoxocastasterone), 9S9C (apo BRL2, tetragonal crystal form) and 9U0G (apo BRL2, monoclinic crystal form). Source data are provided with this paper.
