# Supplementary material for: A mechanistic framework for the recognition of chemically diverse brassinosteroids by BRI1-family receptor kinases
Source: Nat Plants. 2026 Aug 5;12(8):1579–95. doi: 10.1038/s41477-026-02346-0 (PMC13481262; doi:10.1038/s41477-026-02346-0)
Supplement: Supplementary file 1 [file 41477_2026_2346_MOESM13_ESM.pdf]

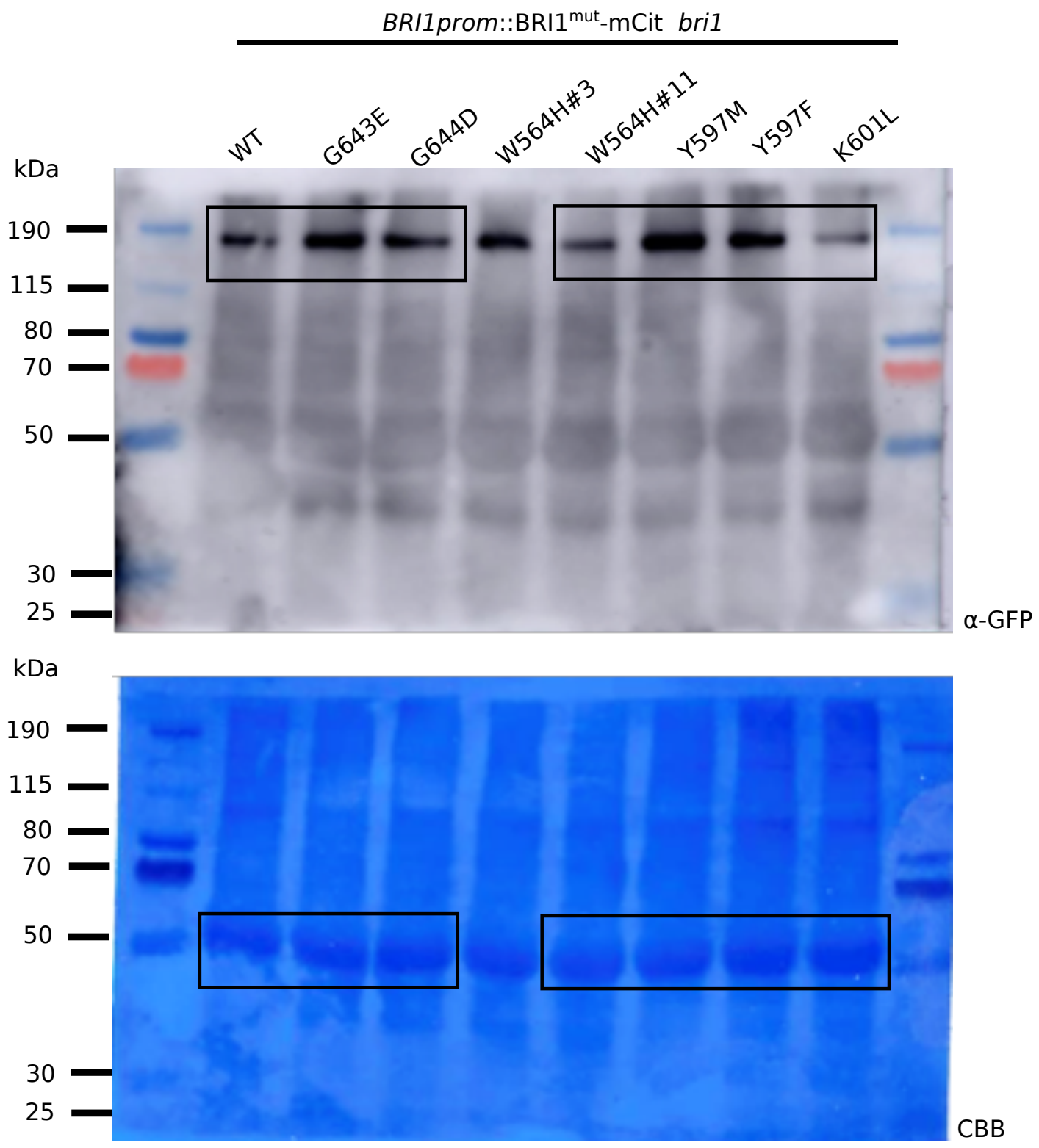

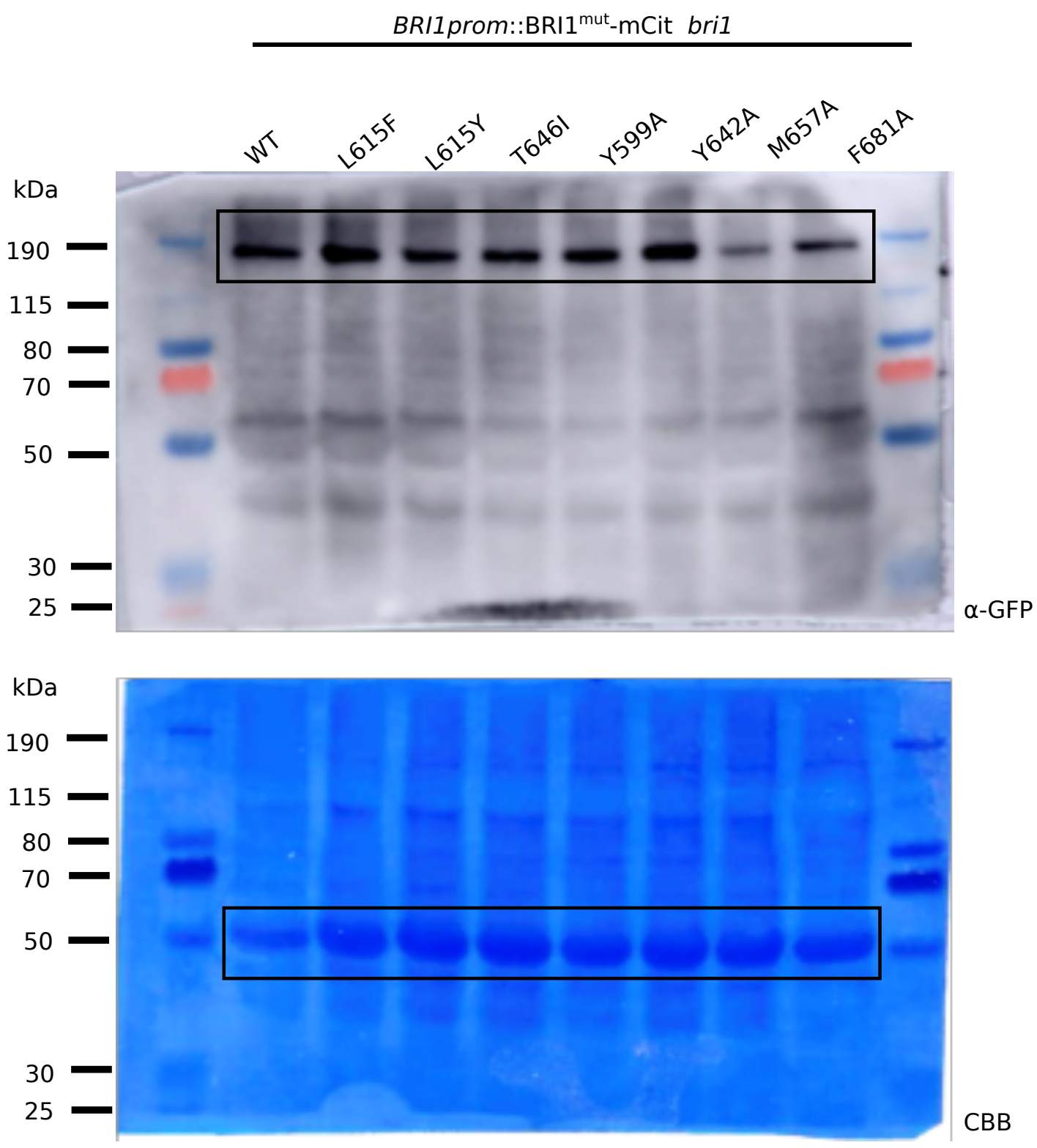

Source Data for Fig. 5e (uncropped western blots and coomassie stained membranes)
