# Supplementary material for: A mechanistic framework for the recognition of chemically diverse brassinosteroids by BRI1-family receptor kinases
Source: Nat Plants. 2026 Aug 5;12(8):1579–95. doi: 10.1038/s41477-026-02346-0 (PMC13481262; doi:10.1038/s41477-026-02346-0)
Supplement: Supplementary file 2 [file 41477_2026_2346_MOESM1_ESM.pdf]

nature plants

Supplementary information https://doi.org/10.1038/s41477-026-02346-0

A mechanistic framework for the recognition of chemically diverse brassinosteroids by BRI1-family receptor kinases

In the format provided by the authors and unedited

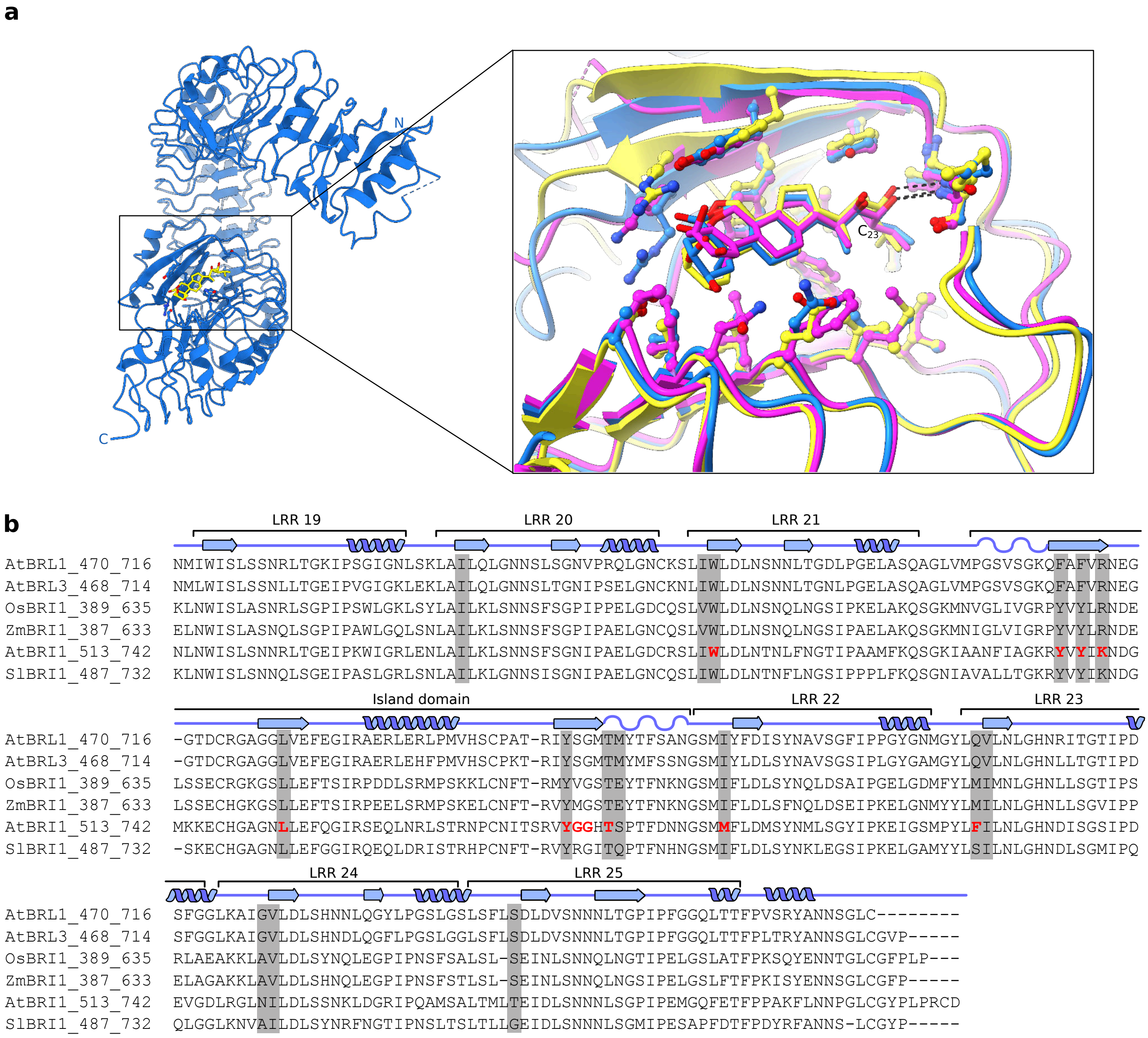

Supplementary Fig. 1 | BRI1, BRL1 and BRL3 share a structurally conserved BR-binding pocket. a, Right: Ribbon diagram of the BRL3 ectodomain bound to brassinolide (yellow, bonds representation). Left: Close-up of a structural superposition of the BR-binding pockets of BRL3 (blue), BRI1 (purple; r.m.s.d. ~ 0.7 Å over 172 C_{α} atoms), and BRL1 (yellow; r.m.s.d. ~ 0.6 Å over 166 C_{α} atoms). Key residues involved in brassinolide coordination are shown in ball-and-stick representation. The conserved hydrogen bond between the C_{23} hydroxyl group of brassinolide and the island domain backbone is indicated with a black dotted line. **b,** Multiple sequence alignment of BR-binding pocket regions from *Arabidopsis thaliana* BRI1, BRL1, and BRL3, along with orthologs from rice (OsBRI1, https://www.uniprot.org/ UniProt-ID Q942F3), maize (ZmBRI1, A0A3L6FIW6), and tomato (SlBRI1, F2XYF6), generated using MUSCLE[105]. Secondary structure elements based on AtBRI1 and calculated with DSSP[106] are shown above the alignment. BR-contacting residues (compare Fig. 2b) are highlighted in gray; residues mutated in this study (compare Fig. 5a,c) are shown in red.

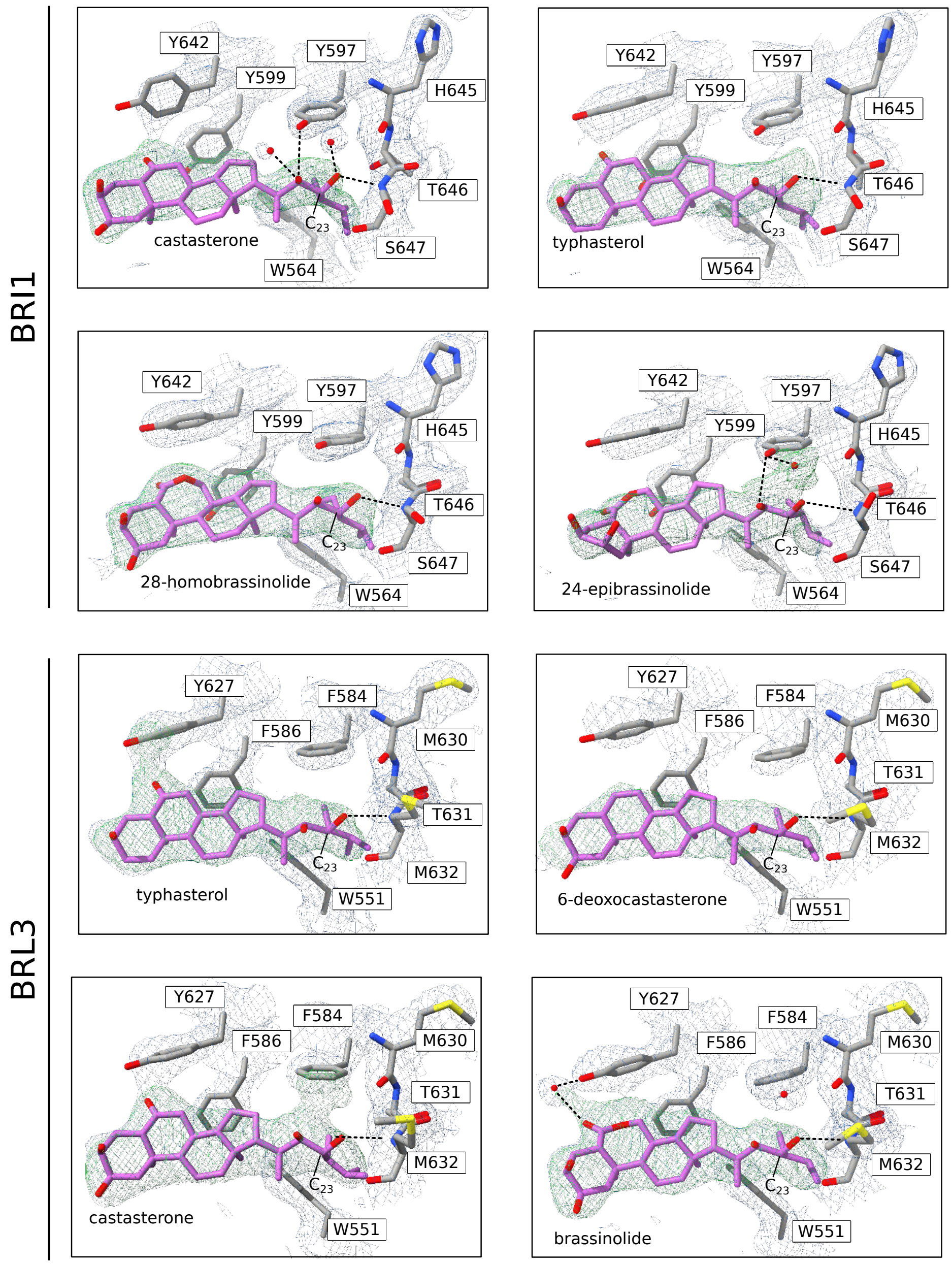

Supplementary Fig 2. | Details of the BRI1 and BRL3 steroid hormone binding pockets bound to different BRs. Shown are the steroid binding sites of BRI1 (top panels) and BRL3 (bottom panels) bound to the different brassinosteroids (in bonds representation, in purple) described in Fig. 3a,b. Residues in contact with the ligand are shown alongside (in gray), water molecules are depicted as red spheres and hydrogen bond interactions as dotted lines (in black). Final $2mF_o$-DF_c (gray mesh) and mF_o-DF_c (green mesh) omit maps were contoured at 1 and 3 σ, respectively. Given the moderate resolution of several datasets, the level of detail observable in the electron density maps limits the precision with which the exact orientation of the BR side-chain could be modeled with confidence.

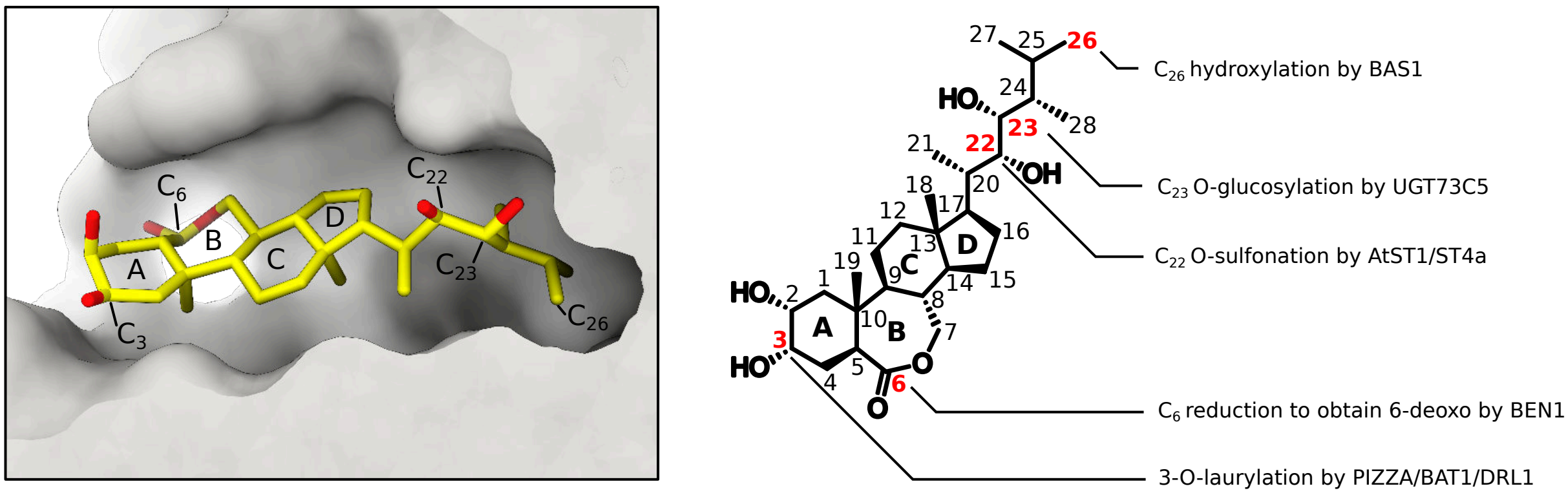

Supplementary Fig 3. | BR catabolic enzymes modify BRs at positions critical for receptor and co-receptor binding. Left panel: Molecular surface view of the BRI1 hormone binding pocket (in gray) occupied by brassinolide (in bonds representation, in yellow; PDB-ID: pdb_00003rj0). The A, B, C, and D rings that make up the hormone's steroid core are labeled and carbon atoms targeted for catabolic modifications are highlighted. Right panel: Chemical structure of brassinolide with carbon numbering. Red, bold numbers indicate sites of BR catabolic reactions: C_{26} hydroxylation by BAS1[17], C_{23} O-glucosylation by UGT73C5[18], C_{22} O-sulfonation by AtST1/ST4a[19], C_6 reduction by BEN1[107] and C_3 O-laurylation by PIZZA/BAT1/DRL1[108–110].

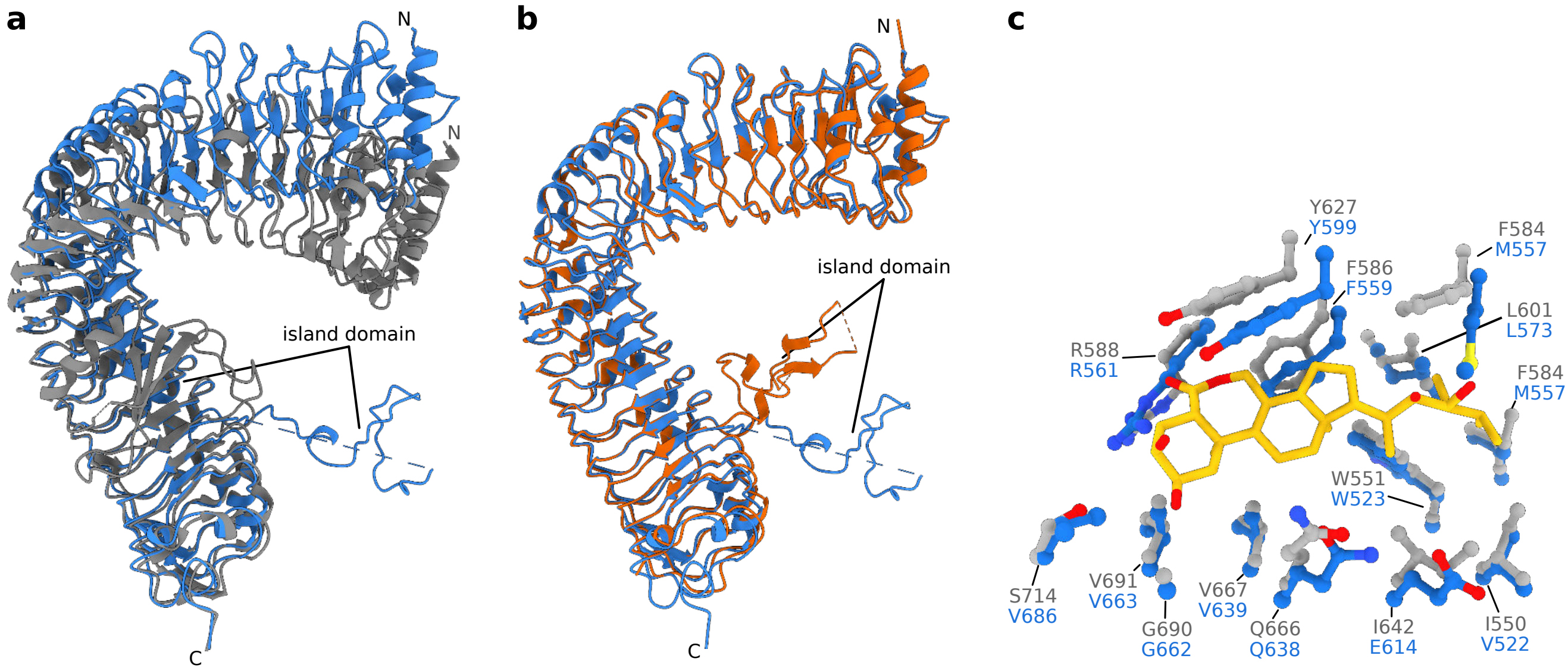

Supplementary Fig 4. | BRL2 features structurally conserved LRR and island domains. a, Structural superposition of the BRI1 ectodomain (ribbon diagram, in gray; PDB-ID: pdb_00003rj0) and BRL2 (monoclinic crystal form, in blue, , r.m.s.d. is ~0.8 Å comparing 318 corresponding C_α atoms). The island domain of BRL2 appears partially disordered and is engaged in a domain-swapped interaction with a neighboring molecule. **b,** Structural superposition of the tetragonal and monoclinic crystal forms of the BRL2 ectodomain (tetragonal isoform in orange, monoclinic isoform in blue, r.m.s.d. is ~0.65 Å comparing 646 corresponding C_α atoms). **c,** Close-up view of the BRL2 BR-binding pocket (in blue, AlphaFoldDB-ID: AF-Q9ZPS9-F1) superimposed with BRL3 (right panel, in gray, brassinolide in bonds representation and in yellow, r.m.s.d. is ~ 0.8 Å comparing 349 C_α atoms). Residues lining the BR binding site are shown in ball-and-stick representation.

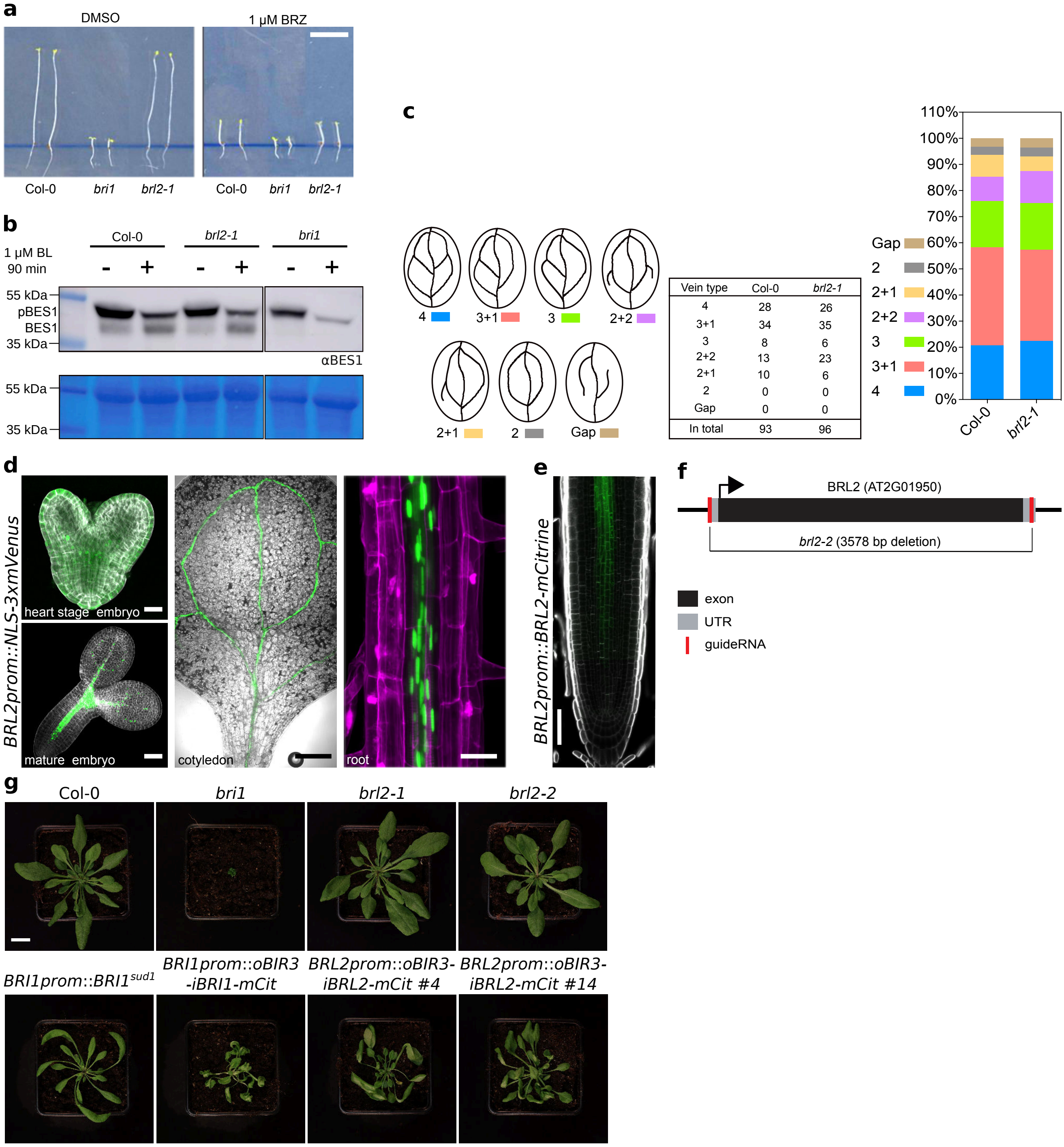

Supplementary Fig. 5 | BRL2 is a vascular receptor kinase with no detectable function in BR signaling. a, Hypocotyl growth assay of dark-grown brassinazole (BRZ)[103] treated vs. untreated control seedlings. Seeds of Col-0 and *brl2-1* were germinated and grown for 5 d on $^{1/2}$MS plates supplemented with 1 µM BRZ or 0.1% (v/v) DMSO (scale bar = 1 cm). **b,** BES1 (theoretical molecular mass ~ 39.2 kDa) western blot. 14-d-old Col-0, *brl2-1* and *bri1* seedlings were grown on $^{1/2}$MS plates with 1% (w/v) sucrose, transferred to $^{1/2}$MS liquid medium containing 1% (w/v) sucrose and either 1 µM brassinolide (BL) or 0.01 % DMSO (as control) for 90 min, and probed with an anti-BES1 antibody[41]. **c,** Cotyledon vein patterning analysis performed using 2-week-old Col-0 and *brl2-1* seedlings. Leaf patterns are categorized in seven groups: 4 (4 distinct regions are formed by the intersection of the leaf veins, blue), 3+1 (3 distinct regions formed with 1 free branch, beige), 3 (3 distinct regions formed, lemon green), 2+2 (2 distinct regions formed with 2 free branches, light purple), 2+1 (2 distinct regions formed with 1 free branch, canary), 2 (2 distinct regions formed, gray) and gap (no distinct region formed, earthy yellow). **d,** Confocal images of a representative *BRL2prom*::NLS-3xmVenus reporter (in green) line in different stages: heart-stage embryo, mature embryo (cell wall stained with Renaissance SR2200, in gray; scale bar = 20 µm), cotyledon (scale bar = 200 µm) and root from a 10-d-old seedling (cell wall stained with propidium iodide, magenta, scale bar = 30 µm). **e,** Confocal imaging of a representative *BRL2prom:*BRL2-mCitrine line. Shown is the primary root from a 10-d-old seedling (cell wall stained with Renaissance SR2200, in gray; scale bar = 50 µm). **f,** Schematic diagram of the *brl2-2* CRISPR/Cas9 allele. The BRL2 genomic locus is shown, including the translation start codon (black arrow), exon (black rectangle) and 5′ and 3′- primer untranslated regions (UTRs, gray blocks). The putative binding sites of the designed guide RNAs are indicated in red. **g,** Rosette phenotypes of 5-week-old plants, germinated on $^{1/2}$MS for 2 weeks before transferring to soil for an additional 3 weeks (scale bar = 2 cm).

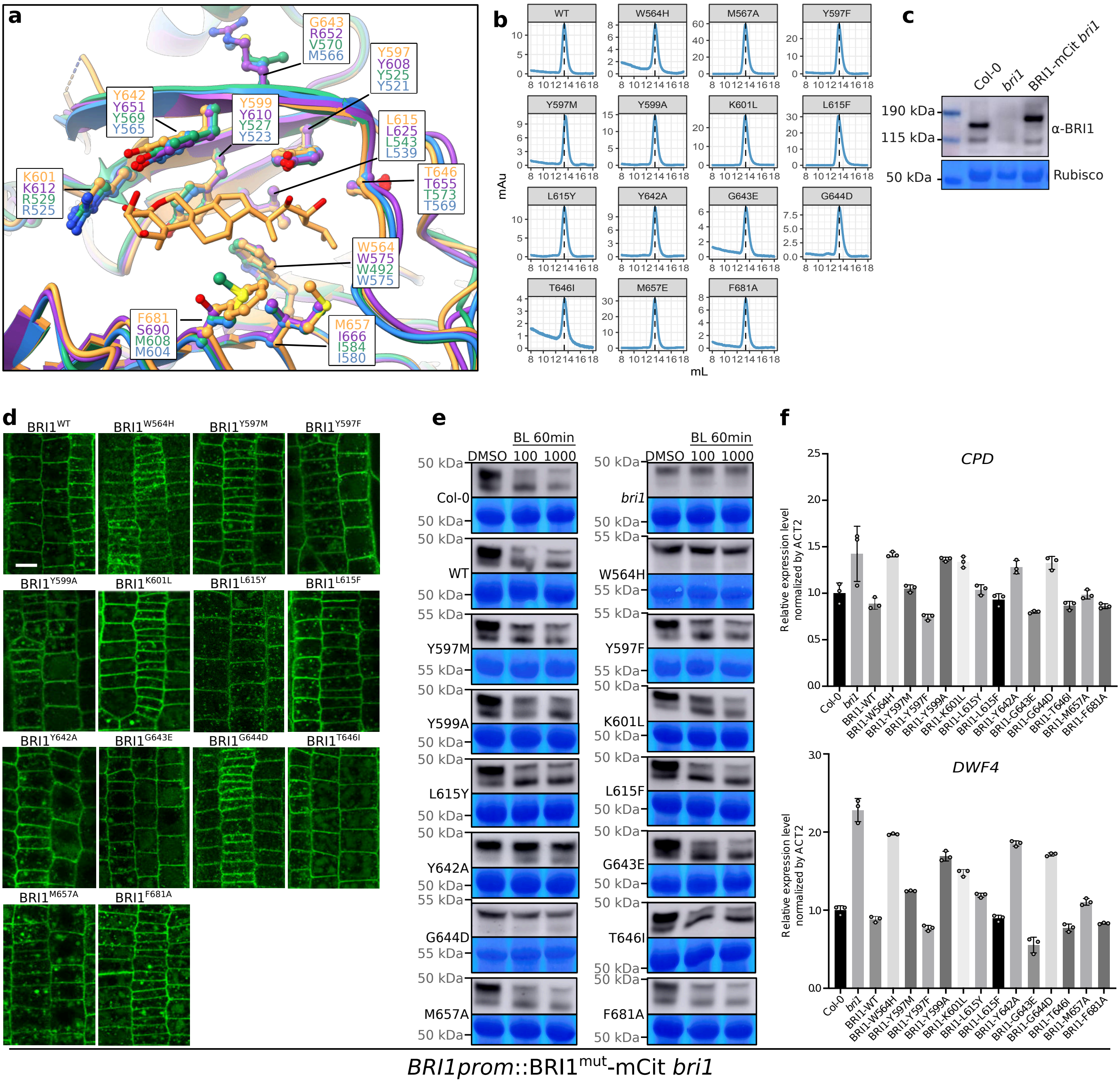

Supplementary Fig. 6 | Mutations of conserved residues in the BRI1 hormone binding pocket alter BR signaling without compromising the plasma-membrane localization of the mutant receptor. a, Structural superposition of the brassinolide-bound AtBRI1 hormone binding pocket (Arabidopsis thaliana, in orange, PDB-ID: pdb_00003rj0) with the corresponding regions in ZmBRI1 (Zea mays, in blue, AlphaFoldDB-ID: AF-A0A3L6FIW6-F1, r.m.s.d ~ 0.7 Å comparing 175 corresponding C_α atoms), SlBRI1 (Solanum lycopersicum, in purple, AlphaFoldDB-ID: AF-F2XYF6-F1, r.m.s.d ~ 0.6 Å between 178 C_α atoms), and OsBRI1 (Oryza sativa, in green, AlphaFoldDB-ID: AF-Q942F3-F1, r.m.s.d ~ 0.7 Å between 170 C_α atoms). Mutated residues in AtBRI1 are displayed as ball-and-stick models, the corresponding amino acid side-chains from ZmBRI1, SlBRI1, and OsBRI1 are included. **b,** Analytical size-exclusion chromatography elution profiles for recombinant wild-type (WT) and point-mutant BRI1 ectodomains used in the GCI assays. **c,** BRI1 protein level in Col-0, *bri1* and *BRI1prom*::BRI1-mCitrine / *bri1* lines, detected with a primary anti-BRI1 antibody[104]. ReadyBlue-stained RuBisCO is shown as loading control below. **d,** Confocal imaging of T2 generation *BRI1prom*::BRI1^{mut}-mCitrine signal in the mersistematic zone of 10-d-old primary roots mounted for confocal imaging (scale bar = 20 µm). **e,** Brassinolide (BL)-stimulated BES1 dephosphorylation. 14-d-old seedlings of Col-0 wild-type, *bri1* and *BRI1prom*::BRI1^{mut}-mCit T3 complementation lines were treated with 0.1% (v/v) DMSO or 100 nM BL and 1000 nM BL for 60 min before protein extraction and anti-BES1 antibody[41] immuno-blotting. The upper band represents phosphorylated BES1, the lower band (more) dephosphorylated BES1. ReadyBlue-stained RuBisCO is shown as loading control below. **f,** qRT-PCR analysis of the BR marker genes *CPD* and *DWF4* in Col-0 wild-type, bri1 and *BRI1prom*::BRI1^{mut}-mCit T3 complementation, with *ACT2* used as internal reference gene. Each dot represents a biological replicate (n=3).

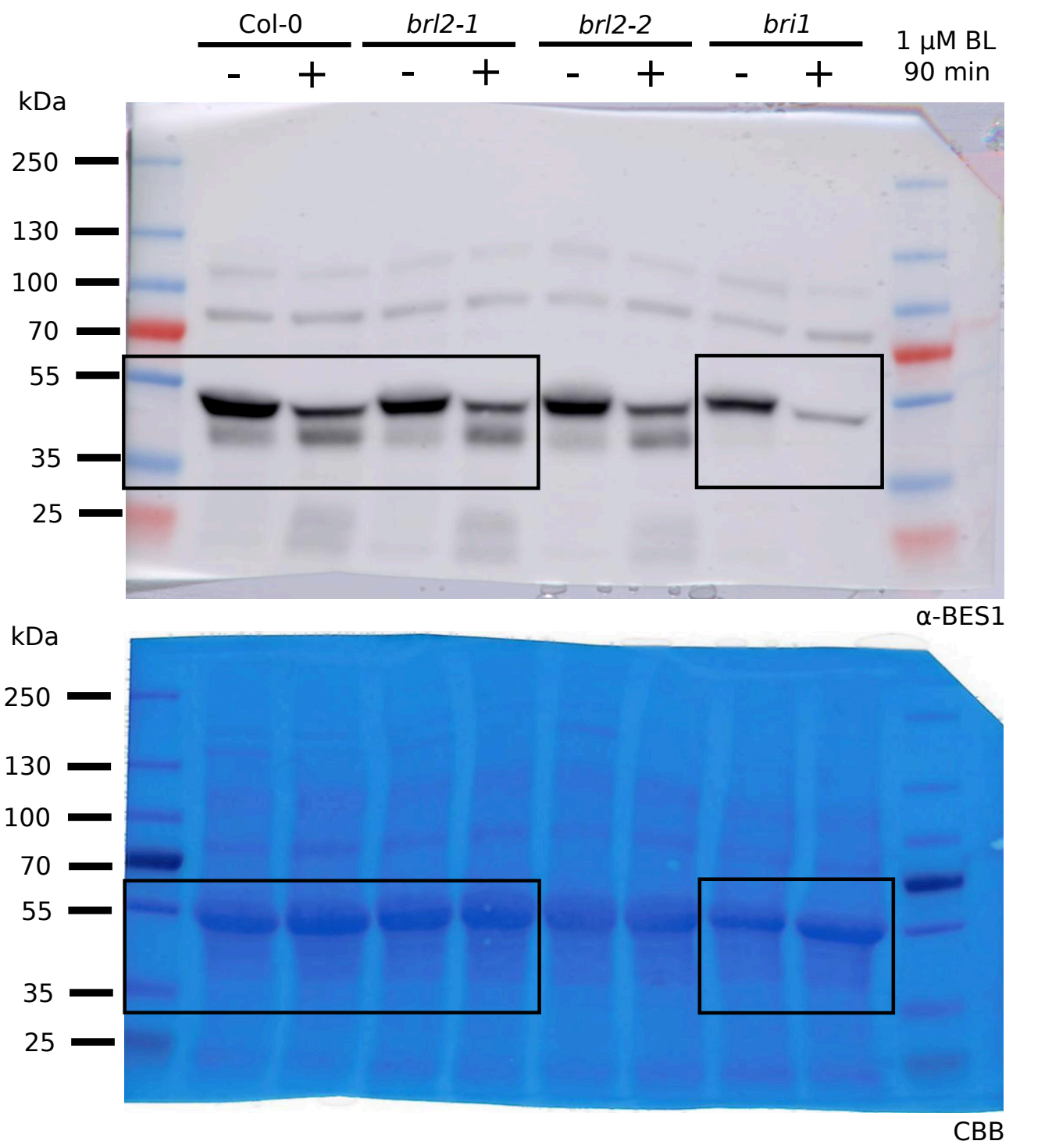

Uncropped western blots and coomassie stained membranes for Supplementary Fig. 5b

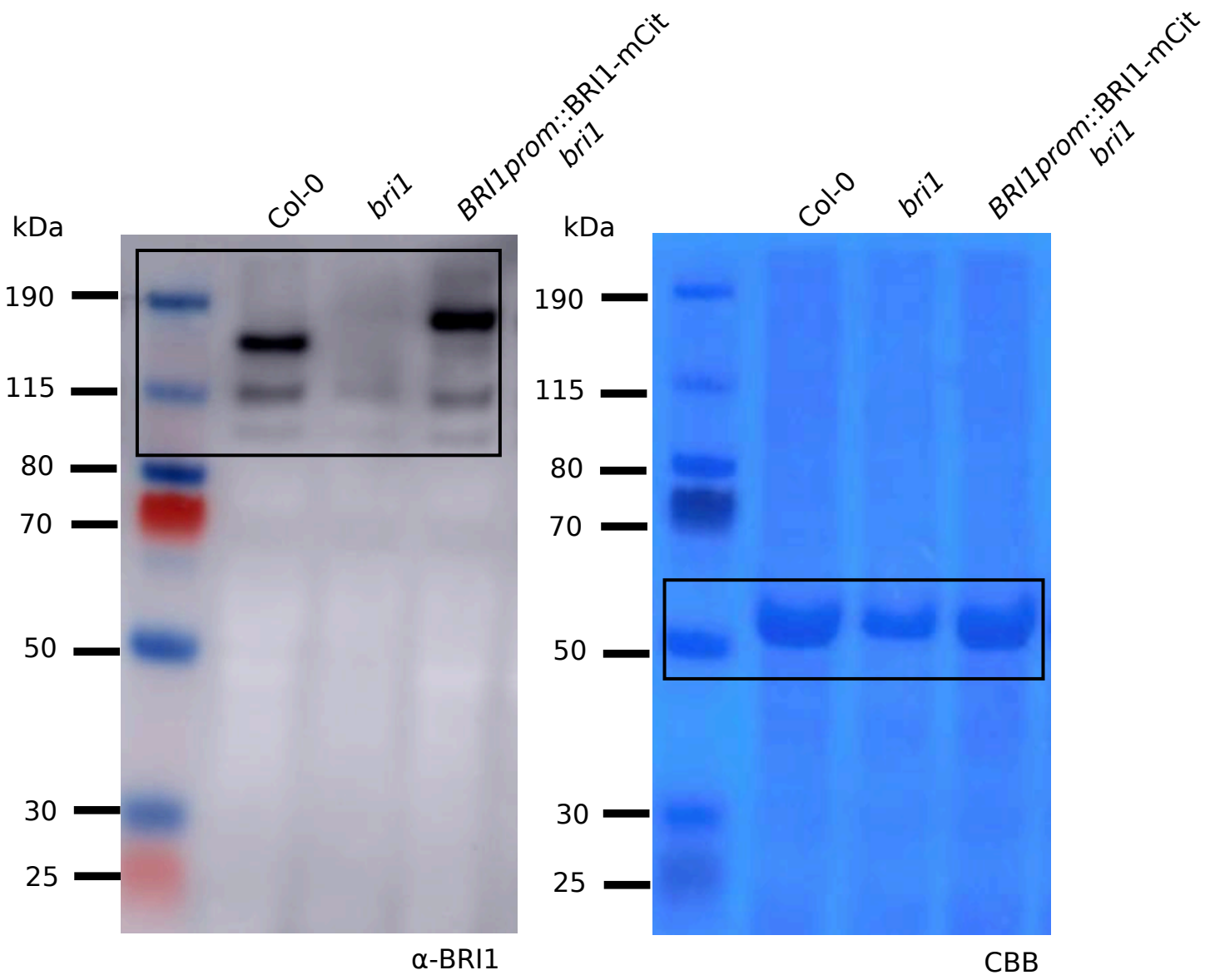

Uncropped western blots and coomassie stained membranes for Supplementary Fig. 6c

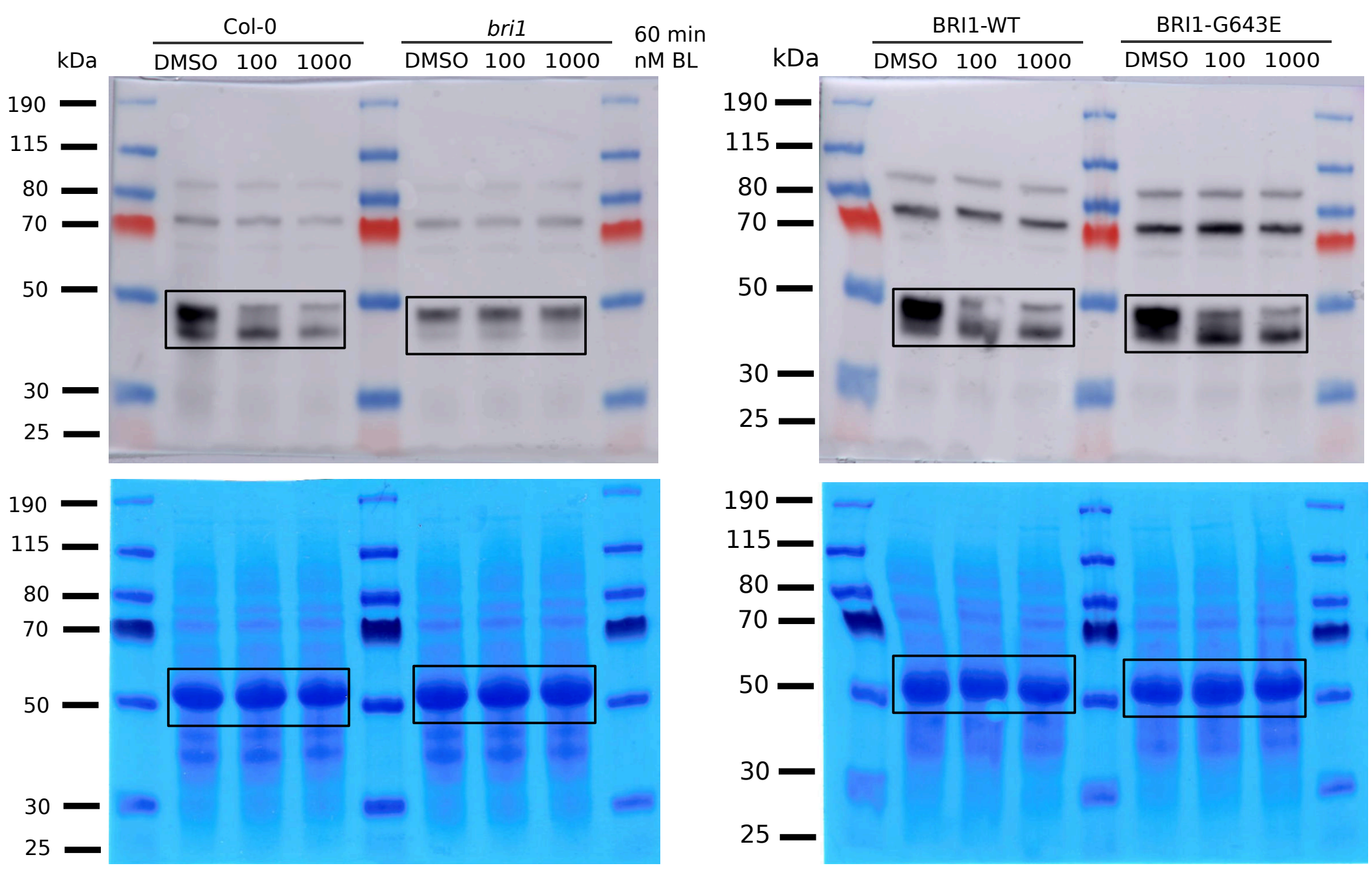

Uncropped western blots and coomassie stained membranes for Supplementary Fig. 6e

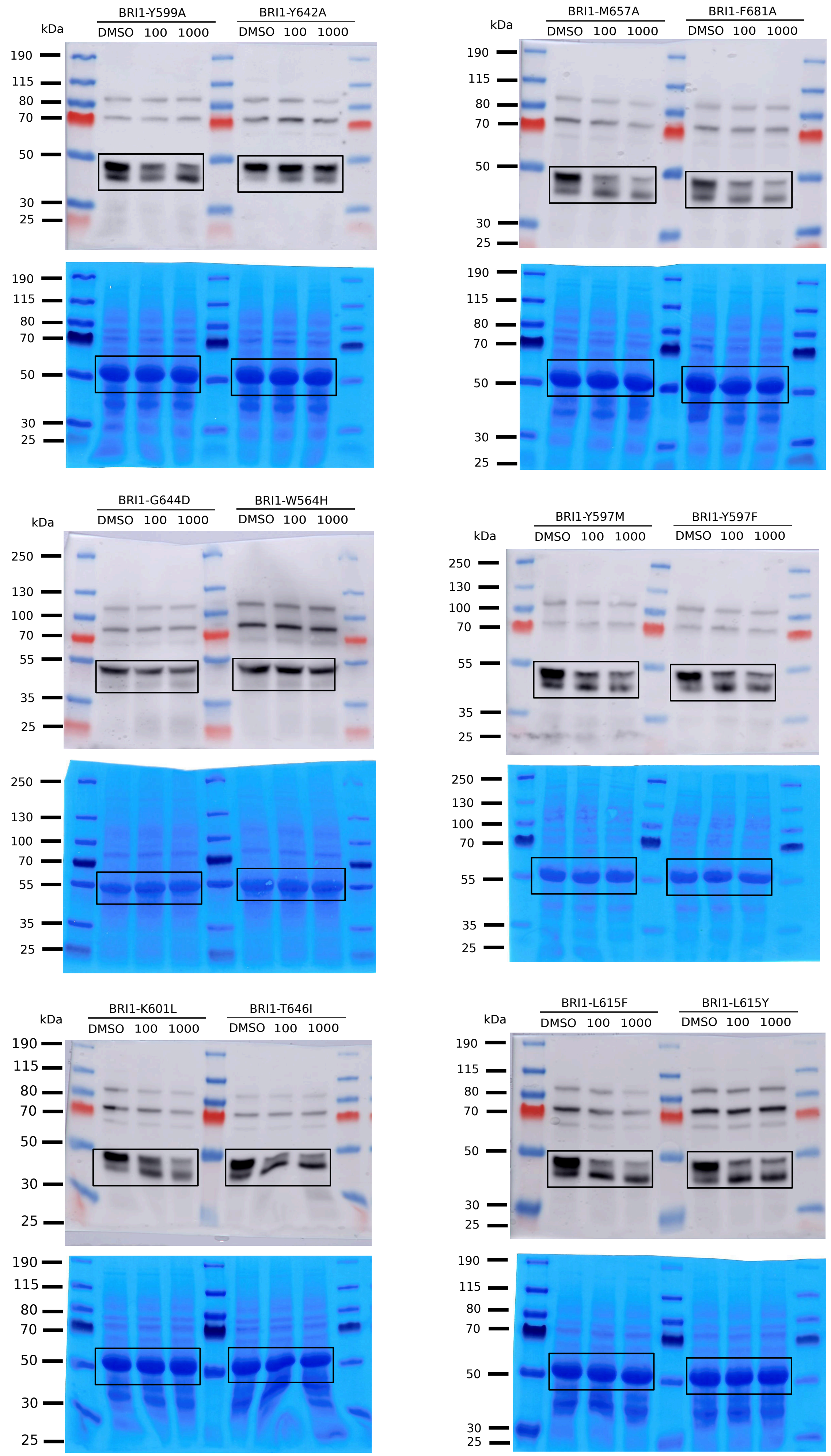

Uncropped western blots and coomassie stained membranes for Supplementary Fig. 6e (continued)

Supplementary References

105. Edgar, R. C. MUSCLE: a multiple sequence alignment method with reduced time and space complexity. *BMC Bioinformatics* **5**, 113 (2004).

106. Kabsch, W. & Sander, C. Dictionary of protein secondary structure: pattern recognition of hydrogen-bonded and geometrical features. *Biopolymers* **22**, 2577–2637 (1983).

107. Yuan, T. et al. BEN1, a gene encoding a dihydroflavonol 4-reductase (DFR)-like protein, regulates the levels of brassinosteroids in *Arabidopsis thaliana*. *Plant J.* **51**, 220–233 (2007).

108. Schneider, K. et al. *Arabidopsis* PIZZA has the capacity to acylate brassinosteroids. *PLoS ONE* **7**, e46805 (2012).

109. Choi, S. et al. BAT1, a putative acyltransferase, modulates brassinosteroid levels in *Arabidopsis*. *Plant J.* **73**, 380–391 (2013).

110. Zhu, W. et al. Homeostasis of brassinosteroids regulated by DRL1, a putative acyltransferase in *Arabidopsis*. *Mol. Plant* **6**, 546–558 (2013).

Supplementary Note 1: Chemical synthesis and analysis of BRs

General methods

The NMR spectra were taken on a JEOL JNM-ECA 500 (JEOL, Tokyo, Japan; ^{1}H, 500 MHz; ^{13}C, 125 MHz) spectrometer equipped with a 5mm JEOL Royal probe. ^{1}H NMR and ^{13}C NMR chemical shifts (δ) were calibrated using tetramethylsilane (TMS, ^{1}H δ=0 ppm) or solvents: $CDCl_3$ (^{1}H δ=7.27 ppm, ^{13}C δ=77.00 ppm) or DMSO-d_6 (^{1}H δ=2.46 ppm, ^{13}C δ=40.00 ppm). Chemical shifts are given in ppm (δ-scale), coupling constants (J) in Hz. All values were obtained by first order analysis. The NMR data were processed using the ACD/NMR Processor Academic Edition (ver. 12.01). For ESI or APCI HRMS analysis, the samples were dissolved in chloroform or methanol. Samples were analyzed with 1290 Infinity II Liquid Chromatographer (Agilent) with HPLC column infinityLab Poroshell 120 EC-C18 (4.6×50 mm; 2.7 μm). For API HRMS analysis, the samples were dissolved in chloroform (or chloroform:methanol; 1:1; v/v, in the case of hydroxylated compounds) to a concentration 10 $\mu g \cdot mL^{-1}$. The ASAP (Atmospheric Solids Analysis Probe) was dipped into the sample solution, placed into the ion source and analyzed in full scan mode. The source of the Synapt G2-Si Mass Spectrometer (Waters, Manchester, UK) was operated in positive ionization mode (ASAP+) and, if not stated otherwise, at source temperature of 120 °C. The Corona needle current was kept at 5 μA and the collision energy at value 4. The probe temperature was ramped up from 50 °C to 600 °C in 3 min. Data were acquired from 50 to 1000 Da with 1.0 s scan time in High Resolution Mode and processed using the Masslynx 4.1 software (Waters). Mass accuracy of 1 ppm or less was achieved with the described instrumentation for all compounds.

Merck silica gel Kieselgel 60 (230–400 mesh) was used for column chromatography. The HPLC system consisted of a Waters semi-preparative HPLC system including quaternary pump, liquid handler, UV-VIS and ELSD detectors. The HPLC semi preparative column was filled with silica gel. Reagents and solvents were purchased from Sigma–Aldrich and were not purified.

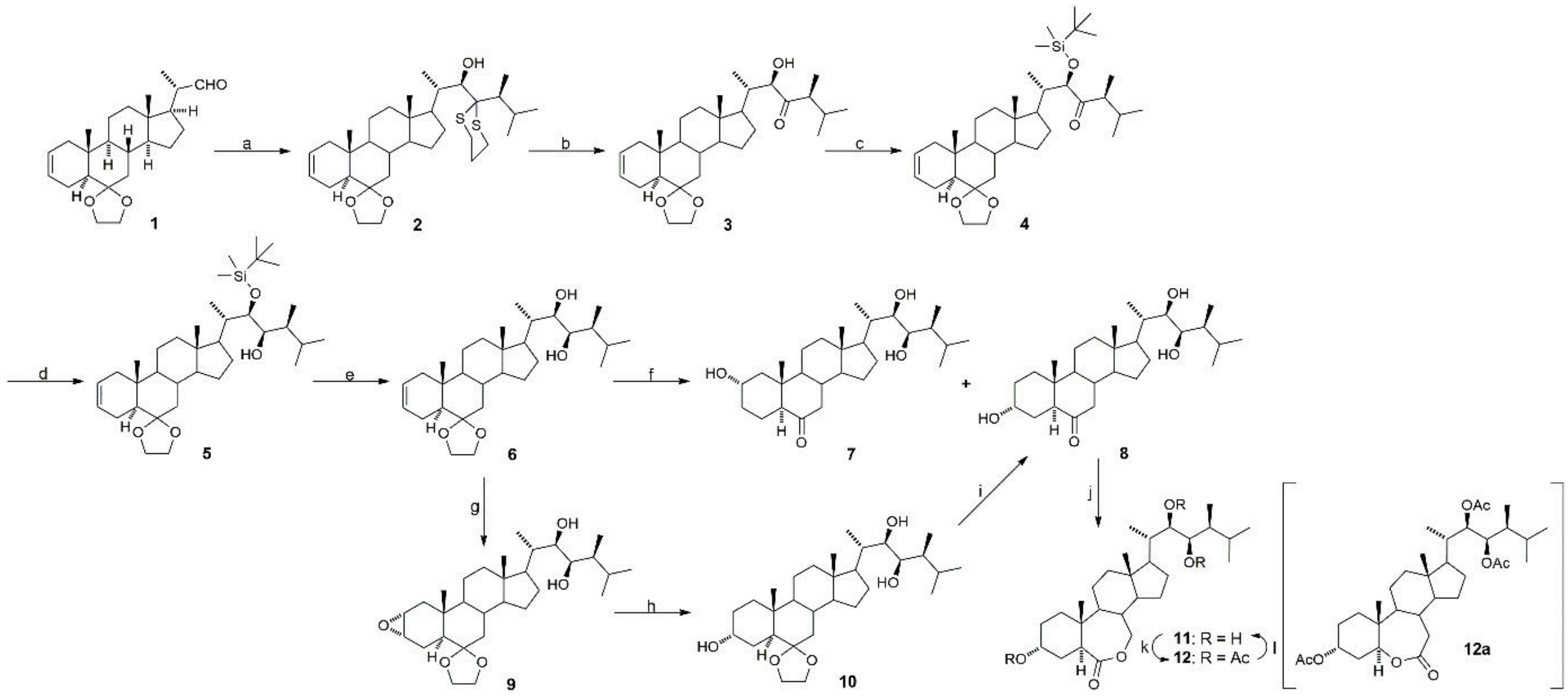

Scheme 1: Synthesis of 3-deoxycastasterone (**7**), typhasterol (**8**), and 2-deoxybrassinolide (**11**). **a**: (*S*)-2-(3-methylbutan-2-yl)-1,3-dithiane/n-BuLi/THF, r.t. then -78 °C, 1h; **b**:

NaH_2PO_4/2-methyl-2-butene/ $NaClO_2$/H_2O/ethanol, 0 °C, 10 min; **c**: TBSOTf/2,6-lutidine/CH_2Cl_2, 0 °C, 1 h; **d**: DIBAL-H/toluen, -78 °C, 1h; **e**: TBAF/THF, r.t., 1h; **f**: 1, BH_3/THF, r.t., 6h; 2, NaOH/H_2O_2/H_2O/THF, r.t., 12h; **g**: MCPBA/EtOAc, r.t., 4h; **h**: $LiAlH_4$/THF/Et_2O, 40 °C, 3h; **i**: HCl/H_2O/THF, 40 °C, 4h; **j**: TFPAA/CH_2Cl_2, r.t., 4h; **k**: pyridine/Ac_2O, 50 °C, 12h; **l**: MeONa/MeOH/THF, r.t., 1h. Note: absolute configuration for carbons 8, 9, 13, and 17 is shown only for compound **1** but is identical for all compounds prepared.

Synthesis of (22*R*)-6,6-ethylenedioxy-23,23-(trimethylenedithio)-5α-campest-2-en-22-ol (2)

1.6 M solution of *n*-BuLi in hexane (3.0 mL, 4.8 mmol) was added to a solution of (*S*)-2-(3-methylbutan-2-yl)-1,3-dithiane[1] (790 mg, 4.0 mmol) in THF (15 mL) at room temperature. The mixture was stirred for 30 minutes and then cooled to -78 °C. A solution of aldehyde **1**[2] (1.0 g, 2.69 mmol) in THF (3 mL) was added dropwise to the reaction mixture. The solution was stirred at -78 °C for 30 min, quenched with saturated aqueous NH_4Cl (20 mL), and warmed to room temperature. The product was extracted with ethyl acetate. The organic layer was washed with water followed by drying over $MgSO_4$, filtrated and evaporated under reduced pressure. The residue was purified by column chromatography on silica gel (3-5% ethyl acetate in cyclohexane) to give alcohol **2** (1.22 g, 81 %) as a viscous oil. ^{1}H NMR (500 MHz, DMSO-d_6): δ 5.63 (m, 1H, H-3), 5.51 (m, 1H, H-2), 4.50 (d, 1H, J = 6.4 Hz, H-22), 3.92-3.79 (m, 3H, H_{ketal}), 3.69 (q, 1H, J = 6.6 Hz, H_{ketal}), 2.83-2.60 (m, 5H), 2.12-1.69 (m, 11H), 1.62-1.23 (m, 8H), 1.15-0.75 (m, 4H), 1.11 (d, 3H, J = 6.4 Hz, CH_3), 0.99 (d, 3H, J = 7.0 Hz, CH_3), 0.89 (d, 3H, J = 6.7 Hz, CH_3), 0.86 (d, 3H, J = 6.7 Hz, CH_3), 0.80 (s, 3H, CH_3), 0.66 (s, 3H, CH_3). ^{13}C NMR (125 MHz, DMSO-d_6): 125.45, 124.86, 108.99, 73.86, 65.27, 63.78, 63.56, 55.49, 53.79, 52.49, 47.57, 43.26, 42.13, 40.80, 40.62, 39.46, 38.13, 35.37, 32.99, 27.73, 27.25, 26.33, 25.51, 24.75, 24.39, 23.73, 21.07, 20.49, 18.44, 14.58, 13.53, 11.59, 9.87. HRMS (ESI+) calculated for $C_{33}H_{55}O_3S_2$ ($[M + H]^+$) 563.3593, found 563.3597.

Synthesis of (22*R*)-6,6-ethylenedioxy-22-hydroxy-5α-campest-2-en-23-one (3)

A solution of NaH_2PO_4 (511 mg, 4.26 mmol) in water (11 mL), 2-methyl-2-butene (2.25 mL, 21.3 mmol) and $NaClO_2$ (80%, 1.44 g, 16.0 mmol) were sequentially added at 0 °C to a stirred solution of compound **2** (1.2 g, 2.13 mmol) in ethanol (100 mL). The reaction mixture was stirred for 10 min and quenched with aqueous saturated $Na_2S_2O_3$ (mL). The mixture was diluted with ethyl acetate and extracted with water. The organic layer was dried over $MgSO_4$, filtrated and evaporated under reduced pressure. The residue was purified by column chromatography on silica gel (10% ethyl acetate in cyclohexane) to give hydroxyketone 3 (736 mg, 73%) as a viscous oil. ^{1}H NMR (500 MHz, $CDCl_3$): δ 5.66 (m, 1H, H-3), 5.54 (m, 1H, H-2), 4.33 (dd, 1H, J = 5.5, 1.5 Hz, H-22), 4.01-3.90 (m, 3H, H_{ketal}), 3.80 (q, 1H, J = 6.6 Hz, H_{ketal}), 3.31 (d, 1H, J = 5.5 Hz, OH), 2.45 (p, 1H, J = 7.3 Hz, H-24), 2.13-1.60 (m, 12H), 1.57-0.77 (m, 9H), 1.08 (d, 3H, J = 7.3 Hz, CH_3), 0.94 (d, 3H, J = 6.7 Hz, CH_3), 0.892 (s, 3H,

CH_3), 0.887 (d, 3H, *J* = 6.7 Hz, CH_3), 0.75 (s, 3H, CH_3), 0.74 (d, 3H, *J* = 6.7 Hz, CH_3). ^{13}C NMR (125 MHz, $CDCl_3$): 216.23, 125.64, 124.78, 110.00, 77.31, 65.61, 64.09, 55.86, 53.27, 52.52, 48.11, 48.02, 42.35, 41.15, 41.17, 39.47, 38.18, 35.85, 33.43, 28.70, 28.25, 24.00, 21.50, 21.41, 20.80, 19.03, 15.18, 13.59, 12.52, 11.91. HRMS (ESI+) calculated for $C_{30}H_{49}O_4$ ($[M + H]^+$) 473.3631, found 473.3631.

Synthesis of (22*R*)-6,6-ethylenedioxy-22-((*tert*-butyldimethylsilyl)oxy)-5α-campest-2-en-23-one (4)

TBSOTf (670 µl, 2.92 mmol) was added at 0 °C to a stirred solution of hydroxyketone **3** (700 mg, 1.48 mmol) and 2,6-lutidine (850 µL, 7.3 mmol) in CH_2Cl_2 (10 mL). The reaction mixture was stirred for 1 hour at 0 °C and then quenched with saturated aqueous $NaHCO_3$ (10 mL). The mixture was diluted with ethyl acetate and extracted with water. The organic layer was dried over $MgSO_4$, filtrated and evaporated under reduced pressure. The residue was purified by column chromatography on silica gel (5% ethyl acetate in cyclohexane) to give silyl ether **4** (811 mg, 94%) as a viscous oil. ^1H NMR (500 MHz, $CDCl_3$): δ 5.67 (m, 1H, H-3), 5.55 (m, 1H, H-2), 4.31 (d, 1H, *J* = 0.9 Hz, H-22), 4.02-3.90 (m, 3H, H_{ketal}), 3.80 (q, 1H, *J* = 6.6 Hz, H_{ketal}), 2.38 (dq, 1H, *J* = 8.6, 7.0 Hz, H-24), 2.14-1.60 (m, 11H), 1.57-0.75 (m, 10H), 1.02 (d, 3H, *J* = 7.0 Hz, CH_3), 0.93 (s, 9H, 3×CH_3), 0.92 (d, 3H, *J* = 6.7 Hz, CH_3), 0.89 (s, 3H, CH_3), 0.83 (d, 3H, *J* = 6.7 Hz, CH_3), 0.81 (d, 3H, *J* = 6.7 Hz, CH_3), 0.73 (s, 3H, CH_3), 0.06 (s, 3H, CH_3), -0.01 (s, 3H, CH_3). ^{13}C NMR (125 MHz, $CDCl_3$): 214.65, 125.65, 124.82, 110.03, 79.17, 65.62, 64.10, 56.17, 53.26, 52.37, 48.49, 48.08, 42.37, 41.20, 41.15, 39.62, 38.53, 35.88, 33.41, 29.11, 28.56, 26.89, 25.88 (3×C), 24.13, 21.56, 21.45, 20.79, 19.39, 15.94, 13.59, 12.87, 11.74, -4.22, -5.00. HRMS (ESI+) calculated for $C_{36}H_{63}O_4Si$ ($[M + H]^+$) 587.4496, found 587.4491.

Synthesis of (22*R*,23*R*)-6,6-ethylenedioxy-22-((*tert*-butyldimethylsilyl)oxy)-5α-campest-2-en-23-ol (5)

1M Solution of DIBAL-H in toluene (6.4 mL, 6.4 mmol) was added to a stirred solution of compound **4** (750 mg, 1.28 mmol) in dry toluene (30 mL) at -78 °C. The reaction mixture was stirred at the same temperature for 1 hour and then quenched with MeOH (6 mL). Saturated aqueous solution of sodium potassium tartrate (30 mL) was added and the mixture was diluted with ethyl acetate. The aqueous layer was separated and extracted twice with ethyl acetate. The combined organic layers were washed with water, dried over $MgSO_4$, filtrated and evaporated under reduced pressure. The residue was purified by column chromatography on silica gel (2-5% ethyl acetate in cyclohexane) to give alcohol **5** (715 mg, 95%) as a viscous oil (containing 12% of inseparable 23*S*-isomer). ^1H NMR (500 MHz, $CDCl_3$): δ 5.56 (m, 1H, H-3), 5.53 (m, 1H, H-2), 3.99-3.88 (m, 3H, H_{ketal}), 3.78 (q, 1H, *J* = 6.6 Hz, H_{ketal}), 3.58 (d, 1H, *J* = 7.5 Hz, H-23), 3.49 (dd, 1H, *J* = 7.5, 2.9 Hz, H-22), 2.11-1.85 (m, 6H), 1.78-1.37 (m, 9H), 1.24-0.75 (m, 8H), 0.94 (d, 3H, *J* = 6.7 Hz, CH_3), 0.92 (s, 9H, 3×CH_3), 0.90 (d, 3H, *J* = 6.7 Hz, CH_3), 0.89 (d, 3H, *J* = 6.7 Hz, CH_3), 0.87 (s, 3H, CH_3), 0.83 (d, 3H, *J* = 7.0 Hz, CH_3), 0.68 (s, 3H, CH_3), 0.12 (s, 3H, CH_3), 0.10 (s, 3H, CH_3). ^{13}C NMR (125 MHz, $CDCl_3$): 125.67,

124.80, 110.02, 76.09, 73.02, 65.59, 64.09, 56.18, 53.33, 52.21, 48.07, 42.54, 41.21, 41.15, 40.91, 39.75 (2×C), 35.88, 33.34, 30.79, 28.55, 26.14 (3×C), 24.21, 21.44, 21.04, 20.81, 19.88, 18.60, 13.60, 12.91, 11.61, 10.00, -3.57, -4.09. HRMS (APCI+) calculated for $C_{36}H_{65}O_4Si$ ($[M + H]^+$) 589.4652, found 589.4651.

Synthesis of (22*R*,23*R*)-6,6-ethylenedioxy-5α-campest-2-ene-22,23-diol (6)

1M Solution of TBAF (6.5 mL, 6.5 mmol) was added to a solution of compound **5** (700 mg, 1.19 mmol) in THF (30 mL). The reaction mixture was stirred at room temperature for 1 hour. Then, it was diluted with ethyl acetate and washed with saturated solution of NH_4Cl (20 mL). The combined organic layers were washed with water, dried over $MgSO_4$, filtrated and evaporated under reduced pressure. The residue was purified by column chromatography on silica gel (25% ethyl acetate in cyclohexane) to give diol **6** (524 mg, 93%) as white solid. ^1H NMR (500 MHz, $CDCl_3$): δ 5.67 (m, 1H, H-3), 5.55 (m, 1H, H-2), 4.01-3.90 (m, 3H, H_{ketal}), 3.79 (q, 1H, *J* = 6.6 Hz, H_{ketal}), 3.73 (dd, 1H, *J* = 8.4, 2.0 Hz, H-23), 3.57 (dd, 1H, *J* = 8.4, 1.1 Hz, H-22), 2.12-1.91 (m, 5H), 1.81-1.37 (m, 10H), 1.31-0.76 (m, 7H), 0.98 (d, 3H, *J* = 6.7 Hz, CH_3), 0.96 (d, 3H, *J* = 6.7 Hz, CH_3), 0.91 (d, 3H, *J* = 6.7 Hz, CH_3), 0.86 (s, 3H, CH_3), 0.86 (d, 3H, *J* = 7.0 Hz, CH_3), 0.71 (s, 3H, CH_3). ^{13}C NMR (125 MHz, $CDCl_3$): 125.65, 124.78, 110.03, 74.87, 73.42, 65.59, 64.08, 55.87, 53.36, 52.40, 48.03, 42.36, 41.18 (2×C), 39.95, 39.79, 36.79, 35.86, 33.40, 30.75, 27.71, 23.99, 21.40, 20.86 (2×C), 20.77, 13.59, 11.88 (2×C), 10.12. HRMS (ESI+) calculated for $C_{30}H_{51}O_4$ ($[M + H]^+$) 475.3787, found 475.3787.

Synthesis of (22*R*,23*R*)-2α,22,23-trihydroxy-5α-campestan-6-one (3-deoxycastasterone, 7) and (22*R*,23*R*)-3α,22,23-trihydroxy-5α-campestan-6-one (typhasterol, 8)

1M Solution of borane in THF (1 mL, 1.0 mmol) was added dropwise to the solution of olefin **6** (50 mg, 0.1 mmol) in dry THF (2 mL). The reaction mixture was stirred at room temperature for 6 h. Then, the reaction mixture was quenched by water (2 mL) and cooled to 10 °C. Aqueous solution of NaOH (10%, 1 mL) was added followed by dropwise addition of 30% hydrogen peroxide (2 mL). This reaction mixture was stirred at room temperature overnight. It was then poured into a mixture of sodium sulfite (500 mg) in water (5 mL) and acetic acid (0.5 mL) and stirred for additional 30 min. After then, the reaction mixture was diluted with ethyl acetate and extracted twice with water. The organic layer was dried over $MgSO_4$ and evaporated under reduced pressure. A crude mixture of products was dissolved in a mixture of THF and acetone (5:1, 3 mL) and 5% aqueous HCl (1 mL) was added. The reaction mixture was heated under reflux for 3 h. Then it was diluted with ethyl acetate and extracted twice with water. The organic layer was again dried over $MgSO_4$ and evaporated under reduced pressure. The residue was purified by HPLC (75% ethyl acetate in cyclohexane) to give two alcohols, **7** (19 mg, 40%) and **8** (15 mg, 32%), as white solids.

Compound **7** (3-deoxycastasterone): ^1H NMR (500 MHz, $CDCl_3$): δ 3.78 (m, 1H, H-2β), 3.73 (dd, 1H, *J* = 8.4, 1.8 Hz, H-23), 3.56 (dd, 1H, *J* = 8.4, 1.2 Hz, H-22), 2.30 (dd, *J* = 13.0, 4.4 Hz, H-7a), 2.17 (dd, *J* = 12.1, 2.9 Hz, H-5α), 2.09-1.94 (m, 3H), 1.85-1.46 (m, 11H), 1.38-

1.09 (m, 11H), 0.97 (d, 3H, *J* = 6.7 Hz, CH_3), 0.95 (d, 3H, *J* = 6.7 Hz, CH_3), 0.91 (d, 3H, *J* = 6.7 Hz, CH_3), 0.85 (d, 3H, *J* = 7.0 Hz, CH_3), 0.76 (s, 3H, CH_3), 0.69 (s, 3H, CH_3). ^{13}C NMR (125 MHz, $CDCl_3$): 211.91, 74.68, 73.50, 67.06, 57.91, 56.58, 54.09, 52.25, 47.07, 46.63, 43.06, 42.75, 40.00, 39.43, 37.67, 36.76, 34.60, 30.73, 27.61, 23.79, 21.28, 20.86, 20.73, 19.48, 14.17, 11.91, 11.88, 10.09. HRMS (ESI+) calculated for $C_{28}H_{49}O_4$ ($[M + H]^+$) 449.3631, found 449.3627.

Compound **8** (typhasterol): ^1H NMR (500 MHz, $CDCl_3$): δ 4.18 (m, 1H, H-3β), 3.73 (bd, 1H, *J* = 8.3 Hz, H-23), 3.57 (bd, 1H, *J* = 8.3, 1.2 Hz, H-22), 2.74 (t, 1H, *J* = 7.9 Hz, H-5α), 2.31 (dd, *J* = 13.1, 4.6 Hz, H-7a), 2.04-1.94 (m, 3H), 1.84-1.47 (m, 15H), 1.41-1.19 (m, 6H), 1.10 (m, 1H), 0.97 (d, 3H, *J* = 6.7 Hz, CH_3), 0.95 (d, 3H, *J* = 6.7 Hz, CH_3), 0.92 (d, 3H, *J* = 6.7 Hz, CH_3), 0.85 (d, 3H, *J* = 6.9 Hz, CH_3), 0.74 (s, 3H, CH_3), 0.69 (s, 3H, CH_3). ^{13}C NMR (125 MHz, $CDCl_3$): 212.74, 74.72, 73.50, 65.42, 56.68, 53.74, 52.28, 51.65, 46.80, 42.77, 41.55, 40.01, 39.51, 38.02, 36.78, 31.64, 30.74, 28.14, 27.66, 27.64, 23.79, 21.08, 20.87, 20.75, 12.32, 11.92, 11.88, 10.01. HRMS (APCI+) calculated for $C_{28}H_{49}O_4$ ($[M + H]^+$) 449.3631, found 449.3626.

Synthesis of (22*R*,23*R*)-2α,3α-epoxy-6,6-ethylenedioxy-5α-campestane-22,23-diol (9)

3-Chloroperoxybenzoic acid (77%, 375 mg, 1.68 mmol) was added to a solution of olefin **6** (200 mg, 0.42 mmol) in ethyl acetate (10 mL) and the reaction mixture was stirred at room temperature for 4 hours. The reaction was quenched with saturated aqueous solution of Na_2SO_3 (5 mL) and $NaHCO_3$ (5 mL). The organic layer was separated, washed with water, dried over $MgSO_4$, filtrated and evaporated under reduced pressure. The residue was purified by column chromatography on silica gel (25% ethyl acetate in cyclohexane) to give epoxide **9** (186 mg, 90%) as an off-white solid. ^1H NMR (500 MHz, $CDCl_3$): δ 3.99-3.89 (m, 3H, H_{ketal}), 3.79 (m, 1H, H_{ketal}), 3.72 (dd, 1H, *J* = 8.6, 1.5 Hz, H-23), 3.56 (d, 1H, *J* = 8.4 Hz, H-22), 3.25 (m, 1H), 3.10 (dd, 1H, *J* = 5.8, 4.0 Hz), 2.14 (ddd, 1H, *J* = 14.8, 4.1, 1.2 Hz), 2.09-1.96 (m, 2H), 1.97 (dt, 1H, *J* = 12.7, 3.3 Hz), 1.92 (dd, 1H, *J* = 14.8, 6.1 Hz), 1.81 (m, 1H), 1.75 (dd, 1H, *J* = 13.1, 3.4 Hz), 1.68-0.67 (m, 17H), 0.97 (d, 3H, *J* = 6.7 Hz, CH_3), 0.95 (d, 3H, *J* = 6.7 Hz, CH_3), 0.92 (d, 3H, *J* = 6.7 Hz, CH_3), 0.97 (d, 3H, *J* = 6.7 Hz, CH_3), 0.95 (d, 3H, *J* = 6.7 Hz, CH_3), 0.90 (d, 3H, *J* = 6.7 Hz, CH_3), 0.89 (s, 3H, CH_3), 0.85 (d, 3H, *J* = 6.9 Hz, CH_3), 0.69 (s, 3H, CH_3). ^{13}C NMR (125 MHz, $CDCl_3$): 109.78, 74.83, 73.39, 65.47, 64.08, 55.71, 53.06, 52.83, 52.35, 50.46, 43.22, 42.25, 40.91, 39.97, 39.68, 39.59, 36.80, 34.73, 33.10, 30.74, 27.69, 23.99, 20.87, 20.81, 20.77, 20.07, 14.73, 11.89, 11.84, 10.13. HRMS (ESI+) calculated for $C_{30}H_{51}O_5$ ($[M + H]^+$) 491.3736, found 491.3739.

Synthesis of (22*R*,23*R*)-6,6-ethylenedioxy-5α-campestane-3α,22,23-triol (10)

1M Solution of $LiAlH_4$ in diethylether (1 mL, 1.0 mmol) was added dropwise to a solution of compound **9** (130 mg, 0.27 mmol) in dry THF (6 mL). The reaction mixture was stirred at 40 °C for 3 hours and then quenched with MeOH (3 mL). Saturated aqueous solution of sodium potassium tartrate (10 mL) was added and the mixture was diluted with ethyl acetate. The aqueous layer was separated and extracted with ethyl acetate (2×). The combined organic layers were washed with water, dried over $MgSO_4$, filtrated and evaporated under reduced pressure. The residue was purified by column chromatography on silica gel (90% ethyl

acetate in cyclohexane) to give triol **10** (114 mg, 87%) as a viscous oil. ^{1}H NMR (500 MHz, $CDCl_3$): δ 4.13 (m, 1H, H-3β), 4.02-3.92 (m, 3H, H_{ketal}), 3.75-3.71 (m, 2H, H-23, H_{ketal}), 3.55 (d, 1H, *J* = 8.5 Hz, H-22), 2.08-1.93 (m, 3H), 1.79 (dd, 1H, *J* = 13.1, 3.7 Hz), 1.70-1.05 (m, 22H), 0.97 (d, 3H, *J* = 6.7 Hz, CH_3), 0.95 (d, 3H, *J* = 6.7 Hz, CH_3), 0.93 (s, 3H, CH_3), 0.90 (d, 3H, *J* = 6.7 Hz, CH_3), 0.85 (d, 3H, *J* = 6.9 Hz, CH_3), 0.71 (s, 3H, CH_3). ^{13}C NMR (125 MHz, $CDCl_3$): 110.59, 75.44, 72.53, 65.68, 65.44, 64.05, 56.22, 53.85, 52.66, 45.05, 42.56, 40.64, 40.09, 39.60, 37.66, 37.17, 33.82, 33.35, 30.87, 27.86, 27.51, 27.18, 24.11, 20.92, 20.87, 20.66, 13.04, 11.93, 11.87, 10.28. HRMS (ESI+) calculated for $C_{30}H_{53}O_5$ ($[M + H]^+$) 493.3893, found 493.3895.

Synthesis of typhasterol (8) from compound 10

Aqueous solution of HCl (5%, 0.5 mL) was added to a solution of compound **10** (80 mg, 0.16 mmol) in THF (5 mL) and the reaction mixture was stirred at 40 °C for 4 hours and then diluted with ethyl acetate. Organic layer was washed twice with water, dried over $MgSO_4$, filtrated and evaporated under reduced pressure. The residue was purified by column chromatography on silica gel (90% ethyl acetate in cyclohexane) to give typhasterol (**8**) (75 mg, 90%) as a white solid. The spectroscopic data were entirely consistent with those reported for typhasterol above.

Synthesis of (22*R*,23*R*)-3α,22,23-trihydroxy-7a-homo-7-oxa-5α-campestan-6-one (2-deoxybrassinolide, 11)

Freshly prepared trifluoroperoxyacetic acid (4 mL) was added to a solution of typhasterol (**8**) (60 mg, 0.13 mmol) in dichloromethane (10 mL) and the reaction mixture was stirred at room temperature for 4 hours and quenched with saturated aqueous solutions of Na_2SO_3 (5 mL) and $NaHCO_3$ (5 mL). The aqueous layer was separated and extracted twice with dichloromethane. The combined organic layers were washed with water, dried over $MgSO_4$, filtrated and evaporated under reduced pressure to give inseparable crude mixture of isomeric lactones (65 mg) with compound **11** as the major isomer (ratio approx. 3:1). For separation the mixture had to be acetylated. Crude lactones were dissolved in pyridine (6 mL) and acetic anhydride (1 mL) was added. The reaction mixture was stirred at 50 °C for 12 hours. Then, ethanol (5 mL) and ethyl acetate (10 mL) were added and the mixture was stirred for additional 30 min. The mixture was washed twice with water, dried over $MgSO_4$, filtrated and evaporated under reduced pressure. The residue was purified by HPLC (30% ethyl acetate in cyclohexane) to give two triacetates, **12** (50 mg, 63%) and **12a** (15 mg, 19%), as a viscous oil.
Compound **12** (40 mg, mmol) was dissolved in THF (5 mL) and 5M methanolic solution of sodium methoxide (0.1 mL) was added. The reaction mixture was stirred at room temperature for 1 hour and quenched with 5% aqueous solution of HCl (1 mL). The mixture was diluted with ethyl acetate, washed twice with water, dried over $MgSO_4$, filtrated and evaporated under reduced pressure. The residue was purified by HPLC (ethyl acetate) to give lactone **11** (28 mg, 89%) as a white solid.

Compound **11** (2-deoxybrassinolide): ^{1}H NMR (500 MHz, $CDCl_3$): δ 4.17 (m, 1H, H-3β), 4.11-4.08 (m, 2H, H-7aα, H-7aβ), 3.73 (dd, 1H, *J* = 8.4, 1.2 Hz, H-23), 3.55 (d, 1H, *J* = 8.4 Hz, H-22), 3.19 (dd, 1H, *J* = 12.2, 4.6 Hz, H-5α), 2.14 (ddd, 1H, *J* = 14.9, 12.5, 2.3 Hz), 2.02-1.96 (m, 3H), 1.81-1.19 (m, 19H), 0.90 (m, 1H), 0.97 (d, 3H, *J* = 6.7 Hz, CH_3), 0.95 (d, 3H, *J* = 6.7 Hz, CH_3), 0.91 (d, 3H, *J* = 6.7 Hz, CH_3), 0.90 (s, 3H, CH_3), 0.85 (d, 3H, *J* = 6.9 Hz, CH_3), 0.72 (s, 3H, CH_3). ^{13}C NMR (125 MHz, $CDCl_3$): 176.78, 74.59, 73.53, 70.39, 64.88, 58.29, 52.25, 51.37, 42.44, 41.75, 40.02, 39.50, 36.79, 36.28, 32.45, 30.73, 29.69, 28.23, 27.57, 24.71, 22.15, 20.85, 20.74, 14.55, 14.12, 11.82, 11.72, 10.10. HRMS (APCI+) calculated for $C_{28}H_{49}O_5$ ($[M + H]^+$) 465.3580, found 465.3574.

Compound **12** ((22*R*,23*R*)-7a-homo-7-oxa-6-oxo-5α-campestan-3α,22,23-triyl triacetate): ^{1}H NMR (500 MHz, $CDCl_3$): δ 5.33 (dd, 1H, *J* = 8.9, 1.2 Hz, H-23), 5.15 (d, 1H, *J* = 8.9 Hz, H-22), 5.09 (m, 1H, H-3β), 4.12-4.03 (m, 2H, H-7aα, H-7aβ), 3.02 (dd, 1H, *J* = 12.2, 4.3 Hz, H-5α), 2.17 (ddd, 1H, *J* = 15.0, 12.5, 2.5 Hz), 2.10-1.96 (m, 3H), 2.07, 2.01, 2.00 (all s, 1H, CH_3), 1.85-1.13 (m, 17H), 1.01 (d, 3H, *J* = 6.7 Hz, CH_3), 0.96 (d, 3H, *J* = 6.7 Hz, CH_3), 0.94 (d, 3H, *J* = 6.7 Hz, CH_3), 0.91 (d, 3H, *J* = 6.7 Hz, CH_3), 0.90 (s, 3H, CH_3), 0.73 (s, 3H, CH_3). ^{13}C NMR (125 MHz, $CDCl_3$): 176.04, 170.56, 170.51, 170.30, 75.67, 74.05, 70.29, 68.41, 58.37, 52.29, 51.42, 42.55, 42.46, 39.78, 39.56, 39.43, 36.97, 36.11, 33.67, 30.34, 29.73, 27.98, 25.14, 24.69, 22.12, 21.37, 20.93, 20.86 (2×C), 20.31, 14.61, 12.71, 11.59, 11.05. HRMS (APCI+) calculated for $C_{34}H_{55}O_8$ ($[M + H]^+$) 591.3897, found 591.3896.

Compound **12a** ((22*R*,23*R*)-7a-homo-6-oxa-7-oxo-5α-campestan-3α,22,23-triyl triacetate): ^{1}H NMR (500 MHz, $CDCl_3$): δ 5.33 (dd, 1H, *J* = 8.9, 1.5 Hz, H-23), 5.15 (d, 1H, *J* = 8.9 Hz, H-22), 5.14 (m, 1H, H-3β), 4.46 (dd, 1H, *J* = 11.2, 5.5 Hz, H-5α), 2.53 (d, 1H, *J* = 13.3 Hz, H-7aβ), 2.45 (dd, 1H, *J* = 13.3, 11.3 Hz, H-7aα), 2.12-1.95 (m, 3H), 2.07, 2.01, 2.00 (all s, 1H, CH_3), 1.90-1.09 (m, 17H), 1.01 (d, 3H, *J* = 6.7 Hz, CH_3), 0.96 (d, 3H, *J* = 6.7 Hz, CH_3), 0.95 (d, 3H, *J* = 6.7 Hz, CH_3), 0.914 (s, 3H, CH_3), 0.908 (d, 3H, *J* = 6.7 Hz, CH_3), 0.73 (s, 3H, CH_3). ^{13}C NMR (125 MHz, $CDCl_3$): 174.74, 170.53 (2×C), 170.16, 79.67, 75.61, 74.03, 69.56, 58.02, 55.45, 52.62, 42.52, 39.79, 39.64, 39.58, 38.03, 36.97, 34.80, 32.93, 31.84, 30.36, 27.65, 25.14, 24.83, 22.18, 21.31, 20.94, 20.86 (2×C), 20.31, 12.70, 11.64, 11.59, 11.06. HRMS (APCI+) calculated for $C_{34}H_{55}O_8$ ($[M + H]^+$) 591.3897, found 591.3888.

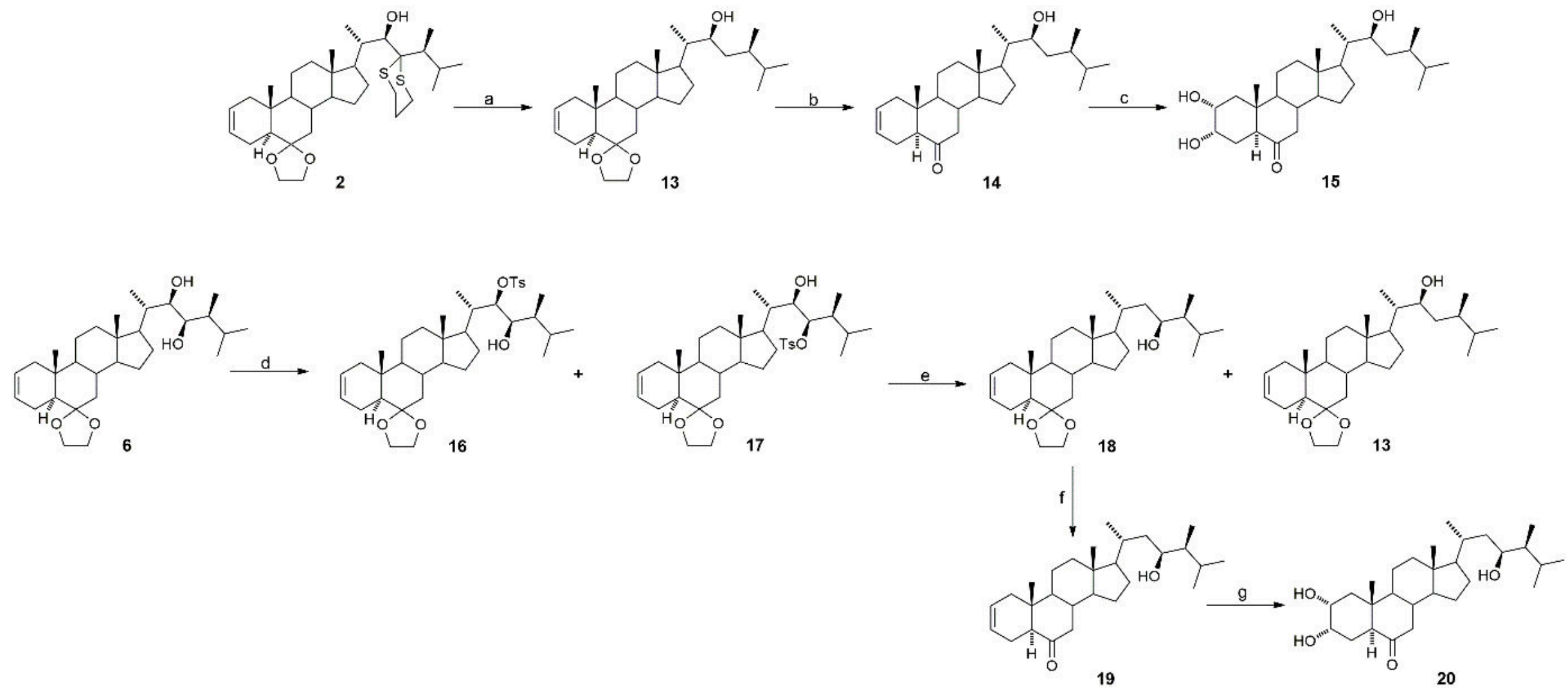

Scheme 2: Synthesis of 23-deoxycastasterone (**15**), 22-deoxycastaterone (**20**). **a**: Ra-Ni/EtOH, r.t., 6h; **b**: HCl/H_2O/THF, 40 °C, 4h; **c**: OsO_4/K_2CO_3/$K_2[Fe(CN)_6]$/$CH_3SO_2NH_2$/HQ-4-ClBz/t-BuOH/H_2O, r.t., 10 h; **d**: *p*-TsCl/pyridine, r.t., 3 days; **e**: $LiAlH_4$/THF/Et_2O, r.t., 3h; **f**: HCl/H_2O/THF, 40 °C, 2h; **g**: OsO_4/K_2CO_3/$K_2[Fe(CN)_6]$/$CH_3SO_2NH_2$/HQ-4-ClBz/t-BuOH/H_2O, r.t., 10 h.

Synthesis of (22*S*)-6,6-ethylenedioxy-5α-campest-2-en-22-ol (13)

Raney-Nickel catalyst 2400 (Merck, 1.4g) was added to a solution of compound **2** (200 mg, mmol) in ethanol (20 mL). The resulting suspension was shaken vigorously for 6 hours and then the liquid was decanted. Catalyst was washed with ethanol (2× 10 mL) and the combined ethanolic fractions were evaporated under reduced pressure. The residue was purified by column chromatography on silica gel (5-10% ethyl acetate in cyclohexane) to give alcohol **13** (85 mg, 52%) as a viscous oil. ^{1}H NMR (500 MHz, $CDCl_3$): δ 5.67 (m, 1H, H-3), 5.55 (m, 1H, H-2), 4.01-3.89 (m, 3H, H_{ketal}), 3.81-3.75 (m, 2H, H-22, H_{ketal}), 2.11-1.90 (m, 4H), 1.81-1.70 (m, 3H), 1.63-0.76 (m, 18H), 0.895 (d, 3H, J = 6.7 Hz, CH_3), 0.889 (s, 3H, CH_3), 0.880 (d, 3H, J = 6.7 Hz, CH_3), 0.84 (d, 3H, J = 6.7 Hz, CH_3), 0.82 (d, 3H, J = 6.7 Hz, CH_3) 0.71 (s, 3H, CH_3). ^{13}C NMR (125 MHz, $CDCl_3$): 125.67, 124.80, 110.05, 71.66, 65.59, 64.08, 55.89, 53.41, 52.58, 48.05, 42.46, 41.21 (2×C), 39.79, 39.35, 39.29, 35.87, 35.29, 33.40, 32.00, 27.71, 24.08, 21.42, 20.87, 19.98, 17.80, 15.76, 13.60, 11.92, 11.22. HRMS (ESI+) calculated for $C_{30}H_{51}O_3$ ($[M + H]^+$) 459.3838, found 459.3834.

Synthesis of (22*S*)-22-hydroxy-5α-campest-2-en-6-one (14)

Aqueous solution of HCl (5%, 0.5 mL) was added to a solution of compound **13** (70 mg, 0.15 mmol) in THF (5 mL) and the reaction mixture was stirred at 40 °C for 4 hours and then diluted with ethyl acetate. Organic layer was washed twice with water, dried over $MgSO_4$, filtrated and evaporated under reduced pressure. The residue was purified by column chromatography on silica gel (10% ethyl acetate in cyclohexane) to give compound **14** (55 mg, 87%) as a white solid. ^{1}H NMR (500 MHz, $CDCl_3$): δ 5.68 (m, 1H, H-3), 5.56 (m, 1H, H-

2), 3.67 (td, *J* = 6.8, 1.1 Hz, H-22), 2.37-2.34 (m, 2H), 2.25 (m, 1H), 2.06-1.92 (m, 4H), 1.74 (m, 1H), 1.62-0.96 (m, 17H), 0.89 (d, 3H, *J* = 6.7 Hz, CH_3), 0.87 (d, 3H, *J* = 6.7 Hz, CH_3), 0.82 (d, 3H, *J* = 6.7 Hz, CH_3), 0.80 (d, 3H, *J* = 7.0 Hz, CH_3), 0.71 (s, 3H, CH_3), 0.68 (s, 3H, CH_3). ^{13}C NMR (125 MHz, $CDCl_3$): 212.06, 124.87, 124.46, 71.48, 56.59, 53.74, 53.27, 52.39, 46.92, 42.66, 39.99, 39.41, 39.31, 39.26, 39.22, 37.70, 35.22, 31.96, 27.57, 23.83, 21.64, 21.07, 19.92, 17.77, 15.71, 13.45, 11.82, 11.16. HRMS (ESI+) calculated for $C_{28}H_{47}O_2$ ($[M + H]^+$) 415.3576, found 415.3578.

Synthesis of (22*S*)-2α,3α,22-trihydroxy-5α-campestan-6-one (23-deoxycastasterone, 15)

Solution of osmium tetroxide in *t*-butanol (0.2M, 0.15 mL, 0.03 mmol) was added to a solution of compound **14** (40 mg, 0.1 mmol), hydroquinidine 4-chlorobenzoate (25 mg; 0.05 mmol), methanesulfonamide (5 mg; 0.05 mmol), potassium carbonate (100 mg; 0.72 mmol), and potassium ferricyanide (240 mg; 0.73 mmol) in the mixture of *t*-butanol and water (5 mL; 1 : 1 v/v). The reaction mixture was stirred at room temperature for 10 h and quenched with saturated solution of Na_2SO_3 (3 mL) was then added. After an additional 30 minutes of stirring, the reaction mixture was diluted with ethyl acetate (30 mL) and extracted with water (2 × 20 mL). Organic layer was dried over $MgSO_4$, filtrated and evaporated under reduced pressure. The residue was purified by column chromatography on silica gel (0.5% isopropanol in ethyl acetate) to give triol **15** (31 mg, 72%) as a white solid. ^1H NMR (500 MHz, $CDCl_3$ + 5% CD_3OD): 3.95 (m, 1H, H-3β), 3.73 (m, 1H, H-2β), 3.71 (bt, 1H, *J* = 6.4 Hz, H-22), 2.65 (dd, 1H, *J* = 12.4, 2.3 Hz, H-5α), 2.25 (dd, 1H, *J* = 13.0, 4.6 Hz, H-7β), 2.01-1.21 (m, 21H), 1.06 (m, 1H), 0.85 (d, 3H, *J* = 6.7 Hz, CH_3), 0.83 (d, 3H, *J* = 6.7 Hz, CH_3), 0.78 (d, 3H, *J* = 7.0 Hz, CH_3), 0.77 (d, 3H, *J* = 7.0 Hz, CH_3), 0.71 (s, 3H, CH_3), 0.64 (s, 3H, CH_3). ^{13}C NMR (125 MHz, $CDCl_3$ + 5% CD_3OD): 213.04, 71.30, 67.97, 67.88, 56.48, 53.57, 52.27, 50.71, 46.62, 42.75, 42.54, 39.84, 39.31, 39.13, 39.08, 37.70, 35.14, 31.90, 29.57, 27.50, 23.77, 21.09, 19.84, 17.68, 15.59, 13.42, 11.81, 11.08. HRMS (ESI+) calculated for $C_{28}H_{49}O_4$ ($[M + H]^+$) 449.3631, found 449.3633.

Synthesis of (22*R*,23*R*)-6,6-ethylenedioxy-23-hydroxy-5α-campest-2-en-22-yl *p*-toluenesulfonate (16) and (22*R*,23*R*)-6,6-ethylenedioxy-22-hydroxy-5α-campest-2-en-23-yl *p*-toluenesulfonate (17)

p-Toluenesulfonyl chloride (200 mg, 1.05 mmol) was added to a solution of compound **6** (100 mg; 0.21 mmol) in pyridine (4 mL). The reaction mixture was stirred at room temperature for 3 days. Then, the mixture was diluted with ethyl acetate and aqueous solution of HCl (5%, 5 mL) was added. The aqueous layer was separated and extracted with ethyl acetate (2×). The combined organic layers were washed with water, dried over $MgSO_4$, filtrated and evaporated under reduced pressure. The residue was purified by column chromatography on silica gel (10% ethyl acetate in cyclohexane). This chromatography did not lead to separation of both tosylates and they were used in the next reaction step as a mixture of isomers. Yield of both isomers 91 mg (69%). Analytical sample was used for HPLC separation (8% ethyl acetate in cyclohexane) to give two tosylates, **16** and **17,** as a viscous oil.

Compound **16**: ^{1}H NMR (500 MHz, $CDCl_3$): 7.85 (d, 2H, J = 8.4 Hz, H_{Ts}), 7.34 (d, 2H, J = 8.4 Hz, H_{Ts}), 5.67 (m, 1H, H-3), 5.55 (m, 1H, H-2), 4.77 (d, 1H, J = 8.6 Hz, H-22), 4.01-3.91 (m, 4H, H-23, H_{ketal}),3.80 (m, 1H, H_{ketal}), 2.46 (s, 3H, CH_3), 2.31 (d, 1H, J = 4.9 Hz, OH), 2.13-0.68 (m, 22H), 0.95 (d, 3H, J = 6.7 Hz, CH_3), 0.94 (d, 3H, J = 6.7 Hz, CH_3), 0.90 (d, 3H, J = 7.0 Hz, CH_3), 0.88 (d, 3H, J = 7.0 Hz, CH_3), 0.87 (s, 3H, CH_3), 0.66 (s, 3H, CH_3). ^{13}C NMR (125 MHz, $CDCl_3$): 144.50, 134.67, 129.61 (2×C), 127.62 (2×C), 125.72, 124.69, 109.92, 91.04, 71.41, 65.60, 64.12, 55.87, 53.52, 51.87, 48.08, 42.24, 41.26, 41.19, 40.88, 39.66, 37.72, 35.84, 33.27, 30.59, 27.95, 23.95, 21.70, 21.41, 20.84, 20.76, 20.70, 13.61, 12.57, 11.61, 10.17. HRMS (APCI+) calculated for $C_{37}H_{57}O_6S$ ($[M + H]^+$) 629.3876, found 629.3865.

Compound **17**: ^{1}H NMR (500 MHz, $CDCl_3$): 7.83 (d, 2H, J = 8.3 Hz, H_{Ts}), 7.34 (d, 2H, J = 8.3 Hz, H_{Ts}), 5.66 (m, 1H, H-3), 5.55 (m, 1H, H-2), 5.04 (d, 1H, J = 8.6 Hz, H-22), 4.00-3.89 (m, 3H, H_{ketal}), 3.81-3.75 (m, 2H, H-22, H_{ketal}), 2.45 (s, 4H, OH, CH_3), 2.13-0.76 (m, 22H), 1.00 (d, 3H, J = 6.7 Hz, CH_3), 0.94 (d, 3H, J = 6.7 Hz, CH_3), 0.888 (s, 3H, CH_3), 0.8875 (d, 3H, J = 7.0 Hz, CH_3), 0.84 (d, 3H, J = 7.0 Hz, CH_3), 0.70 (s, 3H, CH_3). ^{13}C NMR (125 MHz, $CDCl_3$): 144.50, 134.44, 129.65 (2×C), 127.58 (2×C), 125.64, 124.81, 110.03, 89.23, 72.97, 65.59, 64.09, 55.79, 53.32, 52.06, 48.03, 42.29, 41.18, 41.16, 40.57, 39.73, 37.32, 35.86, 33.41, 29.76, 27.65, 23.95, 21.65, 21.42, 21.14, 20.86, 20.66, 13.60, 11.96, 11.77, 11.15. HRMS (APCI+) calculated for $C_{37}H_{57}O_6S$ ($[M + H]^+$) 629.3876, found 629.3868.

Synthesis of (23*S*)-6,6-ethylenedioxy-5α-campest-2-en-23-ol (18) and (22*S*)-6,6-ethylenedioxy-5α-campest-2-en-22-ol (13)

1M Solution of $LiAlH_4$ in diethylether (1 mL, 1.0 mmol) was added dropwise to a solution of compounds **16** and **17** (50 mg, 0.08 mmol) in dry THF (5 mL). The reaction mixture was stirred at room temperature for 3 hours and then quenched with MeOH (3 mL). Saturated aqueous solution of sodium potassium tartrate (10 mL) was added and the mixture was diluted with ethyl acetate. The aqueous layer was separated and extracted with ethyl acetate (2×). The combined organic layers were washed with water, dried over $MgSO_4$, filtrated and evaporated under reduced pressure. The residue was purified by column chromatography on silica gel (5-10% ethyl acetate in cyclohexane) to give crude mixture of compounds **13** and **18** (32 mg). Both compounds were separated by HPLC (8% ethyl acetate in cyclohexane) to afford two alcohols **13** (11 mg, 31%) and **18** (14 mg, 39%) as a viscous oil. The spectroscopic data for compound **13** were entirely consistent with those reported above.

Compound **18**: ^{1}H NMR (500 MHz, $CDCl_3$): 5.67 (m, 1H, H-3), 5.55 (m, 1H, H-2), 4.01-3.88 (m, 4H, H-23, H_{ketal}), 3.79 (q, 1H, J = 6.6 Hz, H_{ketal}), 2.12-1.86 (m, 5H), 1.80-0.75 (m, 20H), 0.98 (d, 3H, J = 6.7 Hz, CH_3), 0.96 (d, 3H, J = 6.7 Hz, CH_3), 0.93 (d, 3H, J = 6.7 Hz, CH_3), 0.89 (s, 3H, CH_3), 0.84 (d, 3H, J = 7.0 Hz, CH_3), 0.70 (s, 3H, CH_3). ^{13}C NMR (125 MHz, $CDCl_3$): 125.68, 124.81, 110.05, 70.98, 65.58, 64.09, 57.00, 55.93, 53.42, 48.07, 42.61, 42.45, 42.06, 41.22, 41.20, 39.80, 35.88, 33.97, 33.33, 30.43, 28.40, 24.13, 21.43, 21.11, 20.85, 20.49, 19.21, 13.60, 11.97, 9.27. HRMS (APCI+) calculated for $C_{30}H_{51}O_3$ ($[M + H]^+$) 459.3838, found 459.3831.

Synthesis of (23*S*)-23-hydroxy-5α-campest-2-en-6-one (19)

Aqueous solution of HCl (5%, 0.5 mL) was added to a solution of compound **18** (12 mg, 0.03 mmol) in THF (3 mL) and the reaction mixture was stirred at 40 °C for 2 hours and then diluted with ethyl acetate. Organic layer was washed twice with water, dried over $MgSO_4$, filtrated and evaporated under reduced pressure. The residue was purified by column chromatography on silica gel (10% ethyl acetate in cyclohexane) to give compound **19** (9 mg, 83%) as a viscous oil. ^{1}H NMR (500 MHz, $CDCl_3$): 5.69 (m, 1H, H-3), 5.57 (m, 1H, H-2), 3.90 (ddd, 1H, *J* = 7.9, 5.7, 2.6 Hz, H-23), 2.38-2.34 (m, 2H), 2.27 (m, 1H), 2.09-1.89 (m, 5H), 1.75 (m, 1H), 1.66-1.04 (m, 16H), 0.99 (d, 3H, *J* = 6.7 Hz, CH_3), 0.96 (d, 3H, *J* = 6.7 Hz, CH_3), 0.93 (d, 3H, *J* = 6.7 Hz, CH_3), 0.84 (d, 3H, *J* = 7.0 Hz, CH_3), 0.72 (s, 3H, CH_3), 0.69 (s, 3H, CH_3). ^{13}C NMR (125 MHz, $CDCl_3$): 212.09, 124.94, 124.51, 70.97, 58.87, 58.67, 53.81, 53.34, 46.97, 42.87, 42.49, 41.98, 40.04, 39.50, 39.32, 37.67, 33.93, 30.41, 28.31, 23.93, 21.70, 21.11, 21.09, 20.48, 19.18, 13.50, 11.91, 9.26. HRMS (APCI+) calculated for $C_{28}H_{47}O_2$ ($[M + H]^+$) 415.3576, found 415.3569.

Synthesis of (23*S*)-2α,3α,23-trihydroxy-5α-campestan-6-one (22-deoxycastasterone, 20)

Solution of osmium tetroxide in *t*-butanol (0.2M, 0.05 mL, 0.01 mmol) was added to a solution of compound **19** (8 mg, 0.02 mmol), hydroquinidine 4-chlorobenzoate (10 mg; 0.02 mmol), methanesulfonamide (3 mg; 0.03 mmol), potassium carbonate (20 mg; 0.14 mmol), and potassium ferricyanide (45 mg; 0.14 mmol) in the mixture of *t*-butanol and water (3 mL; 1 : 1 v/v). The reaction mixture was stirred at room temperature for 10 h and quenched with saturated solution of Na_2SO_3 (2 mL) was then added. After an additional 30 minutes of stirring, the reaction mixture was diluted with ethyl acetate (20 mL) and extracted with water (2 × 20 mL). Organic layer was dried over $MgSO_4$, filtrated and evaporated under reduced pressure. The residue was purified by HPLC (70% ethyl acetate in cyclohexane) to give triol **20** (6 mg, 69%) as a white solid (lyophilized from *t*-butanol). ^{1}H NMR (500 MHz, $CDCl_3$ + 10% CD_3OD): 4.03 (m, 1H, H-3β), 3.89 (ddd, 1H, *J* = 8.0, 5.7, 2.6 Hz, H-23), 3.75 (m, 1H, H-2β), 2.68 (dd, 1H, *J* = 12.2, 3.4 Hz, H-5α), 2.29 (dd, 1H, *J* = 13.1, 4.6 Hz, H-7β), 2.07-1.05 (m, 22H), 0.97 (d, 3H, *J* = 6.7 Hz, CH_3), 0.95 (d, 3H, *J* = 6.7 Hz, CH_3), 0.92 (d, 3H, *J* = 6.7 Hz, CH_3), 0.83 (d, 3H, *J* = 6.7 Hz, CH_3), 0.75 (s, 3H, CH_3), 0.67 (s, 3H, CH_3). ^{13}C NMR (125 MHz, $CDCl_3$ + 10% CD_3OD): 212.38, 70.94, 68.33, 68.20, 56.79, 56.57, 53.62, 50.68, 46.72, 42.98, 42.58, 42.43, 41.89, 40.12, 39.37, 37.65, 33.90, 30.38, 28.29, 26.25, 23.90, 21.14, 21.09, 20.47, 19.11, 13.52, 11.96, 9.24. HRMS (APCI+) calculated for $C_{28}H_{49}O_4$ ($[M + H]^+$) 449.3631, found 449.3629.

References

1. Hurski, A. L., Ermolovich, Y. V., Zhabinskii, V. N. & Khripach, V. A. The development and use of a general route to brassinolide, its biosynthetic precursors, metabolites and analogues. *Org. Biomol. Chem*. **13**, 1446-1452 (2015).
2. Eignerova, B., Slavikova, B., Budesinsky, M., Dracinsky, M., Klepetarova, B., Stastna, E., Kotora, M. Synthesis of Fluorinated Brassinosteroids Based on Alkene

Cross-Metathesis and Preliminary Biological Assessment. *J. Med. Chem*. **52**, 5753-5757 (2009).

^{1}H and ^{13}C NMR spectra of compounds prepared

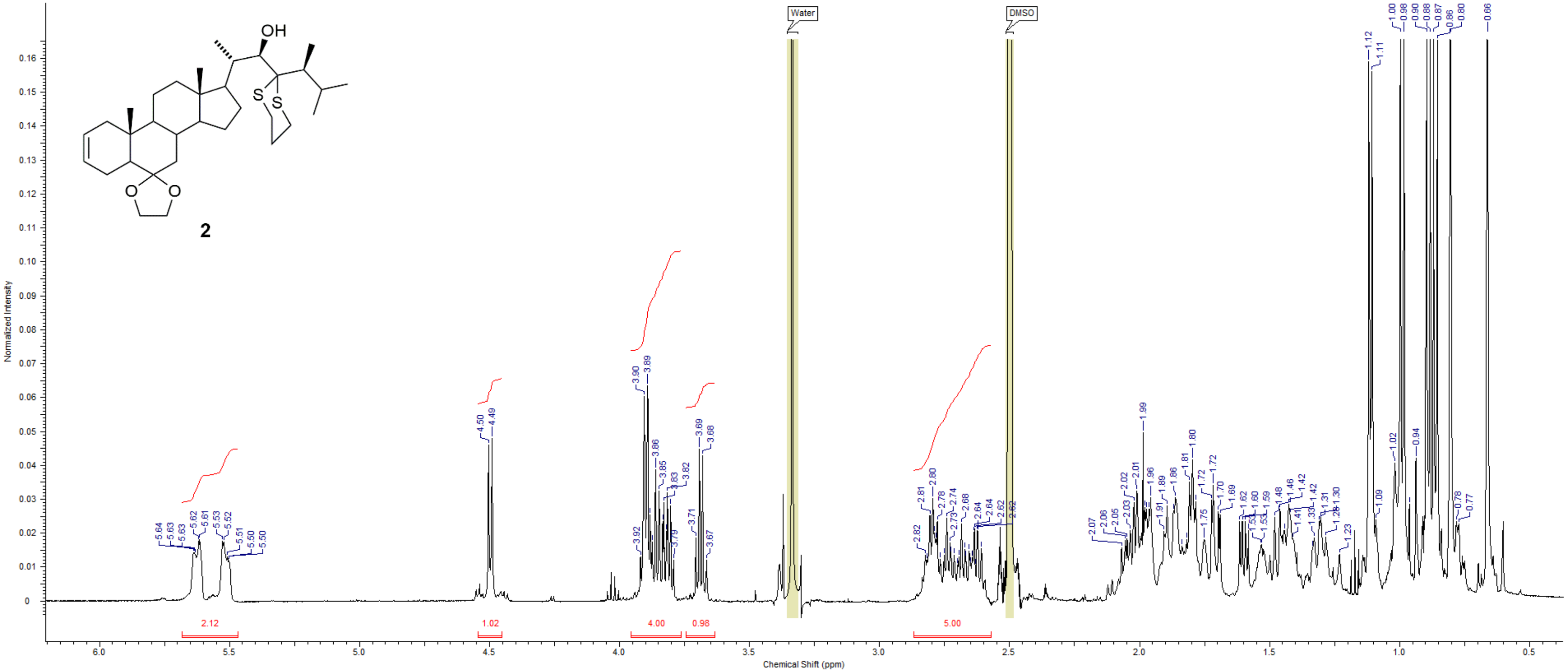

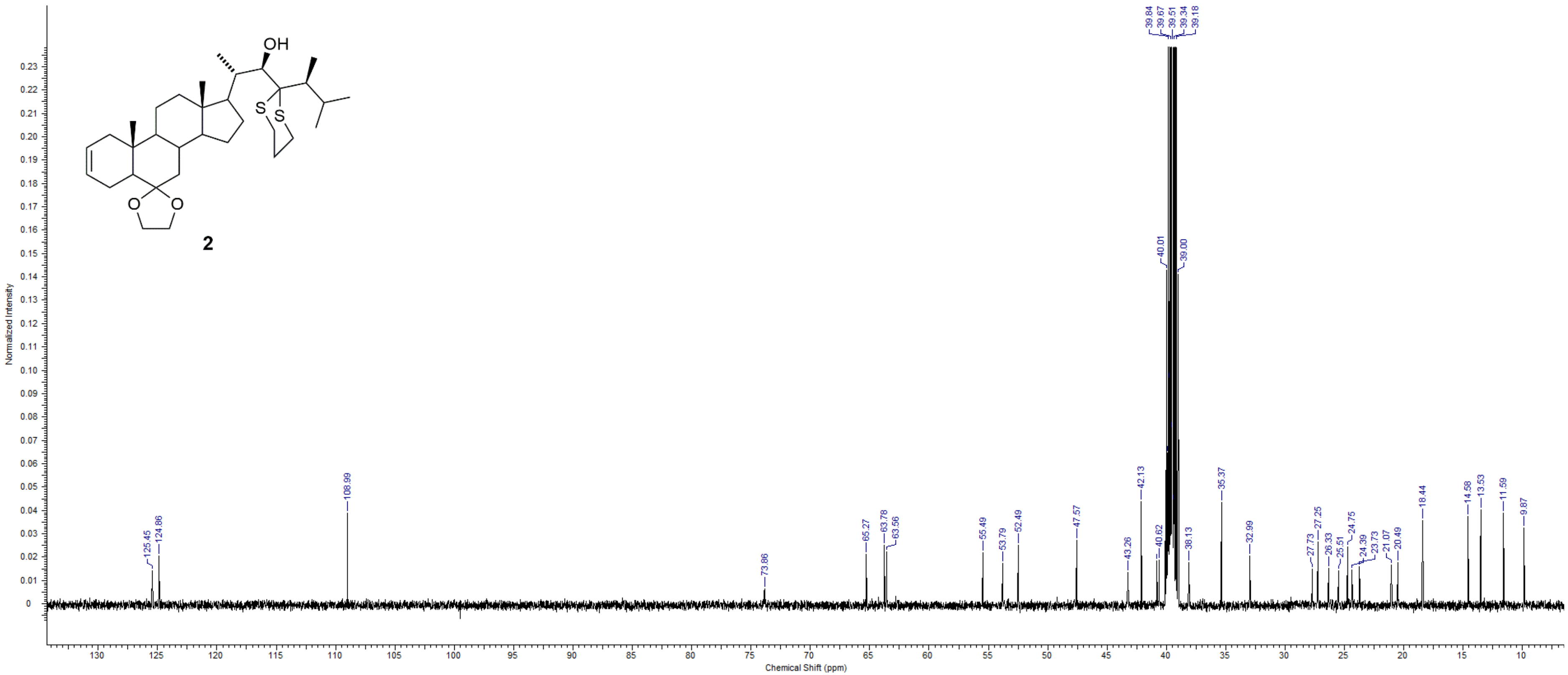

OH
S
S
O
O
2
Normalized Intensity
Chemical Shift (ppm)
125.45
124.86
108.99
73.86
65.27
63.78
63.56
55.49
53.79
52.49
47.57
43.26
42.13
40.62
40.01
39.84
39.67
39.51
39.34
39.18
39.00
38.13
35.37
32.99
27.73
27.25
26.33
25.51
24.75
24.39
23.73
21.07
20.49
18.44
14.58
13.53
11.59
9.87

Single Mass Analysis
Tolerance = 1.0 PPM / DBE: min = -1.5, max = 50.0
Element prediction: Off
Number of isotope peaks used for i-FIT = 3
Monoisotopic Mass, Even Electron Ions
2131 formula(e) evaluated with 1 results within limits (up to 50 best isotopic matches for each mass)
Elements Used:

Mass	Calc. Mass	mDa	PPM	DBE	Formula	i-FIT	i-FIT Norm	Fit Conf %	C	H	N	O	S
563.3597	563.3593	0.4	0.7	6.5	C33 H55 O3 S2	92.0	n/a	n/a	33	55		3	2

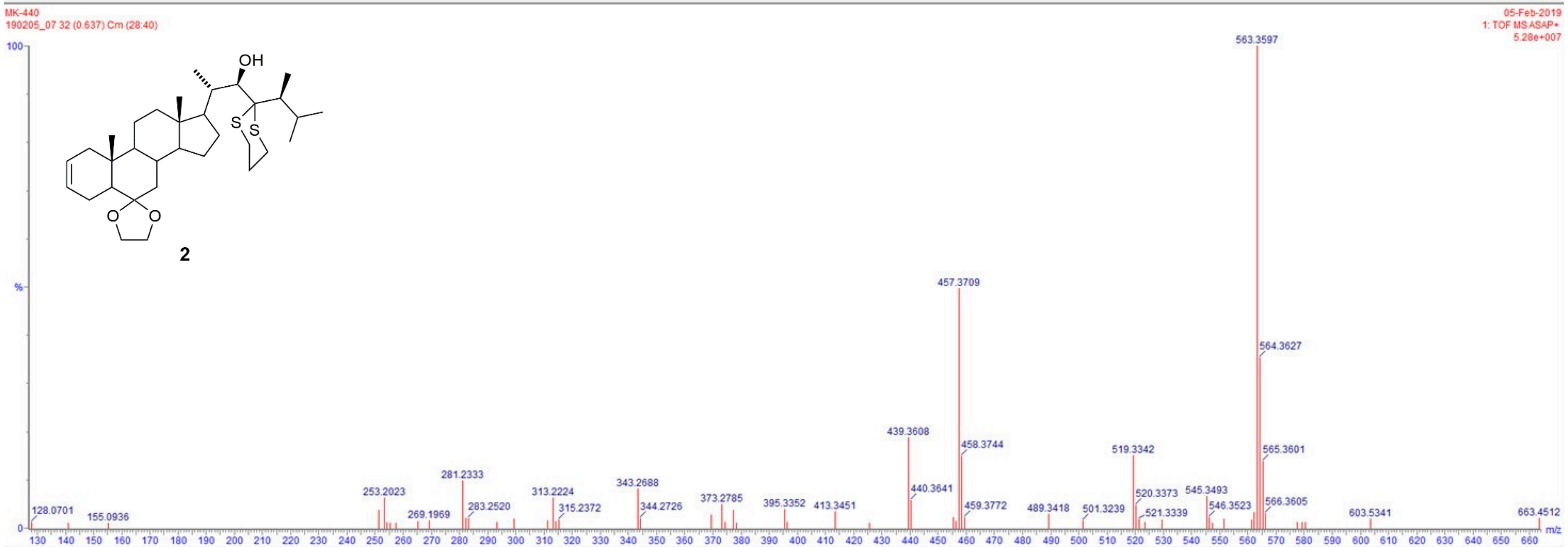

For Help, press F1

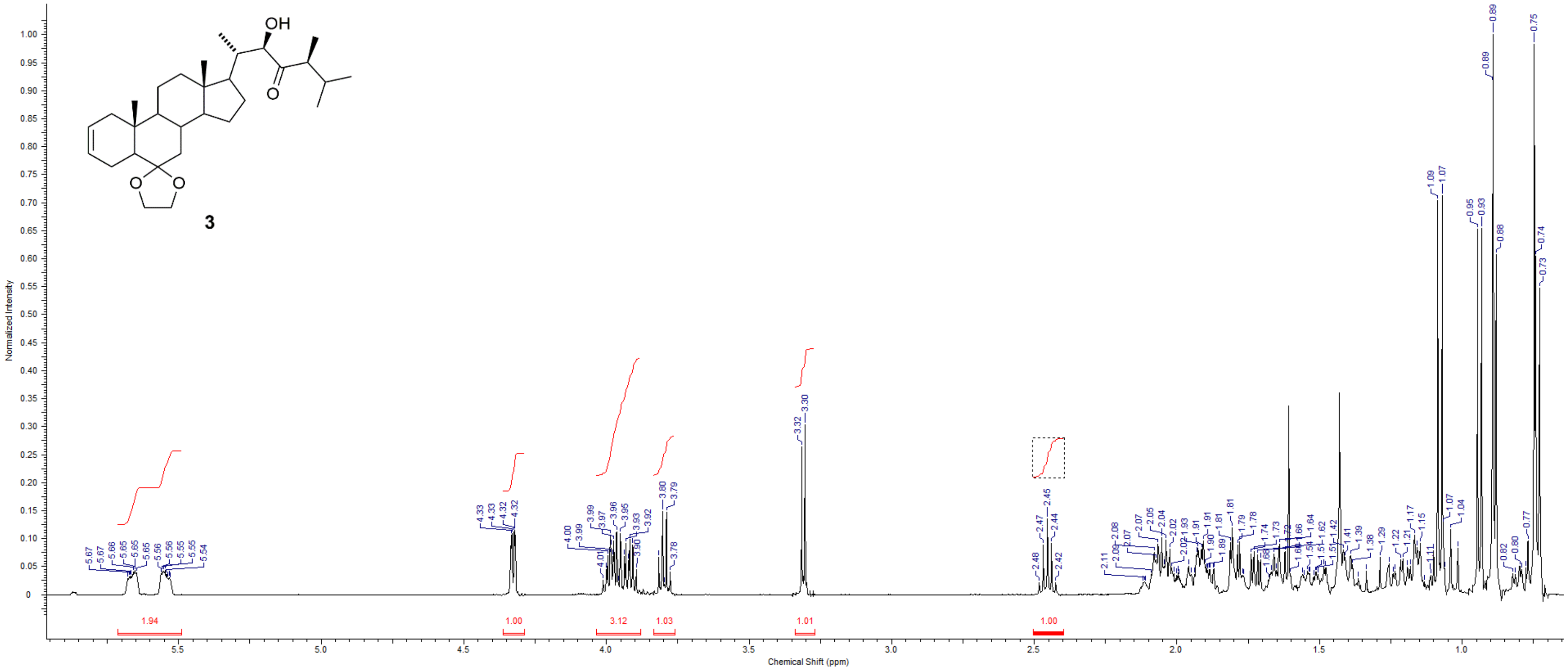

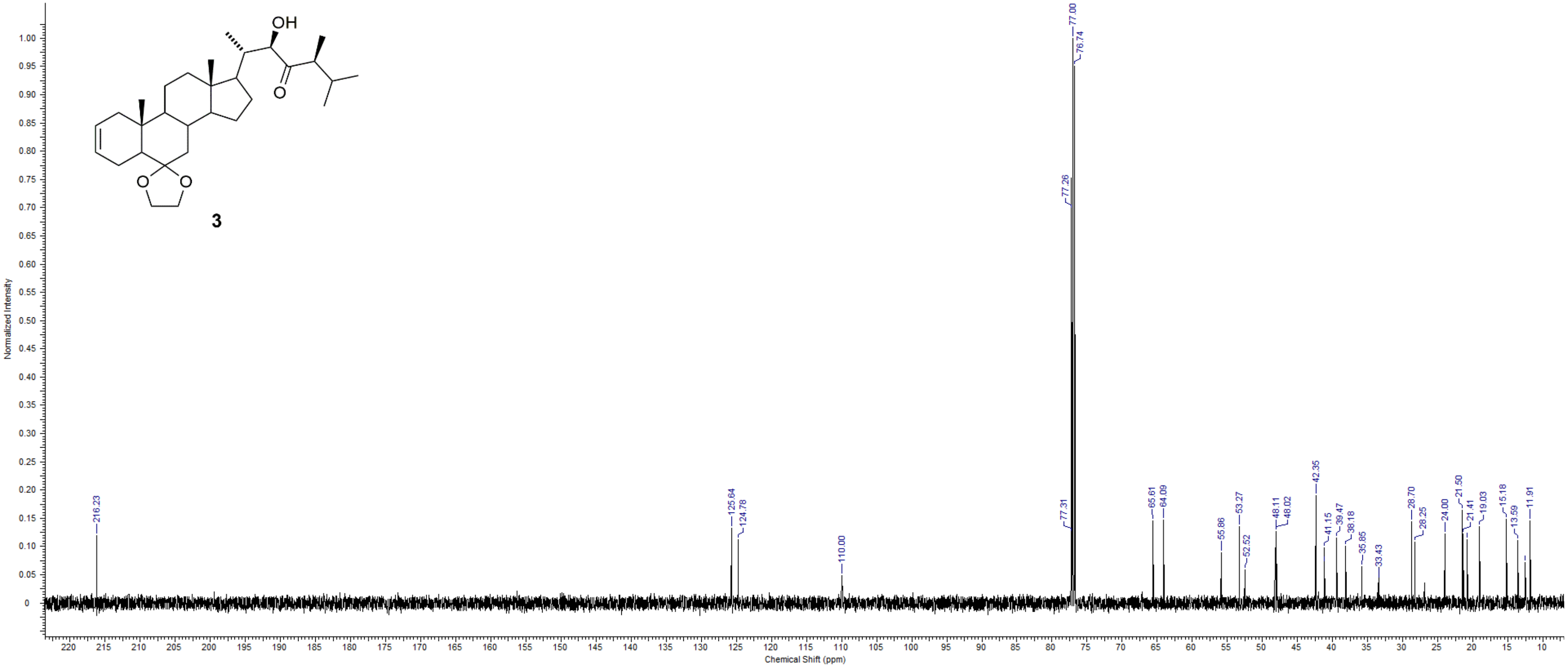
OH
O
O
O
3
Normalized Intensity
Chemical Shift (ppm)
216.23
125.64
124.78
110.00
77.31
77.26
77.00
76.74
65.61
64.09
55.86
53.27
52.52
48.11
48.02
42.35
41.15
39.47
38.18
35.85
33.43
28.70
28.25
24.00
21.50
21.41
19.03
15.18
13.59
11.91

Single Mass Analysis
Tolerance = 1.0 PPM / DBE: min = -1.5, max = 50.0
Element prediction: Off
Number of isotope peaks used for i-FIT = 3
Monoisotopic Mass, Even Electron Ions
641 formula(e) evaluated with 1 results within limits (up to 50 best isotopic matches for each mass)
Elements Used:

Mass	Calc. Mass	mDa	PPM	DBE	Formula	i-FIT	i-FIT Norm	Fit Conf %	C	H	N	O
473.3631	473.3631	0.0	0.0	6.5	C30 H49 O4	13.1	n/a	n/a	30	49		4

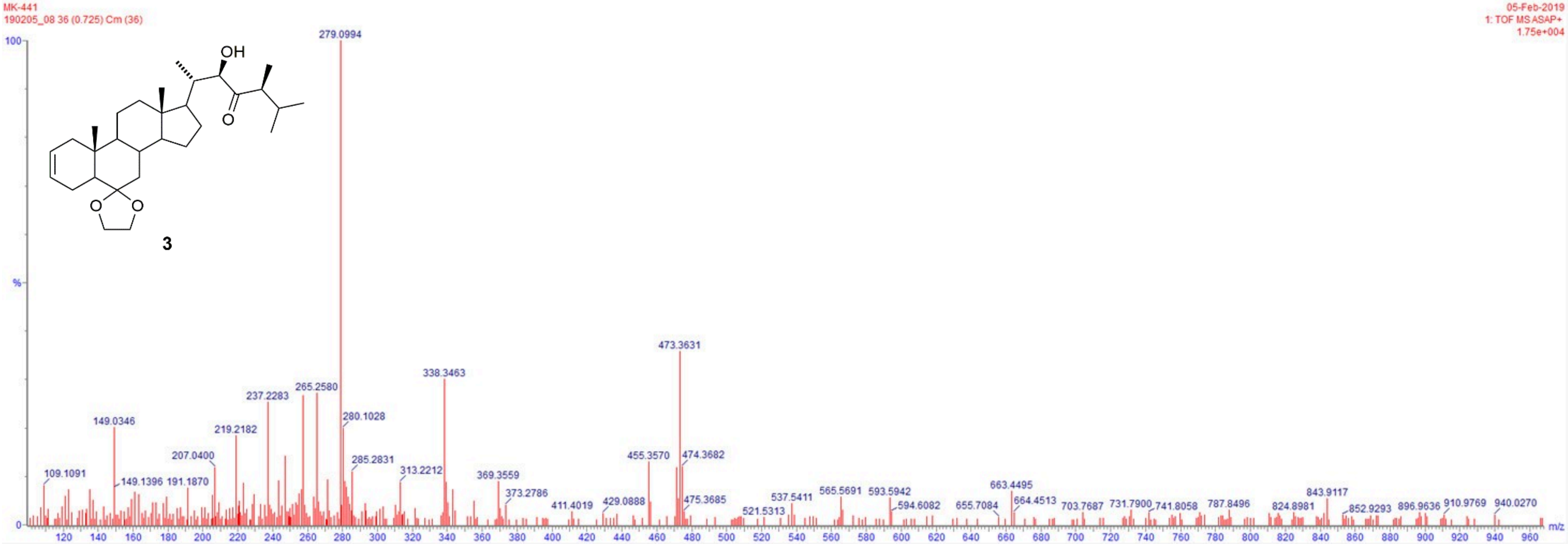

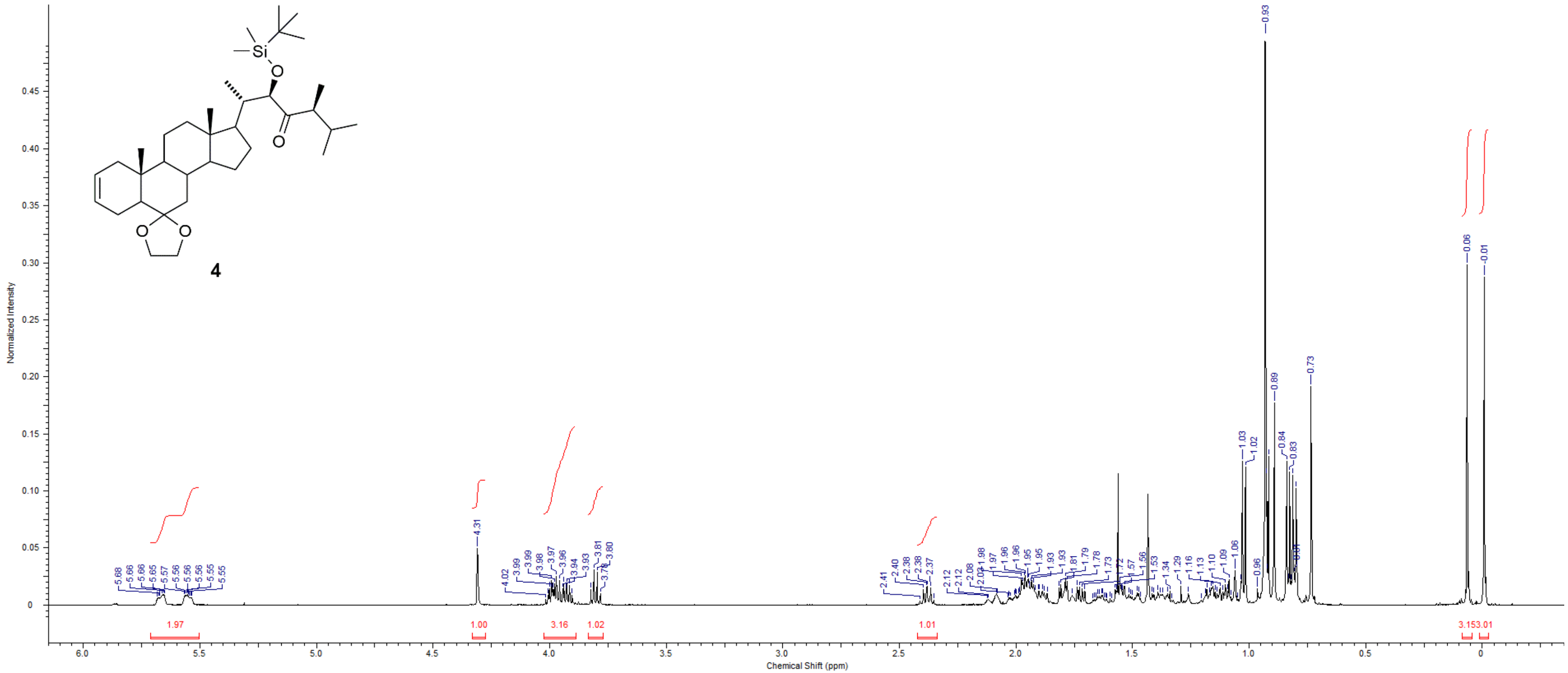

Si
O
O
O
O
4
Normalized Intensity
Chemical Shift (ppm)
1.97
1.00
3.16
1.02
1.01
3.15
3.01

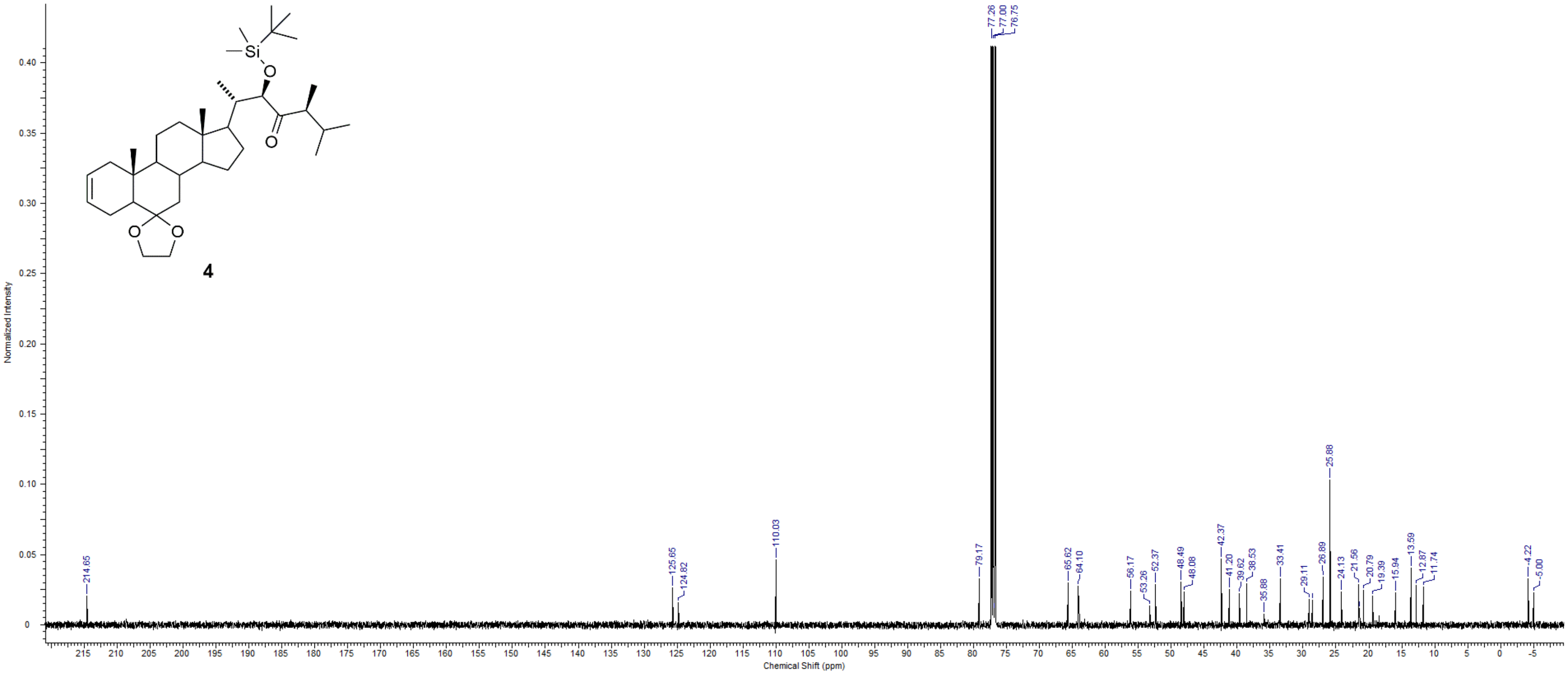

4
Normalized Intensity
Chemical Shift (ppm)
214.65
125.65
124.82
110.03
79.17
77.26
77.00
76.75
65.62
64.10
56.17
53.26
52.37
48.49
48.08
42.37
41.20
39.62
38.53
35.88
33.41
29.11
26.89
25.88
24.13
21.56
20.79
19.39
15.94
13.59
12.87
11.74
-4.22
-5.00

Single Mass Analysis
Tolerance = 1.0 PPM / DBE: min = -1.5, max = 50.0
Element prediction: Off
Number of isotope peaks used for i-FIT = 3
Monoisotopic Mass, Even Electron Ions
336 formula(e) evaluated with 1 results within limits (up to 50 best isotopic matches for each mass)
Elements Used:

Mass	Calc. Mass	mDa	PPM	DBE	Formula	i-FIT	i-FIT Norm	Fit Conf %	C	H	O	Si
587.4491	587.4496	-0.5	-0.9	6.5	C36 H63 O4 Si	55.7	n/a	n/a	36	63	4	1

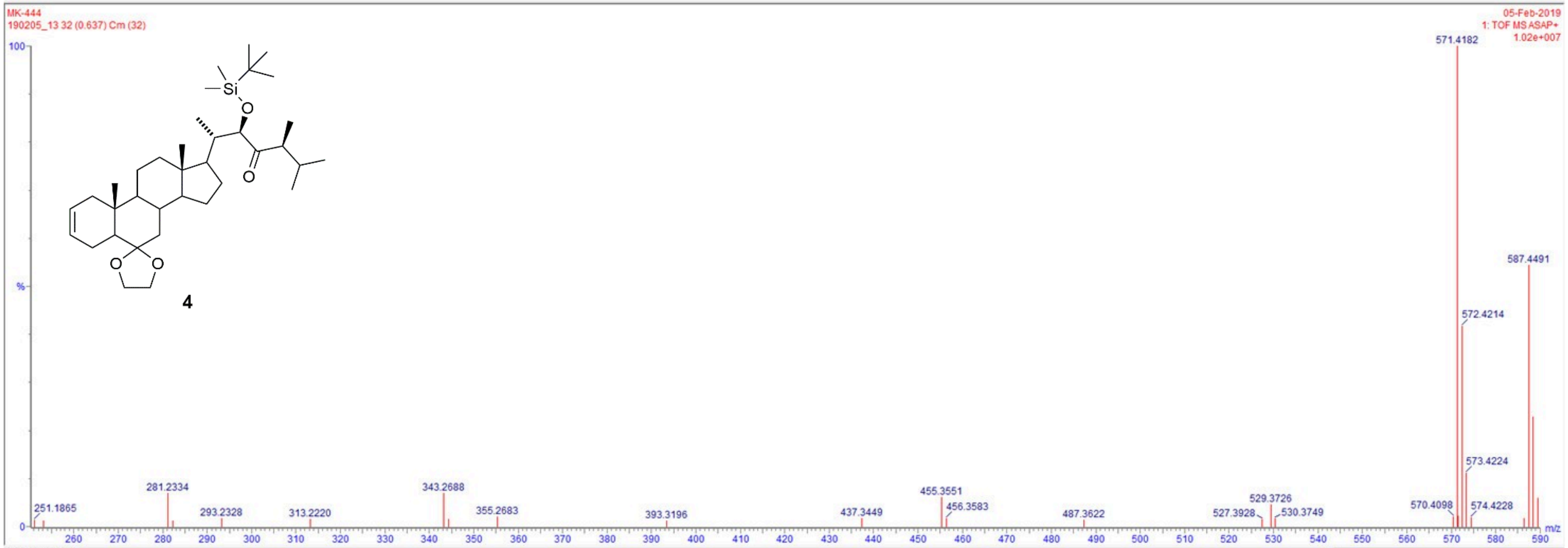

For Help, press F1

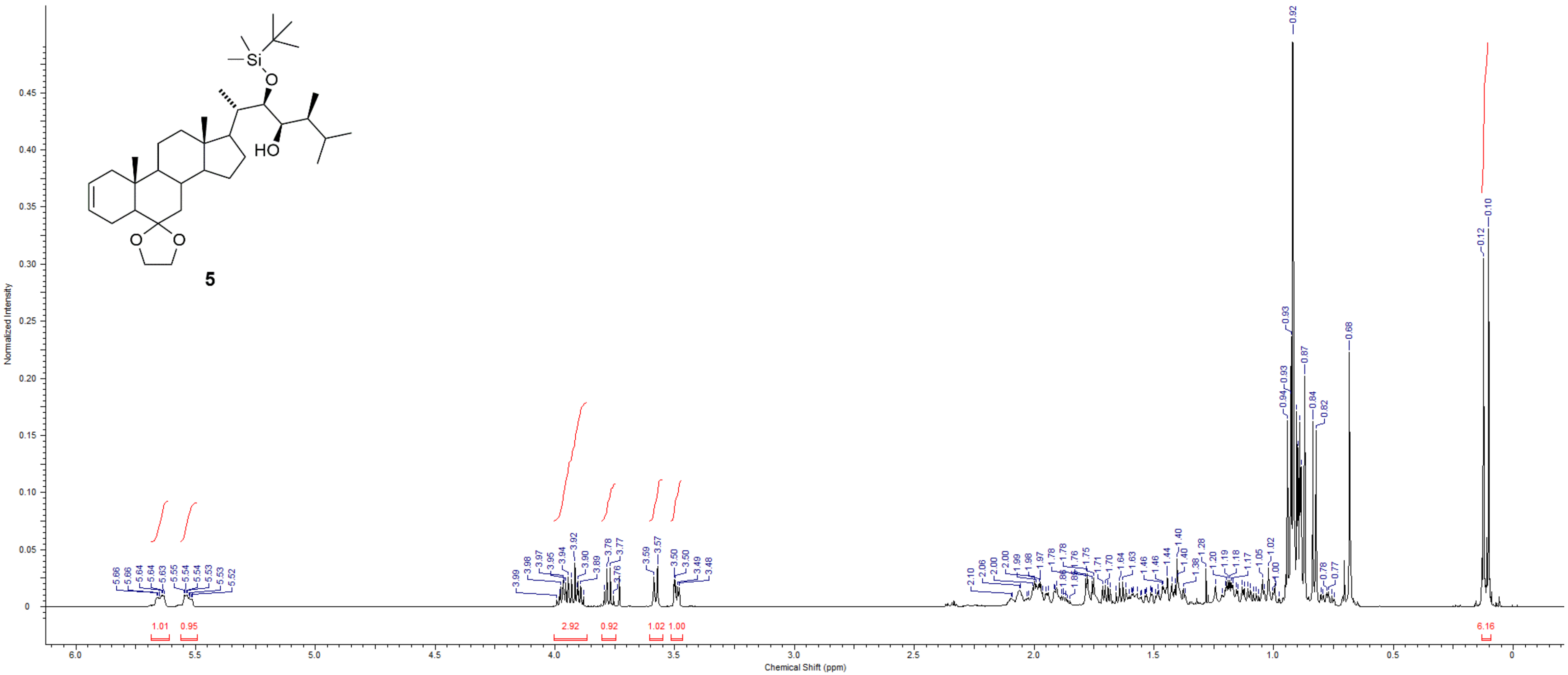

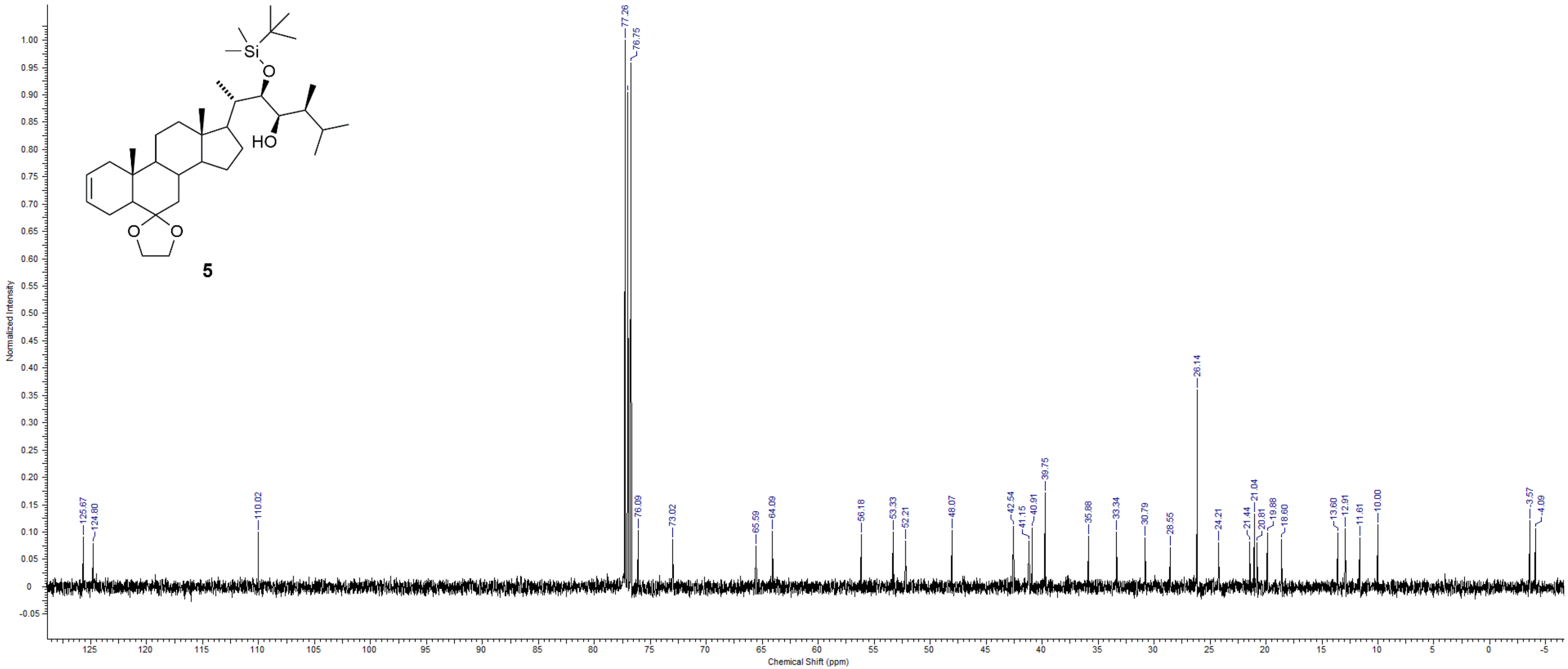

5

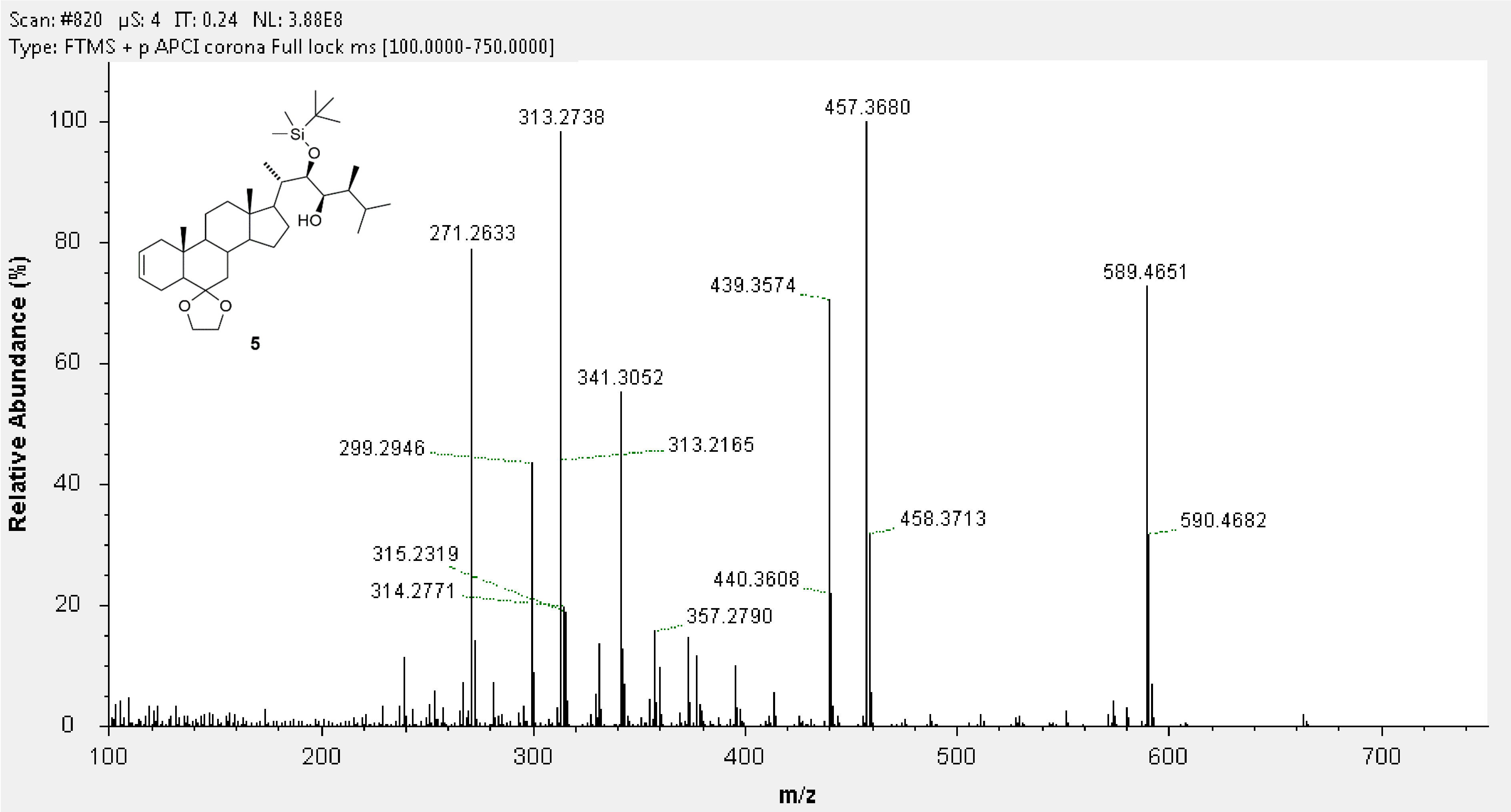
Scan: #820 µS: 4 IT: 0.24 NL: 3.88E8
Type: FTMS + p APCI corona Full lock ms [100.0000-750.0000]
Relative Abundance (%)
m/z
Si
O
HO
O
O
5
313.2738
457.3680
271.2633
439.3574
589.4651
341.3052
299.2946
313.2165
458.3713
590.4682
315.2319
314.2771
440.3608
357.2790
0
20
40
60
80
100
100
200
300
400
500
600
700

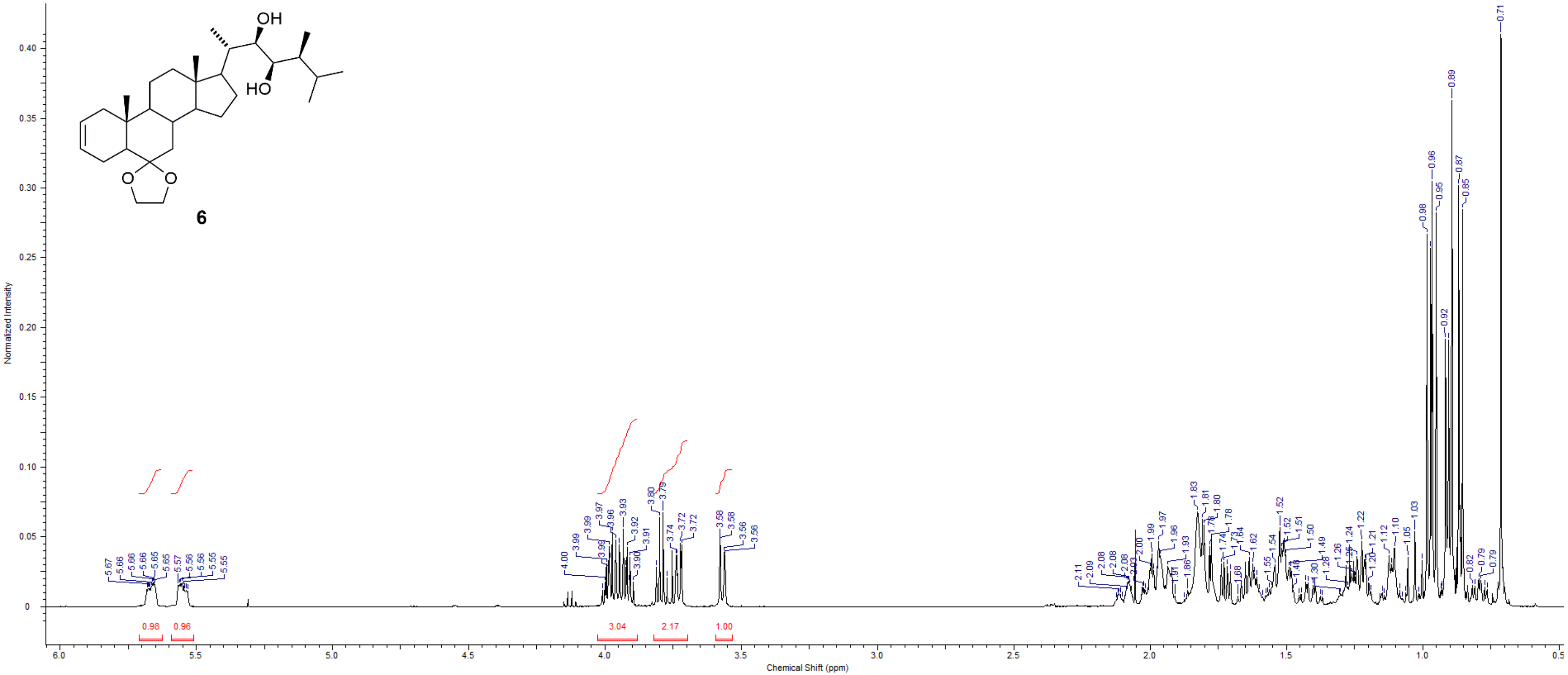

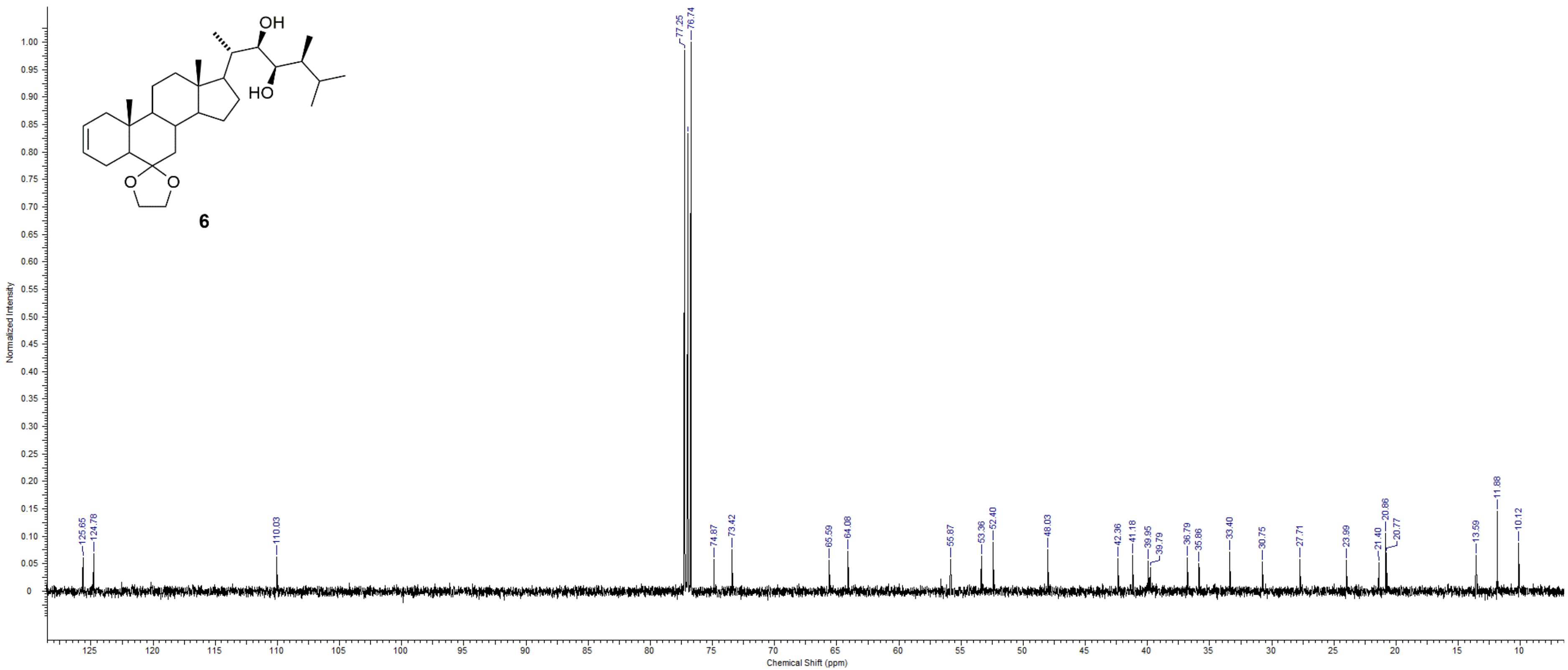

OH
HO
O
O
6
77.25
76.74
125.65
124.78
110.03
74.87
73.42
65.59
64.08
55.87
53.36
52.40
48.03
42.36
41.18
39.95
39.79
36.79
35.86
33.40
30.75
27.71
23.99
21.40
20.86
20.77
13.59
11.88
10.12
Normalized Intensity
Chemical Shift (ppm)

Single Mass Analysis
Tolerance = 1.0 PPM / DBE: min = -1.5, max = 50.0
Element prediction: Off
Number of isotope peaks used for i-FIT = 3
Monoisotopic Mass, Even Electron Ions
648 formula(e) evaluated with 1 results within limits (up to 50 best isotopic matches for each mass)
Elements Used:

Mass	Calc. Mass	mDa	PPM	DBE	Formula	i-FIT	i-FIT Norm	Fit Conf %	C	H	N	O
475.3787	475.3787	0.0	0.0	5.5	C30 H51 O4	119.2	n/a	n/a	30	51		4

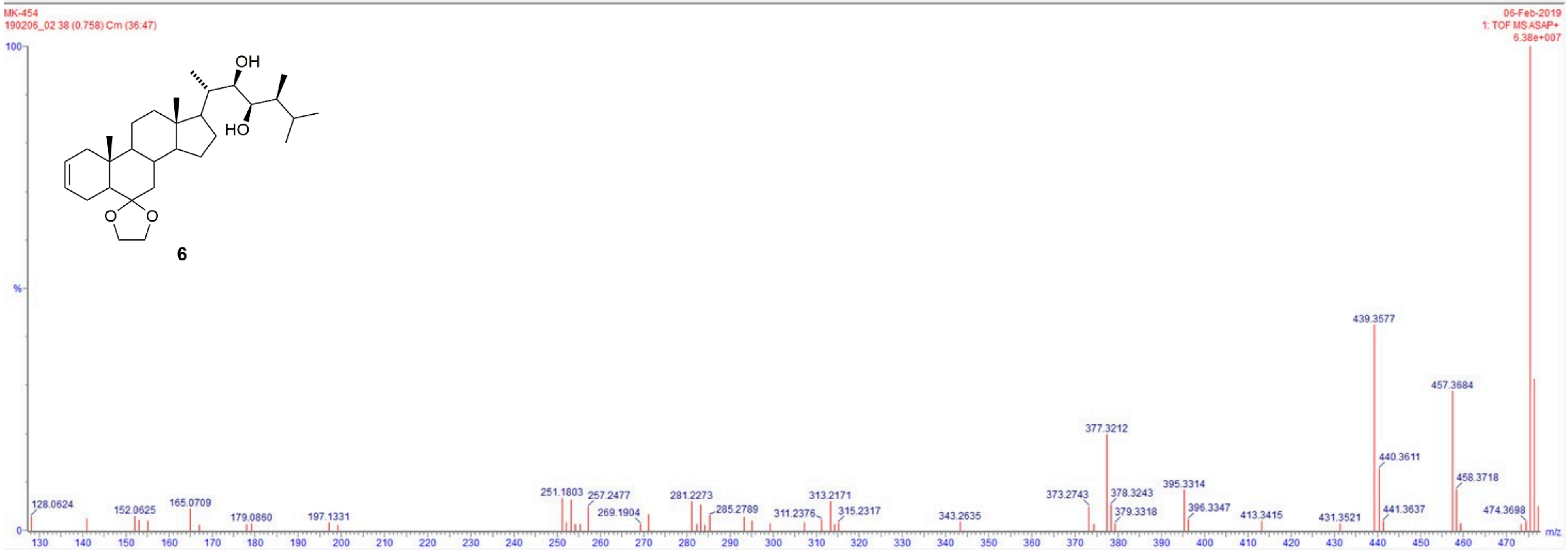

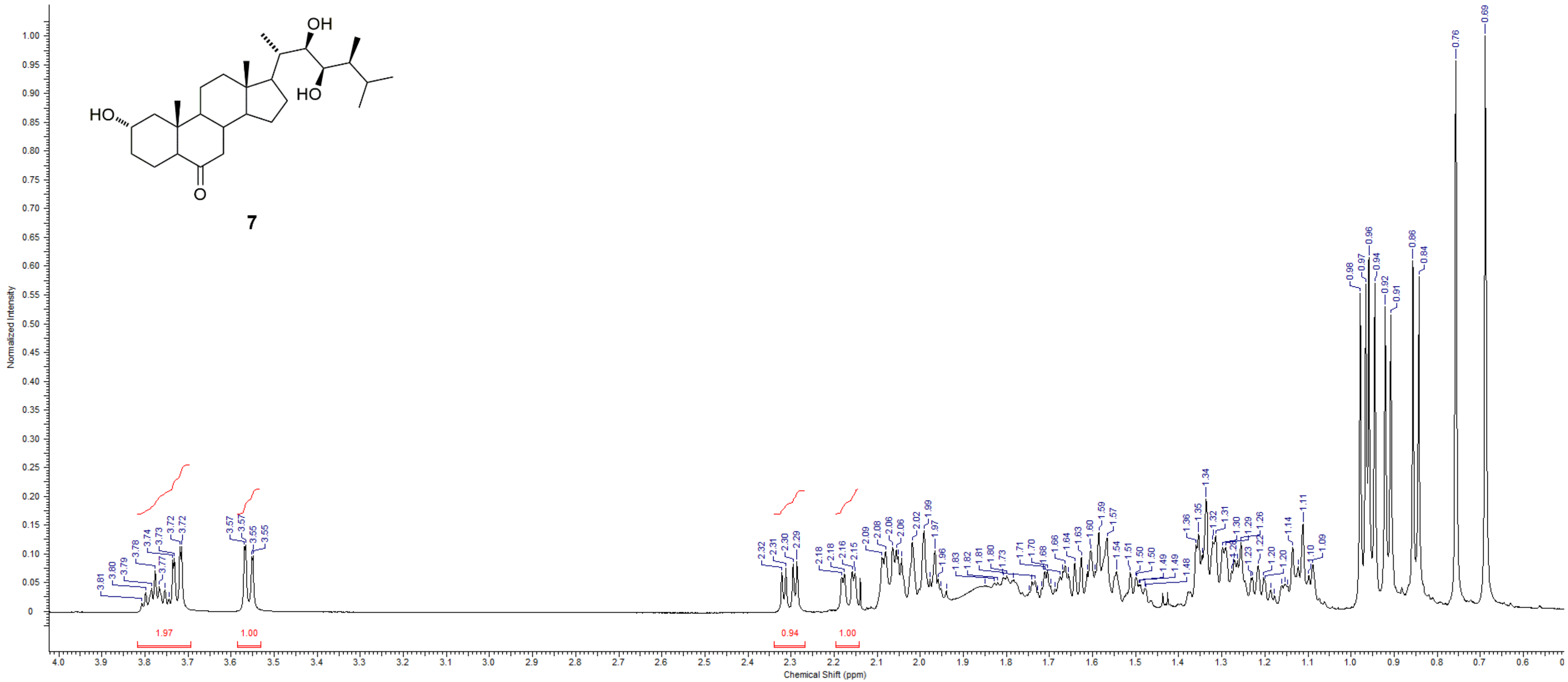
OH
HO
HO
O
7
Normalized Intensity
Chemical Shift (ppm)

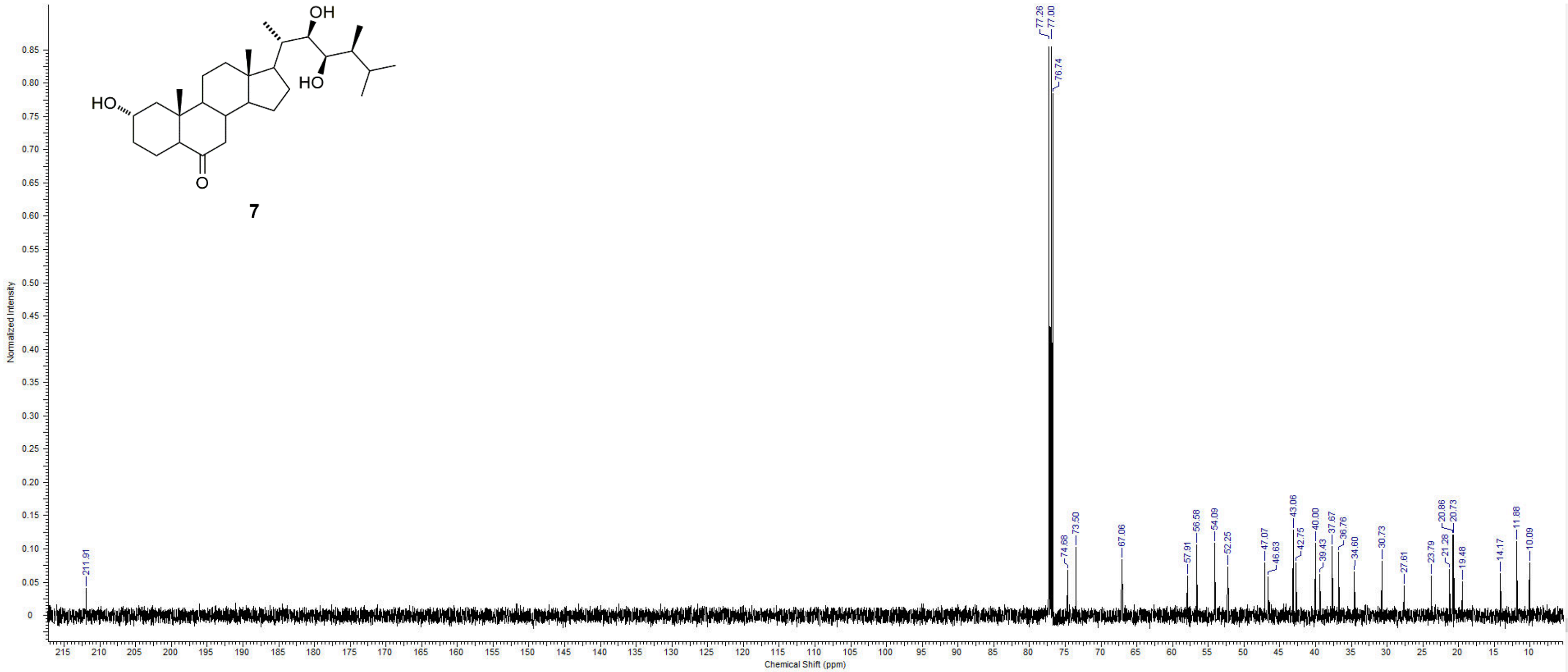
HO
OH
HO
O
7
Normalized Intensity
Chemical Shift (ppm)
211.91
77.26
77.00
76.74
74.68
73.50
67.06
57.91
56.58
54.09
52.25
47.07
46.63
43.06
42.75
40.00
39.43
37.67
36.76
34.60
30.73
27.61
23.79
21.28
20.86
20.73
19.48
14.17
11.88
10.09

Single Mass Analysis
Tolerance = 1.0 PPM / DBE: min = -1.5, max = 50.0
Element prediction: Off
Number of isotope peaks used for i-FIT = 3
Monoisotopic Mass, Even Electron Ions
588 formula(e) evaluated with 1 results within limits (up to 50 best isotopic matches for each mass)
Elements Used:

Mass	Calc. Mass	mDa	PPM	DBE	Formula	i-FIT	i-FIT Norm	Fit Conf %	C	H	N	O
449.3627	449.3631	-0.4	-0.9	4.5	C28 H49 O4	37.6	n/a	n/a	28	49		4

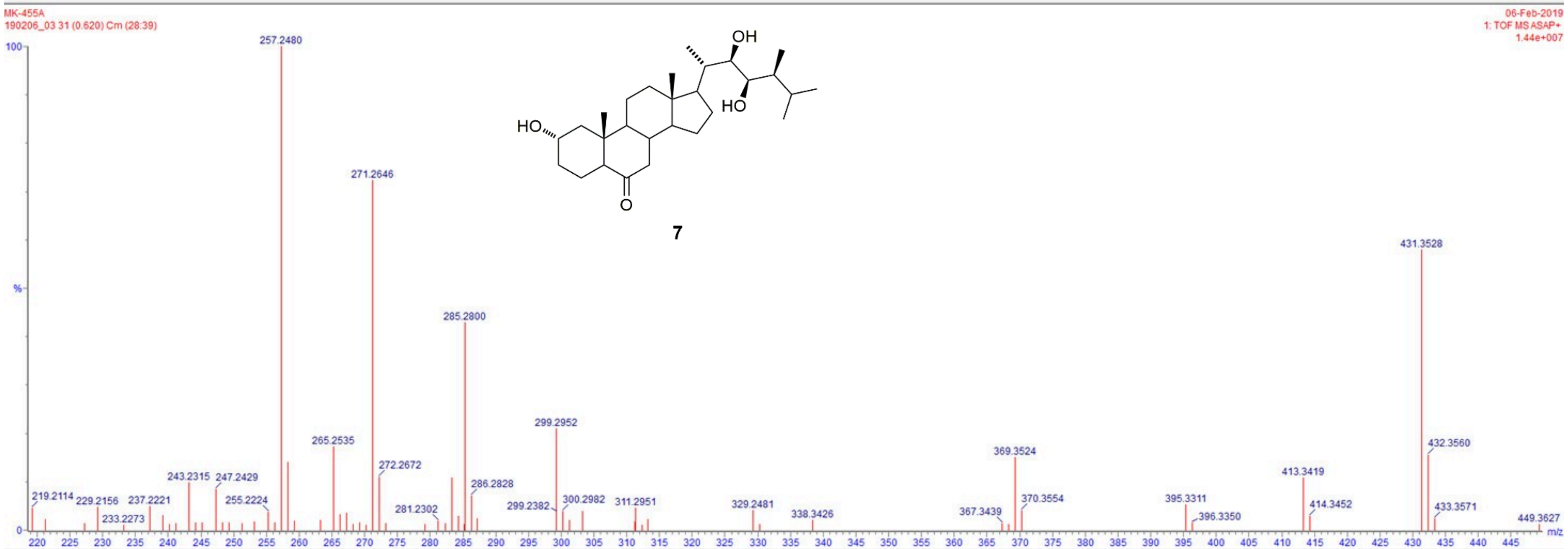

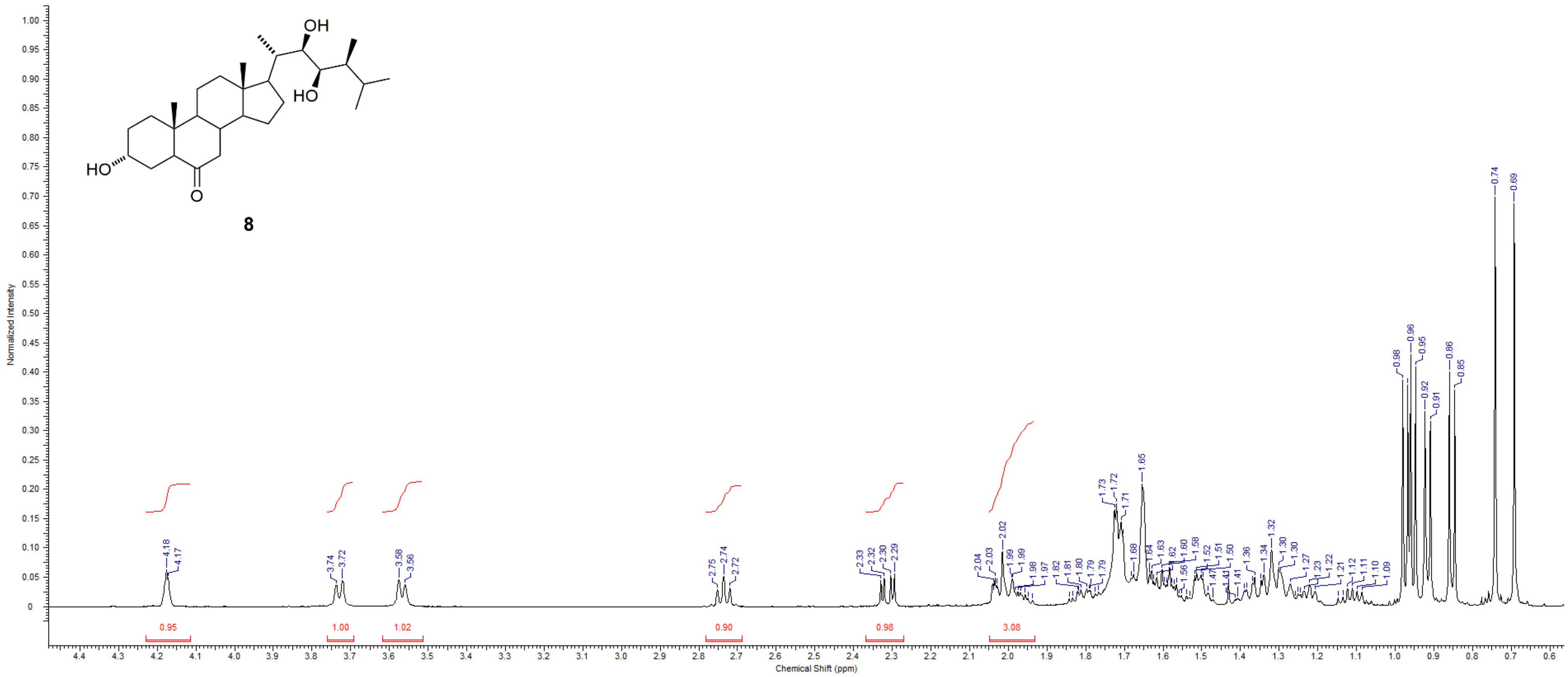

8

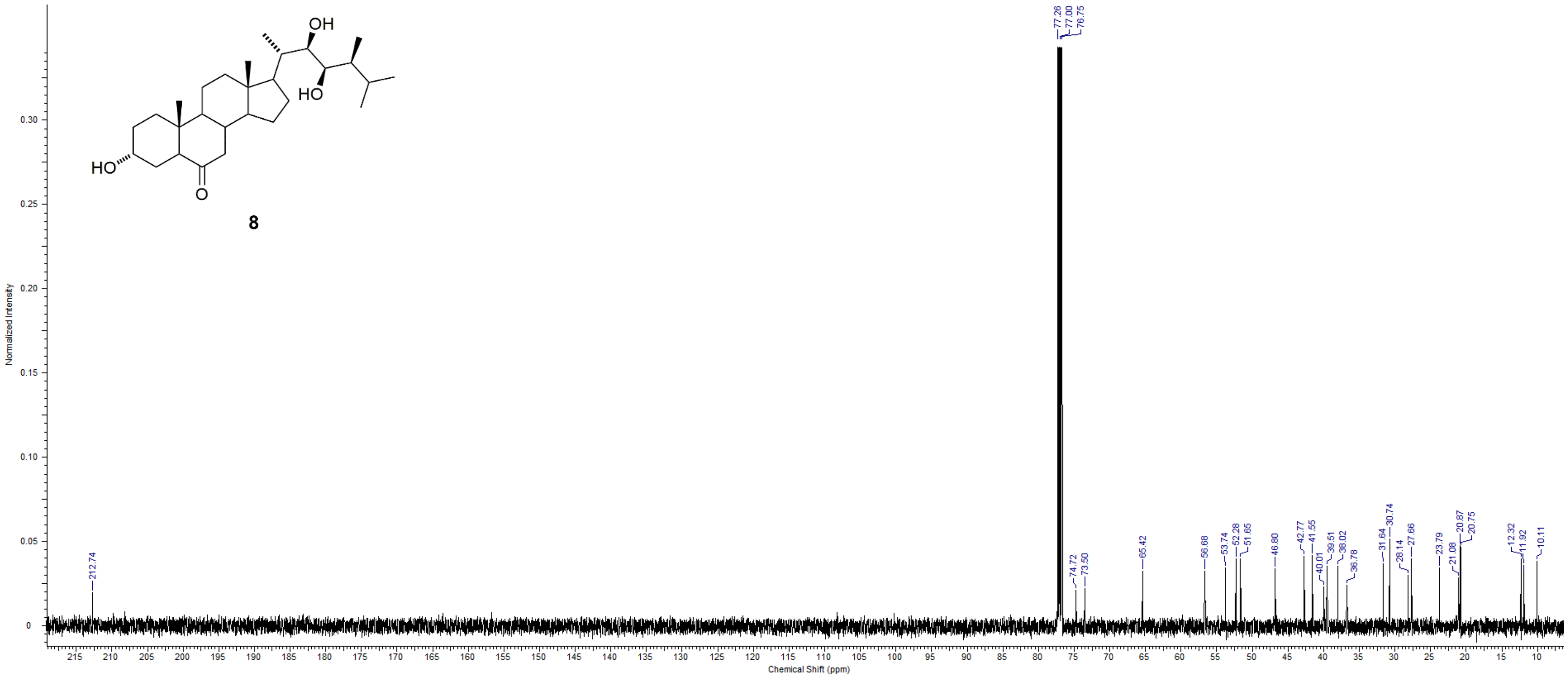
OH
HO
HO
O
8
212.74
77.26
77.00
76.75
74.72
73.50
65.42
56.68
53.74
52.28
51.65
46.80
42.77
41.55
40.01
39.51
38.02
36.78
31.64
30.74
28.14
27.66
23.79
21.08
20.87
20.75
12.32
11.92
10.11
Normalized Intensity
Chemical Shift (ppm)

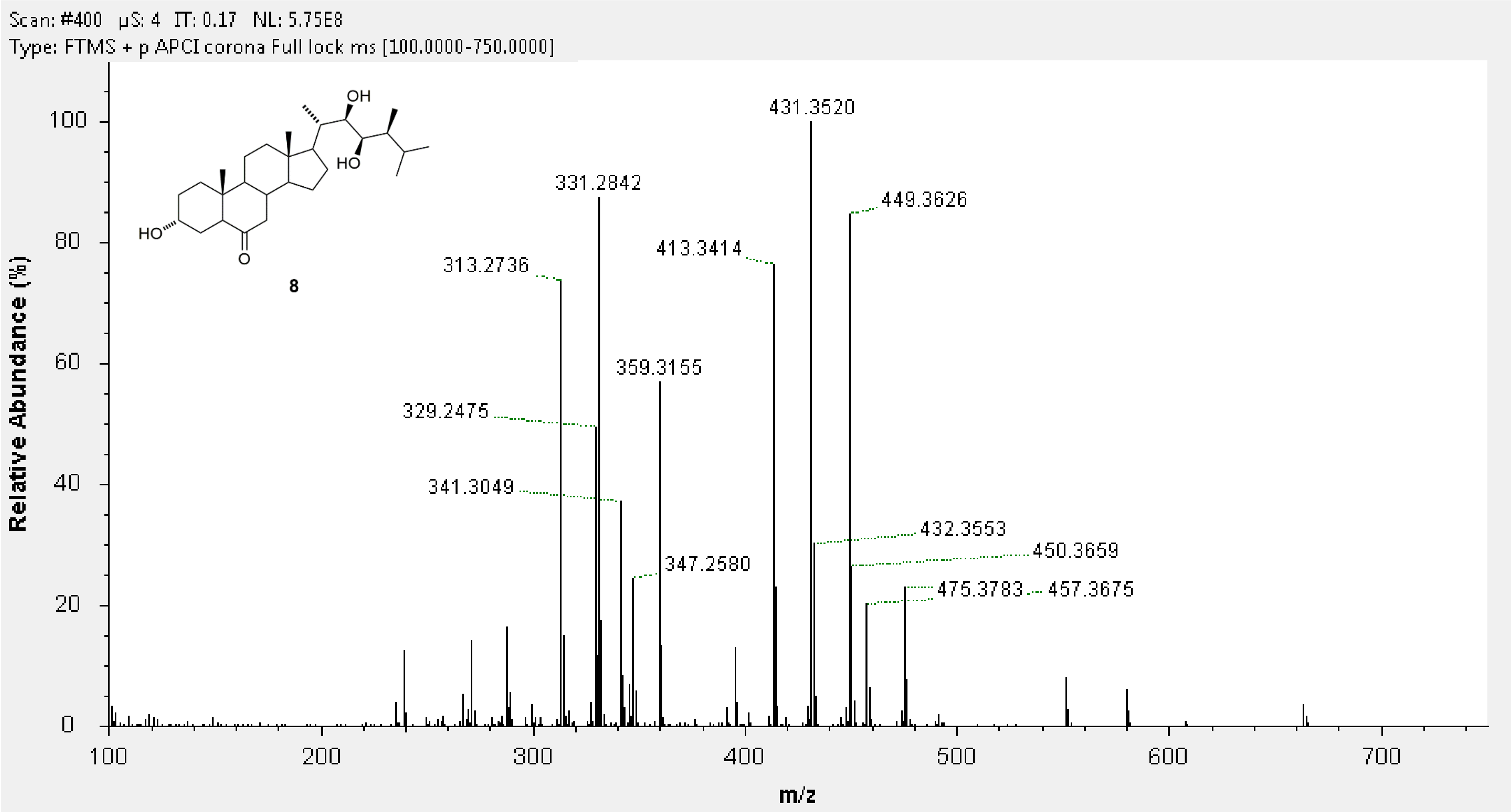

Scan: #400 µS: 4 IT: 0.17 NL: 5.75E8
Type: FTMS + p APCI corona Full lock ms [100.0000-750.0000]
Relative Abundance (%)
m/z
OH
HO
HO
O
8
431.3520
331.2842
449.3626
413.3414
313.2736
359.3155
329.2475
341.3049
432.3553
450.3659
347.2580
475.3783
457.3675

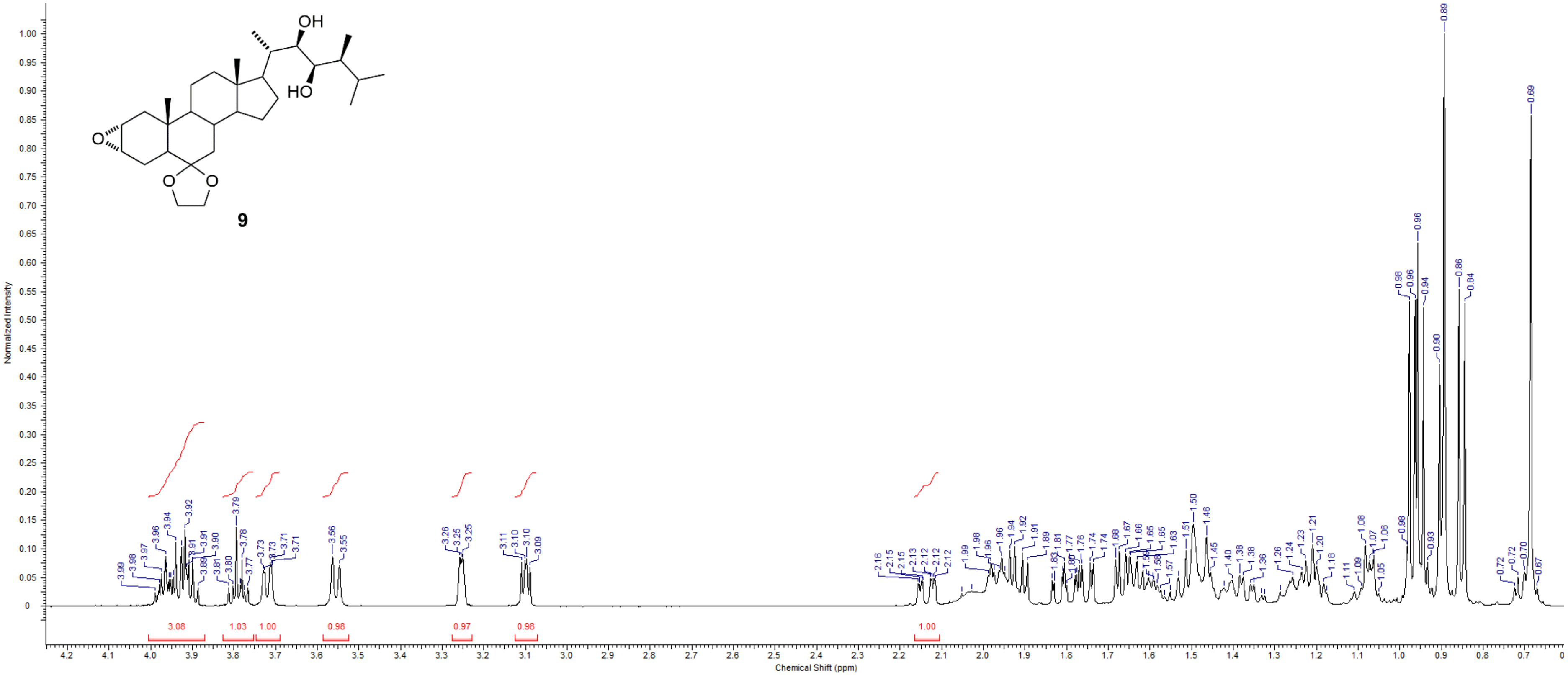

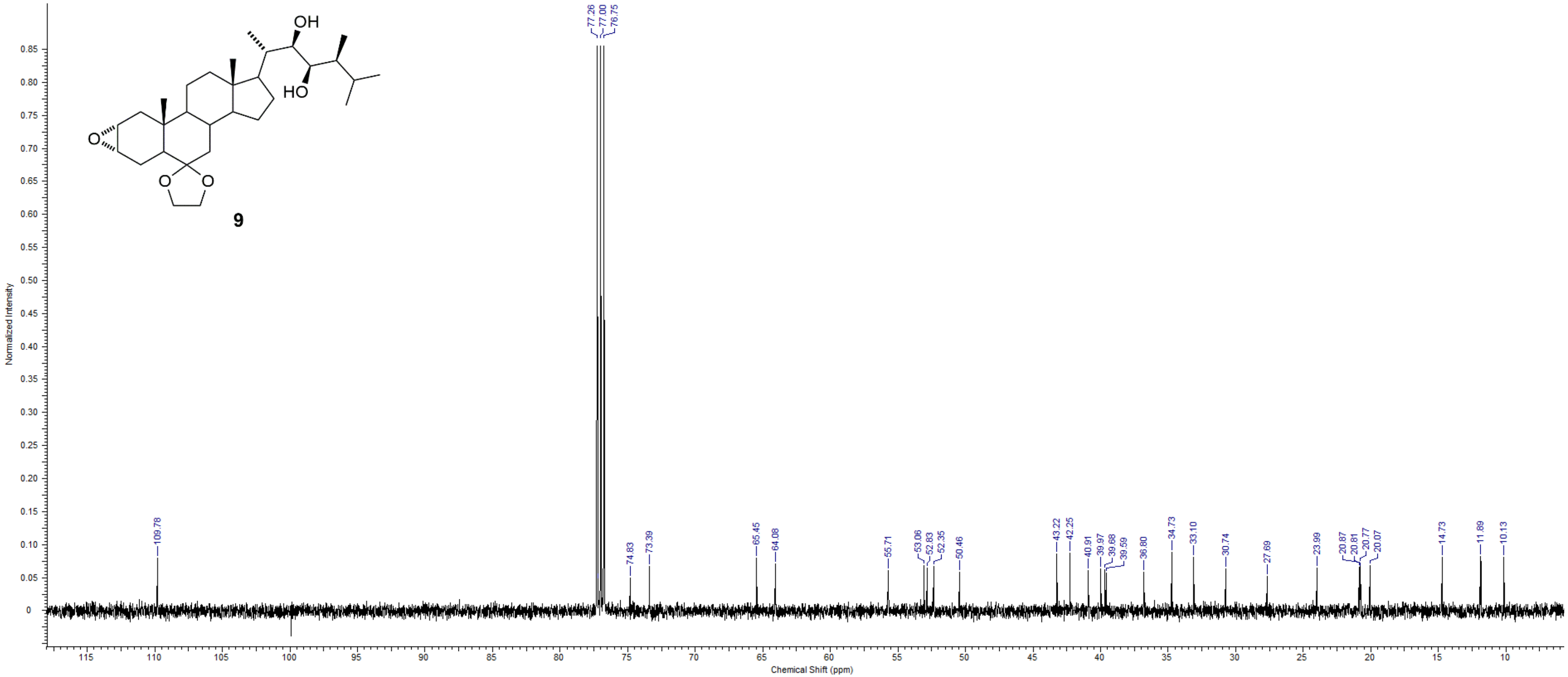

OH
HO
O
9
Normalized Intensity
Chemical Shift (ppm)
109.78
77.26
77.00
76.75
74.83
73.39
65.45
64.08
55.71
53.06
52.83
52.35
50.46
43.22
42.25
40.91
39.97
39.68
39.59
36.80
34.73
33.10
30.74
27.69
23.99
20.87
20.81
20.77
20.07
14.73
11.89
10.13

Single Mass Analysis
Tolerance = 1.0 PPM / DBE: min = -1.5, max = 50.0
Element prediction: Off
Number of isotope peaks used for i-FIT = 3
Monoisotopic Mass, Even Electron Ions
688 formula(e) evaluated with 1 results within limits (up to 50 best isotopic matches for each mass)
Elements Used:

Mass	Calc. Mass	mDa	PPM	DBE	Formula	i-FIT	i-FIT Norm	Fit Conf %	C	H	N	O
491.3739	491.3736	0.3	0.6	5.5	C30 H51 O5	81.2	n/a	n/a	30	51		5

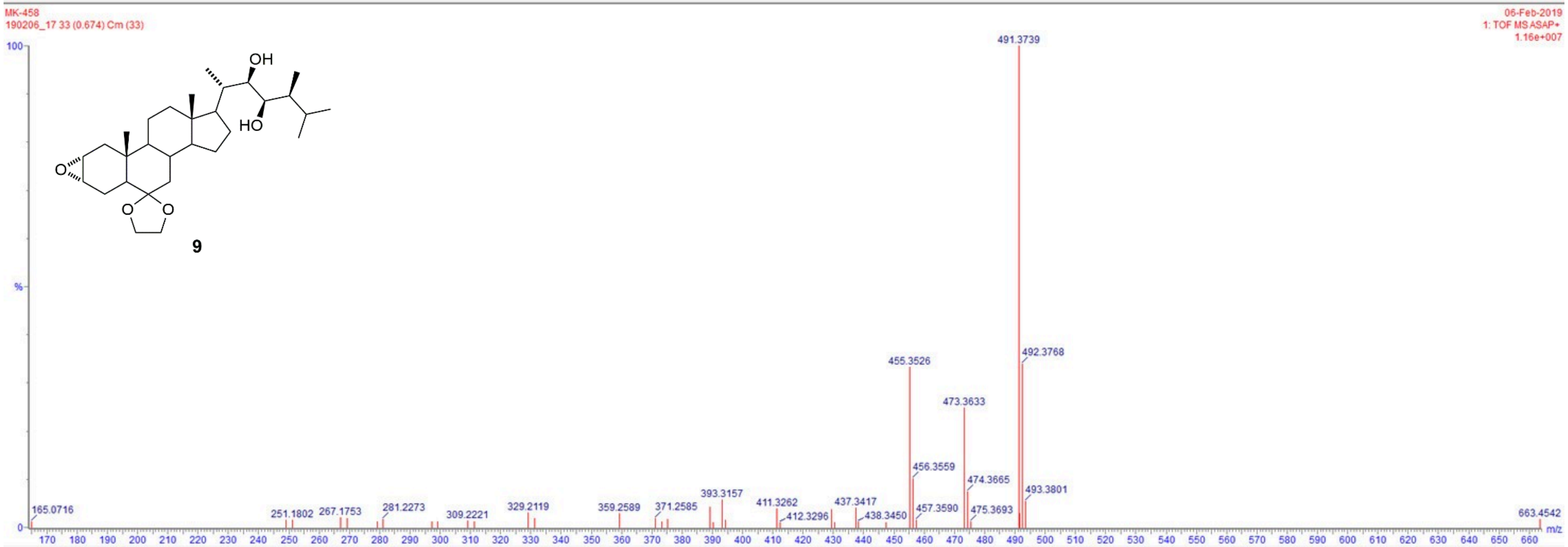

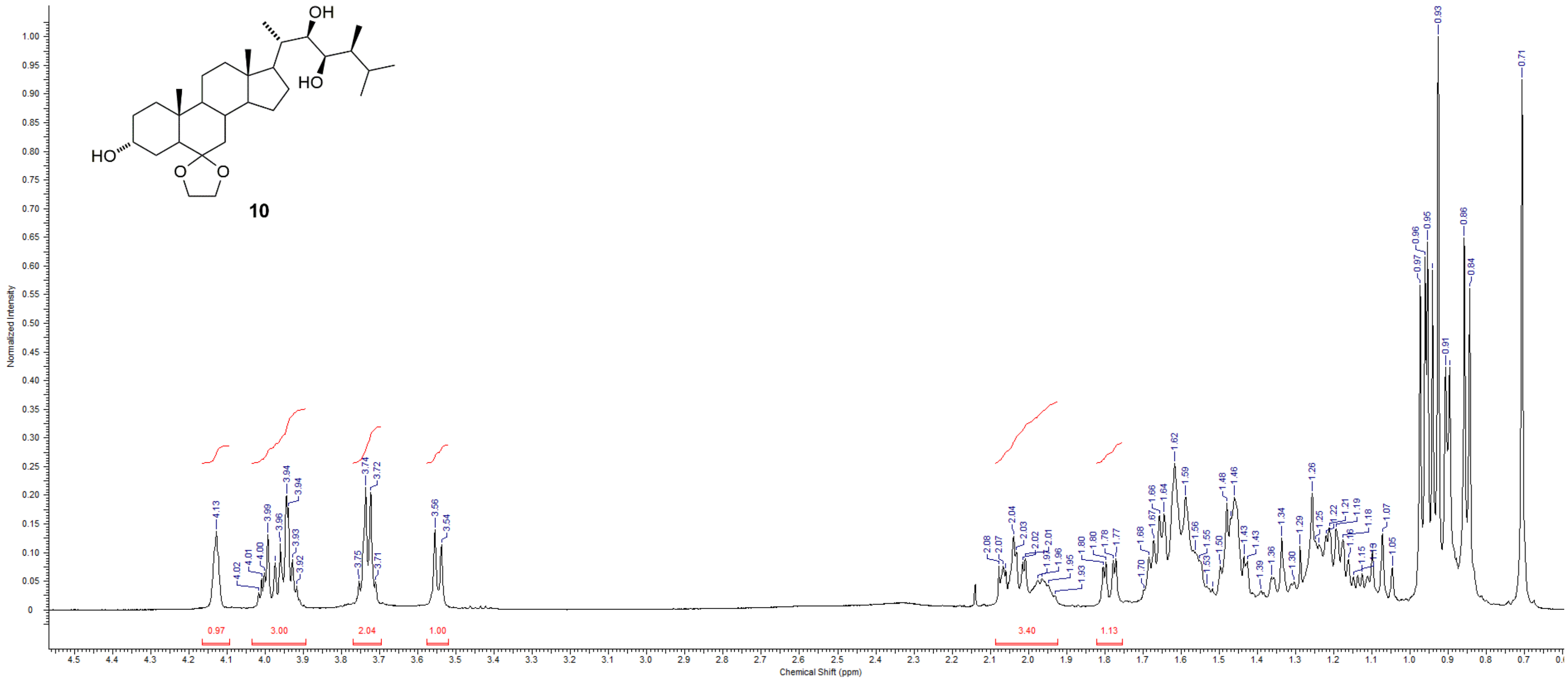

10

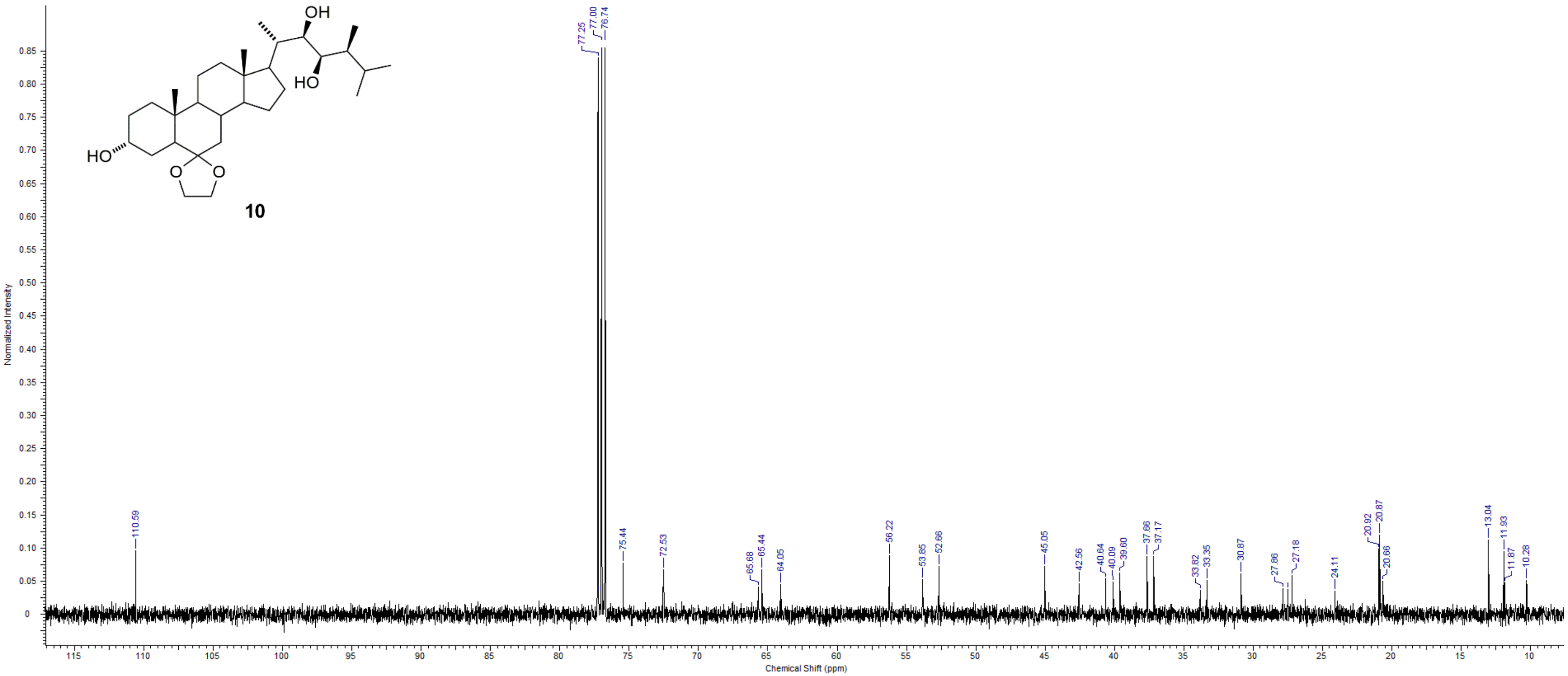
OH
HO
HO
O O
10
77.25
77.00
76.74
110.59
75.44
72.53
65.68
65.44
64.05
56.22
53.85
52.66
45.05
42.56
40.64
40.09
39.60
37.66
37.17
33.82
33.35
30.87
27.86
27.18
24.11
20.92
20.87
20.66
13.04
11.93
11.87
10.28
Normalized Intensity
Chemical Shift (ppm)

Single Mass Analysis
Tolerance = 1.0 PPM / DBE: min = -1.5, max = 50.0
Element prediction: Off
Number of isotope peaks used for i-FIT = 3
Monoisotopic Mass, Even Electron Ions
691 formula(e) evaluated with 1 results within limits (up to 50 best isotopic matches for each mass)
Elements Used:

Mass	Calc. Mass	mDa	PPM	DBE	Formula	i-FIT	i-FIT Norm	Fit Conf %	C	H	N	O
493.3895	493.3893	0.2	0.4	4.5	C30 H53 O5	47.4	n/a	n/a	30	53		5

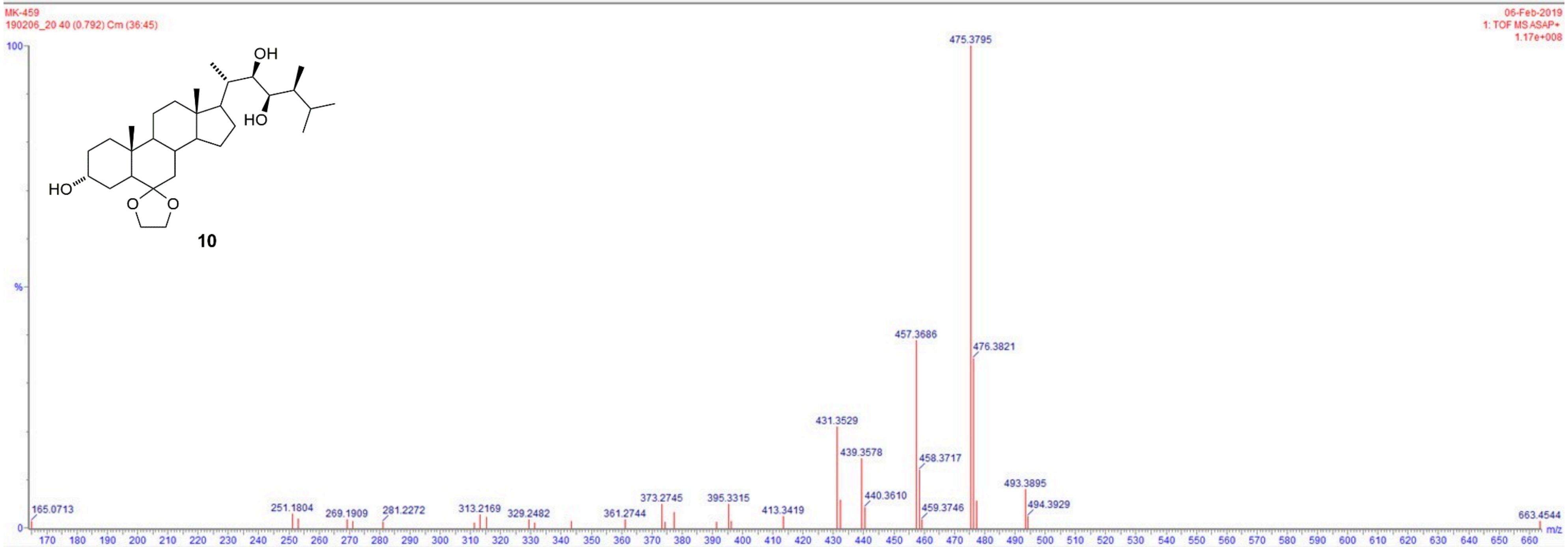

For Help, press F1

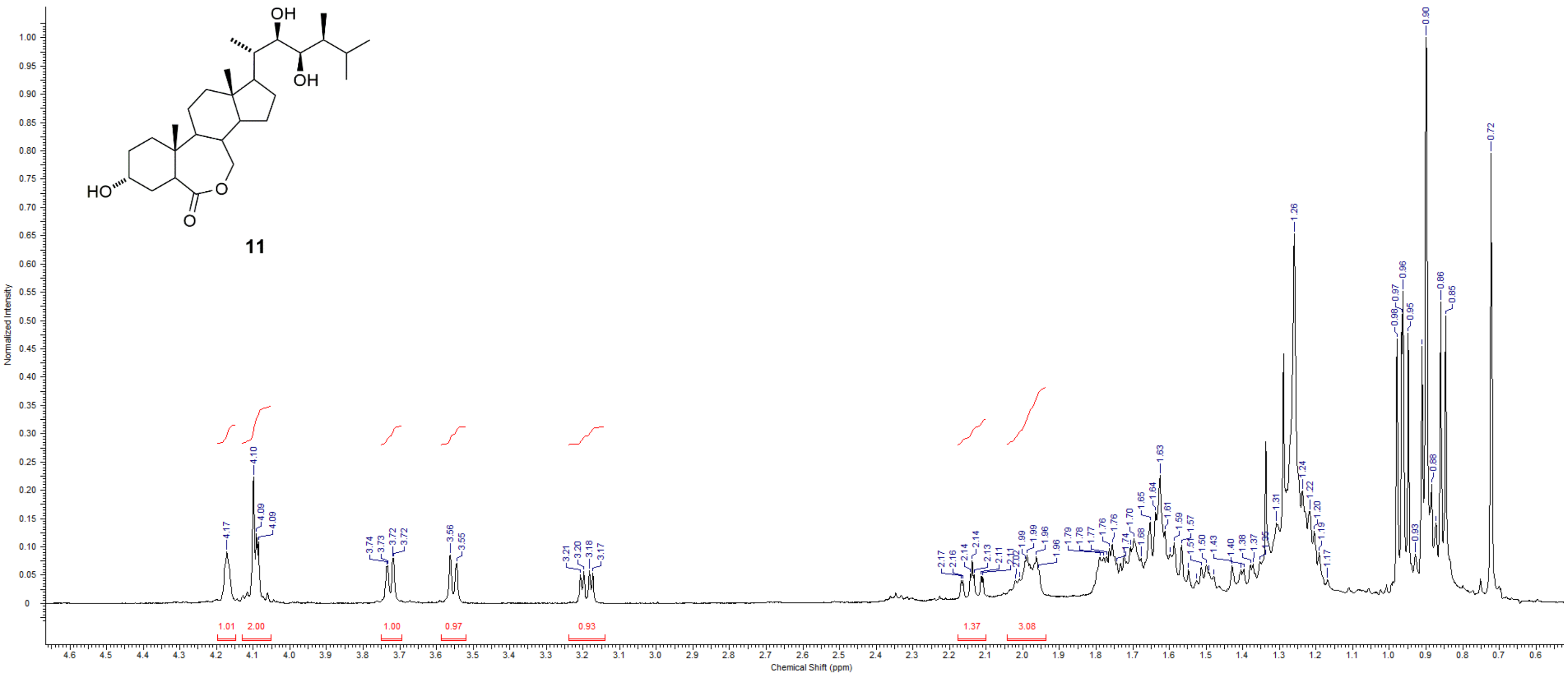
OH
OH
HO
O
O
11
Normalized Intensity
Chemical Shift (ppm)

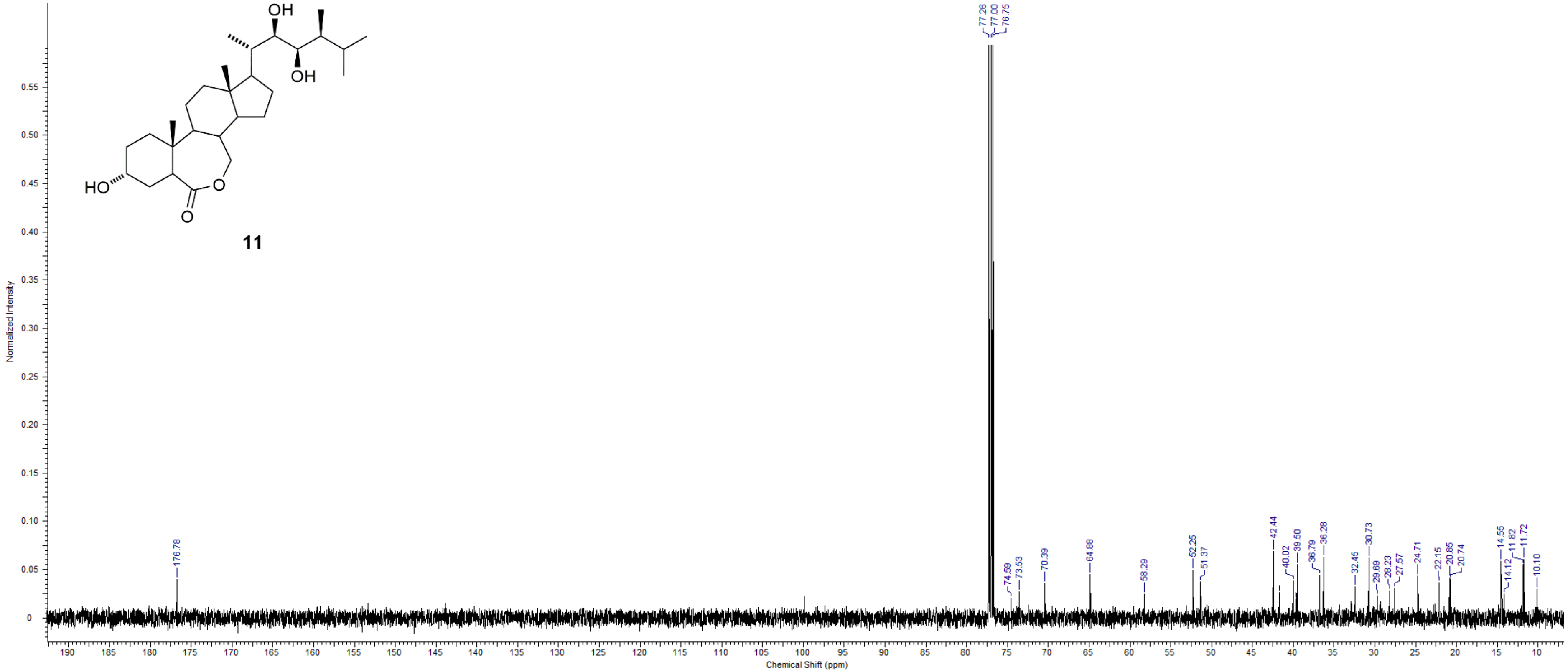

OH
OH
HO
O
O
11
77.26
77.00
76.75
176.78
74.59
73.53
70.39
64.88
58.29
52.25
51.37
42.44
40.02
39.50
36.79
36.28
32.45
30.73
29.69
28.23
27.57
24.71
22.15
20.85
20.74
14.55
14.12
11.82
11.72
10.10
Normalized Intensity
Chemical Shift (ppm)

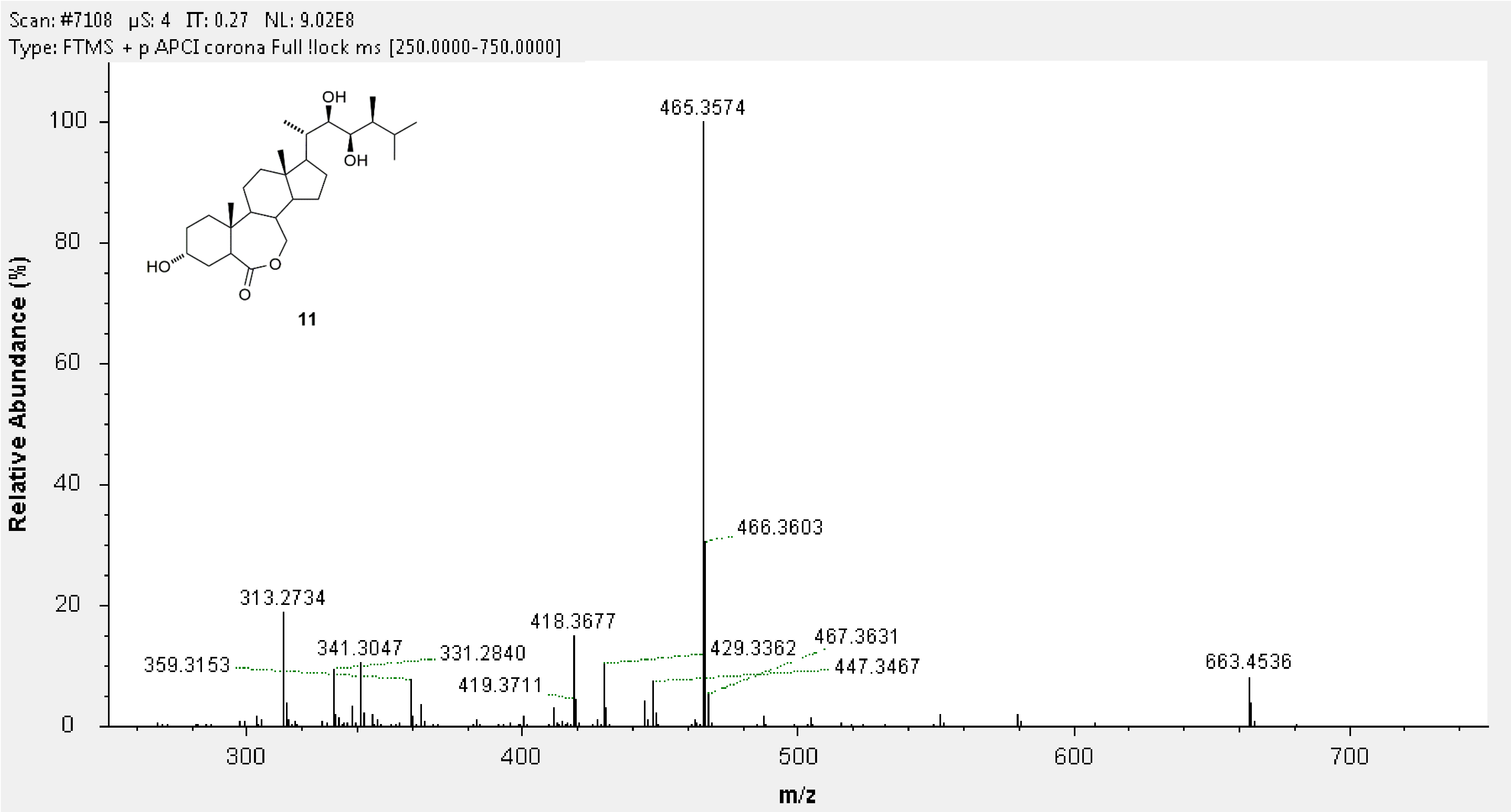
Scan: #7108 μS: 4 IT: 0.27 NL: 9.02E8
Type: FTMS + p APCI corona Full !lock ms [250.0000-750.0000]
OH
OH
HO
O
O
11
Relative Abundance (%)
0
20
40
60
80
100
465.3574
466.3603
313.2734
418.3677
467.3631
341.3047
331.2840
429.3362
359.3153
447.3467
663.4536
419.3711
300
400
500
600
700
m/z

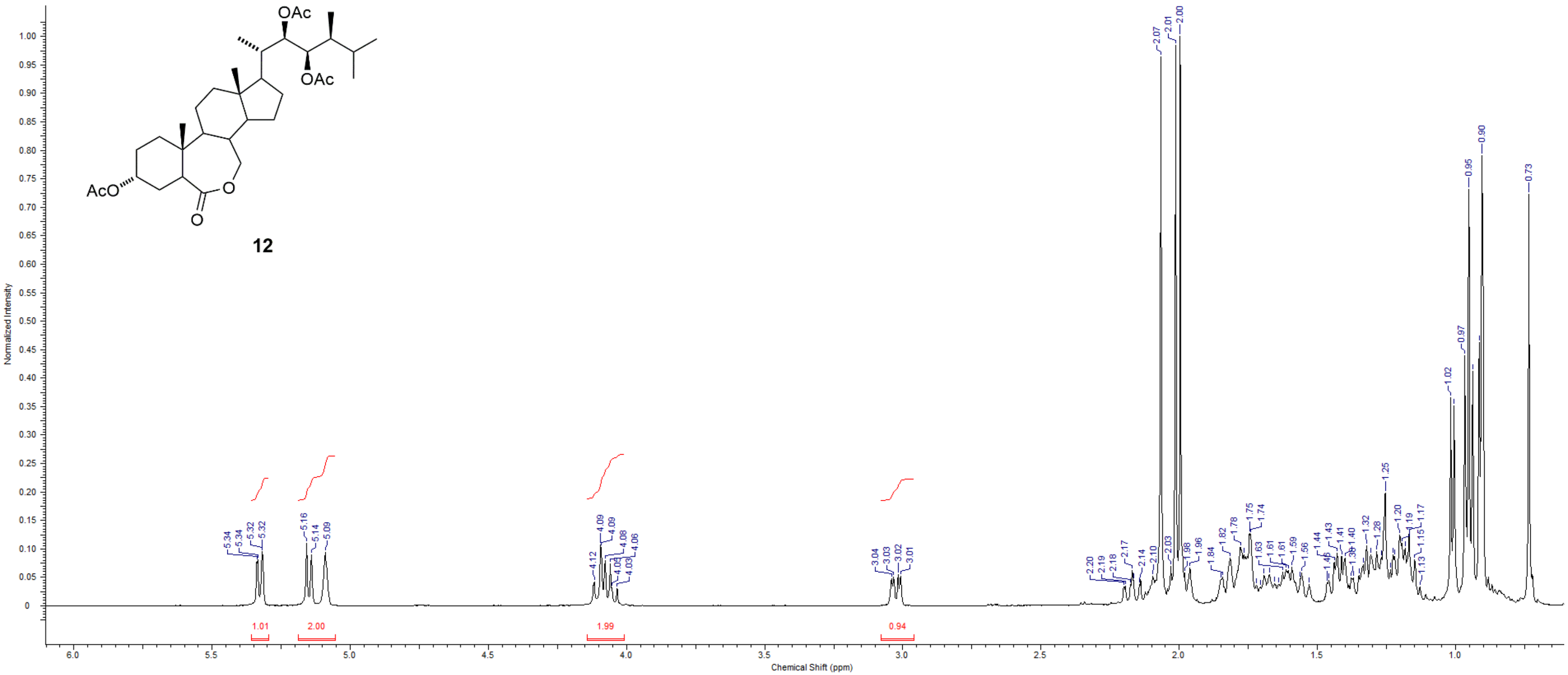
OAc
OAc
AcO
O
O
12
Normalized Intensity
Chemical Shift (ppm)

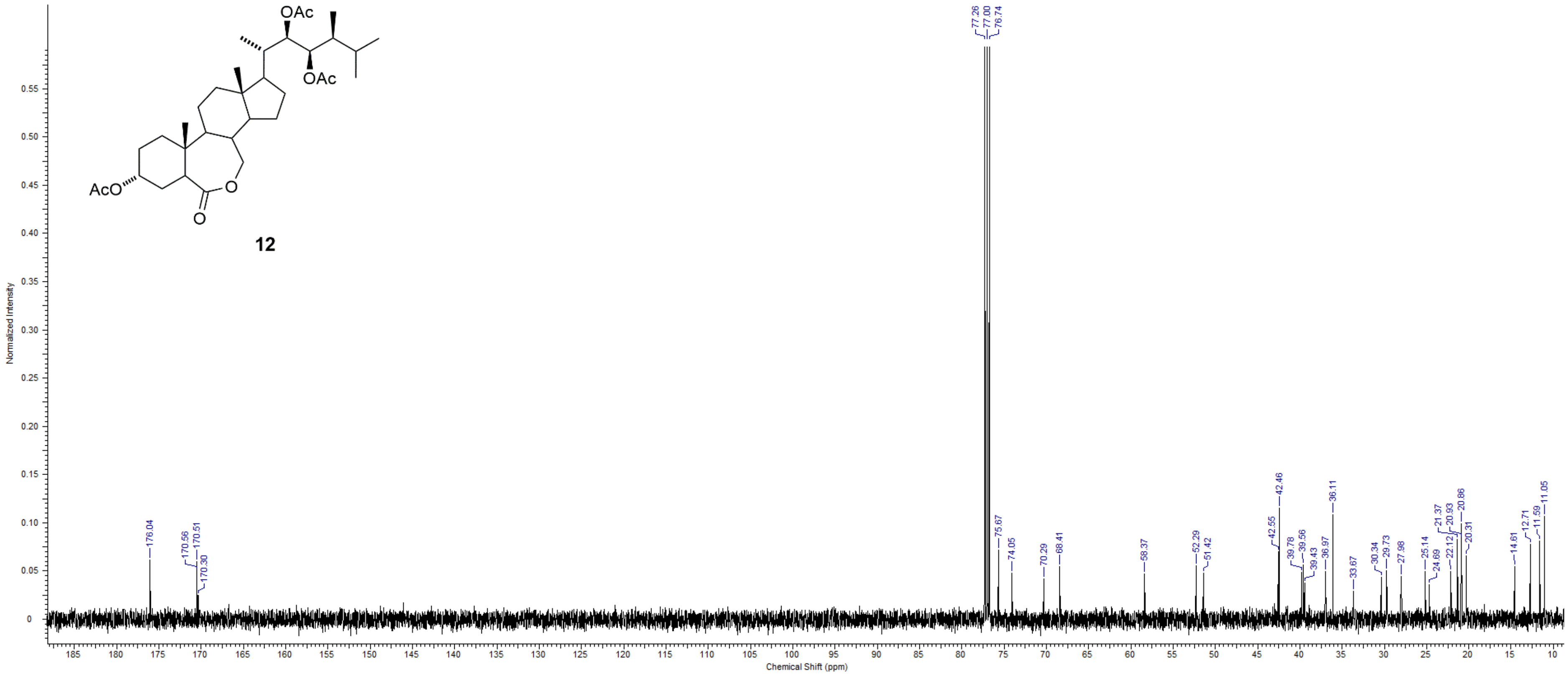
OAc
OAc
AcO
O
O
Normalized Intensity
Chemical Shift (ppm)
177.26 177.00 176.74
176.04
170.56 170.51 170.30
75.67
74.05
70.29
68.41
58.37
52.29
51.42
42.55 42.46
39.78 39.56 39.43
36.97 36.11
33.67
30.34 29.73
27.98
25.14 24.69
22.12 21.37 20.93 20.86 20.31
14.61 12.71 11.59 11.05

12

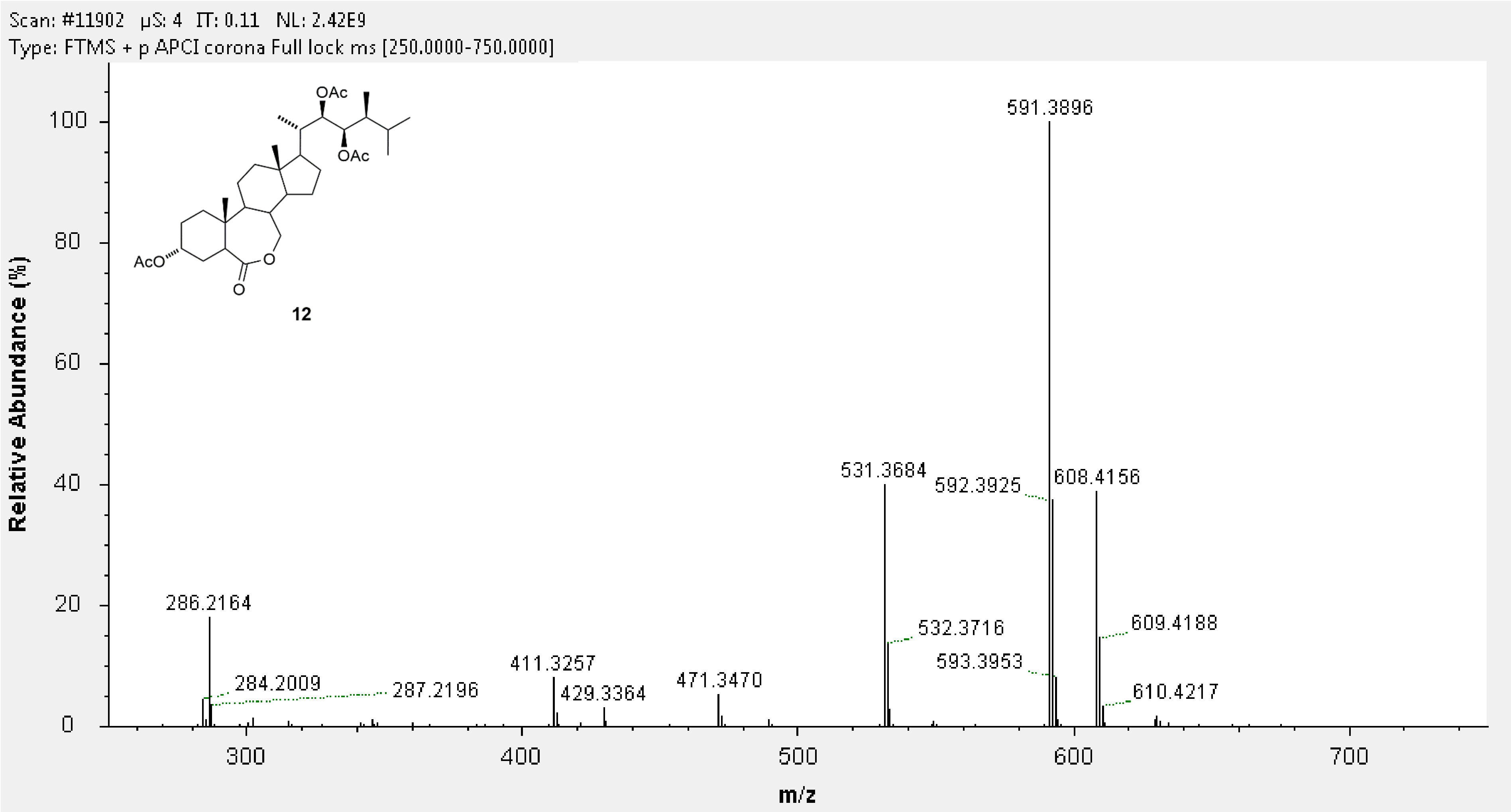
Scan: #11902 µS: 4 IT: 0.11 NL: 2.42E9
Type: FTMS + p APCI corona Full lock ms [250.0000-750.0000]
OAc
OAc
AcO
O
O
O
12
591.3896
531.3684
592.3925
608.4156
286.2164
532.3716
609.4188
593.3953
610.4217
411.3257
429.3364
471.3470
284.2009
287.2196
Relative Abundance (%)
m/z
0
20
40
60
80
100
300
400
500
600
700

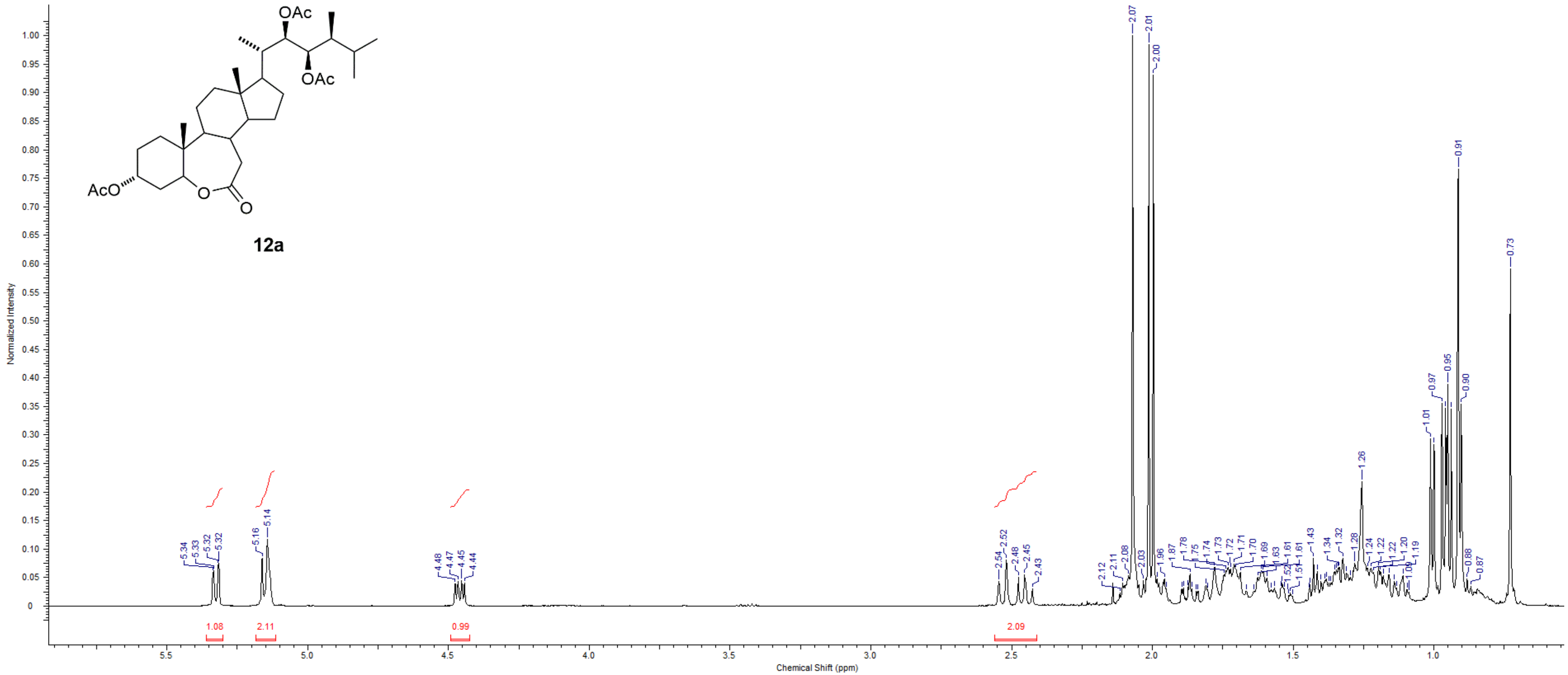
OAc
OAc
AcO
O
O
12a
Normalized Intensity
Chemical Shift (ppm)
1.08
2.11
0.99
2.09

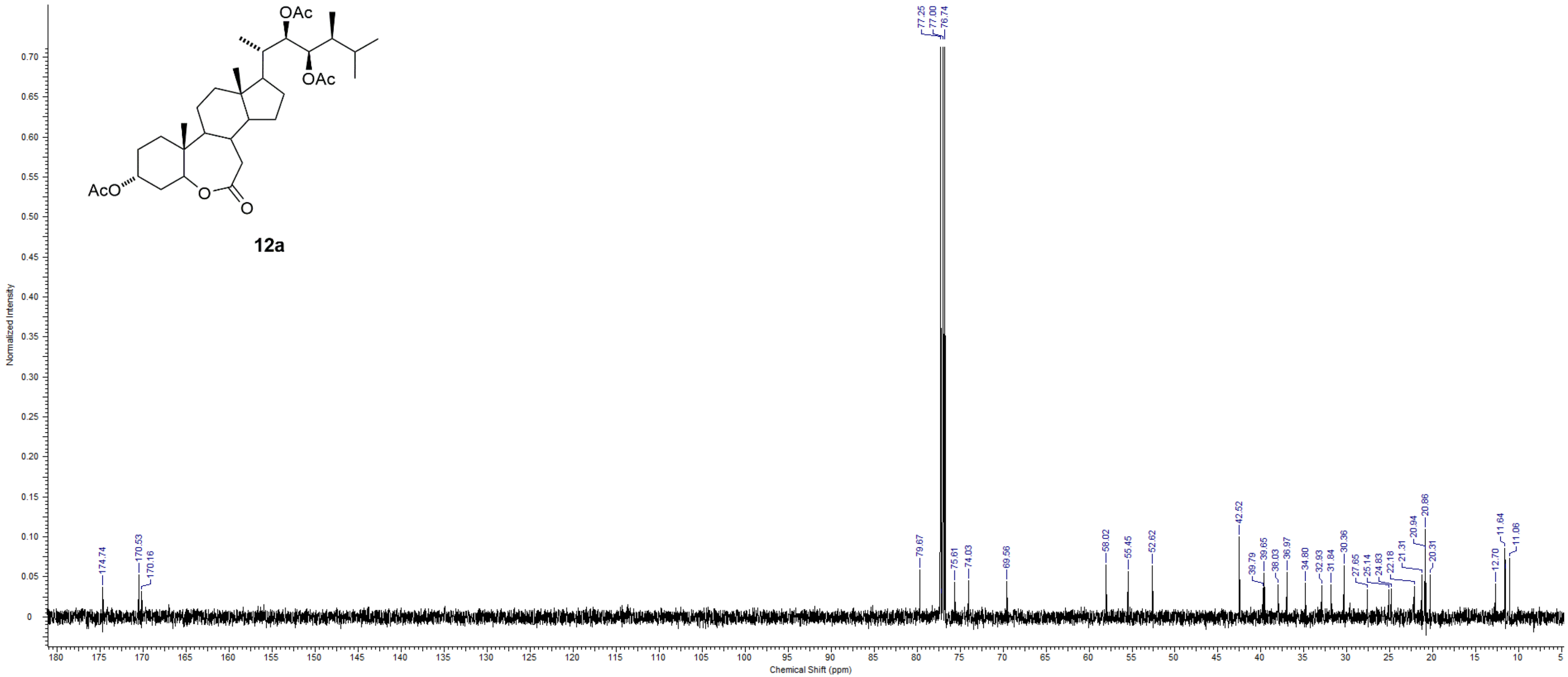
OAc
OAc
AcO
O
O
12a
Normalized Intensity
Chemical Shift (ppm)
77.25
77.00
76.74
174.74
170.53
170.16
79.67
75.61
74.03
69.56
58.02
55.45
52.62
42.52
39.79
39.65
38.03
36.97
34.80
32.93
31.84
30.36
27.65
25.14
24.83
22.18
21.31
20.94
20.86
20.31
12.70
11.64
11.06

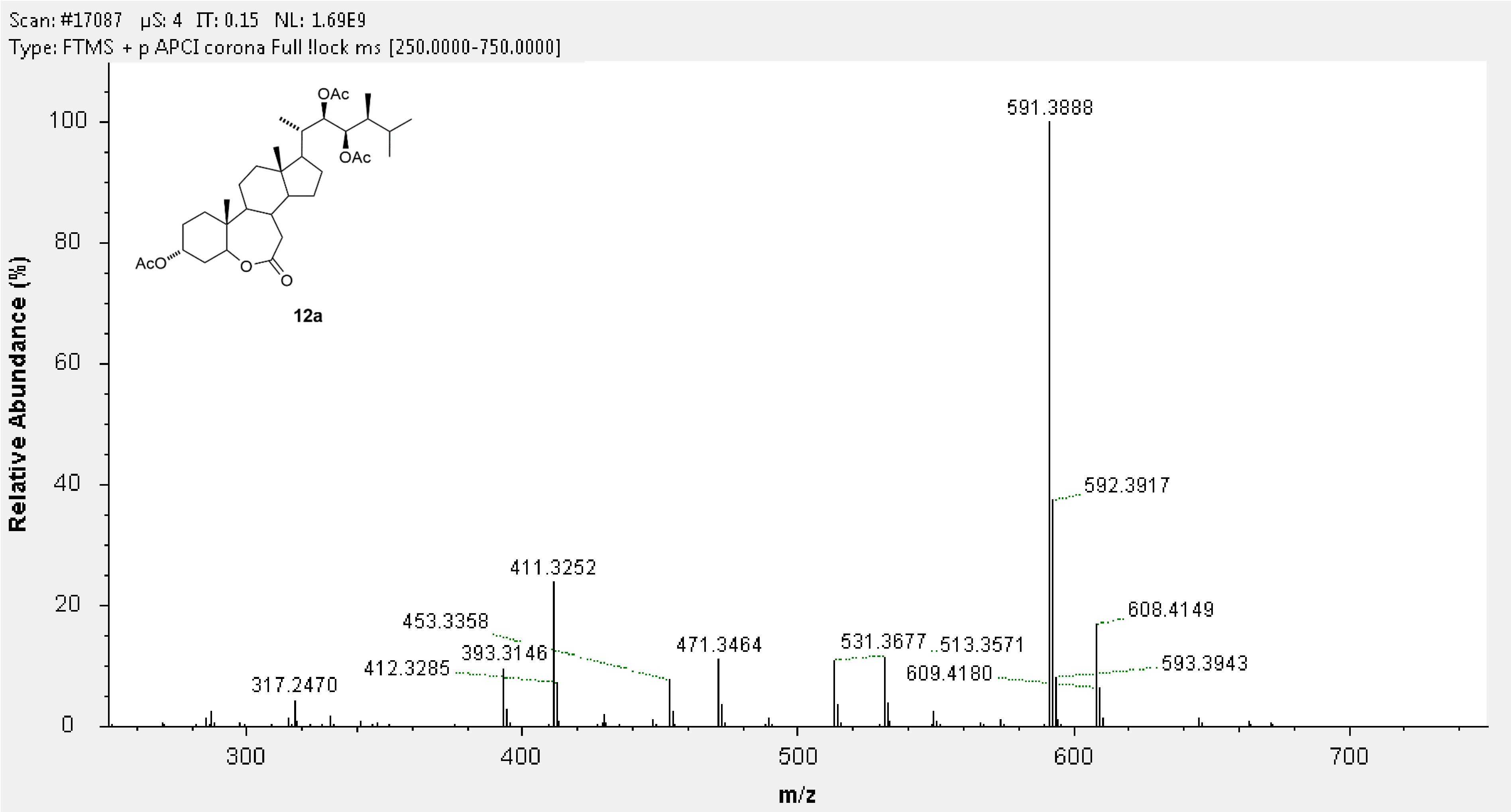

Scan: #17087 µS: 4 IT: 0.15 NL: 1.69E9
Type: FTMS + p APCI corona Full !lock ms [250.0000-750.0000]
OAc
OAc
AcO
O
O
12a
591.3888
592.3917
411.3252
608.4149
453.3358
471.3464
531.3677
513.3571
393.3146
593.3943
412.3285
609.4180
317.2470
Relative Abundance (%)
m/z

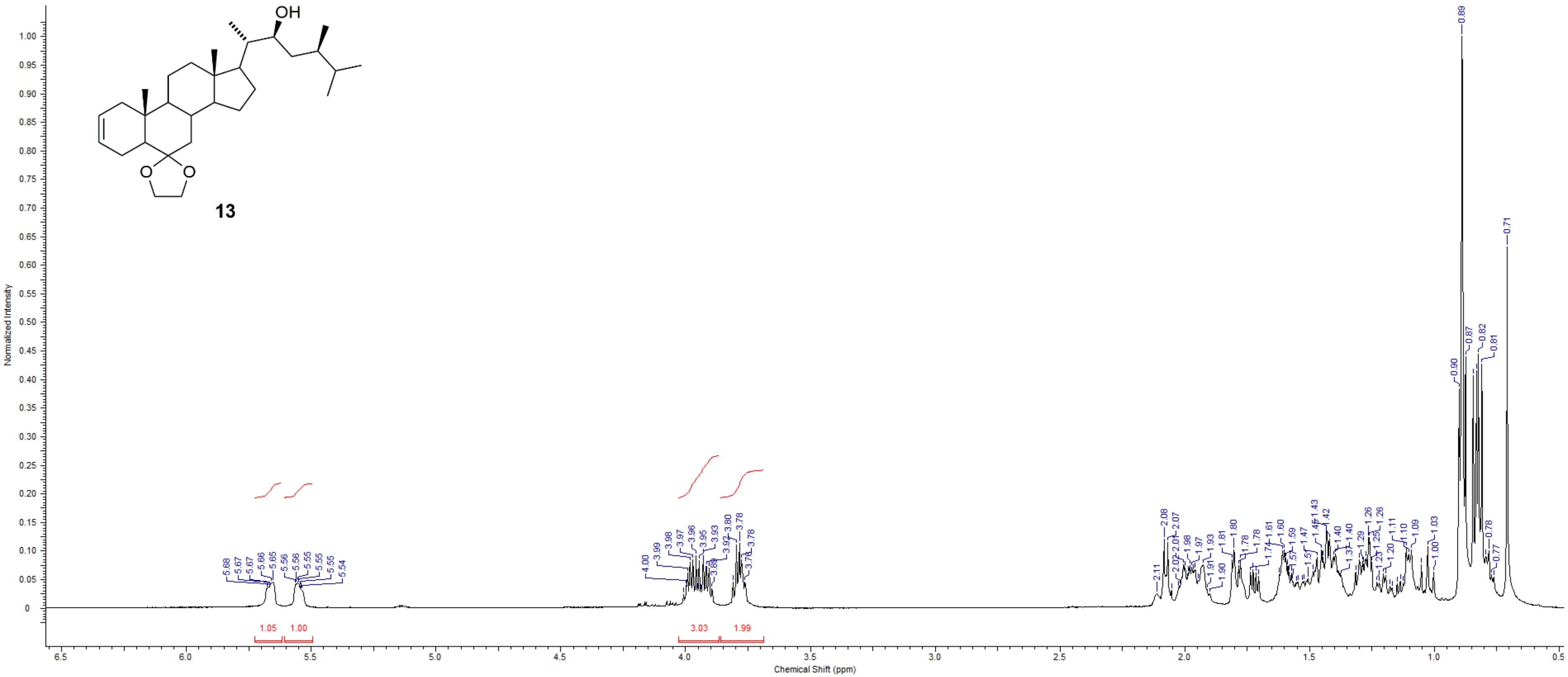
OH
13
Normalized Intensity
Chemical Shift (ppm)
1.05
1.00
3.03
1.99

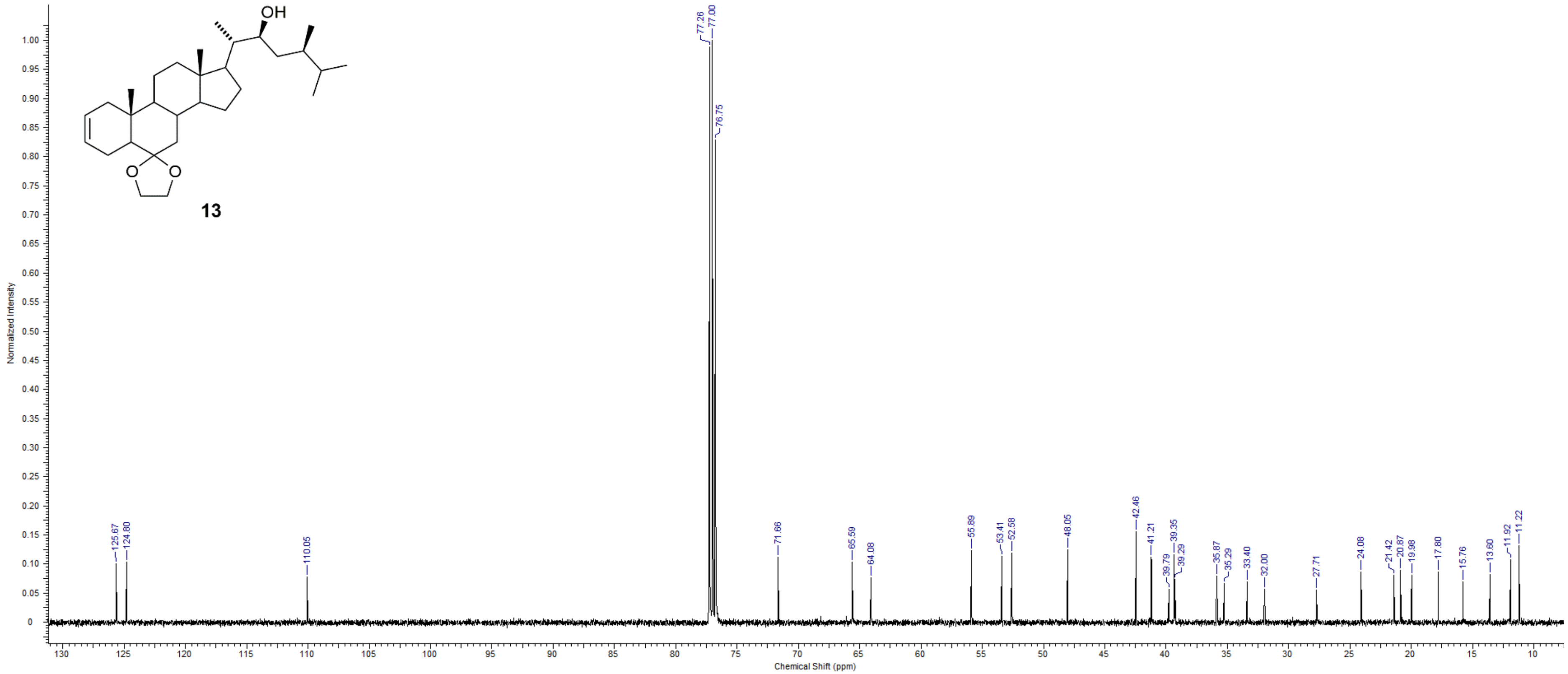
OH
O
O
13
Normalized Intensity
Chemical Shift (ppm)
77.26
77.00
76.75
125.67
124.80
110.05
71.66
65.59
64.08
55.89
53.41
52.58
48.05
42.46
41.21
39.79
39.35
39.29
35.87
35.29
33.40
32.00
27.71
24.08
21.42
20.87
19.98
17.80
15.76
13.60
11.92
11.22

Single Mass Analysis
Tolerance = 1.0 PPM / DBE: min = -1.5, max = 50.0
Element prediction: Off
Number of isotope peaks used for i-FIT = 3
Monoisotopic Mass, Even Electron Ions
608 formula(e) evaluated with 1 results within limits (all results (up to 1000) for each mass)
Elements Used:

Mass	Calc. Mass	mDa	PPM	DBE	Formula	i-FIT	i-FIT Norm	Fit Conf %	C	H	N	O
459.3834	459.3838	-0.4	-0.9	5.5	C30 H51 O3	709.8	n/a	n/a	30	51		3

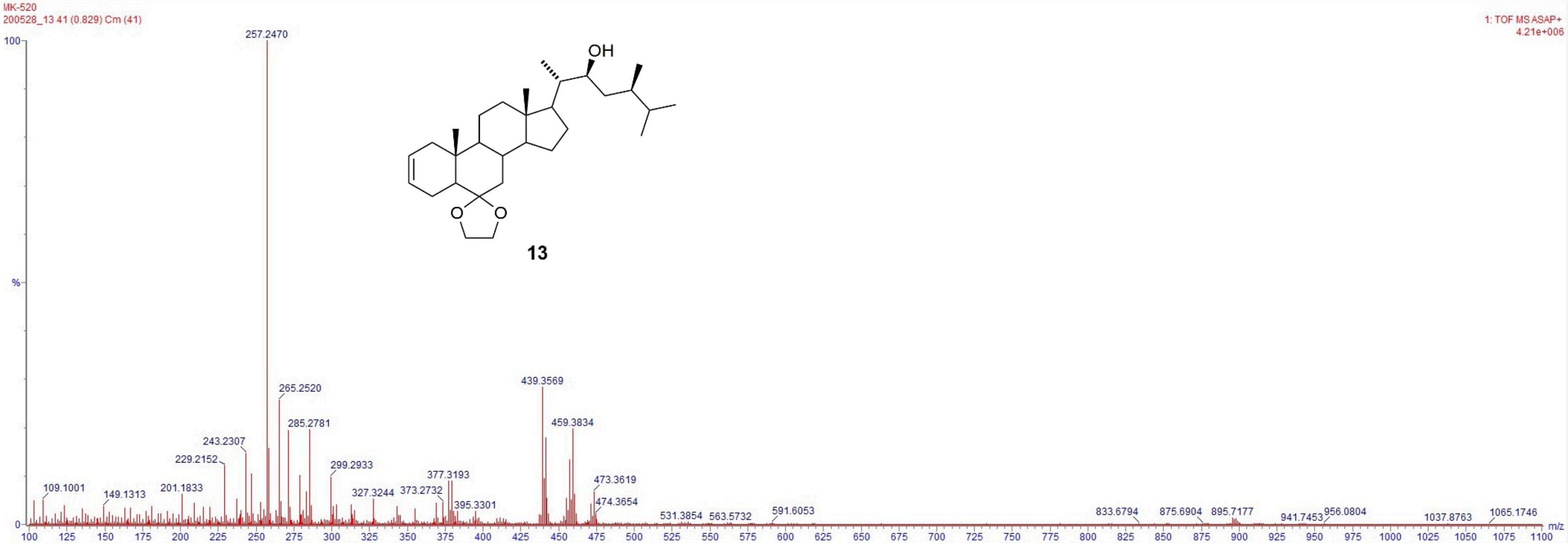

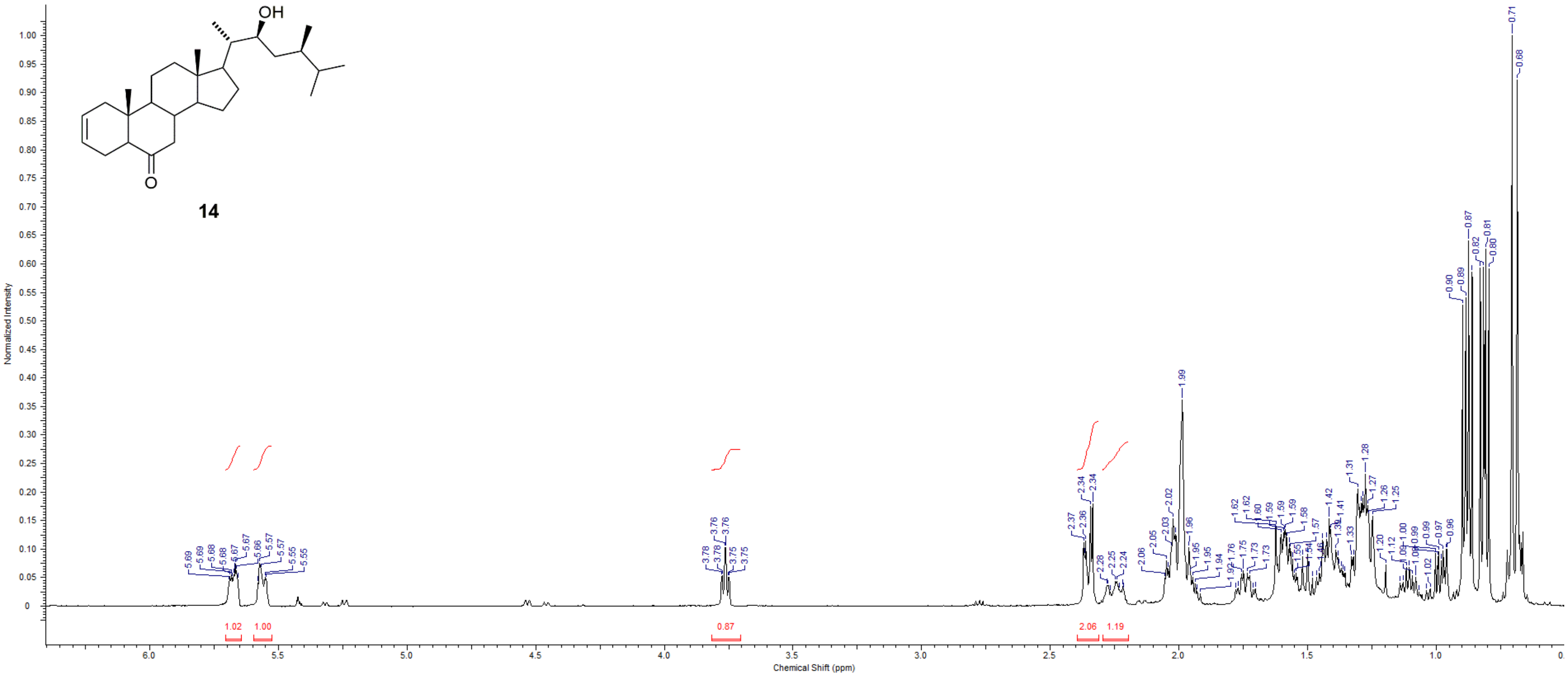
OH
O
14
Normalized Intensity
Chemical Shift (ppm)
1.02
1.00
0.87
2.06
1.19
6.0
5.5
5.0
4.5
4.0
3.5
3.0
2.5
2.0
1.5
1.0

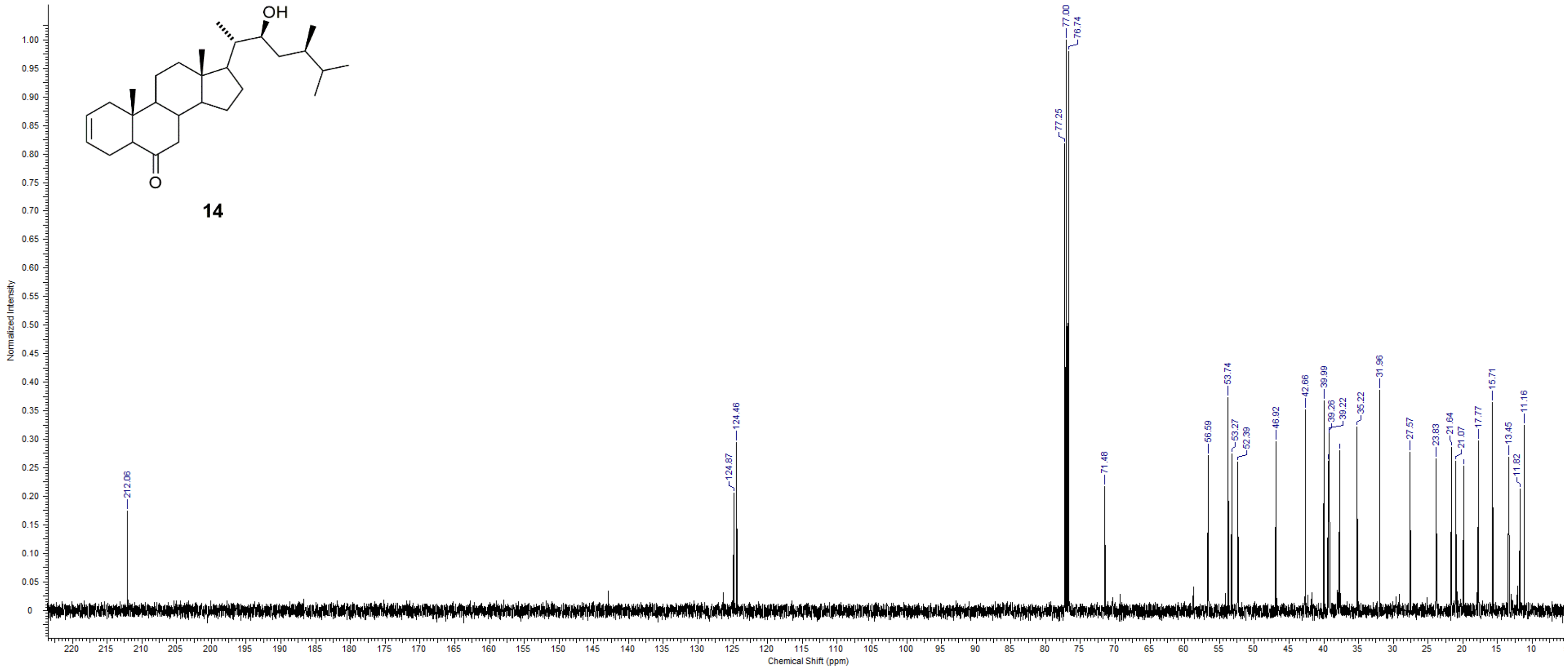
OH
O
14
212.06
124.87
124.46
77.25
77.00
76.74
71.48
56.59
53.74
53.27
52.39
46.92
42.66
39.99
39.26
39.22
35.22
31.96
27.57
23.83
21.64
21.07
17.77
15.71
13.45
11.82
11.16
Normalized Intensity
Chemical Shift (ppm)

Single Mass Analysis
Tolerance = 1.0 PPM / DBE: min = -1.5, max = 50.0
Element prediction: Off
Number of isotope peaks used for i-FIT = 3
Monoisotopic Mass, Even Electron Ions
509 formula(e) evaluated with 1 results within limits (all results (up to 1000) for each mass)
Elements Used:

Mass	Calc. Mass	mDa	PPM	DBE	Formula	i-FIT	i-FIT Norm	Fit Conf %	C	H	N	O
415.3578	415.3576	0.2	0.5	5.5	C28 H47 O2	1259.3	n/a	n/a	28	47		2

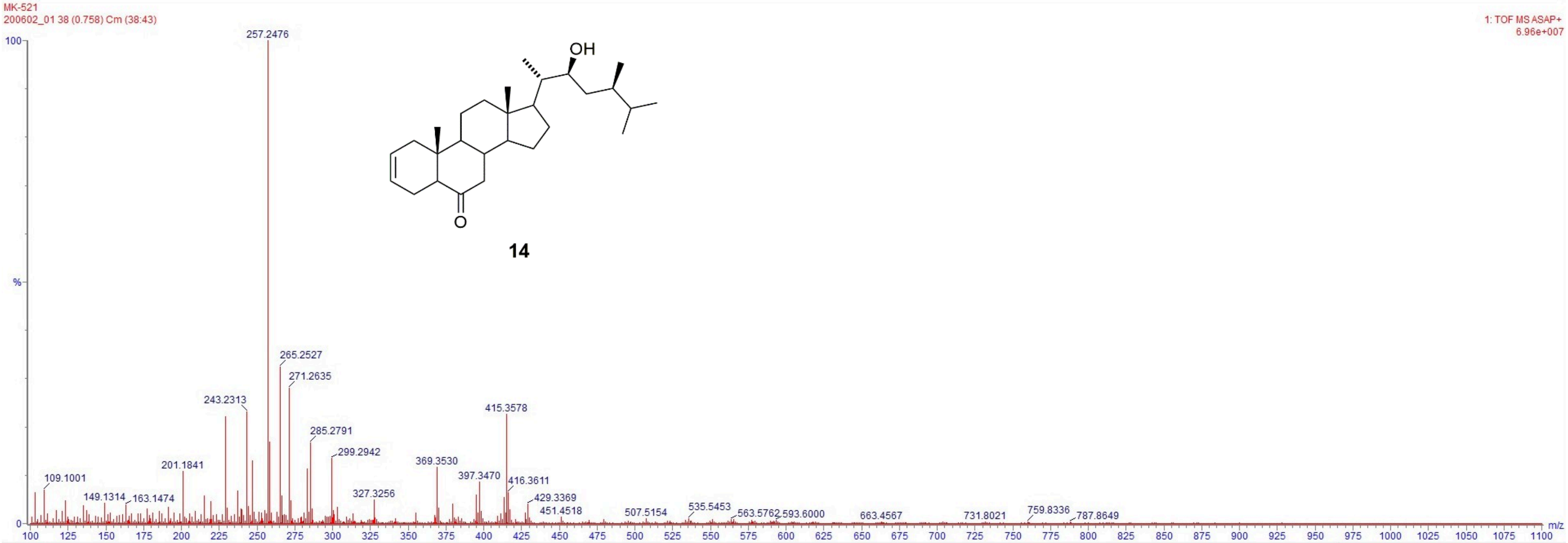

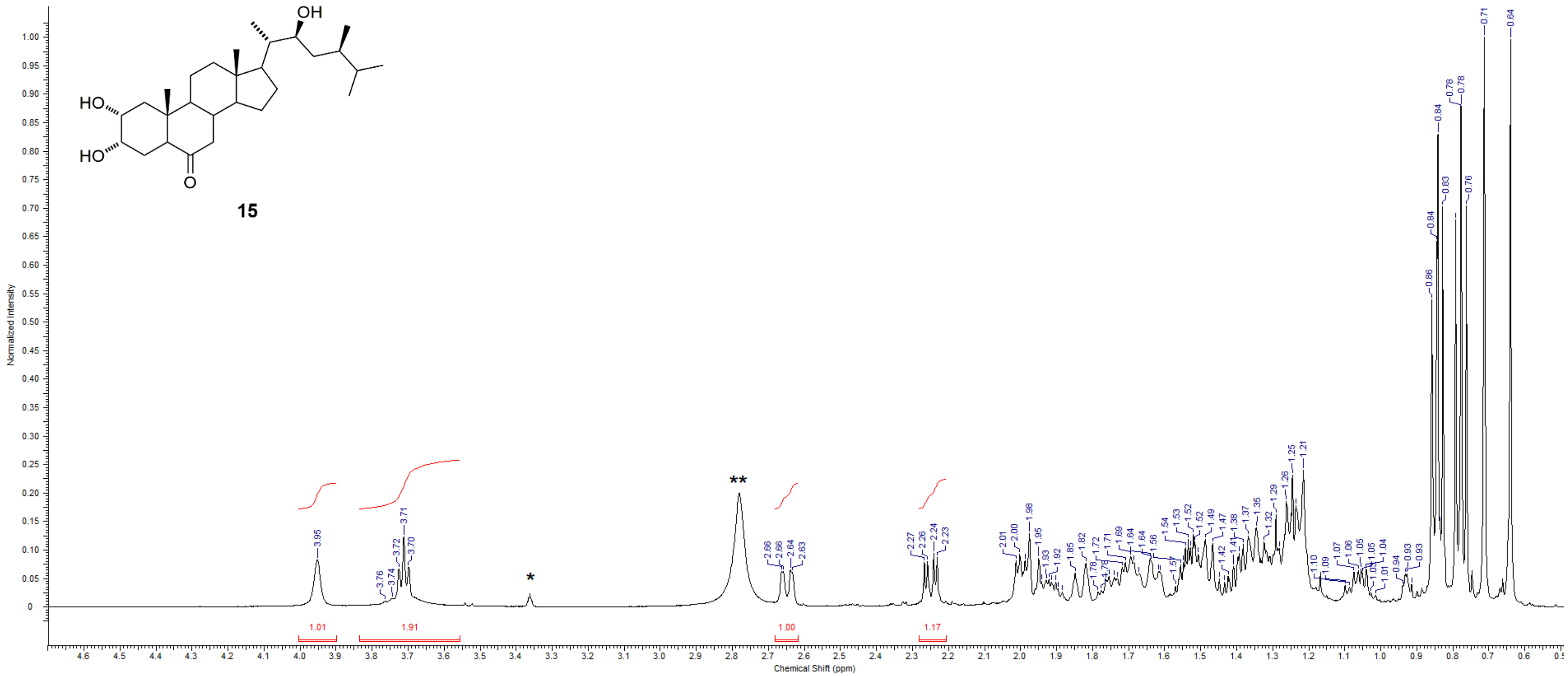

* residual CHD_2OD, ** water signal

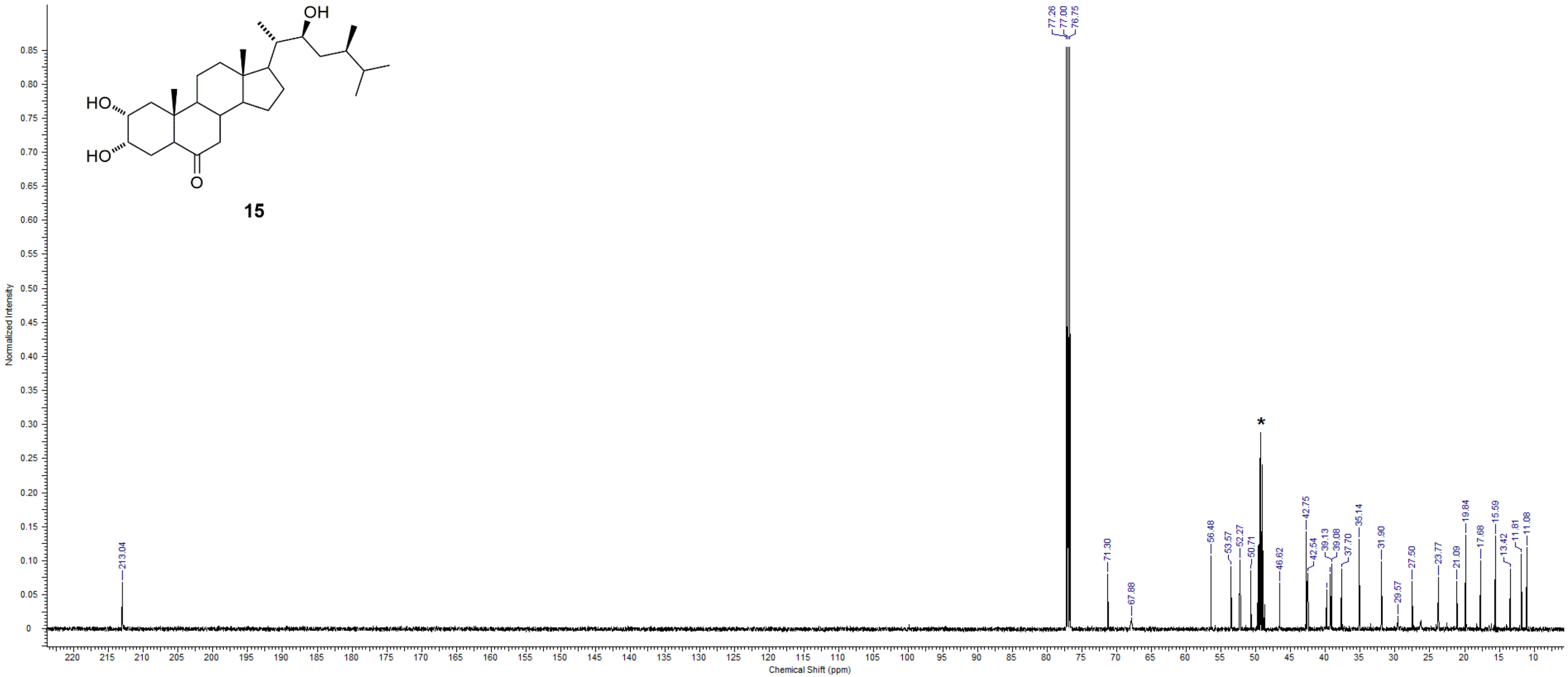

* signal of CD_3OD

Single Mass Analysis
Tolerance = 1.0 PPM / DBE: min = -1.5, max = 50.0
Element prediction: Off
Number of isotope peaks used for i-FIT = 3
Monoisotopic Mass, Even Electron Ions
588 formula(e) evaluated with 1 results within limits (all results (up to 1000) for each mass)
Elements Used:

Mass	Calc. Mass	mDa	PPM	DBE	Formula	i-FIT	i-FIT Norm	Fit Conf %	C	H	N	O
449.3633	449.3631	0.2	0.4	4.5	C28 H49 O4	1350.8	n/a	n/a	28	49		4

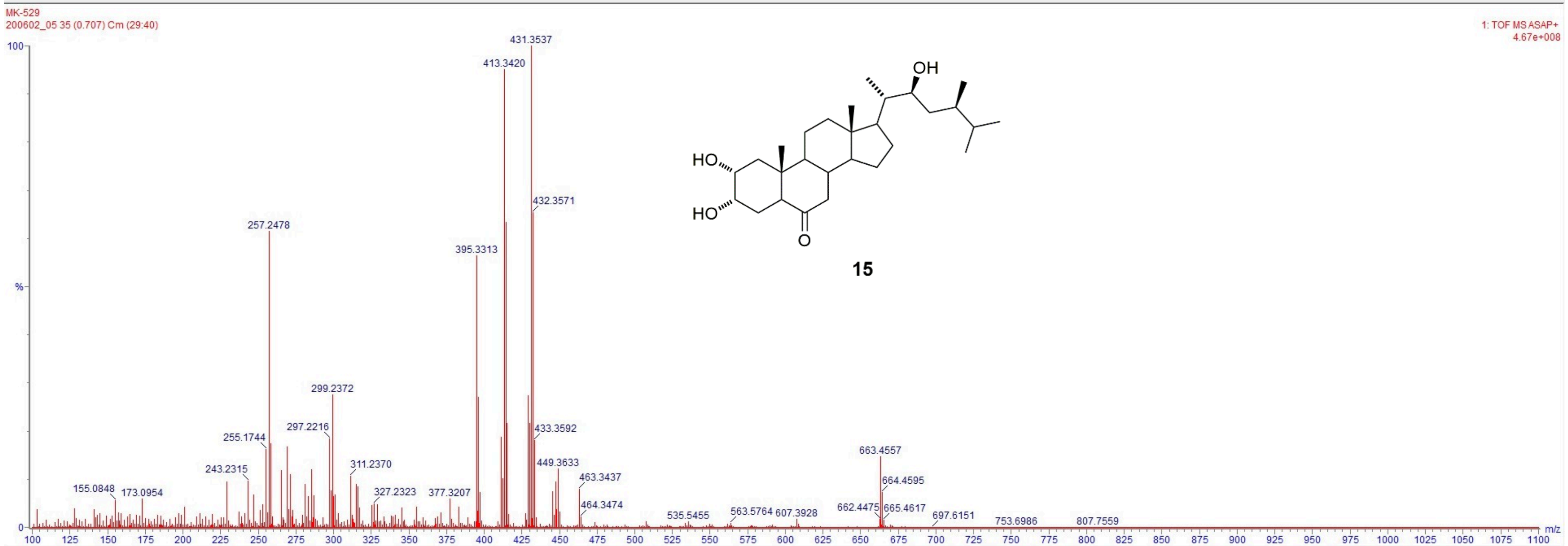

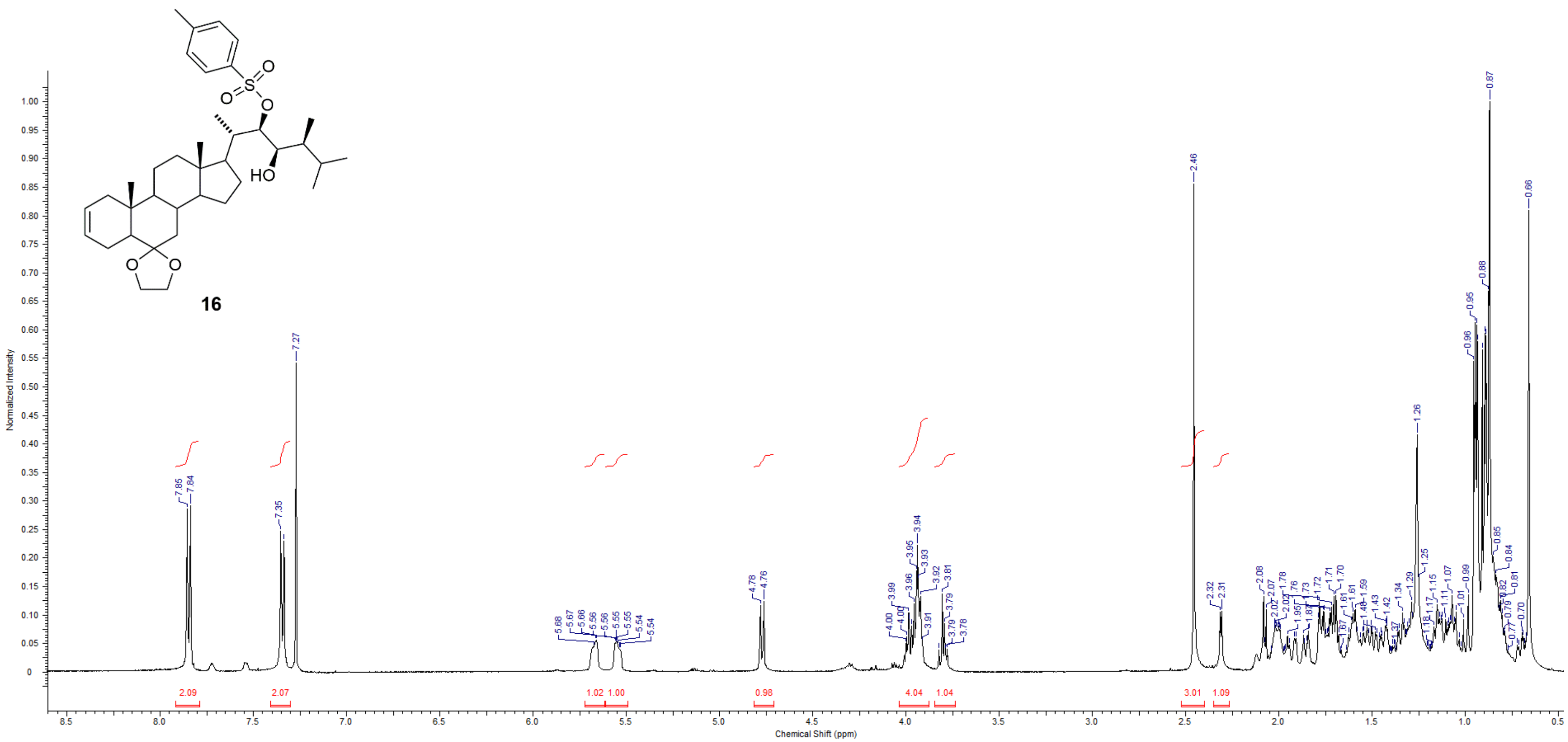
16
Normalized Intensity
Chemical Shift (ppm)

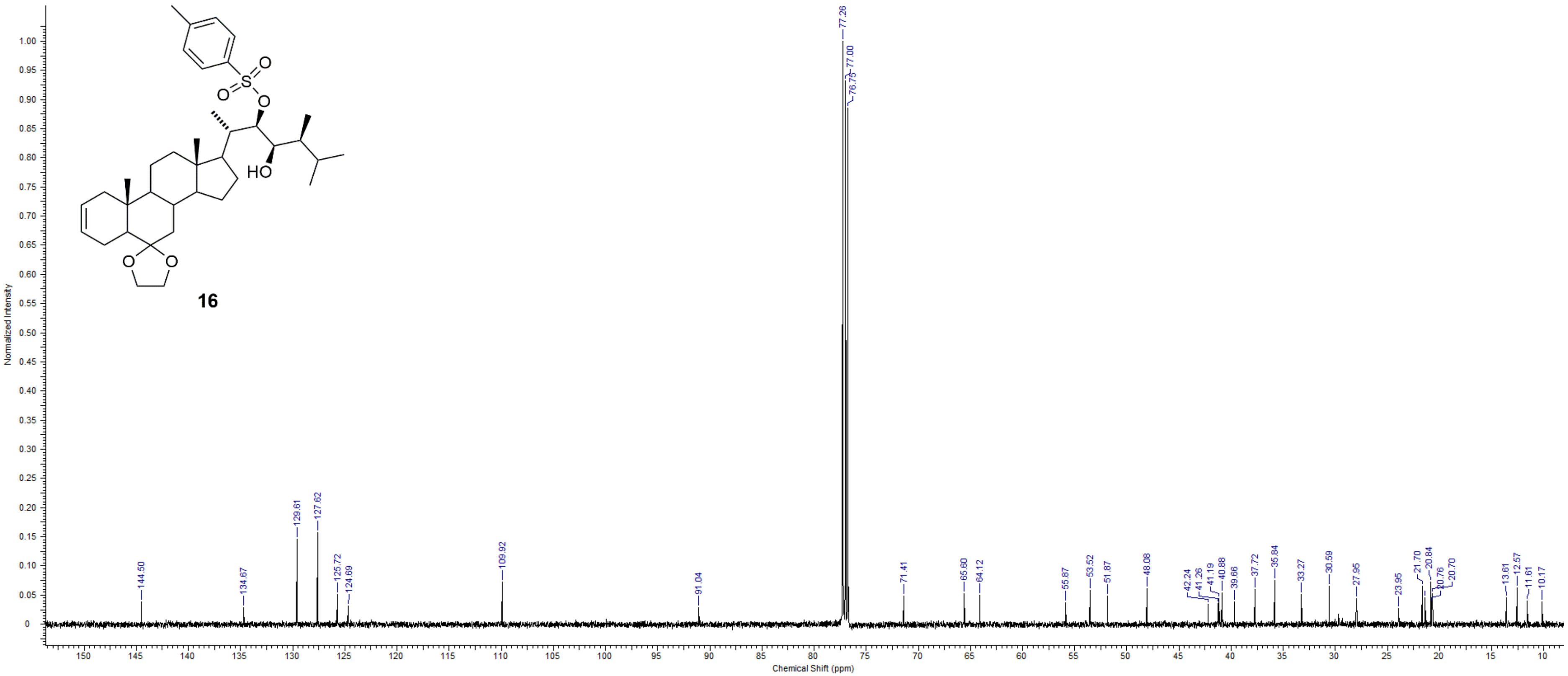
16
Normalized Intensity
Chemical Shift (ppm)
144.50
134.67
129.61
127.62
125.72
124.69
109.92
91.04
77.26
77.00
76.75
71.41
65.60
64.12
55.87
53.52
51.87
48.08
42.24
41.26
41.19
40.88
39.66
37.72
35.84
33.27
30.59
27.95
23.95
21.70
20.84
20.76
20.70
13.61
12.57
11.61
10.17

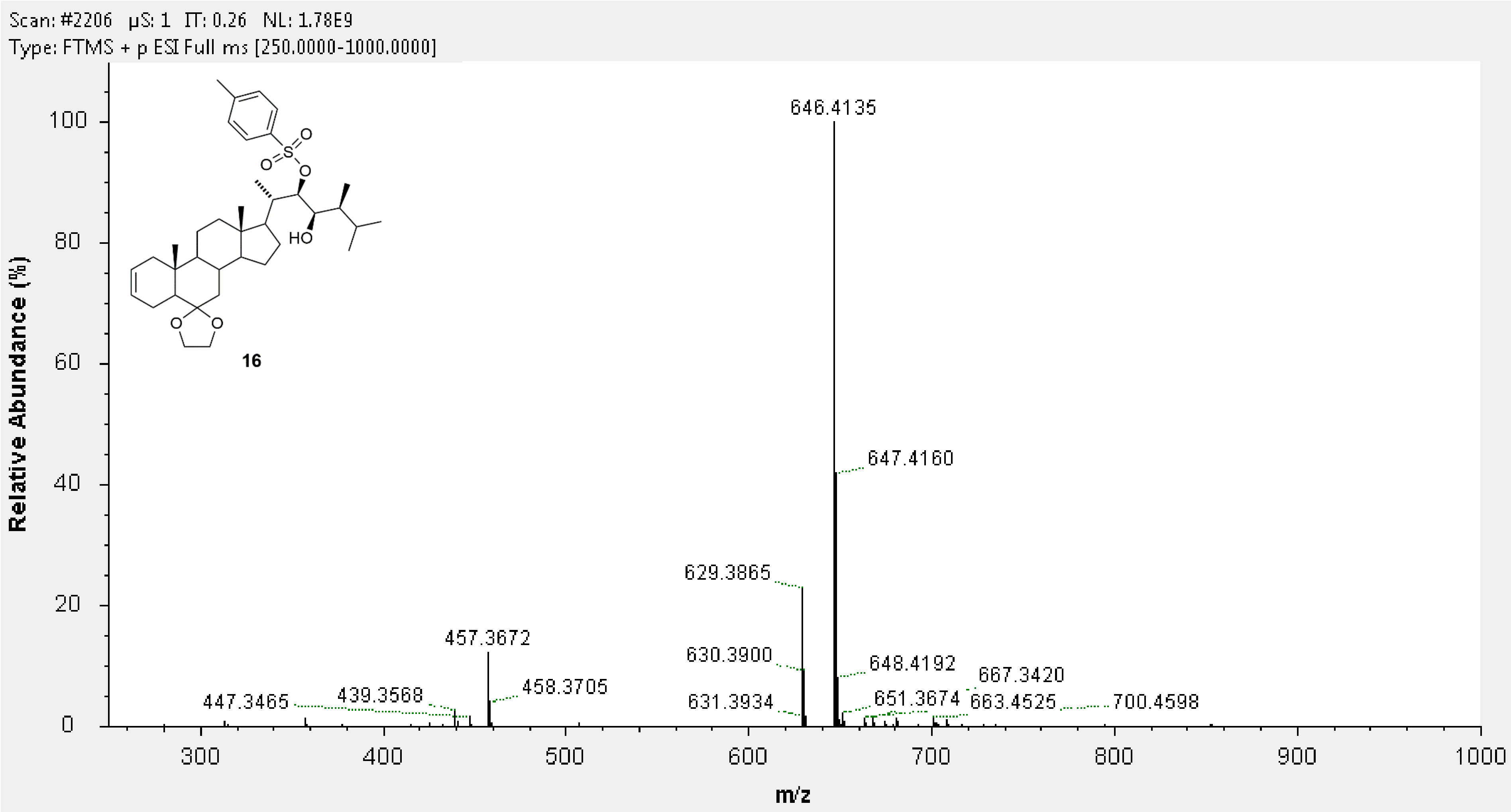
Scan: #2206 µS: 1 IT: 0.26 NL: 1.78E9
Type: FTMS + p ESI Full ms [250.0000-1000.0000]
Relative Abundance (%)
m/z
16
646.4135
647.4160
629.3865
630.3900
631.3934
648.4192
667.3420
651.3674
663.4525
700.4598
457.3672
458.3705
447.3465
439.3568

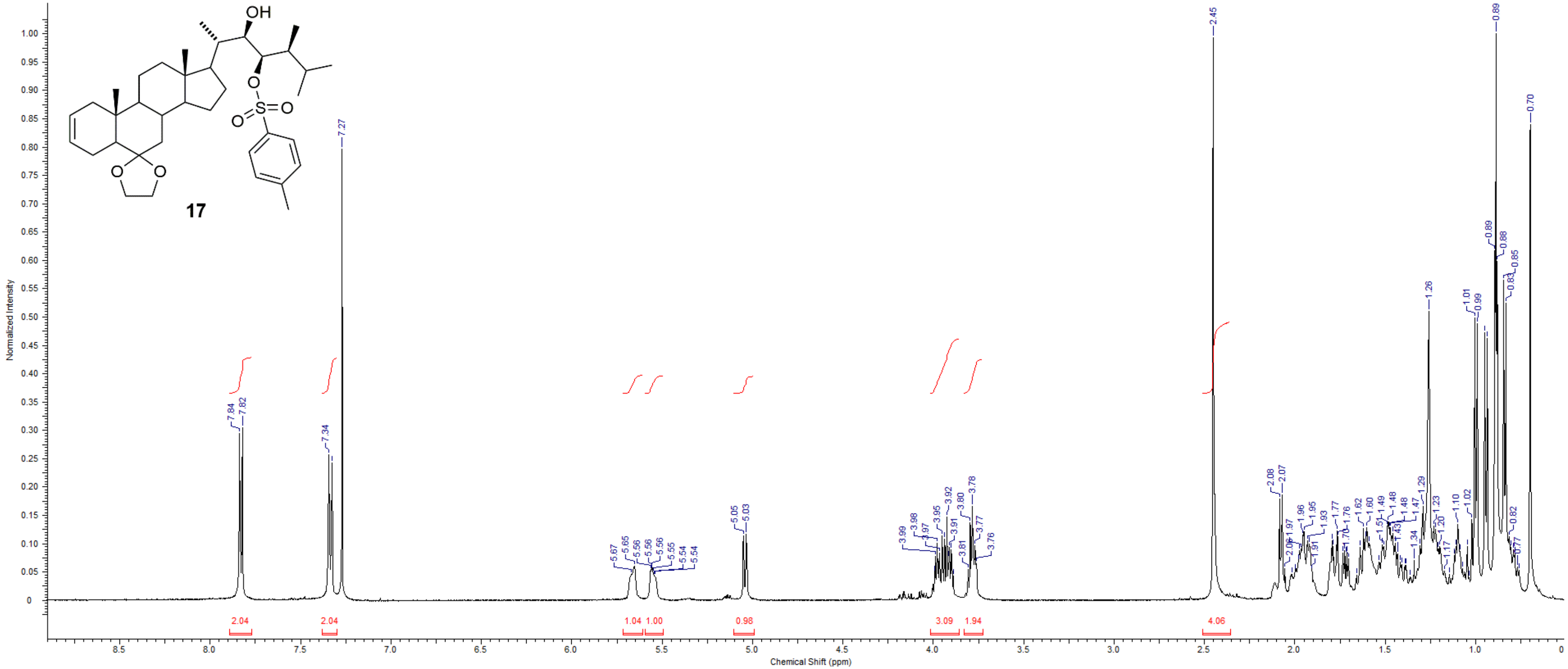
17
Normalized Intensity
Chemical Shift (ppm)

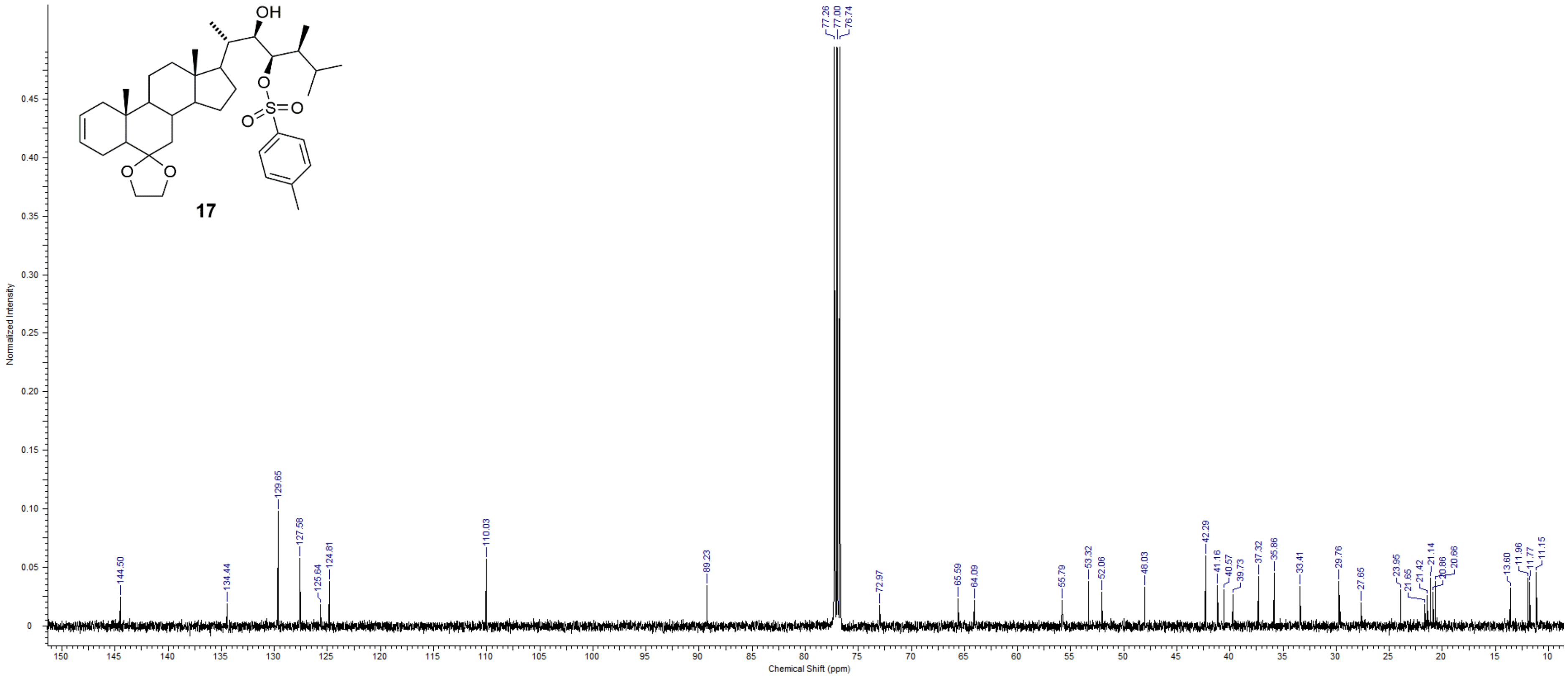

OH
O
S
O
O
O
O
17
77.26
77.00
76.74
144.50
134.44
129.65
127.58
125.64
124.81
110.03
89.23
72.97
65.59
64.09
55.79
53.32
52.06
48.03
42.29
41.16
40.57
39.73
37.32
35.86
33.41
29.76
27.65
23.95
21.65
21.42
21.14
20.86
20.66
13.60
11.96
11.77
11.15
Normalized Intensity
Chemical Shift (ppm)

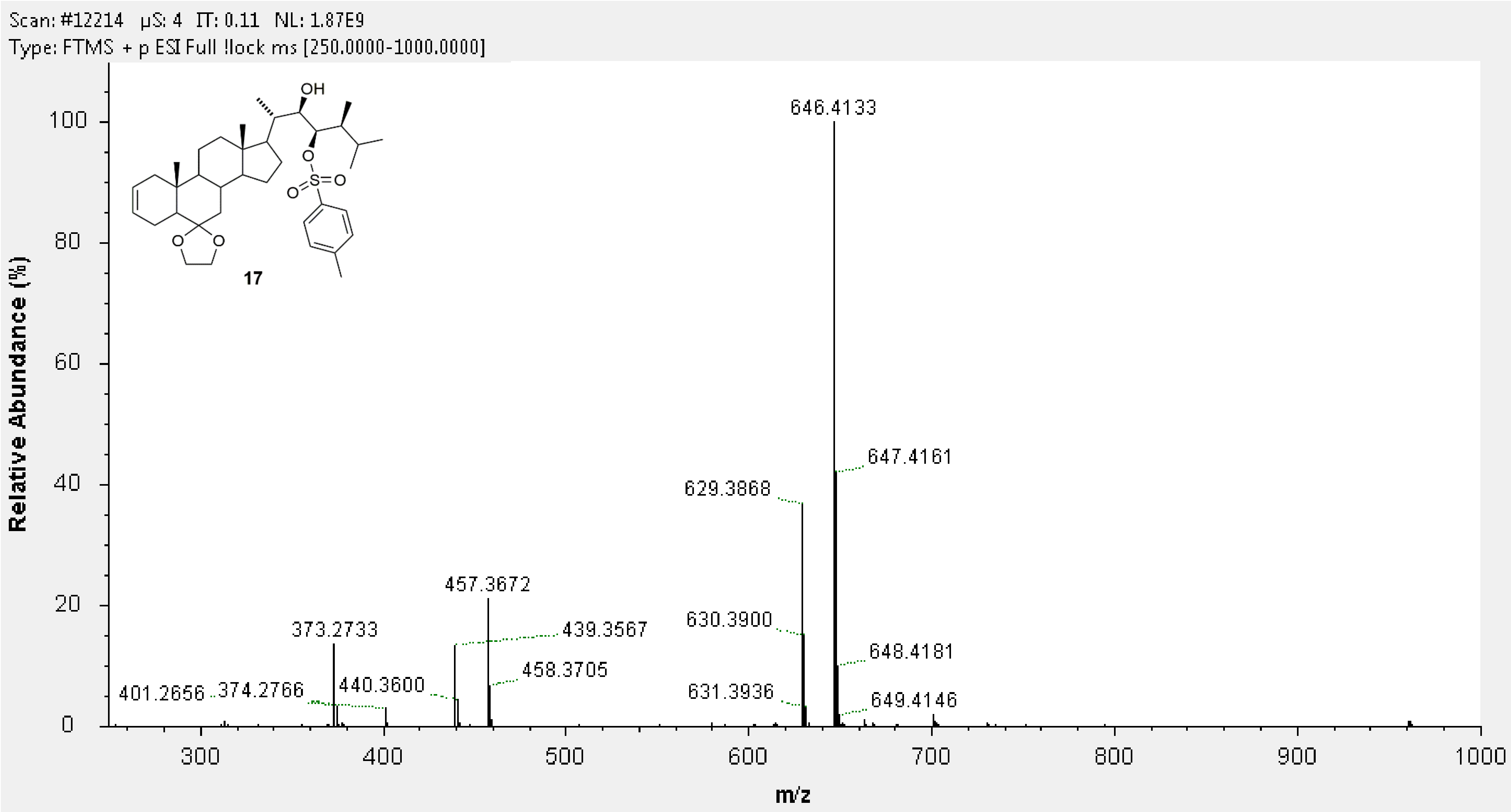
Scan: #12214 µS: 4 IT: 0.11 NL: 1.87E9
Type: FTMS + p ESI Full !lock ms [250.0000-1000.0000]
OH
O
O=S=O
O
O
17
Relative Abundance (%)
m/z
646.4133
647.4161
629.3868
457.3672
373.2733
439.3567
630.3900
648.4181
458.3705
440.3600
401.2656
374.2766
631.3936
649.4146
0
20
40
60
80
100
300
400
500
600
700
800
900
1000

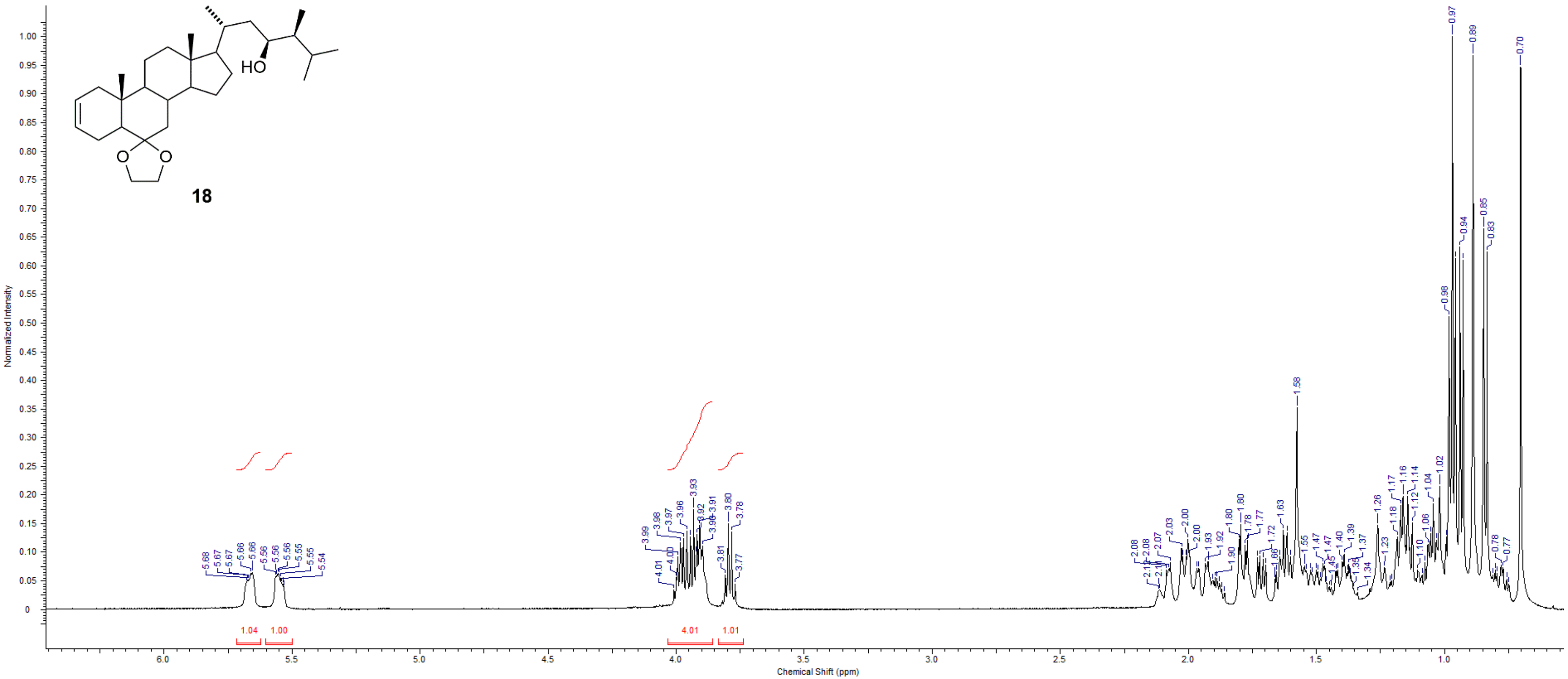
HO
O
O
18
Normalized Intensity
Chemical Shift (ppm)
1.04
1.00
4.01
1.01

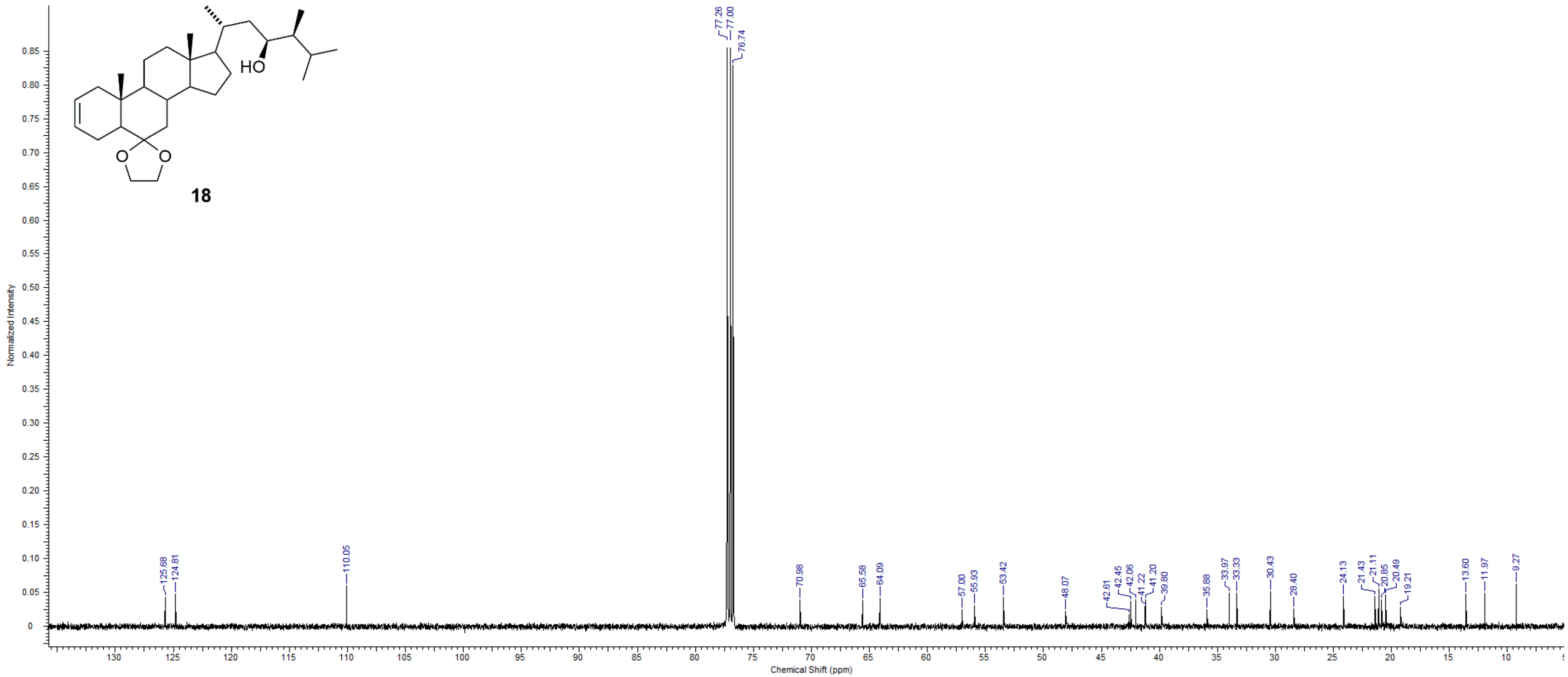
18
HO
O
O
Normalized Intensity
Chemical Shift (ppm)
125.68
124.81
110.05
77.26
77.00
76.74
70.98
65.58
64.09
57.00
55.93
53.42
48.07
42.61
42.45
42.06
41.22
41.20
39.80
35.88
33.97
33.33
30.43
28.40
24.13
21.43
21.11
20.85
20.49
19.21
13.60
11.97
9.27

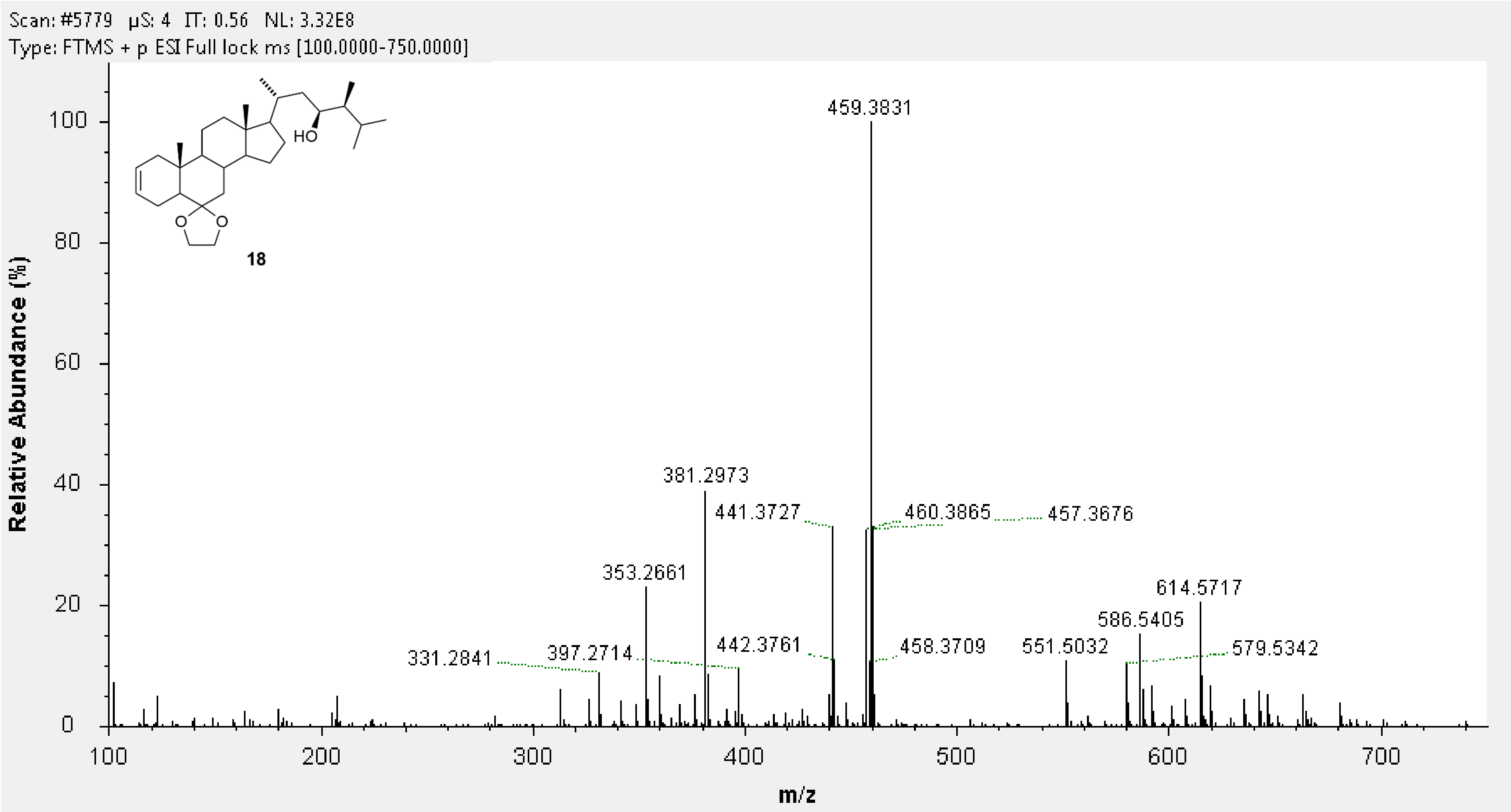
Scan: #5779 µS: 4 IT: 0.56 NL: 3.32E8
Type: FTMS + p ESI Full lock ms [100.0000-750.0000]
Relative Abundance (%)
m/z
HO
O
O
18
459.3831
381.2973
441.3727
460.3865
457.3676
353.2661
614.5717
586.5405
331.2841
397.2714
442.3761
458.3709
551.5032
579.5342

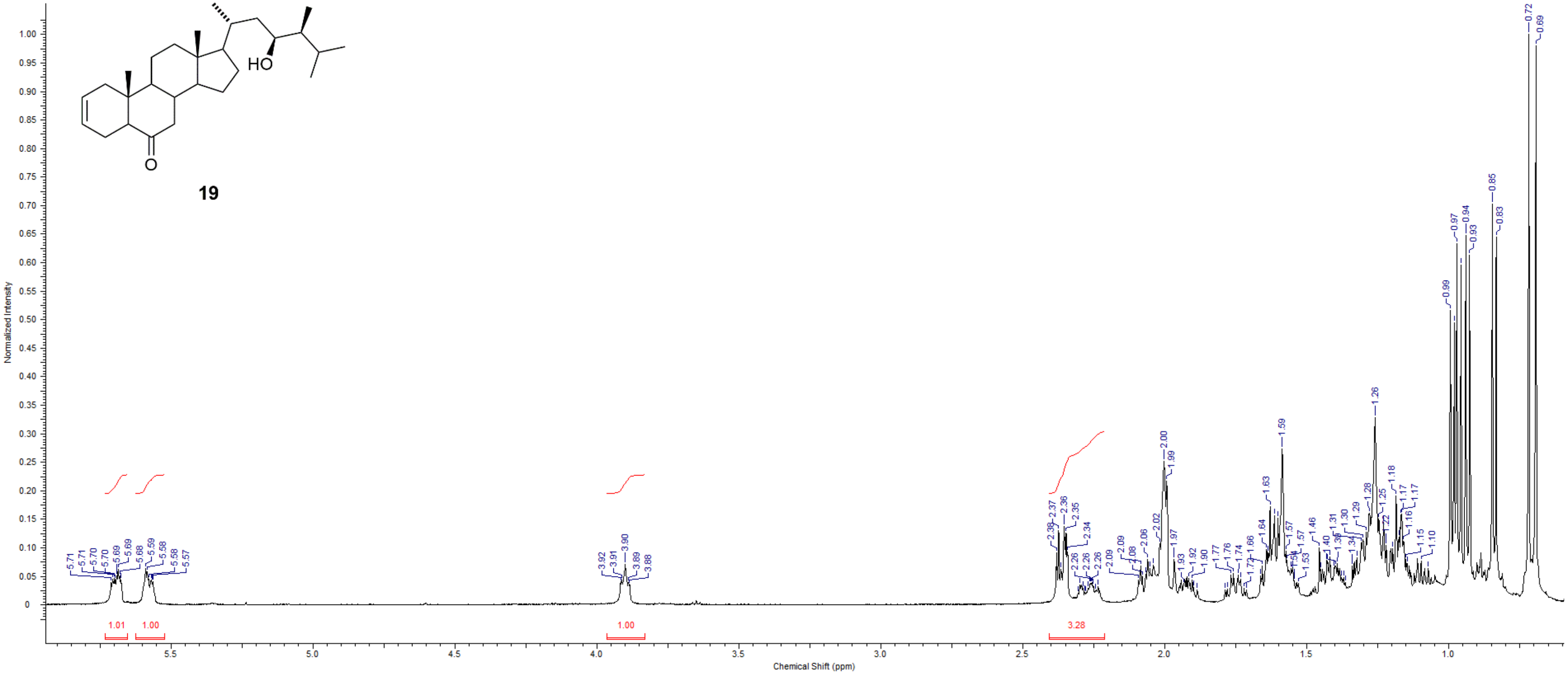
HO
O
19
Normalized Intensity
Chemical Shift (ppm)
1.01
1.00
1.00
3.28

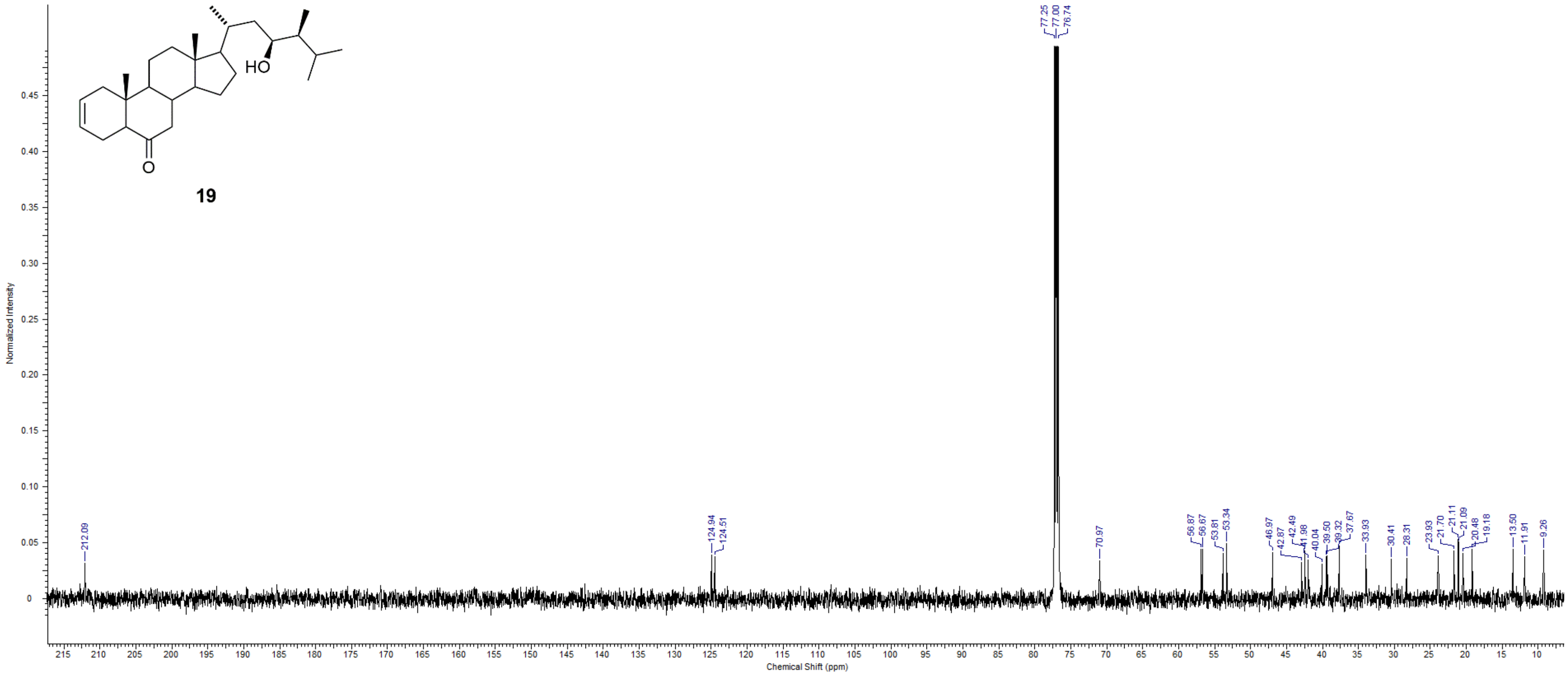

HO
O
Normalized Intensity
Chemical Shift (ppm)
212.09
124.94
124.51
77.25
77.00
76.74
70.97
56.87
56.67
53.81
53.34
46.97
42.87
42.49
41.98
40.04
39.50
39.32
37.67
33.93
30.41
28.31
23.93
21.70
21.11
21.09
20.48
19.18
13.50
11.91
9.26

19

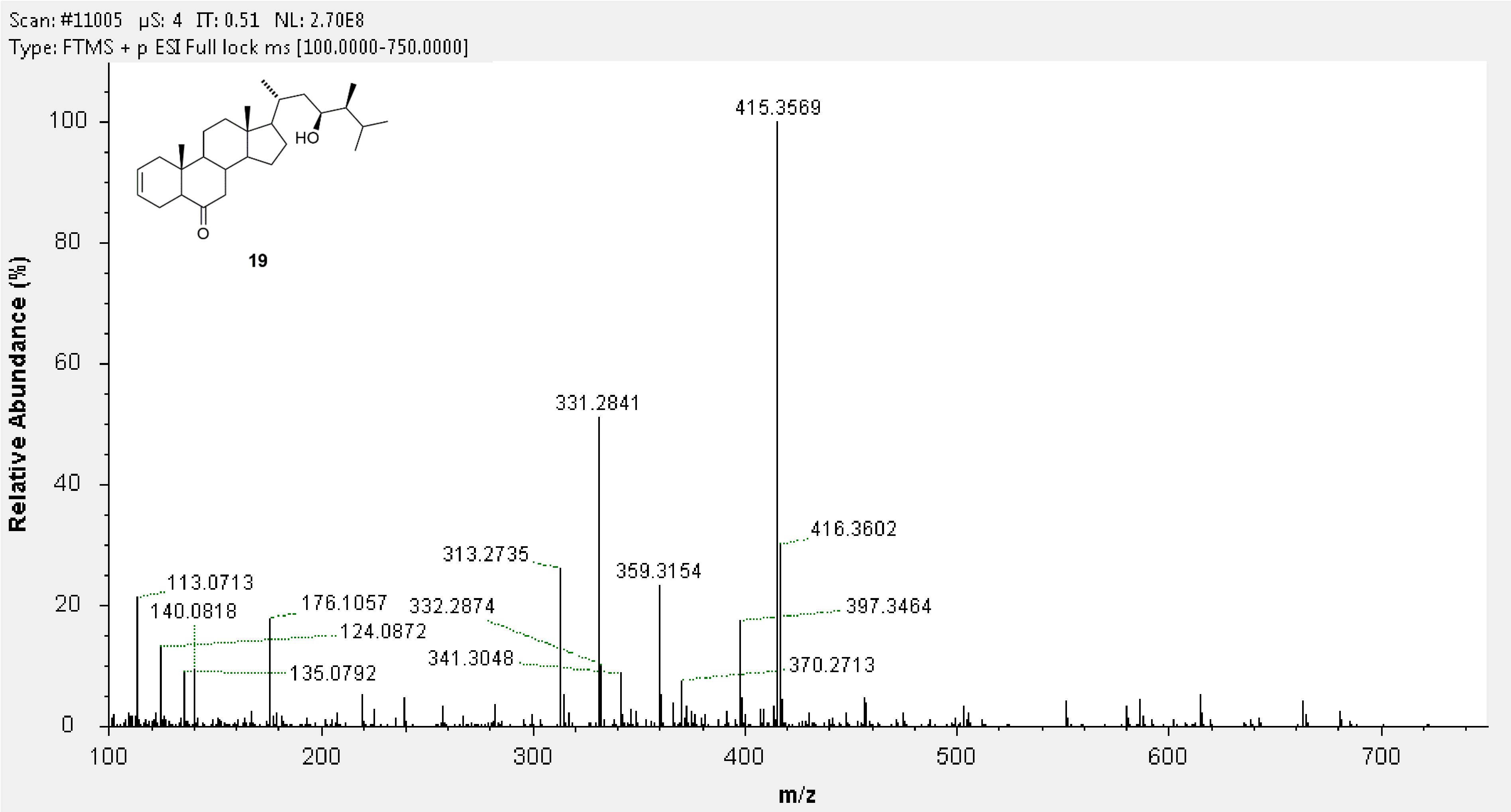

Scan: #11005 µS: 4 IT: 0.51 NL: 2.70E8
Type: FTMS + p ESI Full lock ms [100.0000-750.0000]
HO
O
19
415.3569
331.2841
416.3602
313.2735
359.3154
113.0713
140.0818
176.1057
332.2874
397.3464
124.0872
341.3048
370.2713
135.0792
Relative Abundance (%)
m/z
0
20
40
60
80
100
100
200
300
400
500
600
700

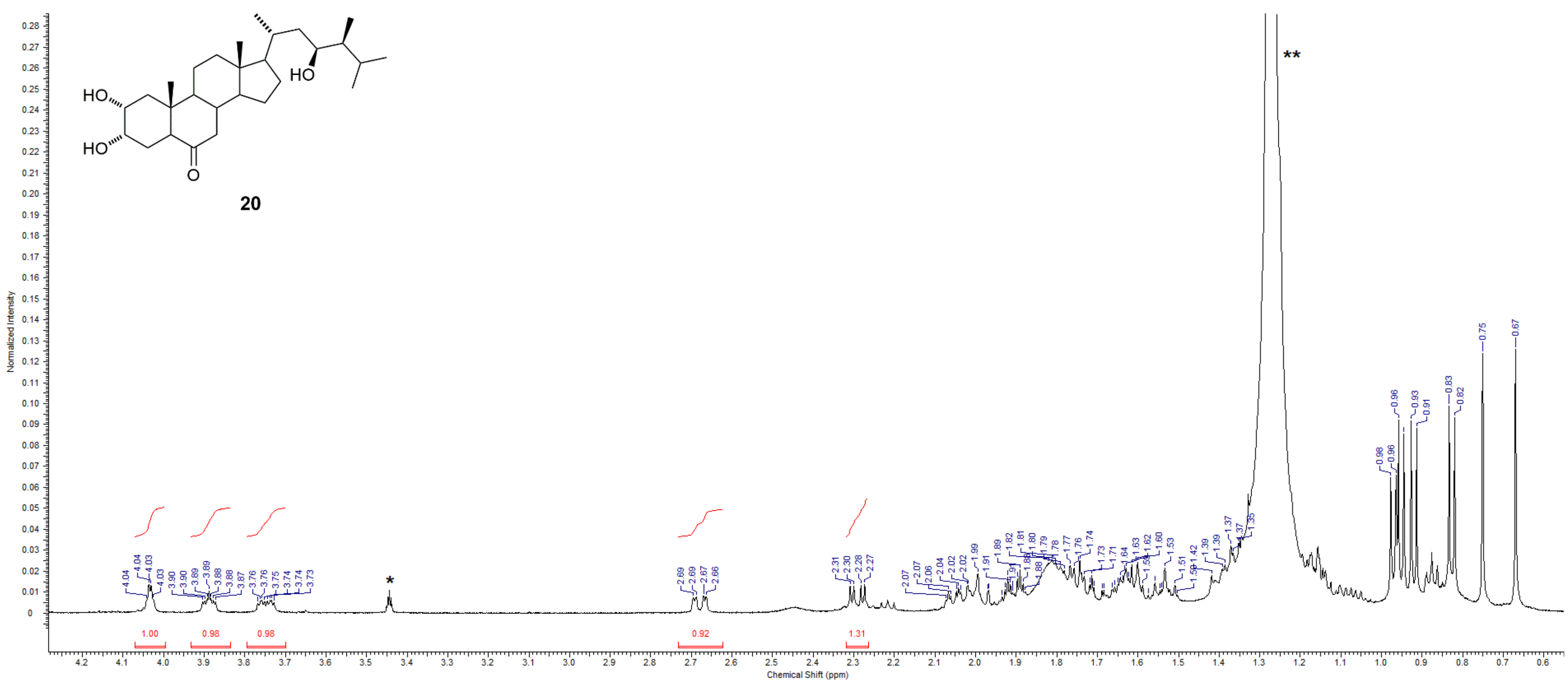

* residual CHD_2OD, ** water signal

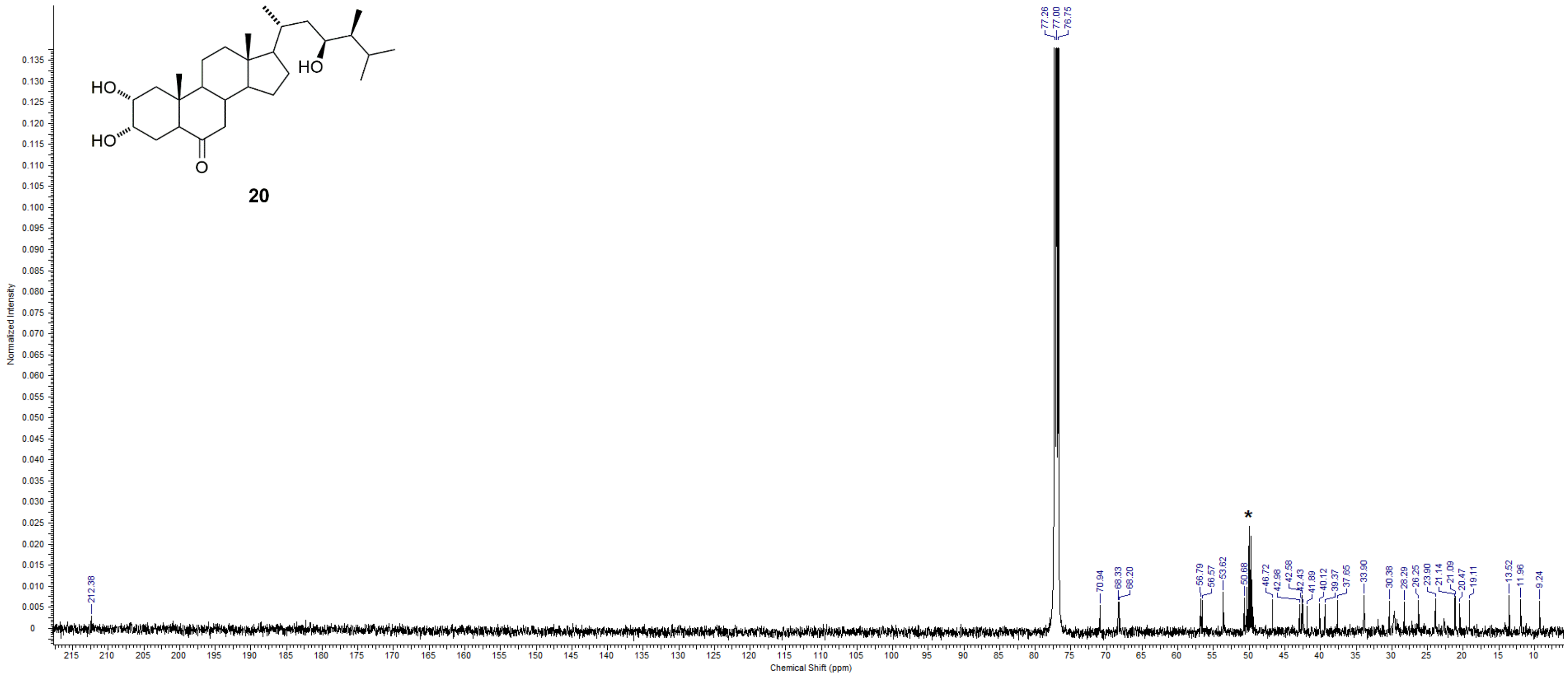

* signal of CD_3OD

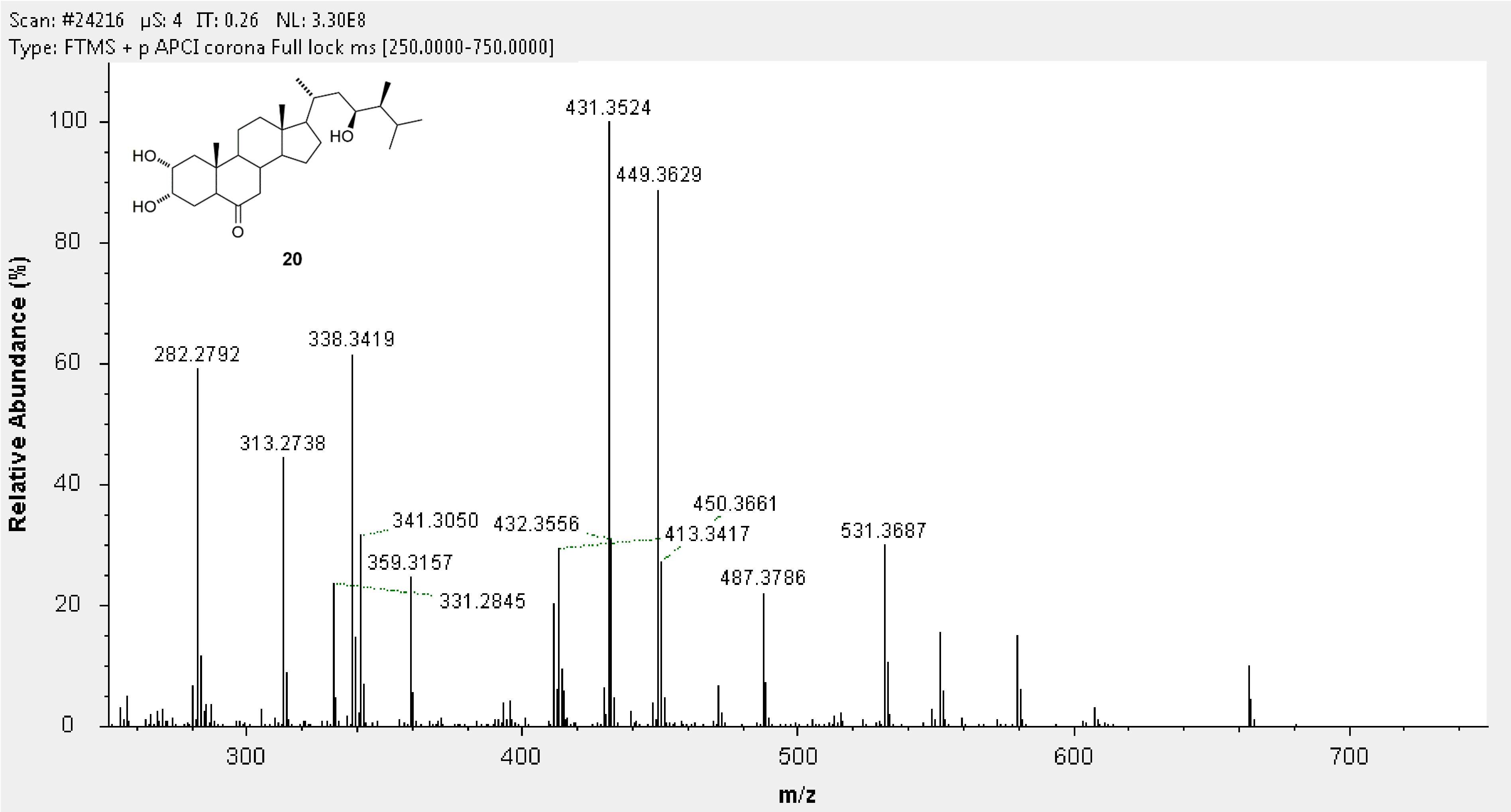
Scan: #24216 μS: 4 IT: 0.26 NL: 3.30E8
Type: FTMS + p APCI corona Full lock ms [250.0000-750.0000]
HO
HO
HO
O
20
431.3524
449.3629
282.2792
338.3419
313.2738
341.3050
432.3556
450.3661
413.3417
531.3687
359.3157
331.2845
487.3786
Relative Abundance (%)
m/z
0
20
40
60
80
100
300
400
500
600
700
