# Supplementary material for: A mechanistic framework for the recognition of chemically diverse brassinosteroids by BRI1-family receptor kinases
Source: Nat Plants. 2026 Aug 5;12(8):1579–95. doi: 10.1038/s41477-026-02346-0 (PMC13481262; doi:10.1038/s41477-026-02346-0)
Supplement: Supplementary file 4 [file 41477_2026_2346_MOESM3_ESM.pdf]

nature portfolio

Peer Review File

A mechanistic framework for the recognition of chemically diverse brassinosteroids by BRI1-family receptor kinases

Corresponding Author: Professor Michael Hothorn

Version 0:

Reviewer comments:

Reviewer #1

(Comments for the Author)
Caregnato et al. present an extensive structural and biochemical analysis of brassinosteroid (BR) perception by BRI1-family receptors. Through crystallography, quantitative binding assays, and physiological analyses, the authors explore how chemical modifications across a series of natural and synthetic BR analogs influence receptor–co-receptor complex formation and biological activity. The study combines multiple BRI1–SERK1 and BRL3 complex structures with detailed kinetic and root-growth data, revealing how side-chain hydroxylation and steroid geometry determine the extent of receptor activation.

Overall, this is an impressive and comprehensive work that integrates several perspectives. The experiments are executed very well, and the dataset is broad and consistent. The study consolidates the current understanding of BR recognition and provides valuable quantitative insight into how ligand chemistry modulates receptor complex stability and signaling strength.

While the study is of high quality, a few aspects would benefit from further clarification, particularly regarding its conceptual advance over previous work and the presentation of the crystallographic data.

Major Comments

1. The structural and kinetic framework presented here closely follows established BRI1–SERK activation models. Much of the mechanistic basis—ligand-mediated co-receptor recruitment and dual-interface stabilization has been previously described, including by the same group. While the present work extends these principles to a broader ligand panel and somewhat higher-resolution data, it feels largely incremental and does not clearly delineate which aspects represent novel conceptual insights versus quantitative refinement. The authors should explicitly discuss how their results advance beyond earlier structural and biochemical analyses—clarifying whether new chemical structural principles emerge, or whether the novelty lies primarily in methodological scope.

2. The crystal structures span 2.14 Å to ~3.2 Å, with the BRL3–6-deoxocastasterone complex representing the lowest-resolution dataset. The authors note that the poorly diffracting crystals did not yield interpretable differential maps in the BR-binding pocket but nevertheless present an omit map in Fig. 3a–b. Even at higher resolutions, the mFo–DFc omit maps appear bulky and poorly defined when contoured at > 4 σ, which can under-represent genuine features and does not support confident assignment of ligand orientation—particularly for the hydroxyl groups and hydrogen-bond geometry. In their current form, these conformational interpretations appear somewhat speculative. Because this aspect forms a central part of the mechanistic argument, it would benefit from more rigorous examination and documentation. At minimum, the authors should provide additional supplementary panels showing both 2mFo–DFc maps (~1 σ) and mFo–DFc omit maps (~+3 σ) for each key ligand and interface, displaying the ligand together with its neighboring residues at identical contour levels. The refinement program and strategy should also be described in greater detail, including the criteria or decisions used to determine certain ligand orientations in lower resolution structures, as well as the overall and per-chain B-factors and ligand occupancies, which are not clearly reported in the main text.

Minor Comments

1. The writing is clear, polished, and technically precise, though the dense terminology may challenge a broader audience. It could be helpful, in a few places, to briefly reorient the reader to the overall signaling context or structural framework, particularly for those less familiar with the BR field.

2. The figures are well designed and convey the data effectively, but the figures' resolution in the provided version appears slightly grainy, and the font styles are not always consistent across panels (when comparing to images, at least how it appears

on my end). I recommend using a uniform sans-serif typeface throughout all figures and ensuring high-resolution export settings consistent with standards.

Reviewer #2

(Comments for the Author)
The manuscript by Caregnato et al. presents a comprehensive analysis of the interactions between the phytohormone brassinosteroid and the receptors in Arabidopsis, combining the biochemical, structural, and plant physiological methods. Interactions of four BR receptors including BRI1, BRL1, BRL2, and BRL3 with a wide range of natural and synthetic BRs, and further with the coreceptor SERK1 were characterized, providing insights into the selectivity and plasticity of the BR-binding pocket. BRL2 were suggested to be a weak BR receptor with different BR binding spectrum. Overall, the experiments were well-performed and the results are well-presented. However, I have several major and minor concerns for the authors:

Major points:
1. The authors suggest that different BR ligands act as antagonists or agonists thus finetuning BR signaling in planta. However, a critical assumption is that these ligands, particularly the BR biosynthesis intermediate are present in the extracellular space of cells where the signaling is initiated. As to our present knowledge that BR biosynthesis largely occurs intracellularly, like in the ER, whether these intermediates are present in the extracellular space need to be further validated. The BR levels quantified by the authors were only for the whole tissues. Specially, according to the BR export by ABCB19 and ABCB1, these transporters are reported to recognize only the bioactive BRs, not the intermediates (Ying et al., Science, 2024; Wei et al., Plant communications, 2025).

2. While the structural and biochemical data support BRL2's medium-affinity binding towards BR, the physiological evidence remains limited. The absence of a clear phenotype in brl2 mutants, though consistent with prior reports, raises questions about its biological significance. This is even puzzled by its much lower binding affinity towards SERK1 compared to BRI1 or BRL3. Complementation or overexpression analyses under specific developmental or environmental contexts (e.g., vascular-specific responses) could strengthen the claim that BRL2 functions as a bona fide BR receptor in vivo.

3. The authors determined the apo structure of BRL2's extracellular domain and identified structural variations with BRI1 or BRL3. However, for the structural comparisons presented in Fig. 4a or Supplementary Fig. 3b, the figure legends suggest that these were carried out using the Alphafold-predicted model. Why not compared with the determined structure? Moreover, would the observed structural variations contribute to the lower BR binding or the coreceptor binding of BRL2? This should be further clarified and investigated by the authors. In addition, one or more BR-bound structures of BRL2 would provide further insights into its ligand selectivity.

4. The authors suggest that the agonistic effect of 2-deoxybrassinolide may be caused by its entering into the biosynthetic pathway. Why other intermediates cannot enter the pathway? No evidence or data is provided here.

Minor points:
1. page 2, line 66, delete "BR_ligand_recognition_MH13.odt".

2. page 3, line 83, check the sentence, also page 4, line 122.

3. A sequence alignment of BRI1, BRL1, BRL2, and BRL3 would help to show the conservation in the binding pocket. Furthermore, according to Fig. 4a, several residues are unconserved besides the mentioned E614 site (page 9, line 262-263), like G662, Q638, M557 of BRL2. Could these variations lead to different binding affinities towards BR?

4. Fig. 3b, the acyl tail of 24-epibrassinolide fits bad into the density. The authors should further check that.

5. Supplementary Table1, for the Ramachandran plot, allowed region is missing. The authors should also further check to avoid the outliers. Moreover, parameters for the data collection are missing.

Reviewer #3

(Comments for the Author)
This manuscript by Caregnato et al presents a detailed characterization of different brassinosteroid hormones and the BRI1 family receptors. Although the field mostly thinks of downstream compounds in the brassinosteroid biosynthetic pathway, such as brassinolide, as the active signaling molecules, the authors show that a vast array of brassinosteroid hormones in the 15 that they test can bind to the receptors. They provide structural, mutational, and in planta evidence of the specificity and consequences of these interactions. Overall, this is a highly relevant and exceptionally conducted study. It will be a must-read in the field and change the way that we think about which BRs are potentially bioactive. I have a few minor comments and suggestions.

1. The authors show binding of many BR intermediates and their concentrations in plants. Is there more that can be said from this about the combined signaling capacity based on these specificities and abundances? How do their results in terms of hormone and receptor affinities translate into how bioactive each compound is in vivo and what their potential roles in signaling are?

2. It is claimed that BRL2 is a "medium affinity BR receptor" based on new binding data and a limited set of in vivo

experiments. It's commendable that the authors generated fresh brl2 CRISPR lines for this analysis, yet it's still unclear if BRL2 has a functional role in vivo. Could they use the same construct to generate a bri1brl1brl2brl3 quadruple receptor mutant? It would be interesting to compare this to the bri1brl1brl3 triple mutant, which to my knowledge is thought to be nearly blind to BRs. Based on their claim, one would expect to see some responsiveness to high concentrations of hormones that bind BRL2 that remains in the triple mutant, but would be abolished in the quadruple mutant. If there is no genetic evidence or functional consequence of the role of BRL2 in mediating BR signaling, it's difficult to justify calling it a "medium affinity BR receptor". This could be softened.
3. The authors also generated a BRL2:NLS-Venus transcriptional reporter. What does a translational line look like and does it give any clues into functional relevance?
4. I'm a bit confused about what the authors hoped to learn from the pBRL2-oBIR3-iBRL2 experiments. Given that these constructs are activated independent of ligand, they can only report on potential signaling activity, but don't yield insight as to what the receptor is actually doing in vivo. Please clarified.

Decision Letter:

20th October 2025

Dear Professor Hothorn, dear Michael,

First, let me apologise for the long time it took us to handle the peer-review of your study. But it is looking good with mostly positive reviewers.

Your Article, "A mechanistic framework for the recognition of chemically diverse brassinosteroids by BRI1-family receptor kinases" has now been seen by three referees. You will see from their comments below that while they find your work of interest, some important points are raised. We are interested in the possibility of publishing your study in Nature Plants, but would like to consider your response to these concerns in the form of a revised manuscript before we make a final decision on publication.

As you can read below, all referees are quite enthusiastic about the quality of the study. Most questions are about the biological significance of BRL2 and of the many tested compounds, which might not be present in the apoplast close to the (co-)receptors. Referee #1 has a more conceptual criticism about the real novelty of the work, being a (still impressive) quantitative refinement of mostly known mechanisms. We understand this concern and even partially share it, but we think that the scale and quality of your study is enough to move forward. Eventually we might switch the label of your study from Article to Resource, which changes basically nothing except the fact that the threshold for biological mechanism discoveries is a bit lower. Some interesting experiments are suggested (better resolution structures, quadruple mutant adding brl2 to the mix). Please do not hesitate to get in touch if you would like to discuss these issues further.

We therefore invite you to revise your manuscript considering all reviewer and editor comments. Please highlight changes in the manuscript text file and do not forget that most responses to reviewers should translate into changes in the text or figures. There is no need for a very precise version with all exact edits; just highlight the phrases you have modified or added to help editors and reviewers spot them.

We are committed to providing a fair and constructive peer-review process. Do not hesitate to contact us if there are specific requests from the reviewers that you believe are technically impossible or unlikely to yield a meaningful outcome.

When revising your manuscript please:

* Include a "Response to referees" document detailing, point-by-point, how you addressed each referee comment. If no action was taken to address a point, you must provide a compelling argument. This response will be sent back to the referees along with the revised manuscript.

* If you have not done so already please begin to revise your manuscript so that it conforms to our Article format instructions at http://www.nature.com/nplants/info/final-submission. Refer also to any guidelines provided in this letter.

* Include a revised version of any required reporting checklist. It will be available to referees (and, potentially, statisticians) to aid in their evaluation if the manuscript goes back for peer review. A revised checklist is essential for re-review of the paper.

* Pay close attention to our href="https://www.nature.com/nature-portfolio/editorial-policies/image-integrity">Digital Image Integrity Guidelines. and to the following points. Please ensure:
-- that unprocessed scans are clearly labelled and match the gels and western blots presented in figures.
-- that control panels for gels and western blots are appropriately described as loading on sample processing controls
-- that all images in the paper are checked for duplication of panels and for splicing of gel lanes.
-- that you retain unprocessed data and metadata files after publication, ideally archiving data in perpetuity. These may be requested during the peer review and production process or after publication if any issues arise.

EXTENDED DATA FIGURES

When re-submitting your manuscript, please ensure that any supplementary figures and tables that are crucial to the

manuscript's conclusions are converted into Extended Data figures and tables to increase visibility of these data. Extended Data figures and tables are online-only (present in the online PDF and full-text HTML versions of the paper), peer-reviewed display items that provide essential background to the article but are not included in the main article due to space constraints. A maximum of ten Extended Data display items (figures and tables) is permitted.

Nature Plants strongly supports public availability of data and are therefore keen that the data used in your paper is placed in an appropriate public data repository. Alternatively, if this is not possible, you may present the data as Extended Data or Supplementary Information. If data can only be shared on request, please explain why in your Data Availability Statement, and also in the correspondence with your editor. Please note that for some data types, deposition in a public repository is mandatory.

Please use the link below to submit your revised manuscript and related files:

Link Redacted

Note: This URL links to your confidential home page and associated information about manuscripts you may have submitted, or that you are reviewing for us. If you wish to forward this email to co-authors, please delete the link to your homepage.

We hope to receive your revised manuscript within 3 months, but that is not a strict deadline, and there is no problem if you need more time. Please let us know when you have a rough idea of your revision schedule, once you have discussed with your co-authors. We will be happy to consider your revision so long as nothing similar has been accepted for publication at Nature Plants or published elsewhere.

We look forward to seeing the revised manuscript and thank you for the opportunity to review your work.

Sincerely,

*********** Reviewer Reports *************

--Reviewer #1=:

Caregnato et al. present an extensive structural and biochemical analysis of brassinosteroid (BR) perception by BRI1-family receptors. Through crystallography, quantitative binding assays, and physiological analyses, the authors explore how chemical modifications across a series of natural and synthetic BR analogs influence receptor–co-receptor complex formation and biological activity. The study combines multiple BRI1–SERK1 and BRL3 complex structures with detailed kinetic and root-growth data, revealing how side-chain hydroxylation and steroid geometry determine the extent of receptor activation.

Overall, this is an impressive and comprehensive work that integrates several perspectives. The experiments are executed very well, and the dataset is broad and consistent. The study consolidates the current understanding of BR recognition and provides valuable quantitative insight into how ligand chemistry modulates receptor complex stability and signaling strength.

While the study is of high quality, a few aspects would benefit from further clarification, particularly regarding its conceptual advance over previous work and the presentation of the crystallographic data.

Major Comments:

1. The structural and kinetic framework presented here closely follows established BRI1–SERK activation models. Much of the mechanistic basis—ligand-mediated co-receptor recruitment and dual-interface stabilization has been previously described, including by the same group. While the present work extends these principles to a broader ligand panel and somewhat higher-resolution data, it feels largely incremental and does not clearly delineate which aspects represent novel conceptual insights versus quantitative refinement. The authors should explicitly discuss how their results advance beyond earlier structural and biochemical analyses—clarifying whether new chemical structural principles emerge, or whether the novelty lies primarily in methodological scope.

2. The crystal structures span 2.14 Å to ~3.2 Å, with the BRL3–6-deoxocastasterone complex representing the lowest-resolution dataset. The authors note that the poorly diffracting crystals did not yield interpretable differential maps in the BR-binding pocket but nevertheless present an omit map in Fig. 3a–b. Even at higher resolutions, the mFo–DFc omit maps appear bulky and poorly defined when contoured at > 4 σ, which can under-represent genuine features and does not support confident assignment of ligand orientation—particularly for the hydroxyl groups and hydrogen-bond geometry. In their current form, these conformational interpretations appear somewhat speculative. Because this aspect forms a central part of the mechanistic argument, it would benefit from more rigorous examination and documentation. At minimum, the authors should provide additional supplementary panels showing both 2mFo–DFc maps (~1 σ) and mFo–DFc omit maps (~+3 σ) for each key ligand and interface, displaying the ligand together with its neighboring residues at identical contour levels. The refinement program and strategy should also be described in greater detail, including the criteria or decisions used to determine certain ligand orientations in lower resolution structures, as well as the overall and per-chain B-factors and ligand

occupancies, which are not clearly reported in the main text.

Minor Comments

1. The writing is clear, polished, and technically precise, though the dense terminology may challenge a broader audience. It could be helpful, in a few places, to briefly reorient the reader to the overall signaling context or structural framework, particularly for those less familiar with the BR field.

2. The figures are well designed and convey the data effectively, but the figures' resolution in the provided version appears slightly grainy, and the font styles are not always consistent across panels (when comparing to images, at least how it appears on my end). I recommend using a uniform sans-serif typeface throughout all figures and ensuring high-resolution export settings consistent with standards.

--Reviewer #2:

The manuscript by Caregnato et al. presents a comprehensive analysis of the interactions between the phytohormone brassinosteroid and the receptors in Arabidopsis, combining the biochemical, structural, and plant physiological methods. Interactions of four BR receptors including BRI1, BRL1, BRL2, and BRL3 with a wide range of natural and synthetic BRs, and further with the coreceptor SERK1 were characterized, providing insights into the selectivity and plasticity of the BR-binding pocket. BRL2 were suggested to be a weak BR receptor with different BR binding spectrum. Overall, the experiments were well-performed and the results are well-presented. However, I have several major and minor concerns for the authors:

Major points:

1. The authors suggest that different BR ligands act as antagonists or agonists thus finetuning BR signaling in planta. However, a critical assumption is that these ligands, particularly the BR biosynthesis intermediate are present in the extracellular space of cells where the signaling is initiated. As to our present knowledge that BR biosynthesis largely occurs intracellularly, like in the ER, whether these intermediates are present in the extracellular space need to be further validated. The BR levels quantified by the authors were only for the whole tissues. Specially, according to the BR export by ABCB19 and ABCB1, these transporters are reported to recognize only the bioactive BRs, not the intermediates (Ying et al., Science, 2024; Wei et al., Plant communications, 2025).

2. While the structural and biochemical data support BRL2's medium-affinity binding towards BR, the physiological evidence remains limited. The absence of a clear phenotype in brl2 mutants, though consistent with prior reports, raises questions about its biological significance. This is even puzzled by its much lower binding affinity towards SERK1 compared to BRI1 or BRL3. Complementation or overexpression analyses under specific developmental or environmental contexts (e.g., vascular-specific responses) could strengthen the claim that BRL2 functions as a bona fide BR receptor in vivo.

3. The authors determined the apo structure of BRL2's extracellular domain and identified structural variations with BRI1 or BRL3. However, for the structural comparisons presented in Fig. 4a or Supplementary Fig. 3b, the figure legends suggest that these were carried out using the Alphafold-predicted model. Why not compared with the determined structure? Moreover, would the observed structural variations contribute to the lower BR binding or the coreceptor binding of BRL2? This should be further clarified and investigated by the authors. In addition, one or more BR-bound structures of BRL2 would provide further insights into its ligand selectivity.

4. The authors suggest that the agonistic effect of 2-deoxybrassinolide may be caused by its entering into the biosynthetic pathway. Why other intermediates cannot enter the pathway? No evidence or data is provided here.

Minor points:

1. page 2, line 66, delete "BR_ligand_recognition_MH13.odt".

2. page 3, line 83, check the sentence, also page 4, line 122.

3. A sequence alignment of BRI1, BRL1, BRL2, and BRL3 would help to show the conservation in the binding pocket. Furthermore, according to Fig. 4a, several residues are unconserved besides the mentioned E614 site (page 9, line 262-263), like G662, Q638, M557 of BRL2. Could these variations lead to different binding affinities towards BR?

4. Fig. 3b, the acyl tail of 24-epibrassinolide fits bad into the density. The authors should further check that.

5. Supplementary Table1, for the Ramachandran plot, allowed region is missing. The authors should also further check to avoid the outliers. Moreover, parameters for the data collection are missing.

--Reviewer #3:

This manuscript by Caregnato et al presents a detailed characterization of different brassinosteroid hormones and the BRI1 family receptors. Although the field mostly thinks of downstream compounds in the brassinosteroid biosynthetic pathway, such as brassinolide, as the active signaling molecules, the authors show that a vast array of brassinosteroid hormones in the 15 that they test can bind to the receptors. They provide structural, mutational, and in planta evidence of the specificity and consequences of these interactions. Overall, this is a highly relevant and exceptionally conducted study. It will be a must-read in the field and change the way that we think about which BRs are potentially bioactive. I have a few minor comments and suggestions.

1. The authors show binding of many BR intermediates and their concentrations in plants. Is there more that can be said from this about the combined signaling capacity based on these specificities and abundances? How do their results in terms of hormone and receptor affinities translate into how bioactive each compound is in vivo and what their potential roles in signaling are?

2. It is claimed that BRL2 is a "medium affinity BR receptor" based on new binding data and a limited set of in vivo experiments. It's commendable that the authors generated fresh brl2 CRISPR lines for this analysis, yet it's still unclear if BRL2 has a functional role in vivo. Could they use the same construct to generate a bri1brl1brl2brl3 quadruple receptor mutant? It would be interesting to compare this to the bri1brl1brl3 triple mutant, which to my knowledge is thought to be nearly blind to BRs. Based on their claim, one would expect to see some responsiveness to high concentrations of hormones that bind BRL2 that remains in the triple mutant, but would be abolished in the quadruple mutant. If there is no genetic evidence or functional consequence of the role of BRL2 in mediating BR signaling, it's difficult to justify calling it a "medium affinity BR receptor". This could be softened.

3. The authors also generated a BRL2:NLS-Venus transcriptional reporter. What does a translational line look like and does it give any clues into functional relevance?

4. I'm a bit confused about what the authors hoped to learn from the pBRL2-oBIR3-iBRL2 experiments. Given that these constructs are activated independent of ligand, they can only report on potential signaling activity, but don't yield insight as to what the receptor is actually doing in vivo. Please clarified.

********************END********************

Version 1:

Reviewer comments:

Reviewer #1

(Comments for the Author)
The revised manuscript is improved, particularly with the addition of supplementary electron density maps and the expanded description of the structural refinement. These additions make the structural models more transparent and easier to evaluate.

Most of the previous points regarding map presentation and methodology have been addressed. One aspect that could still benefit from minor clarification relates to the interpretation of the ligand density. While the additional 2mFo–DFc and omit maps are helpful, the resolution of several datasets (in the ~2.5–3.2 Å range) naturally limits the level of detail that can be assigned to ligand orientation, especially for hydroxyl positioning and hydrogen-bonding interactions. In a few places, the interpretation still reads with a level of precision that may be slightly higher than what the density fully supports.

It would be helpful to briefly acknowledge this limitation and slightly temper the wording where specific interactions are inferred from less well-defined density. A short clarification regarding the choice to fix ligand occupancies at 1 would also improve transparency of the modeling approach.

Reviewer #2

(Comments for the Author)
In the revised manuscript, the authors have performed additional experiments and made textual revisions to address my previous concerns. I acknowledge that accurately determining the in vivo presence and concentration of the proposed BR ligands remains technically challenging at present, leaving open the question of whether they function as physiologically relevant hormones. This limitation may also compromise the overall significance of the findings, as also noted by Reviewer #1. The functional characterization of BRL2 has now been moved to the supplementary information, which helps to sharpen the manuscript's focus on the ligand specificity of BR receptors. I have no further comments.

Reviewer #3

(Comments for the Author)

The authors have satisfactorily addressed all of my comments, including softening claims about BRL2. I have no further comments. This is a strong and timely manuscript.

Decision Letter:

Our ref: NPLANTS-250920777A

3rd April 2026

Dear Dr. Hothorn, dear Michael,

First, let me apologise for the long time it took us to handle the peer-review of your study. But I have good news.

Thank you for submitting your revised manuscript "A mechanistic framework for the recognition of chemically diverse brassinosteroids by BRI1-family receptor kinases" (NPLANTS-250920777A). It has now been seen by the original referees and their comments are below. The reviewers find that the paper has improved in revision, and therefore we'll be happy in principle to publish it in Nature Plants, pending minor revisions to satisfy the referees' final requests and to comply with our editorial and formatting guidelines.

As you can read below, the referees are now almost totally satisfied with the manuscript. Referees #1 and #2 still think that you might want to temper some wording and acknowledge a few limitations in the main text - this will not weaken the conclusions or lower the significance of the results. If this is properly done, there won't be any need for sending back the manuscript to reviewers.

As the current version of your manuscript is in a PDF format, please email us *now* a copy of a text-only file in an editable format (Microsoft Word or LaTex) as we cannot proceed with PDFs. Don't forget to CC plants@nature.com.

We therefore invite you to revise your manuscript considering all reviewer and editor comments. While you are working on this, we will be performing detailed checks on your paper and will send you a checklist detailing our editorial and formatting requirements in about a week (hopefully). Please do not upload the final materials and make any revisions until you receive this additional information from us. Once you receive the checklist, you can integrate all changes into a final version.

Thank you again for your interest in Nature Plants Please do not hesitate to contact me if you have any questions.

Sincerely,

*********** Reviewer Reports *************

--Reviewer #1:

The revised manuscript is improved, particularly with the addition of supplementary electron density maps and the expanded description of the structural refinement. These additions make the structural models more transparent and easier to evaluate.

Most of the previous points regarding map presentation and methodology have been addressed. One aspect that could still benefit from minor clarification relates to the interpretation of the ligand density. While the additional 2mFo–DFc and omit maps are helpful, the resolution of several datasets (in the ~2.5–3.2 Å range) naturally limits the level of detail that can be assigned to ligand orientation, especially for hydroxyl positioning and hydrogen-bonding interactions. In a few places, the interpretation still reads with a level of precision that may be slightly higher than what the density fully supports.

It would be helpful to briefly acknowledge this limitation and slightly temper the wording where specific interactions are inferred from less well-defined density. A short clarification regarding the choice to fix ligand occupancies at 1 would also improve transparency of the modeling approach.

--Reviewer #2:

In the revised manuscript, the authors have performed additional experiments and made textual revisions to address my previous concerns. I acknowledge that accurately determining the in vivo presence and concentration of the proposed BR ligands remains technically challenging at present, leaving open the question of whether they function as physiologically relevant hormones. This limitation may also compromise the overall significance of the findings, as also noted by Reviewer #1. The functional characterization of BRL2 has now been moved to the supplementary information, which helps to sharpen the manuscript’s focus on the ligand specificity of BR receptors. I have no further comments.

--Reviewer #3:

The authors have satisfactorily addressed all of my comments, including softening claims about BRL2. I have no further comments. This is a strong and timely manuscript.

**************** END *******************

Version 2:

Decision Letter:

9th June 2026

Dear Professor Hothorn, dear Michael,

We are pleased to inform you that your Article entitled "A mechanistic framework for the recognition of chemically diverse brassinosteroids by BRI1-family receptor kinases", has now been accepted for publication in Nature Plants.

Over the next few weeks, your paper will be copyedited to ensure that it conforms to Nature Plants style. We look particularly carefully at the titles of all papers to ensure that they are relatively brief and understandable.

Once your paper is typeset, you will receive an email with a link to choose the appropriate publishing options for your paper and our Author Services team will be in touch regarding any additional information that may be required.

After the grant of rights is completed, you will receive a link to your electronic proof via email with a request to make any corrections within 48 hours. If, when you receive your proof, you cannot meet this deadline, please inform us at rjsproduction@springernature.com immediately.

You will not receive your proofs until the publishing agreement has been received through our system.

You may wish to make your media relations office aware of your accepted publication, in case they consider it appropriate to organize some internal or external publicity. Once your paper has been scheduled you will receive an email confirming the publication details. This is normally 3-4 working days in advance of publication. If you need additional notice of the date and time of publication, please let the production team know when you receive the proof of your article to ensure there is sufficient time to coordinate. Further information on our embargo policies can be found here: https://www.nature.com/authors/policies/embargo.html

Due to the importance of these deadlines, we ask you please us know now whether you will be difficult to contact over the next month. If this is the case, we ask you provide us with the contact information (email, phone and fax) of someone who will be able to check the proofs on your behalf, and who will be available to address any last-minute problems.

Acceptance of your manuscript is conditional on all authors' agreement with our publication policies (see http://www.nature.com/authors/policies/index.html). In particular your manuscript must not be published elsewhere.

If you have any questions about our publishing options, costs, Open Access requirements, or our legal forms, please contact ASJournals@springernature.com

Authors may need to take specific actions to achieve compliance with funder and institutional open access mandates. If your research is supported by a funder that requires immediate open access (e.g. according to Plan S principles or the NIH public access policy) then you should select the gold OA route, and we will direct you to the compliant route where possible. Because authors warrant under our subscription licensing terms that they haven’t committed to licensing any version of their article under a licence inconsistent with the terms of our agreement – including the applicable embargo period – publication under the subscription model isn’t suitable for authors whose funders require no embargo.

An online order form for reprints of your paper is available at https://www.nature.com/reprints/author-reprints.html. All co-authors, authors' institutions and authors' funding agencies can order reprints using the form appropriate to their geographical region.

We welcome the submission of potential cover material (including a short caption of around 40 words) related to your manuscript; suggestions should be sent to Nature Plants as electronic files (the image should be 300 dpi at 210 x 297 mm in either TIFF or JPEG format). Please note that such pictures should be selected more for their aesthetic appeal than for their scientific content, and that colour images work better than black and white or grayscale images. Please include a written description of your image and how it was created along with your image files. Please do not try to design a cover with the Nature Plants logo etc., and please do not submit composites of images related to your work. I am sure you will understand that we cannot make any promise as to whether any of your suggestions might be selected for the cover of the journal.

You can now use a single sign-on for all your accounts, view the status of all your manuscript submissions and reviews, access usage statistics for your published articles and download a record of your refereeing activity for the Nature journals.

To assist our authors in disseminating their research to the broader community, our SharedIt initiative provides you with a unique shareable link that will allow anyone (with or without a subscription) to read the published article. Recipients of the link with a subscription will also be able to download and print the PDF.

As soon as your article is published, you will receive an automated email with your shareable link.

With kind regards,

Nature Plants

P.S. Click on the following link if you would like to recommend Nature Plants to your librarian
http://www.nature.com/subscriptions/recommend.html#forms

** Visit the Springer Nature Editorial and Publishing website at www.springernature.com/editorial-and-publishing-jobs for more information about our career opportunities. If you have any questions please click here.**

Open Access This Peer Review File is licensed under a Creative Commons Attribution 4.0 International License, which permits use, sharing, adaptation, distribution and reproduction in any medium or format, as long as you give appropriate credit to the original author(s) and the source, provide a link to the Creative Commons license, and indicate if changes were made.
In cases where reviewers are anonymous, credit should be given to 'Anonymous Referee' and the source.
The images or other third party material in this Peer Review File are included in the article's Creative Commons license, unless indicated otherwise in a credit line to the material. If material is not included in the article's Creative Commons license and your intended use is not permitted by statutory regulation or exceeds the permitted use, you will need to obtain permission directly from the copyright holder.
To view a copy of this license, visit https://creativecommons.org/licenses/by/4.0/

Response to reviewers - Caregnato, Chen et al., A mechanistic framework for the recognition of chemically diverse brassinosteroids by BRI1-family receptor kinases.

Author response in blue, changes to the manuscript are highlighted in red.

--Reviewer #1

Caregnato et al. present an extensive structural and biochemical analysis of brassinosteroid (BR) perception by BRI1-family receptors. Through crystallography, quantitative binding assays, and physiological analyses, the authors explore how chemical modifications across a series of natural and synthetic BR analogs influence receptor–co-receptor complex formation and biological activity. The study combines multiple BRI1–SERK1 and BRL3 complex structures with detailed kinetic and root-growth data, revealing how side-chain hydroxylation and steroid geometry determine the extent of receptor activation.

Overall, this is an impressive and comprehensive work that integrates several perspectives. The experiments are executed very well, and the dataset is broad and consistent. The study consolidates the current understanding of BR recognition and provides valuable quantitative insight into how ligand chemistry modulates receptor complex stability and signaling strength.

While the study is of high quality, a few aspects would benefit from further clarification, particularly regarding its conceptual advance over previous work and the presentation of the crystallographic data.

Major Comments:

1. The structural and kinetic framework presented here closely follows established BRI1–SERK activation models. Much of the mechanistic basis—ligand-mediated co-receptor recruitment and dual-interface stabilization has been previously described, including by the same group. While the present work extends these principles to a broader ligand panel and somewhat higher-resolution data, it feels largely incremental and does not clearly delineate which aspects represent novel conceptual insights versus quantitative refinement. The authors should explicitly discuss how their results advance beyond earlier structural and biochemical analyses—clarifying whether new chemical structural principles emerge, or whether the novelty lies primarily in methodological scope.

Our response: We agree with the reviewer that the fundamental mechanism of BR ligand binding and ligand-induced co-receptor recruitment has been well established. Building on the structural, biochemical, and genetic data available at the time, a major agrochemical company designed a series of novel BR agonists and antagonists and provided them to us for evaluation. However, none of these compounds exhibited bioactivity, nor did they demonstrate detectable binding to BRI1 in vitro. The experiments presented in our manuscript were undertaken to address these limitations by systematically defining the chemical space within brassinosteroids that is required for bioactivity. To better emphasize this objective and its significance, we have revised the discussion section accordingly (lines 383-389) "The most unexpected finding is that hydroxylation at C_{23} and the C_{24} stereochemistry in the BR aliphatic tail are critical determinants for high affinity receptor binding (Fig. 2b–d) and BR bioactivity (Fig. 3c and Fig. 7). Consistent with this, C_{23} hydroxylase mutants display severe dwarfism[9,51]. A second key finding from our analysis is that improper ligand positioning within the steroid-binding pocket can be accommodated by the isolated receptor, yet becomes detrimental for co-receptor recruitment. For instance, the absence of a C_6 ketone is tolerated by BRI1 and BRL3 (Fig. 2b–d), but significantly compromises high-affinity binding to SERK1 (Fig. 6c–f and Fig. 7)."

In addition, we present for first complete mutational analysis of the steroid binding site in BR receptors . We have added the following statement to our discussion section (lines 401-406) "We identified a single point mutation, Trp564His, that results in a strong BR loss-of-function phenotype. This finding

demonstrates that BRI1 can be effectively inactivated by a single amino acid substitution, rather than requiring the combination of multiple mutations as previously reported[46]. Two additional residues (Tyr599 and Tyr642) are critically involved in the proper positioning of the ligand in the BR binding pocket, and we report a novel gain-of-function allele (Phe681Ala) at the entry of the steroid binding pocket (Fig. 5d-f and Fig. 7)."

Our summary statement now reads (lines 426-428) "Taken together, our work defines the chemical determinants for a small molecule-based molecular glue mechanism allowing formation of an active receptor – steroid ligand – co-receptor signaling complex."

2. The crystal structures span 2.14 Å to ~3.2 Å, with the BRL3–6-deoxocastasterone complex representing the lowest-resolution dataset. The authors note that the poorly diffracting crystals did not yield interpretable differential maps in the BR-binding pocket but nevertheless present an omit map in Fig. 3a–b.

Our response: This is a misunderstanding. Crystals of B**RI1** co-crystallized with 6-deoxocastasterone yielded poorly diffracting crystals, while we could obtain a 3.2 Å structure of B**RL3** bound to 6-deoxocastasterone. We have rephrased the result section to better highlight this fact (lines 168-171) "Crystals grown with cathasterone, 24-epicastasterone, or 28-homocastasterone showed no interpretable difference electron density in the BR-binding pocket, while a BRI1 - 6-deoxocastasterone complex yielded poorly diffracting crystals."

Even at higher resolutions, the mFo–DFc omit maps appear bulky and poorly defined when contoured at > 4 σ, which can under-represent genuine features and does not support confident assignment of ligand orientation—particularly for the hydroxyl groups and hydrogen-bond geometry. In their current form, these conformational interpretations appear somewhat speculative. Because this aspect forms a central part of the mechanistic argument, it would benefit from more rigorous examination and documentation. At minimum, the authors should provide additional supplementary panels showing both 2mFo–DFc maps (~1 σ) and mFo–DFc omit maps (~+3 σ) for each key ligand and interface, displaying the ligand together with its neighboring residues at identical contour levels. The refinement program and strategy should also be described in greater detail, including the criteria or decisions used to determine certain ligand orientations in lower resolution structures, as well as the overall and per-chain B-factors and ligand occupancies, which are not clearly reported in the main text.

Our response: We fully agree with the reviewer that it would be desirable to obtain high-resolution structures of the BRI1, BRL2 and BRL3 ectodomains in complex with different brassinosteroids. However, BRI1, BRL2, and BRL3 are highly glycosylated (see Fig. 4a) and tend to form crystals with few crystal contacts and an associated high solvent content ($BRI1_{monoclinic}$ V_M=3.75 Å^3/Da, 68% solvent content; $BRL3_{triclinic}$ V_M=3.5 Å^3/Da, 65% solvent content; $BRL2_{tetragonal}$ V_M=3.86 Å^3/Da 70% solvent content; $BRL2_{monoclinic}$ V_M=3.5 Å^3/Da, 65% solvent content; an "average" crystal with ~50% solvent content would have a Matthews coefficient V_M of ~2.5 Å^3/Da). In addition, the poor solubility of BRs in aqueous buffers limits the concentrations that can be used in co-crystallization experiments. We have collected more than 200 datasets for BRI1, BRL3 and BRL2 and selected the best diffracting crystals, the resolution range of which matches well the resolution distribution of all

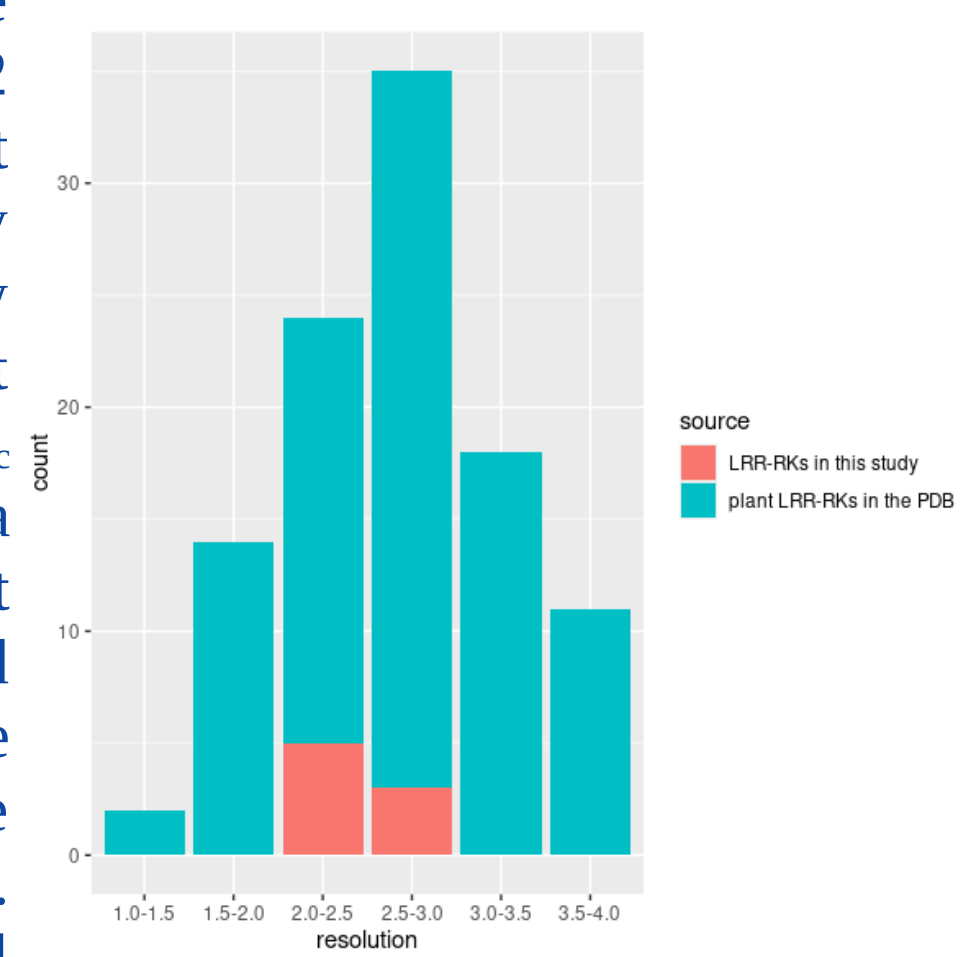

Reviewer Fig. 1. Resolution distribution histogram of plant LRR-RK structures currently available in the Protein Data Bank (February 2026).

~100 plant LRR-RK structures currently deposited in the PDB (Reviewer Fig. 1). Following the reviewers suggestion, we have added a new Supplementary Fig. 2, which provides $2mF_o$-DF_c and mF_o-DF_c omit maps of the BRI1 and BRL3 ligand binding sites bound to the brassinosteroids shown in Fig. 3a, as requested. We have updated the method section (lines 537-543) "Restraints for the BR ligand were generated from SMILES strings using phenix.elbow[72] or JLigand[73]. Ligand were fit manually into mFo–DFc omit maps and ligand occupancies were set to 1. All structures were completed through iterative cycles of manual model building in COOT[74] and restrained TLS refinement in phenix.refine[75]. Analysis of the refined structures with phenix.molprobity[76] revealed good to excellent stereochemistry. Final omit maps of the different BR ligands were generated with phenix.polder[77]."
The revised Supplementary Table 1 contains now additional data collection statistics (data collection wavelength, highest resolution shell, R_{meas}, CC1/2, I/sigI, completeness and data redundancy for highest shell), per-chain B-factors for all brassinosteroid ligands, as well as the mean B-factors for the N-glyans, buffer and solvent molecules. In response to reviewer #2, several structures have been refined with improved stereochemistry. All structures have now been released from the PDB (February 18, 2026).

Minor Comments

1. The writing is clear, polished, and technically precise, though the dense terminology may challenge a broader audience. It could be helpful, in a few places, to briefly reorient the reader to the overall signaling context or structural framework, particularly for those less familiar with the BR field.

Our response: Based on the reviewer comments (see below) we have shortened the result and discussion sections of our revised manuscript, and included more comprehensive summary statements in the different subsections, including (lines 161-165) "Overall, our quantitative binding data demonstrate that BR receptors recognize a surprisingly broad range of structurally diverse BRs with high affinity. The configuration and size of the aliphatic side chain, particularly the presence of a C_{23} hydroxyl group and the correct C_{24} stereochemistry, drive high-affinity receptor binding." (lines 282-284) "These experiments suggest that BRL2 may have the capacity to initiate a SERK-dependent signaling cascade, yet its physiological ligand and physiological function remain to be characterized." (lines 323-327) "Overall, we observed a strong correlation between the in vitro ligand-binding affinities and the in vivo growth phenotypes of BRI1 receptor mutants that target amino acids involved in ligand coordination. Our analysis revealed novel loss-of-function (Trp564-His) and gain-of-function (Phe681-Ala) alleles, which can be used to precisely control BRI1 activity in planta." (lines 360-361) "We hypothesise that BRs that bind to BRI1 with high affinity but prevent SERK association may act as receptor antagonists." (lines 426-428) "Taken together, our work defines the molecular determinants for a small molecule-based molecular glue mechanism allowing formation of an active receptor – steroid ligand – co-receptor signaling complex."

2. The figures are well designed and convey the data effectively, but the figures' resolution in the provided version appears slightly grainy, and the font styles are not always consistent across panels (when comparing to images, at least how it appears on my end). I recommend using a uniform sans-serif typeface throughout all figures and ensuring high-resolution export settings consistent with standards.

Our response: Checked and corrected throughout. High resolutions figures have been included in the revision upload.

--Reviewer #2:

The manuscript by Caregnato et al. presents a comprehensive analysis of the interactions between the phytohormone brassinosteroid and the receptors in Arabidopsis, combining the biochemical, structural, and plant physiological methods. Interactions of four BR receptors including BRI1, BRL1, BRL2, and BRL3 with a wide range of natural and synthetic BRs, and further with the coreceptor SERK1 were characterized, providing insights into the selectivity and plasticity of the BR-binding pocket. BRL2 were suggested to be a weak BR receptor with different BR binding spectrum. Overall, the experiments were well-performed and the results are well-presented. However, I have several major and minor concerns for the authors:

Major points:

1. The authors suggest that different BR ligands act as antagonists or agonists thus finetuning BR signaling in planta. However, a critical assumption is that these ligands, particularly the BR biosynthesis intermediate are present in the extracellular space of cells where the signaling is initiated. As to our present knowledge that BR biosynthesis largely occurs intracellularly, like in the ER, whether these intermediates are present in the extracellular space need to be further validated. The BR levels quantified by the authors were only for the whole tissues. Specially, according to the BR export by ABCB19 and ABCB1, these transporters are reported to recognize only the bioactive BRs, not the intermediates (Ying et al., Science, 2024; Wei et al., Plant communications, 2025).

Our response: We fully agree with the reviewer that a bona fide BR receptor ligand must be present in the apoplastic cell wall compartment to exert biological activity. Throughout the results and discussion sections, we have therefore taken care not to imply a direct correlation between the in vitro binding affinities of different BRs to BRI1 and their biological activities in planta. Given the extremely low endogenous concentrations of BR metabolites in plant tissues, it is, to the best of our knowledge, currently not possible to quantify these compounds at cellular, let alone subcellular, resolution. Consequently, we cannot determine which of the tested compounds are present in the cell wall compartment. We have however extended our BR metabolite analysis, which now includes typhasterol, to three different genotypes (wild type, a *bri1* loss-of-function mutant and a constitutive active oBIR3-iBRI1 chimeric line). The data are presented in revised Fig. 3d, the corresponding statement in the result section now reads (lines 218-225) “We next compared BR levels in wild-type, *bri1* null[47] and oBIR3-iBRI1[52] constitutively active mutant seedlings. In the *bri1* mutant, brassinolide, castasterone and typhasterol levels were elevated by two to 3-fold, respectively, compared to wild type (Fig. 3d), as previously described[45]. This aligns with earlier findings that BR metabolic and catabolic gene expression is altered in *bri1* loss-of-function mutants[53], partly mediated by the BR-regulated transcription factor BZR1[40,54]. In oBIR3-iBRI1 seedlings, which display constitutive BR responses[52], brassinolide and castasterone levels were reduced compared to wild type (Fig. 3d).”

In the case of ABCB1 and ABCB19, stimulation of ATPase activity and transporter binding has been established for brassinolide, 24-epibrassinolide and 24-epicastasterone, direct transport evidence for brassinolide (Ying et al., Science, 2024; Wei et al., Plant Communications, 2025). The very early biosynthetic pathway metabolites campesterol and (22S)-22-hydroxycampesterol have been tested in ATPassays (Ying et al., Science, 2024; Wei et al., Plant Communications, 2025). It remains possible that other BR metabolites such as castasterone, 28-norbrassinolide, typhasterol or 2-deoxybrassinolide could be transported by ABCB1/ABCB19, which would be interesting to test in the future. Given the fact that ABCB1/ABCB19 has also been reported to be involved in auxin transport and auxin physiology (see Geisler, https://doi.org/10.1016/j.molp.2024.05.009 and references therein) and that the *abcb1 abcb19* double mutant does not phenocopy *bri1* null mutant alleles (Ying et al., Science, 2024), other transporters or other delivery mechanism may exist that allow brassinosteroids to reach the

extracellular space. It is also noteworthy that ER localization has only been established for a subset of BR biosynthetic enzymes, and that BR biosynthetic pathway is not complete (Kim et al., Plant Physiol, 2006; Northey et al., Nat Plants, 2016; Vukasinovic et al., Nat Plants, 2021). To better highlight these outstanding questions in BR transport and signaling, we have added a statement to our discussion section (lines 412-416) “Based on binding profiles (Fig. 2c,d, Fig. 6c,d), bioactivity data, and in vivo BR levels[11,48,50] (Fig. 3d), we propose that brassinolide and castasterone (C_{28}), 28-norbrassinolide (C_{27}), and possibly 28-homocastasterone (C_{29}) may act as physiological BR ligands in Arabidopsis, but it is presently unknown if and how these metabolites reach the cell wall compartment to interact with the ligand binding domains of BR receptors.”

2. While the structural and biochemical data support BRL2’s medium-affinity binding towards BR, the physiological evidence remains limited. The absence of a clear phenotype in brl2 mutants, though consistent with prior reports, raises questions about its biological significance. This is even puzzled by its much lower binding affinity towards SERK1 compared to BRI1 or BRL3. Complementation or overexpression analyses under specific developmental or environmental contexts (e.g., vascular-specific responses) could strengthen the claim that BRL2 functions as a bona fide BR receptor in vivo.

Our response: We agree with the reviewer that the physiological functions of BRL2 in Arabidopsis are currently not well defined. To address this, we undertook substantial efforts to identify a loss-of-function phenotype for BRL2 in Arabidopsis thaliana. Specifically, we (1) re-examined the reported leaf vein patterning phenotype (Clay & Nelson, 2002), (2) performed BR response assays, and (3) generated and phenotyped a new brl2 CRISPR/Cas9 knockout allele. However, none of these approaches revealed a reproducible phenotype that could serve as a basis for the complementation assays suggested by the reviewer. Importantly, our transcriptional and newly added translational reporter lines (see response to Reviewer #3) consistently support the conclusion that BRL2 functions as a vascular receptor kinase. In parallel, we are generating a *brl1 brl2 brl3* CRISPR/Cas9 triple knock-out mutant to investigate potential roles of BRL2 in vascular development and function, in collaboration with a research group specializing in plant vascular biology. However, we consider such extensive functional analyses to be beyond the scope of the present manuscript, which focuses on the ligand specificity of BR receptors. We have therefore moved the revised functional analysis of BRL2 to the Supplementary Information. The data are presented in the newly generated Supplementary Fig. 4. The corresponding result section now reads (lines 267-281) ”VH1/BRL2 has been characterized as a vascular receptor kinase involved in leaf vein patterning[34,55]. The previously described *brl2-1* mutant[55] showed wild type-like responses in BR hypocotyl growth and BES1 dephosphorylation assays (Supplementary Fig. 5a,b). In our hands, the leaf vein patterns of *brl2-1* plants were not significantly different from the Col-0 wild type (Supplementary Fig. 5c). Transcriptional *BRL2prom*::NLS-3xmVenus and translational *BRL2prom*::BRL2-mCitrine reporter lines confirmed the expression of BRL2 in vascular tissues across different developmental stages (Supplementary Fig. 5d,e). We generated a second *brl2-2* loss-of-function allele using CRISPR/Cas9 gene editing (Supplementary Fig. 5f), which like *brl2-1* was indistinguishable from wild type (Supplementary Fig. 5g). We have previously reported that receptor chimeras, in which the extracellular and transmembrane domains of BIR3 (oBIR3) are fused to the cytoplasmic kinase domain of a SERK-dependent receptor kinase (iRK), trigger constitutive, ligand-independent signaling responses[38,52]. A *BRL2prom*::oBIR3-iBRL2 chimera in Col-0 wild-type background had elongated, slender petioles and curly leaves, similar to BRI1^{sud1} gain-of-function mutants[24,47] (Supplementary Fig. 5g). However, the rosette phenotypes were milder than those of *BRI1prom*::oBIR3-iBRI1 chimeras[52] (Supplementary Fig. 5g).“ We have removed all statements relating to BRL2 being a BR receptor, as requested by the reviewer. Specifically, the sub-section heading has been revised to (line 243) “BRL2 binds BRs with medium affinity in vitro”, following statement has been removed from the result section: “Our experiments suggest that BRL2

functions as a medium-affinity receptor for C_{28} BRs (Fig. 1b)" In line with this, the BR-binding pocket of BRI1, BRL1/3 and BRL2 share a high degree of structural and sequence conservation. The only notable exception is Glu614 in BRL2, which corresponds to Met657 and Ile642 in BRI1 and BRL3, respectively (Fig. 4a, Supplementary Fig. 3b)."

The toned-down summary of the BRL2 result section now reads (lines 282-284) "These experiments suggest that BRL2 may have the capacity to initiate a SERK-dependent signaling cascade, yet its physiological ligand and function remain to be characterized."

Regarding the lower binding affinity of BRL2 vs. SERK1, we have re-performed the binding assay shown in Fig. 6b (intially performed with a constant concentration of 1 µM brassinolide in the GCI buffer) in the presence of a higher concentration of brassinolide (5 µM). We have also included the SERK1 homolog BAK1. As shown in Reviewer Fig. 2, we observe a somewhat higher affinity binding in the presence of a higher, saturating concentration of brassinolide, but possibly due to the higher concentration of DMSO in these assays the data are much more noisy (known dissociation constants for SERKs vs. ligand-bound RKs range from the mid-nanomolar to the low micromolar range). We thus decided to keep the current data shown in Fig. 6b to allow for comparison with BRI1, BRL1 and BRL3. We have added the following clarification to the figure legend (lines 812-814) "GCI binding kinetics of amine-coupled and brassinolide-bound BRI1, BRL1, BRL2, and BRL3 ectodomains vs. SERK1 in the presence of a constant concentration of 1 µM BL."

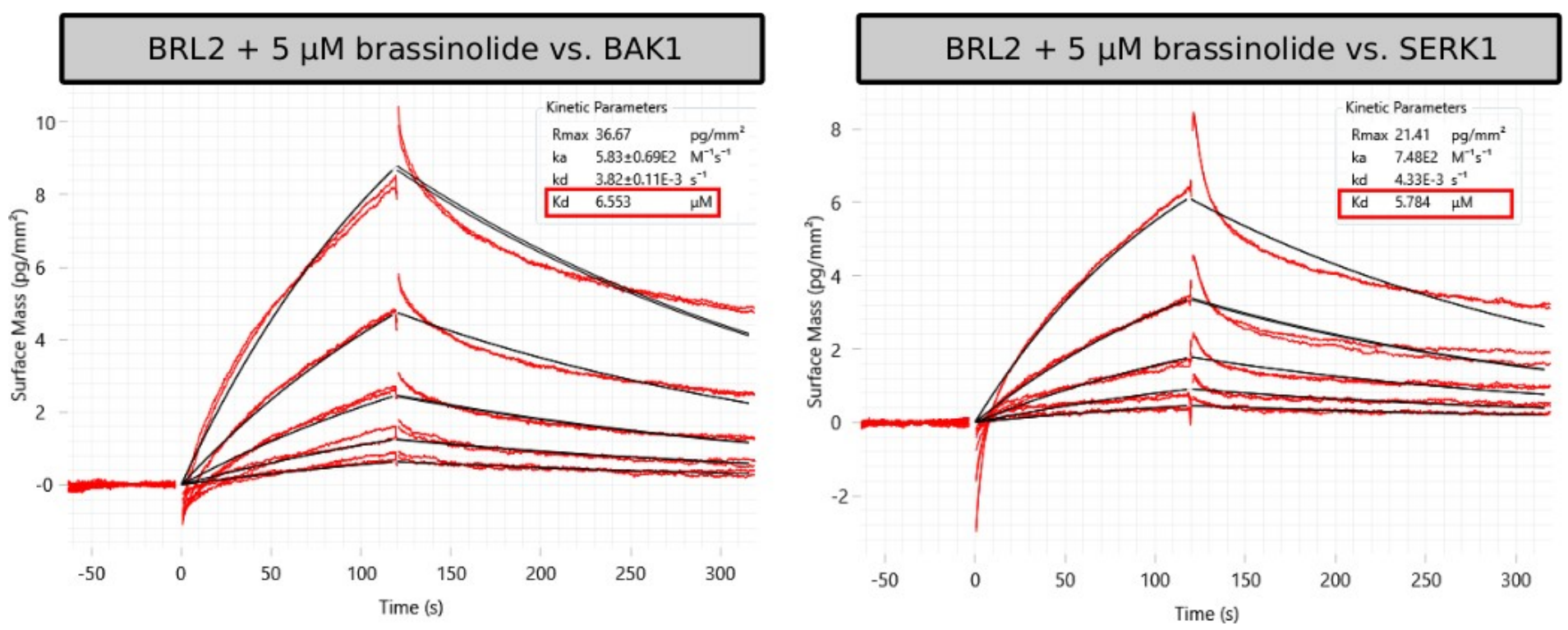

Reviewer Fig. 2. Grating-coupled interferomtry binding assay in the presence of a constant concentration of 5 µM brassinolide. The BRL2 ectodomain was coupled to the GCI chip and the isolated SERK1 and BAK1 ectodomains were used as analytes.

The phenotype of our *BRL2prom*::oBIR3-iBRL2 chimeric line further supports the idea that BRL2 can be activated by association with SERKs (see response to reviewer #3 below). We have previously demonstrated that the BIR ectodomain used in this construct specifically binds SERKs, but not the sequence-related NIK/CIK/CLERK family of co-receptor kinases in Arabidopsis (compare Fig. 6 in Hohmann et al., https://doi.org/10.1105/tpc.20.00138), suggesting that BRL2 can be activated by interaction with a SERK co-receptor (Supplementary Fig. 5g).

Following the reviewer's request, we have removed the following statements from the discussion section "Importantly, our quantitative BR binding assay identifies BRL2 as a functional BR receptor with a distinct ligand binding spectrum (Fig. 2c,d, Fig. 4b,c). BRL2 shares a conserved BR binding mechanism (Supplementary Fig. 3b) and weakly interacts with SERK co-receptors (Fig. 6b). The gain-of-function phenotype of oBIR3-iBRL2 receptor chimera (Fig. 4e) further supports the notion that BRL2 shares the SERK-based receptor activation mechanism[52] with BRI1[28,35,36], BRL1 and BRL3 (Fig. 6b). We speculate that the inability of BRL2 to replace BRI1 in *bri1* mutants[29,33] is caused by its distinct BR ligand binding spectrum (Fig. 4b,c), while its receptor activation mechanism and cytoplasmic signaling pathway appear to be conserved[60,61]. Taken together, BRL2, which emerged early on in land plant evolution, may represents an ancestral BR receptor[62]."

The deleted paragraph has been replaced by this statement (lines 420-422) "Our quantitative biochemical assays also suggest that BRL2 can bind BRs with medium affinity (Fig. 4e) and can

weakly interact with SERK co-receptors (Fig. 6b).", which accurately summarizes our in vitro findings without speculating on a potential function of BRL2 as a BR receptor, as requested.

3. The authors determined the apo structure of BRL2's extracellular domain and identified structural variations with BRI1 or BRL3. However, for the structural comparisons presented in Fig. 4a or Supplementary Fig. 3b, the figure legends suggest that these were carried out using the Alphafold-predicted model. Why not compared with the determined structure?

Our response: In the structure(s) of apo BRL2, the island domain of the receptor appears partially disordered, as previously reported for the apo structure of BRI1 (see Supplementary Fig. 7 in Hothorn et al. https://doi.org/10.1038/nature10153). Moreover, the BRL2 island domain engages in a domain-swapped interaction with a neighboring molecule. These structural features of BRL2 are highlighted in newly added figure panels Fig. 4a,b. The BRL2 Alphafold model presents the island domain bound in cis to the LRR core of BRL2 and thus likely mimics a brassinosteroid-bound conformation of the receptor. We therefore used the BRL2 Alphafold model for the structural superpositions with BRI1 (revised Fig. 4d) and BRL3 (newly added Supplementary Fig. 4c).

Moreover, would the observed structural variations contribute to the lower BR binding or the co-receptor binding of BRL2? This should be further clarified and investigated by the authors. In addition, one or more BR-bound structures of BRL2 would provide further insights into its ligand selectivity.

Our response: For the revision of this manuscript, we have attempted to determine structures of BRL2 bound to either brassinolide or castasterone by performing crystal soaking and co-crystallisation experiments. Soaking the tetragonal crystal for of BRL2 resulted in crystal cracking and decay, even in stabilizing solutions supplemented with sodium malonate. Co-crystallization of BRL2 in the presence of 50 µM brassinolide yielded a new, monoclinic crystal form diffracting up to 2.6 Å and with two molecules in the asymmetric unit, but with no discernible difference density for brassinolide. Soaking this crystal form with higher concentrations of brassinolide likewise resulted in crystal diffraction decay, so we are unable to report a ligand-bound structure of BRL2. The revised result section reads (lines 247-252) "We expressed and purified the BRL2 ectodomain from insect cells and solved its apo crystal structure to 2.6 Å resolution in two independent crystal forms (see Methods, Supplementary Table 1). The N-glycosylated BRL2 ectodomain adopts a superhelical architecture with 21 LRRs (compared to 25 in BRI1) with a more open conformation (Fig. 4a, Supplementary Fig. 4a). BRL2's island domain is partially disordered and participates in a domain-swapped crystallographic dimer (Fig. 4b, Supplementary Fig. 4b)."
The new data collection and refinement statistics have been included in revised Supplementary Table 1. The revised method section reads (lines 523-527) "A second, monoclinic crystal form for BRL2 developed in 22% (w/v) PEG 3,350, 0.2 M Na citrate, 0.1 M Bis-Tris pH 7.0 and in the presence of 50 µM brassinolide. Datasets were collected at beam line X06DA of the Swiss Light Source, Villigen, Switzerland, the monoclinic BRL2 dataset was collected at beam line ID30B of the European Synchrotron Radiation Facility, Grenoble, France (Supplementary Table 1).

An extended structural and sequence comparison of BRL2 and BRI1/BRL3 is now shown in newly added Fig. 4a,d, and in Supplementary Fig. 4c). A main sequence variation between BRL2 and BRI1/BRL1/3 is replacement of Y597 in BRI1 by Met557 in BRL2. The dissociation constant for brassinolide vs. the BRI1^{Y597M} mutant protein is shown in Fig. 5c, the corresponding mutant growth phenotypes in Fig. 5d-f. Both experiments suggest that Met557 is not a major determinant for BRL2's ligand binding properties when studied in isolation. A second major difference concerns Met657 in BRI1, which corresponds to a glutamate residue in BLR2. The BRI1^{M657E} mutant again has only a

moderately reduced binding affinity for brassinolide (Fig. 5b), suggesting that a combination of several amino acid differences between BRI1 and BRL2 may cause for its reduced binding affinity to brassinolide and other brassinosteroids. Consistently, a previous study, in which we systematically replaced all residue in BRI1 with the corresponding amino acids in BRL2 required the simultaneous mutation of at least three amino acids to observe a strong growth phenotype and five point mutations to see a drastic effect on brassinosteroid binding (Fig. 1 and 2 in Neubus Claus et al., https://doi.org/10.1093/plphys/kiad005). This is highlighted in the introduction (lines 85-87) "Notably, five simultaneous point mutations in the BR-binding pocket were required to completely abolish brassinolide binding in vitro, and to mimic the severe growth phenotype of *bri1* null alleles in vivo[46]." The revised statement in the discussion section reads (lines 401-404) "We identified a single point mutation, Trp564His, that results in a strong BR loss-of-function phenotype. This finding demonstrates that BRI1 can be effectively inactivated by a single amino acid substitution, rather than requiring the combination of multiple mutations as previously reported[46]."

4. The authors suggest that the agonistic effect of 2-deoxybrassinolide may be caused by its entering into the biosynthetic pathway. Why other intermediates cannot enter the pathway? No evidence or data is provided here.

Our response: We agree with the reviewer that the statement: "We speculate that the agonistic effect of 2-deoxybrassinolide may be caused by this metabolite entering the biosynthetic pathway, generating other, bioactive BRs (Fig. 6g and Fig. 1b)." is too speculative. We have removed it from the revised results section.

Minor points:
1. page 2, line 66, delete "BR_ligand_recognition_MH13.odt".

Our response: Corrected.

2. page 3, line 83, check the sentence, also page 4, line 122.

Our response: Corrected.

3. A sequence alignment of BRI1, BRL1, BRL2, and BRL3 would help to show the conservation in the binding pocket. Furthermore, according to Fig. 4a, several residues are unconserved besides the mentioned E614 site (page 9, line 262-263), like G662, Q638, M557 of BRL2. Could these variations lead to different binding affinities towards BR?

Our response: See above, a structure-based sequence alignment comparing BRIL2 with BRI1/BRL1/BRL3 has been added to Fig. 4 (Fig. 4c).

4. Fig. 3b, the acyl tail of 24-epibrassinolide fits bad into the density. The authors should further check that.

Our response: Please see the response to reviewer #1 above. The structure of 24-epibrassinolide has been re-refined, details of the ligand binding site are shown in newly added Supplementary Fig. 2.

5. Supplementary Table1, for the Ramachandran plot, allowed region is missing. The authors should also further check to avoid the outliers. Moreover, parameters for the data collection are missing.

<u>Our response:</u> We have added the suggested Ramachandran plot statistics (favored regions, allowed regions, outliers, all calculated with phenix.molprobity) to Supplementary Table 1. The revised Table 1 now also contains additional data collection statistics (data collection wavelength, highest resolution shell, R_{meas}, CC1/2, I/sigI, completeness and data redundancy for highest shell), per-chain B-factors for all brassinosteroid ligands, as well as the mean B-factors for the N-glyans, buffer and solvent molecules. Several structures have been refined with improved stereochemistry. All structures have now been released from the PDB (February 18, 2026).

--Reviewer #3:
This manuscript by Caregnato et al presents a detailed characterization of different brassinosteroid hormones and the BRI1 family receptors. Although the field mostly thinks of downstream compounds in the brassinosteroid biosynthetic pathway, such as brassinolide, as the active signaling molecules, the authors show that a vast array of brassinosteroid hormones in the 15 that they test can bind to the receptors. They provide structural, mutational, and in planta evidence of the specificity and consequences of these interactions. Overall, this is a highly relevant and exceptionally conducted study. It will be a must-read in the field and change the way that we think about which BRs are potentially bioactive. I have a few minor comments and suggestions.

1. The authors show binding of many BR intermediates and their concentrations in plants. Is there more that can be said from this about the combined signaling capacity based on these specificities and abundances? How do their results in terms of hormone and receptor affinities translate into how bioactive each compound is in vivo and what their potential roles in signaling are?

<u>Our response:</u> We have correlated the observed brassinosteroid binding activities with their behavious in bioassays (Fig. 3c) and with the abundance in seedlings (revised Fig. 3d) and with bioassays (Fig. 3c). Throughout our result and discussion section we have been careful to avoid the claim that our in vitro binding affinities for different BRs are directly linked to their biological activities in planta. Due to their extremely low concentration in plant tissues it is, to the best of our knowledge, impossible to quantity BR metabolite levels at cellular or even at sub-cellular resolution, so we cannot establish which of the compounds tested are present in the cell wall where they could interact with the receptor(s). We have extended our BR metabolite analysis, which now includes typhasterol, to three different genotypes (wild type, a *bri1* loss-of-function mutant and a constitutive active oBIR2-iBRI1 chimeric line). The data are presented in revised Fig. 3d, the corresponding statement in the result section now reads (lines 218-225) “We next compared BR levels in wild-type, *bri1* null[47] and oBIR3-iBRI1[52] constitutively active mutant seedlings. In the *bri1* mutant, brassinolide, castasterone and typhasterol levels were elevated by two to 3-fold, respectively, compared to wild type (Fig. 3d), as previously described[45]. This aligns with earlier findings that BR metabolic and catabolic gene expression is altered in *bri1* loss-of-function mutants[53], partly mediated by the BR-regulated transcription factor BZR1[40,54]. In oBIR3-iBRI1 seedlings, which display constitutive BR responses[52], brassinolide and castasterone levels were reduced compared to wild type (Fig. 3d).”
We have added a statement to our discussion section (lines 412-416) “Based on binding profiles (Fig. 2c,d, Fig. 6c,d), bioactivity data, and in vivo BR levels[11,48,50] (Fig. 3d), we propose that brassinolide and castasterone (C_{28}), 28-norbrassinolide (C_{27}), and possibly 28-homocastasterone (C_{29}) may act as physiological BR ligands in Arabidopsis, but it is presently unknown if and how these metabolites reach the cell wall compartment to interact with the ligand binding domains of BR receptors.” Please also see our response to reviewer #1 major comment #1.

2. It is claimed that BRL2 is a “medium affinity BR receptor” based on new binding data and a limited set of in vivo experiments. It’s commendable that the authors generated fresh brl2 CRISPR lines for

this analysis, yet it's still unclear if BRL2 has a functional role in vivo. Could they use the same construct to generate a bri1brl1brl2brl3 quadruple receptor mutant? It would be interesting to compare this to the bri1brl1brl3 triple mutant, which to my knowledge is thought to be nearly blind to BRs. Based on their claim, one would expect to see some responsiveness to high concentrations of hormones that bind BRL2 that remains in the triple mutant, but would be abolished in the quadruple mutant. If there is no genetic evidence or functional consequence of the role of BRL2 in mediating BR signaling, it's difficult to justify calling it a "medium affinity BR receptor". This could be softened.

Our response: We are currently generating a *brl1 brl2 brl3* CRISPR/Cas9 triple knock-out mutant to investigate potential roles of BRL2 in vascular development and function, in collaboration with a research group specializing in plant vascular biology. However, we consider such extensive functional analyses to be beyond the scope of the present manuscript, which focuses on the ligand specificity of BR receptors. We have therefore moved the revised functional analysis of BRL2 to the Supplementary Information. The data are presented in the newly generated Supplementary Fig. 4. The corresponding result section now reads (lines 268-284) "VH1/BRL2 has been characterized as a vascular receptor kinase involved in leaf vein patterning[34,55]. The previously described *brl2-1* mutant[55] showed wild type-like responses in BR hypocotyl growth and BES1 dephosphorylation assays (Supplementary Fig. 5a,b). In our hands, the leaf vein patterns of *brl2-1* plants were not significantly different from the Col-0 wild type (Supplementary Fig. 5c). Transcriptional *BRL2prom*::NLS-3xmVenus and translational *BRL2prom*::BRL2-mCitrine reporter lines confirmed the expression of BRL2 in vascular tissues across different developmental stages (Supplementary Fig. 5d,e). We generated a second *brl2-2* loss-of-function allele using CRISPR/Cas9 gene editing (Supplementary Fig. 5f), which like *brl2-1* was indistinguishable from wild type (Supplementary Fig. 5g). We have previously reported that receptor chimeras, in which the extracellular and transmembrane domains of BIR3 (oBIR3) are fused to the cytoplasmic kinase domain of a SERK-dependent receptor kinase (iRK), trigger constitutive, ligand-independent signaling responses[38,52]. A *BRL2prom*::oBIR3-iBRL2 chimera in Col-0 wild-type background had elongated, slender petioles and curly leaves, similar to BRI1^{sud1} gain-of-function mutants[24,47] (Supplementary Fig. 5g). However, the rosette phenotypes were milder than those of *BRI1prom*::oBIR3-iBRI1 chimeras[52] (Supplementary Fig. 5g)."
We have removed all statements relating to BRL2 being a BR receptor, as requested by the reviewer. Specifically, the following statement has been removed from the result section: "Our experiments suggest that BRL2 functions as a medium-affinity receptor for C_{28} BRs (Fig. 1b)" In line with this, the BR-binding pocket of BRI1, BRL1/3 and BRL2 share a high degree of structural and sequence conservation. The only notable exception is Glu614 in BRL2, which corresponds to Met657 and Ile642 in BRI1 and BRL3, respectively (Fig. 4a, Supplementary Fig. 3b)." The sub-title of the the corresponding paragraph has been revised accordingly (line 243) "BRL2 binds BRs with medium affinity in vitro". The toned-down summary of the BRL2 result section reads (lines 281-283) "These experiments suggest that BRL2 may have the capacity to initiate a SERK-dependent signaling cascade, yet its physiological ligand and function remain to be characterized."
The following statement has been added to the revised discussion section (lines 420-422) "Our quantitative biochemical assays also suggest that BRL2 can bind BRs with medium affinity (Fig. 4e) and can weakly interact with SERK co-receptors (Fig. 6b)."

3. The authors also generated a BRL2:NLS-Venus transcriptional reporter. What does a translational line look like and does it give any clues into functional relevance?

Our response: We have generated the requested translational reporter line *BRL2prom*::BRL2-mCitrine, which is shown in Supplementary Fig. 5e. The translational reported confirms the presence of BRL2 in the vasculature. The corresponding statement in the result section reads (lines 272-274)

"Transcriptional *BRL2prom*::NLS-3xmVenus and translational *BRL2prom*::BRL2-mCitrine reporter lines confirmed the expression of BRL2 in vascular tissues across different developmental stages (Supplementary Fig. 5d,e)." The corresponding statement in the method section reads (lines 560-563) "The coding sequence of BRL2 was introduced into *pEN-L1(MCS)-L2*[78] by Gibson assembly, and subsequently combined with *L4-BRL2prom-R1* and *mCitrine-pDONR P2R P3* in destination vector *pB7m34GW*[81] to produce the translational reporter construct *BRL2prom::BRL2-mCitrine.*"

4. I'm a bit confused about what the authors hoped to learn from the pBRL2-oBIR3-iBRL2 experiments. Given that these constructs are activated independent of ligand, they can only report on potential signaling activity, but don't yield insight as to what the receptor is actually doing in vivo. Please clarified.

Our response: We and other have used the constitutively active oBIR3-iRK chimera to study the effects of constitutive active RK pathways in a tissue-specific context (Hohmann et al., https://doi.org/10.1105/tpc.20.00138, recent application in Xiao et al. https://doi.org/10.1073/pnas.2510538122). Analysis of our *BRL2prom*::oBIR3-iBRL2 chimera suggests that BRL2 has the ability to signal when present in its normal expression domain (Supplementary Fig. 5g). The phenotype of our *BRL2prom*::oBIR3-iBRL2 chimeric line further supports the idea that BRL2 can be activated by association with SERKs (see response to reviewer #3 below). We have previously demonstrated that the BIR ectodomain used in this construct specifically binds SERKs, but not the sequence-related NIK/CIK/CLERK family of co-receptor kinases in Arabidopsis (compare Fig. 6 in Hohmann et al., https://doi.org/10.1105/tpc.20.00138), suggesting that BRL2 can be activated by interaction with a SERK co-receptor (Supplementary Fig. 5g). As requested by reviewer #2 and reviewer #3 we have moved these findings to the supplement and toned-down our discussion section. We prefer to keep the BRL2 in vivo data in the revised manuscript, as we have already sent the *brl2-2* CRISPR/Cas9 allele and the *BRL2prom*::BIR3-iBRL2 chimeric lines to several labs that requested this material following the publication of our Biorxiv preprint (https://doi.org/10.1101/2025.08.08.669299).
